# Rapid evolution of *BRCA1* and *BRCA2* in humans and other primates

**DOI:** 10.1186/1471-2148-14-155

**Published:** 2014-07-11

**Authors:** Dianne I Lou, Ross M McBee, Uyen Q Le, Anne C Stone, Gregory K Wilkerson, Ann M Demogines, Sara L Sawyer

**Affiliations:** 1 Department of Molecular Biosciences, The University of Texas at Austin, Austin, TX 78712, USA; 2 School of Human Evolution and Social Change, Arizona State University, Tempe, AZ 85281, USA; 3 Department of Veterinary Sciences, Michale E. Keeling Center for Comparative Medicine and Research, The University of Texas MD Anderson Cancer Center, Bastrop, TX 78602, USA

**Keywords:** DNA damage response, Simian primates, Cell cycle, Positive selection

## Abstract

**Background:**

The maintenance of chromosomal integrity is an essential task of every living organism and cellular repair mechanisms exist to guard against insults to DNA. Given the importance of this process, it is expected that DNA repair proteins would be evolutionarily conserved, exhibiting very minimal sequence change over time. However, *BRCA1*, an essential gene involved in DNA repair, has been reported to be evolving rapidly despite the fact that many protein-altering mutations within this gene convey a significantly elevated risk for breast and ovarian cancers.

**Results:**

To obtain a deeper understanding of the evolutionary trajectory of *BRCA1*, we analyzed complete *BRCA1* gene sequences from 23 primate species. We show that specific amino acid sites have experienced repeated selection for amino acid replacement over primate evolution. This selection has been focused specifically on humans and our closest living relatives, chimpanzees (*Pan troglodytes*) and bonobos (*Pan paniscus*). After examining *BRCA1* polymorphisms in 7 bonobo, 44 chimpanzee, and 44 rhesus macaque (*Macaca mulatta*) individuals, we find considerable variation within each of these species and evidence for recent selection in chimpanzee populations. Finally, we also sequenced and analyzed *BRCA2* from 24 primate species and find that this gene has also evolved under positive selection.

**Conclusions:**

While mutations leading to truncated forms of BRCA1 are clearly linked to cancer phenotypes in humans, there is also an underlying selective pressure in favor of amino acid-altering substitutions in this gene. A hypothesis where viruses are the drivers of this natural selection is discussed.

## Background

Defects in the *BRCA1* or *BRCA2* genes are responsible for most hereditary forms of breast cancer and account for as many as 10% of all breast cancer cases [1]. Women with a strong family history of cancer who possess a harmful *BRCA1* or *BRCA2* allele are at high risk for developing breast cancer within their lifetime (80% and 60%, respectively) [2,3]. In addition, *BRCA1* mutation carriers have a 30-40% chance of developing ovarian cancer, while *BRCA2* mutations also increase the risk of ovarian, pancreatic, prostate, and male breast cancer [2]. Cancers occur when heterozygous individuals experience a somatic loss of heterozygosity event at the *BRCA1* or *BRCA2* locus, leaving only the abnormal allele intact. Because both gene products play a critical role in key cellular processes such as DNA repair, cell cycle control, and transcriptional regulation, it is clear why inactivating mutations are so detrimental. The importance of these proteins is further evidenced by the fact that both *BRCA1* and *BRCA2* null mice are embryonic lethal [4].

Given their indispensible functions in maintaining the integrity of the genome, one might expect strict evolutionary conservation of *BRCA1* and *BRCA2* over time. Indeed, some regions of *BRCA1* have experienced purifying selection strong enough to operate even on synonymous mutations [5]. However, contrary to this line of reasoning, a number of groups have documented the rapid evolution of *BRCA1*[6-11] and *BRCA2*[10] in mammals. Rapid evolution occurs when a gene experiences positive natural selection for new, advantageous mutations that arise in a population. Because advantageous mutations commonly involve a change in protein sequence (non-synonymous mutations), recurrent rounds of positive selection in a gene lead to rapid evolution of the encoded protein sequence over time. For *BRCA1*, the evolutionary rate was particularly elevated on the branches leading to humans and chimpanzees (*Pan troglodytes*) [6]. The identification of this signature in *BRCA1* suggests that some alleles and polymorphisms currently circulating within the human population may offer a selectable advantage. However, both the cause and consequence of this unexpected mode of evolution seen in *BRCA1* remain unknown.

Here, we report an extensive evolutionary analysis of the primate *BRCA1* gene. In previous studies of *BRCA1* evolution, only exon 11 was examined with a limited number of primate species included in the analyses [6-11]. To extend previous studies, we have generated full-length *BRCA1* sequences for 17 additional primate species. Using this more extensive dataset, we validate the finding of positive selection in humans and their closest ape relatives (in our study, chimpanzees and also bonobos (*Pan paniscus*)). We also show that specific codons in *BRCA1* have experienced recurrent positive selection over evolutionary time, both within and outside of exon 11, resulting in a small number of highly variable residue positions in an otherwise highly conserved protein. In addition, we sequenced exon 11 of *BRCA1* from populations of chimpanzee, bonobo, and rhesus macaque (*Macaca mulatta*) individuals and found that several unique polymorphisms exist within these populations. Two polymorphisms in the chimpanzee population were found to be in Hardy-Weinberg disequilibrium suggesting that selection may still be operating on this gene in modern times. Lastly, exon 11 of *BRCA2*, another important genetic determinant for hereditary breast and ovarian cancers, was also sequenced from diverse primate species. This gene also bears the surprising signature of positive selection. It is unclear why these critical genes bear this unusual evolutionary signature, but we present one possible hypothesis involving interactions between DNA repair proteins and viruses.

## Results

### *BRCA1* is evolving under positive selection in primates

**Figure 1:**
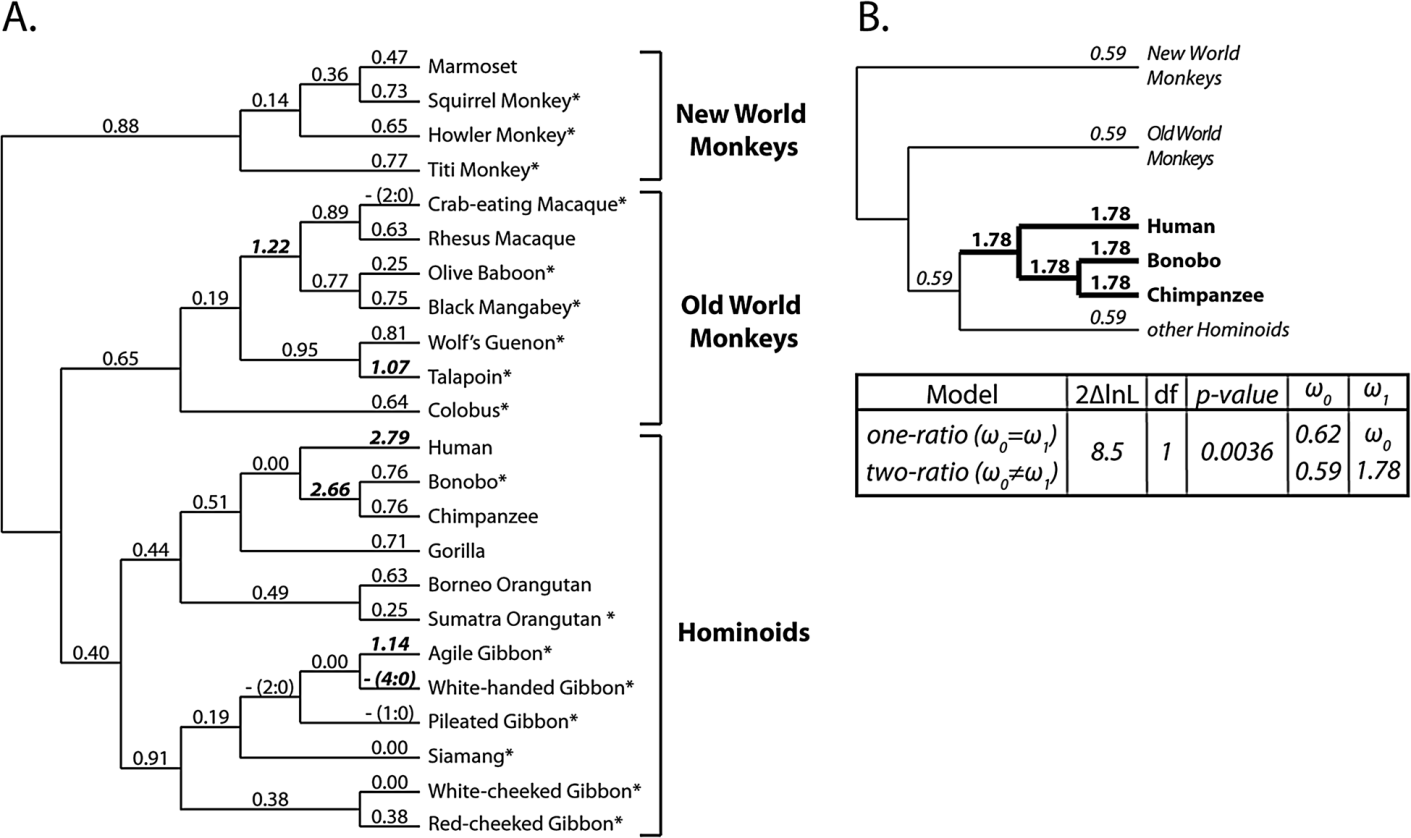
**Evolution of *BRCA1 *over the course of primate speciation. A**. dN/dS values for each branch of the primate phylogeny were calculated using the free-ratio model in PAML [13]. Branches exhibiting dN/dS values > 1 are shown in bold italics. Dashes (-) represent branches where zero synonymous substitutions are predicted to have occurred. On these branches, dS = 0 and dN/dS can therefore not be calculated. In these instances, the numbers of non-synonymous (N) and synonymous (S) substitutions predicted to have occurred along each branch are indicated in parentheses (N:S). Of these, branches that experienced 4 or more non-synonymous substitutions are in bold italics. Asterisks indicate new sequences generated in this study. **B**. The human, bonobo, and chimpanzee clade was isolated and dN/dS values were calculated using the one-ratio and two-ratio models in PAML. The two-ratio model was a better fit as determined by the likelihood ratio test shown in the box. ω0 is the calculated dN/dS for all branches under the one-ratio model, or for background branches under the two-ratio model, and ω1 is the dN/dS for the isolated branches in the two-ratio model.

To expand our understanding of the positive selection shaping *BRCA1* in primates, we obtained cell lines from 17 simian primate species, harvested total RNA, and created cDNA libraries. From these, the 5.6 kilobase full-length coding region of *BRCA1* was sequenced. These sequences were combined with full-length *BRCA1* sequences from six primate species with available genome projects, creating an alignment of 23 full-length *BRCA1* sequences. 17 out of the 23 full-length sequences have never before been analyzed (asterisks in Figure 1A).

The type of selection that a gene has experienced can be inferred from its rate of accumulation of non-synonymous (changing the encoded amino acid; denoted dN) and synonymous (silent; dS) substitutions over time. Protein-altering mutations are far less likely to be tolerated than synonymous mutations, and so dN/dS < < 1 for the vast majority of genes encoded by human and other mammalian genomes [12]. Some genes, such as pseudogenes, evolve neutrally with dN/dS ~ 1 because there is not strong selection for or against new mutations in these genes. Finally, selection in favor of non-synonymous mutations results in a dN/dS > 1. These genes are classified as being under positive selection, and are experiencing continued selection for “innovation” at the protein sequence level. In these genes, not only has the penalty against protein-altering mutations been relaxed, but this very type of mutation is being selectively retained. Using PAML [13], we fit the full-length *BRCA1* alignment (Additional file 1) to models of positive selection where a subset of codons is allowed to evolve with dN/dS > 1 (M2a, M8) and to null models not allowing positive selection (M1a, M7, M8a). Likelihood ratio tests revealed that the dataset fit the positive selection models significantly better than the null models (p < 0.05, Table 1). Thus, *BRCA1* has experienced selection in favor of non-synonymous mutations over the speciation of simian primates.

**Table 1:** **PAML Analysis of *BRCA1 *** **and *BRCA2***

**M1a**-**M2a**	**ω**_**0**_^**a**^	**codon freq.**^**b**^	**2ΔlnL**^**c**^	**df**^**c**^	**p**-**value**^**c**^	**M1a**-**M2a**	**ω**_**0**_^**a**^	**codon freq.**^**b**^	**2ΔlnL**^**c**^	**df**^**c**^	**p**-**value**^**c**^
***BRCA1***	0.4	f61	10.0	2	0.0066	***BRCA2***	0.4	f61	21.3	2	<0.0001
	0.4	f3x4	6.1	2	0.0466		0.4	f3x4	16.1	2	0.0003
	1.6	f61	10.0	2	0.0066		1.6	f61	21.3	2	<0.0001
	1.6	f3x4	6.1	2	0.0466		1.6	f3x4	16.1	2	0.0003
**M7**-**M8**	**ω**_**0**_^**a**^	**codon freq.**^**b**^	**2ΔlnL**^**c**^	**df**^**c**^	**p**-**value**^**c**^	**M7**-**M8**	**ω**_**0**_^**a**^	**codon freq.**^**b**^	**2ΔlnL**^**c**^	**df**^**c**^	**p**-**value**^**c**^
***BRCA1***	0.4	f61	10.6	2	0.0049	***BRCA2***	0.4	f61	23.3	2	<0.0001
	0.4	f3x4	6.2	2	0.0447		0.4	f3x4	18.6	2	<0.0001
	1.6	f61	10.6	2	0.0049		1.6	f61	23.3	2	<0.0001
	1.6	f3x4	6.2	2	0.0447		1.6	f3x4	18.6	2	<0.0001
**M8a**-**M8**	**ω**_**0**_^**a**^	**codon freq.**^**b**^	**2ΔlnL**^**c**^	**df**^**c**^	**p**-**value**^**c**^	**M8a**-**M8**	**ω**_**0**_^**a**^	**codon freq**.^**b**^	**2ΔlnL**^**c**^	**df**^**c**^	**p**-**value**^**c**^
***BRCA1***	0.4	f61	10.1	1	0.0015	***BRCA2***	0.4	f61	19.9	1	<0.0001
	0.4	f3x4	6.2	1	0.013		0.4	f3x4	15.1	1	0.0001
	1.6	f61	10.1	1	0.0015		1.6	f61	19.9	1	<0.0001
	1.6	f3x4	6.2	1	0.013		1.6	f3x4	15.1	1	0.0001

^**a**^Initial seed value for ω (dN/dS).

^**b**^Model of codon frequency.

^**c**^Twice the difference in the natural logs of the likelihoods (2 × ΔlnL) of the two models being compared (a model that allows positive selection (M2a or M8) is compared to a null model (M1a, M7, M8a)). This value is used in a likelihood ratio test along with the degrees of freedom (df). The p-value indicates the confidence with which the null model can be rejected.

We next estimated dN/dS values on each branch on the primate evolutionary tree using the free-ratio model in PAML. As expected, most branches exhibited a dN/dS < 1 (Figure 1A). The branch leading to humans had the most elevated signal with a dN/dS of 2.79. The second highest value of dN/dS on the *BRCA1* tree is found on the branch leading to the last common ancestor of bonobos and chimpanzees, with a dN/dS of 2.66. Because the free-ratio model is highly parameterized, we next compared one-ratio and two-ratio models to determine whether selection has differentially affected the human, chimpanzee, and bonobo clade. As shown in Figure 1B, our simian primate dataset fit the two-ratio model significantly better than the one-ratio model, with the human, chimpanzee, and bonobo clade exhibiting a dN/dS of 1.78, while all other branches had a dN/dS of 0.59. In summary, our extended primate dataset shows that *BRCA1* is experiencing positive selection, and that the most intense selection has operated on the human/chimpanzee/bonobo clade.

**Figure 2:**
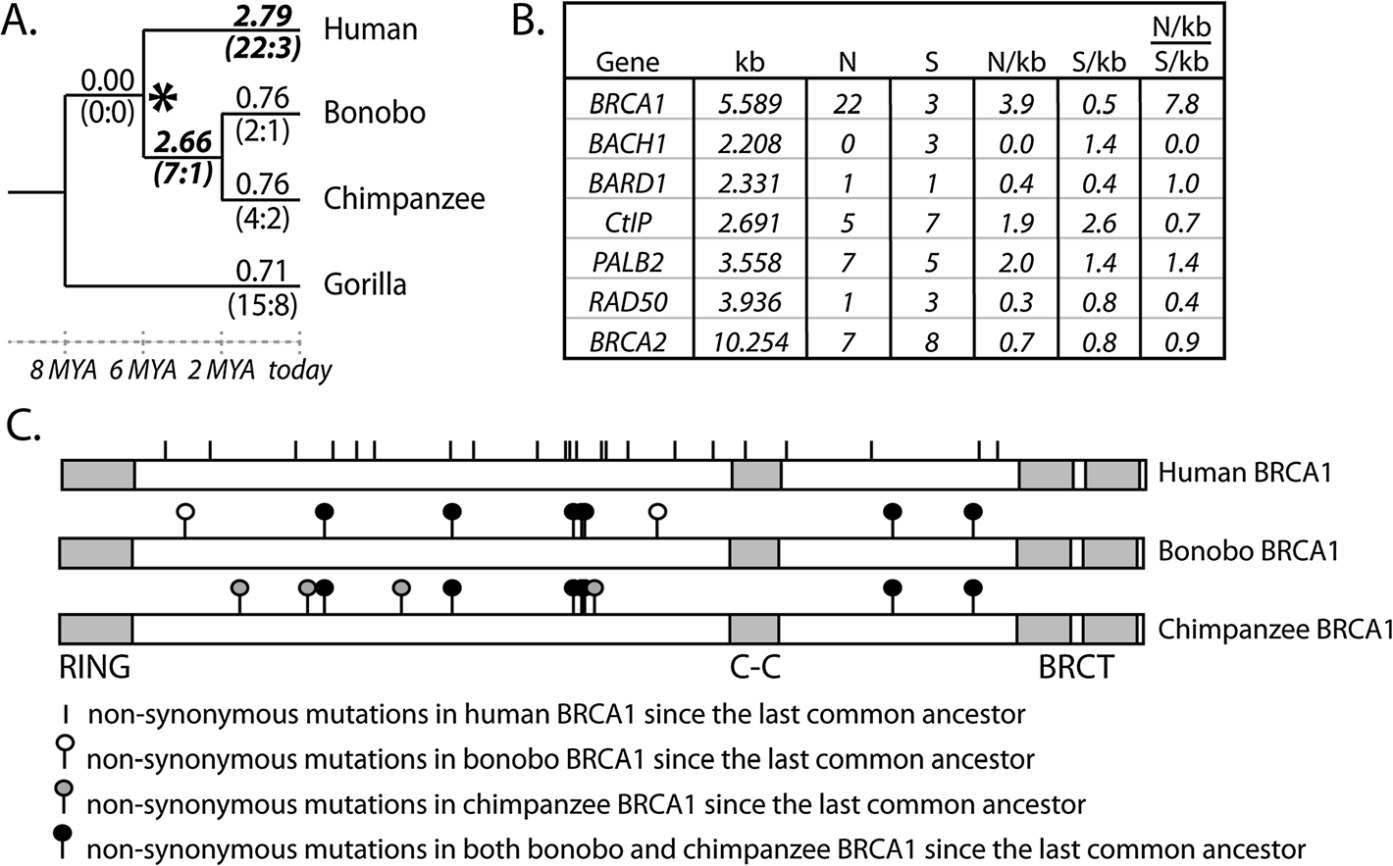
***BRCA1 *evolution in the human**, **bonobo**, **and chimpanzee clade. A**. dN/dS values for *BRCA1* were calculated on each branch of the primate tree using the free-ratio model in PAML. dN/dS values > 1 are shown in bold italics. The numbers of non-synonymous (N) and synonymous (S) substitutions predicted to have occurred along each branch are indicated in parentheses (N:S). The asterisk represents the last common ancestor of humans, bonobos, and chimpanzees. MYA, million years ago. **B**. The number of human-specific non-synonymous (N) and synonymous (S) substitutions in *BRCA1* and other genes encoding BRCA1-interacting proteins. The length of each gene is shown in kilobases (kb). Non-synonymous and synonymous substitutions are shown as number of substitutions per kilobases (N/kb and S/kb, respectively). An “enrichment ratio” of N/kb over S/kb was also calculated. **C**. A domain diagram of BRCA1 is shown with the RING domain, coiled-coil domain (C-C), and BRCT domains indicated. On this are superimposed all of the non-synonymous substitutions predicted to have occurred in the tree shown in panel A since the divergence of humans, bonobos, and chimpanzees from their last common ancestor (asterisk in A). Vertical lines indicate substitutions specific to humans, lines with white circles are substitutions specific to bonobos, and lines with grey circles are substitutions specific to chimpanzees. Lines with black circles indicate substitutions common to both bonobos and chimpanzees.

Based on a comparison of extant and predicted ancestral sequences, humans are estimated to have accumulated 25 substitutions in the *BRCA1* gene since their divergence from chimpanzees and bonobos six million years ago, 22 of which are non-synonymous (Figure 2A). In order to understand how unusual this is, we looked at the evolution of other genes, specifically ones encoding BRCA1-interacting proteins, along the branch leading to humans. Because we do not have extended sequence sets for all of these genes, we took a simpler approach. For each gene, we aligned the human, chimpanzee, and gorilla sequences and manually counted the number of human-specific substitutions (any position where the human gene sequence differs from both the chimpanzee and gorilla gene sequence). These were categorized as non-synonymous (N) or synonymous (S) based on how they affected the codon in which they were found. When these values are normalized to gene size, *BRCA1* has the highest enrichment of non-synonymous substitutions [(N/kb)/(S/kb)]. Care must be taken in comparing this metric between genes, because different genes have different equilibrium codon frequencies, and therefore have different mutational opportunities for synonymous and non-synonymous mutations. However, the *BRCA1* gene has an enrichment ratio that is more than 4-fold higher than any of the other genes shown (Figure 2B).

*BRCA1* encodes a 220 kDa protein with two conserved domains: an N-terminal RING domain and two tandem C-terminal BRCT domains (Figure 2C). The RING domain has E3 ubiquitin ligase activity that is essential in the DNA damage response. The BRCT motifs function as a protein-protein interaction module that binds phosphorylated proteins involved in DNA repair, cell cycle control, chromatin remodeling, and transcription. There is also a coiled-coil region between these two domains. Interestingly, all but one of the non-synonymous substitutions predicted to have occurred in the human/bonobo/chimpanzee clade fall outside of these known structural motifs (Figure 2C).

### Human variation at selected sites in *BRCA1*

**Figure 3:**
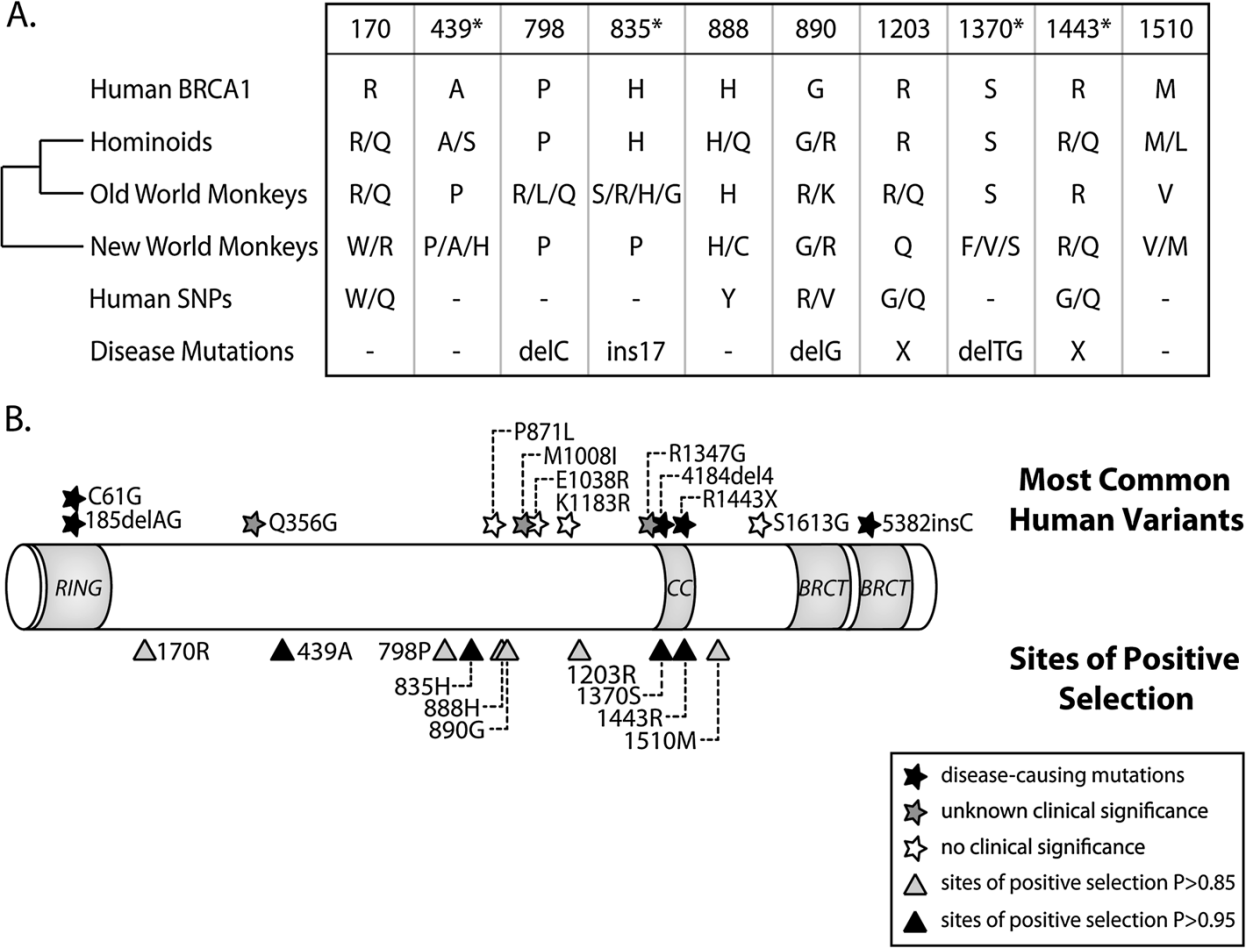
**Specific codons in *BRCA1 *have experienced positive selection during primate speciation. A**. Shown are the ten codons that have evolved under positive selection (dN/dS > 1) in primates with a P > 0.85. Codons with a P > 0.95 are indicated with asterisks. The amino acids encoded at these positions in human BRCA1 are shown, along with those found in hominoids, old world monkeys, and new world monkeys. In addition, human SNPs and disease mutations also found at these sites are listed. X refers to a single nucleotide mutation that results in a termination codon. **B**. A domain diagram of BRCA1 is shown with the RING domain, coiled-coil domain (CC), and BRCT domains. The triangles at the bottom represent sites of positive selection (grey - P > 0.85, black - P > 0.95). The 12 most common human variants recorded in the BIC are shown at the top of the diagram as stars. The black stars indicate disease-causing mutations, white stars represent variants with no known clinical significance, and grey stars are those with unknown significance.

The M8 model allows a class of codons to evolve under positive selection (dN/dS > 1). 10 codons were identified as belonging to this class with a high posterior probability (P = 0.85 or above). These codons do not lie in the region of *BRCA1* where it was previously reported that selection might be acting against synonymous mutations [5], potentially given rise to a false signature of dN/dS > 1. Instead, all 10 sites show high variability between primate species at the protein level, often encoding very dissimilar amino acids (first four rows in Figure 3A). Next, these positively selected codon positions were examined for variability within the human population. The Breast Cancer Information Core (BIC, http://research.nhgri.nih.gov/bic/) is a repository of human *BRCA1* polymorphisms. Using this database, we identified single nucleotide polymorphisms (SNPs) at amino acid sites 170, 888, 890, 1203, and 1443 (Figure 3A). At four out of these five sites (position 888, 890, 1203, and 1443), we find that some human *BRCA1* alleles encode a unique amino acid not observed in any of our primate sequences. In addition, SNPs known to cause human disease occur in six out of 10 sites. In all cases, these disease-linked SNPs are not amino acid-altering mutations, but rather more radical frame-shifting or nonsense mutations (Figure 3A). In particular, nonsense mutations occurring in codon 1443 are among the most common mutations documented in the BIC. In Figure 3B, all 10 sites of positive selection were mapped onto a domain diagram of BRCA1 (bottom) along with the most common human non-synonymous SNPs found in the BIC (top). As described previously for mutations accumulated in the human/chimpanzee/bonobo clade, all but one of the positively selected residues (1370S in the coiled-coil domain) lie outside of any known structural motifs. In summary, the 10 codon positions identified in this analysis are highly variable between primate species and within the human population, and are involved in the etiology of cancers associated with this gene. Disease-associated SNPs at these sites tend to be radical, protein-truncating mutations. However, a presumably distinct phenomenon appears to be driving selection in favor of non-synonymous point mutations at these positions.

### *BRCA1* variation in other primate populations

So far, we have documented sequence differences between the BRCA1 proteins of different primate species. We have shown that non-synonymous substitutions are accumulating in *BRCA1* faster than expected under constrained, or even neutral, evolution. We next wished to explore whether positive selection is still acting on *BRCA1* in modern populations. There is already evidence that this is true in the human population, because several *BRCA1* SNPs have been found to depart from Hardy-Weinberg equilibrium in European populations [14,15] and in Australia [6]. We wished to determine if the same might be true in bonobo and chimpanzee populations. We amplified and sequenced the largest *BRCA1* exon, exon 11 which is ~3.4 kilobases and comprises ~61% of the *BRCA1* coding region, from the genomic DNA of seven bonobo and 44 chimpanzee individuals (Table 2). In bonobos, we found nine polymorphic sites, eight of which were single nucleotide polymorphisms (SNPs), with three of these being non-synonymous. Eight of the SNPs were in Hardy Weinberg equilibrium. Interestingly, one bonobo individual was also homozygous for a seven amino acid deletion (Δ1058-1064) (Table 2). Hardy-Weinberg equilibrium was rejected for this polymorphism, although the support was weak and did not survive correction for multiple testing (Table 2). The chimpanzee sequence set revealed nine SNPs, seven of which were non-synonymous. Interestingly, in this larger sample set (n = 44), three of the non-synonymous SNPs were found to be in Hardy Weinberg disequilibrium, suggesting that selection is acting either for (E309K and G590S) or against (G1077R) these mutations. The support for one of these (E309K) was weak and did not survive correction for multiple testing (Table 2). It is particularly intriguing to see that humans also share with chimpanzees this same S/G SNP at position 590. In both the bonobo and chimpanzee populations, all synonymous SNPs were in Hardy-Weinberg equilibrium.

**Table 2:** **SNP Analysis of *BRCA1 *** **in Bonobo**, **Chimpanzee**, **and Rhesus Macaque Individuals**

**Species**	**SNPs**^**a**^	**Genotype**			**p**-**value**^**b**^	**Human**^**c**^	**Human Polymorphisms**	
		**AA**	**AB**	**BB**			**BIC**^**d**^	**1000 genomes**^**e**^
Bonobo n = 7	I493L	6	1	0	0.841	I		
	T582M	6	1	0	0.841	T		
	L833L	4	3	0	0.471	L	dupAAGTATCCAT*	
	V1047V	5	1	1	0.128	V		
	G1048G	5	1	1	0.128	G	G1048D, G1048V, G1048G	G1048D, G1048V, G1048G
	T1051I	5	1	1	0.128	T		
	Δ1058-1064^HWD^	6	0	1	0.008			
	V1061V	6	1	0	0.841	I	delA*	delA*
	G1062G	6	1	0	0.841	G		
Chimpanzee n = 44	E309K^HWD^	19	14	11	0.023	K	K309T	K309Q, K309T
	E427K	34	9	1	0.663	E		
	S578S	40	4	0	0.752	S	S578Y	S578Y
	G590S^HWD^	20	12	12	*0.004*	S	S590G	S590G
	K731E	19	16	9	0.122	K	delAGAAG*	delAGAAG*
	I925T	34	9	1	0.663	I	I925L	I925V, I925L, insT*
	S1042S	41	3	0	0.823	S		
	G1077R^HWD^	42	1	1	*1.4E*-*5*	G		G1077W, G1077G
	G1100E	20	16	8	0.155	G		
Rhesus n = 44	A225A	42	2	0	0.888	A		
	N375S	43	1	0	0.920	N	delA*, N376S	delA*, N376S
	R466R	42	2	0	0.888	K	K467X*	K467X*
	T487S	43	1	0	0.920	T	insA*	insA*
	N684N	29	14	1	0.647	N		
	V739M	38	6	0	0.624	V	V740L	V740L, insA*
	D773G	29	15	0	0.173	G		
	D852D	40	4	0	0.752	D	insA*	insA*
	N923H	40	4	0	0.752	N		
	K936K	40	4	0	0.752	K		
	A1167E	40	4	0	0.752	A		
	Q1203R	29	14	1	0.647	R	R1203Q, R1203G, R1203X*	R1203Q, R1203G, R1203X*

^**a**^Numbering refers to the amino acid position in the respective primates. In the case of rhesus macaques, amino acids 375 to 936 correspond to amino acids 376 to 937 in humans.

^**b**^p-values were calculated using a chi-squared test with a df = 1. A p-value cutoff (after Bonferroni correction) < 0.0056, 0.0056, and 0.0042 for bonobos, chimpanzees, and rhesus macaque, respectively, was considered statistically significant. Tests that survived this correction have the p-value listed in italics.

^**c**^Amino acid found in the human BRCA1 protein at each of the positions listed.

^**d**^Human variants found at the positions indicated in the Breast Cancer Information Core.

^**e**^Human variants found at the positions indicated in the 1000 Genomes database.

^*^Known human disease-causing variant.

^**HWD**^ SNPs found to be in Hardy-Weinberg Disequilibrium.

We also sequenced exon 11 from 44 rhesus macaque individuals. Rhesus macaques are not part of the human/chimpanzee/bonobo clade and are instead distantly-related members of the Old World monkey clade (Figure 1A). In these macaques, we found 12 SNPs in *BRCA1*, with seven being non-synonymous (Table 2). This includes a SNP found at position 1203, a site of positive selection in the inter-species dataset. This codon is also the site of a known disease-linked mutation in humans; however, the cancer-linked SNP at this position introduces a stop codon. Nonetheless, all of these are in Hardy-Weinberg equilibrium.

Caution must be used when interpreting signatures of selection acting on polymorphisms in primate populations. When sampling primates, it is not possible to get completely random and non-related population sets. Deviations from Hardy-Weinberg equilibrium may occur due to factors other than selection. Reasons for falsely rejecting Hardy Weinberg equilibrium include 1) non-random mating, 2) small population sizes which magnify the effects of genetic drift, 3) introduction of new alleles, 4) population subdivision or admixture, 5) biases in sequencing errors, and 6) linkage disequilibrium with another locus under selection. Because the chimpanzee population consists of individuals from two different subspecies, admixture could plausibly lead to rejection of Hardy Weinberg equilibrium.

We also performed the McDonald-Kreitman and Tajima’s D tests on our datasets (data not shown). The tests were not significant and therefore do not support selection acting on any of these polymorphisms. False conclusions in this test can again result from a population with hidden structure. In summary, while the analyses using the simian primate dataset consisting of 23 species suggest that recurrent positive selection has been acting on *BRCA1* over the course of several million years, the Hardy-Weinberg equilibrium tests performed here and by others indicate that selection is acting on modern day humans, and possibly also chimpanzees.

### *BRCA2* is also evolving under positive selection in primates

Because of the rapidly evolving nature of *BRCA1*, we also completed an evolutionary analysis of *BRCA2*, another strong determinant for hereditary breast and ovarian cancer. Although *BRCA2* has been shown to be under positive selection, only a small number of primate species was included in this study [10]. We sequenced the ~5 kilobase exon 11 from 18 primate species. Exon 11 is the largest of 27 exons and encodes about 50% of the entire BRCA2 protein. The sequences, along with six additional sequences from available genome projects, were assembled into a multiple alignment (Additional file 2). We fit the alignment to positive selection and null models as described above. The positive selection models were again a significantly better fit to the sequence set than the null models, with a p value ≤ 0.0003 (Table 1). In summary, *BRCA2* is under positive selection in primates as well, although this signature appears not to be concentrated on the human/chimpanzee/bonobo clade (Additional file 3).

**Figure 4:**
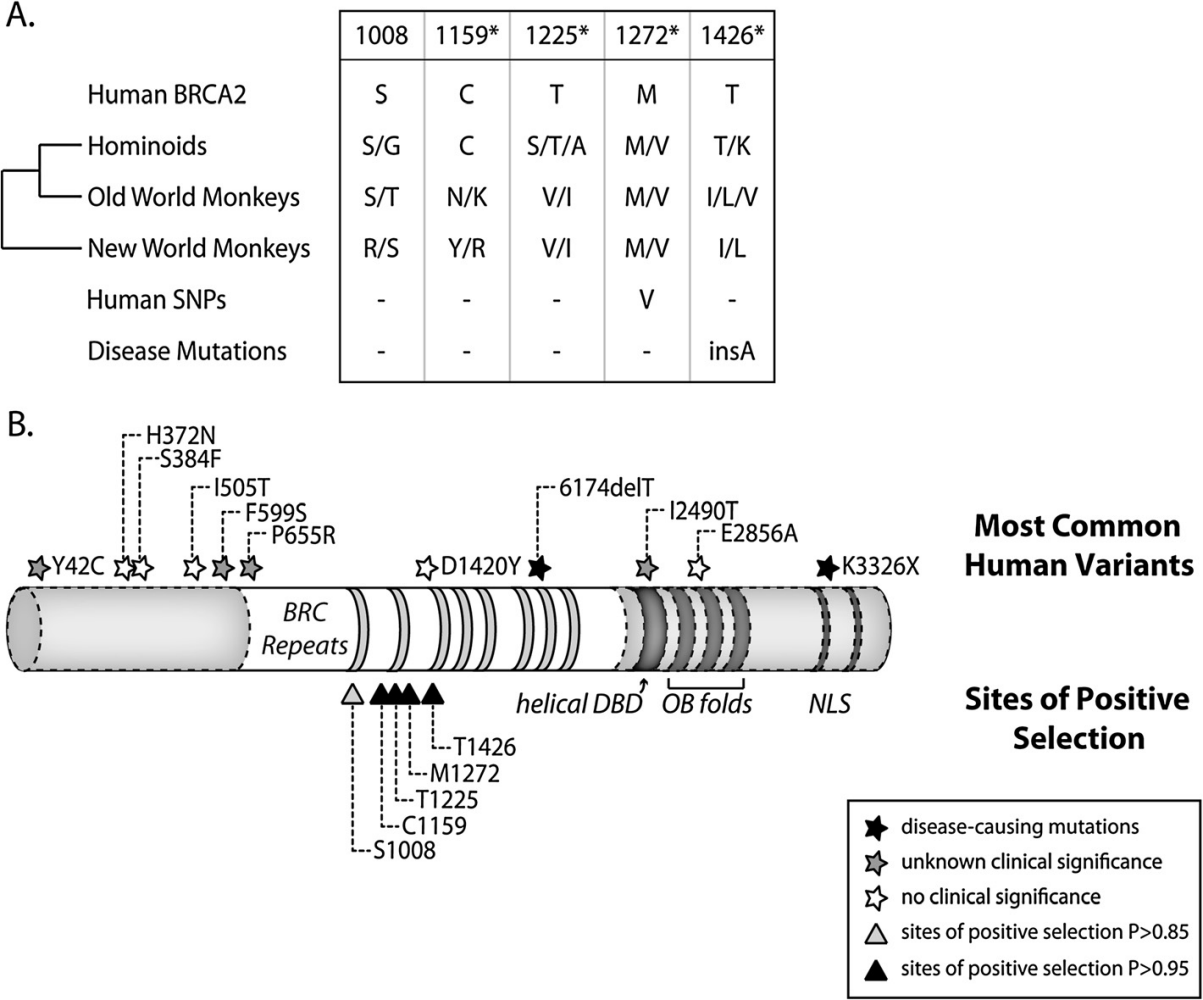
**Codons in exon 11 of *BRCA2 *that have experienced positive selection in primates. A**. 5 codons in exon 11 of *BRCA2* were found to be under positive selection in primates. All sites had a P > 0.95 (indicated with asterisks) except for S1008 (P = 0.9). The amino acid encoded by human *BRCA2* at each of these codons is shown. The amino acids encoded by hominoids, old world monkeys, and new world monkeys are also shown. Human SNPs and disease mutations deposited to the BIC are listed at the bottom. **B**. A domain diagram of BRCA2 is depicted with the 8 BRC repeats, helical DNA binding domain (helical DBD), OB folds, and nuclear localization signals (NLS). Only exon 11 was sequenced in this study (section in white). The sites of positive selection are represented as triangles at the bottom of the diagram. The 11 most common protein-altering variants in the BIC are marked as stars at their respective locations at the top. Black stars correspond to disease-causing mutations, white stars are variants with no known clinical significance, and grey stars are positions with unknown significance.

**Figure 5:**
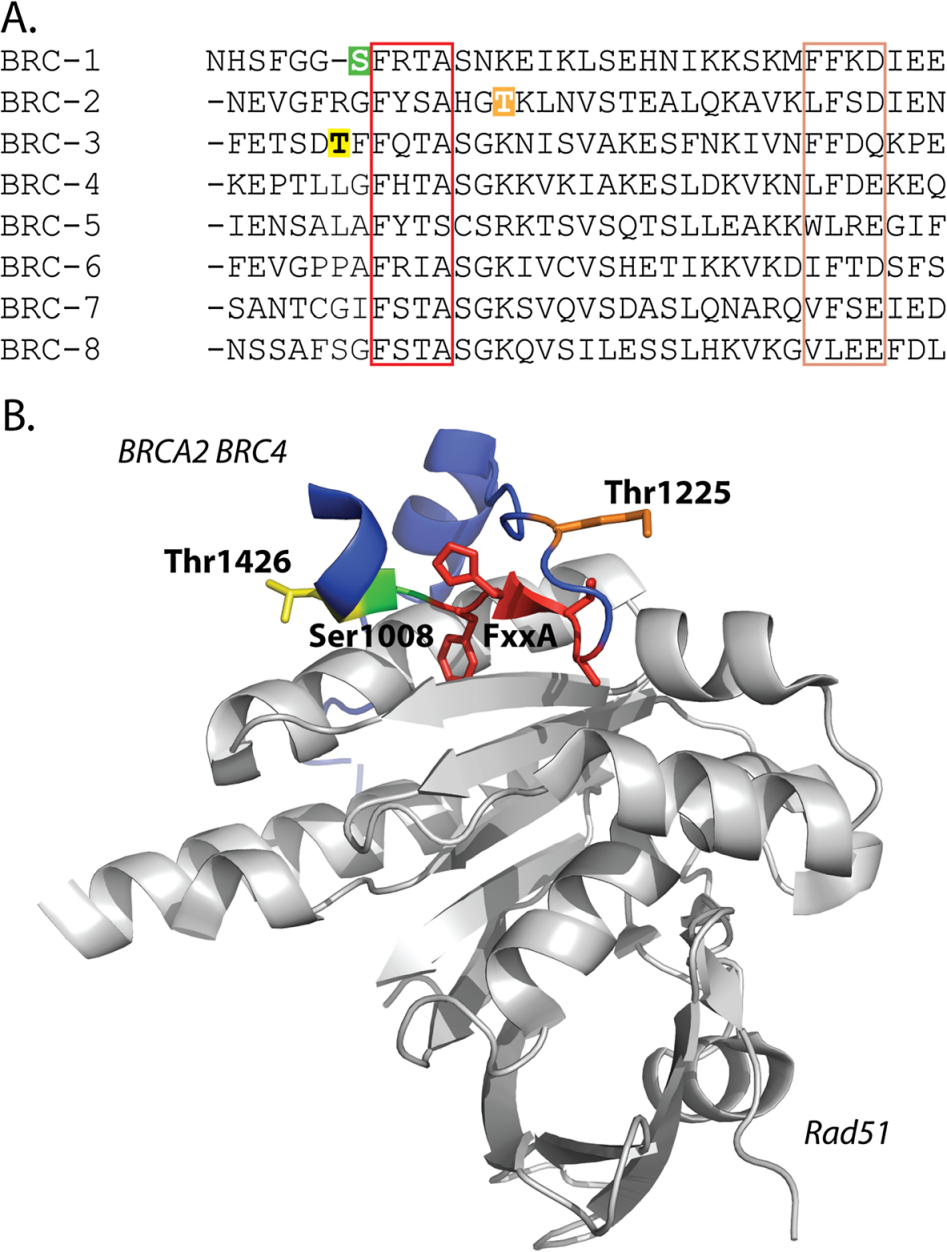
**The sites of positive selection lying within the BRC repeats of BRCA2 are located adjacent to the Rad51 binding region. A**. The 8 BRC repeats of the human BRCA2 protein were aligned using ClustalX. The red and peach colored boxes are the motifs within the BRC repeats thought to facilitate binding with Rad51 [20]. Residues 1008, 1225, and 1426 are colored in green, orange, and yellow, respectively. All three sites lie just adjacent to the FxxA motif which interacts with two hydrophobic pockets in the Rad51 oligomer. **B**. The co-crystal structure of BRC4 (blue) in complex with Rad51 (grey) is shown (PDB ID 1N0W [19]). The FxxA motif is depicted in red. Residues 1008, 1225, and 1426 are shown in green, orange, and yellow, respectively.

In contrast to BRCA1, BRCA2 is a 390 kDa nuclear protein that is exclusively involved in the homologous recombination pathway for repairing double-strand breaks. The eight BRC motifs and the extreme C terminus mediate interactions with and recruitment of Rad51, a protein that catalyzes strand invasion during homologous recombination [16-18]. All eight BRC repeats are encoded within exon 11. The M8 model estimates that five codons are evolving under positive selection with posterior probability > 0.85 (Figure 4A). Two of these positively selected sites were found to have a human polymorphism documented in the BIC (Figure 4A). When all five sites of positive selection are mapped onto a domain diagram of BRCA2 (Figure 4B), they cluster within the first three BRC domains (1008, 1225, and 1426) and the intervening regions (1159 and 1272). To examine this further, we aligned the amino acid sequence of all eight BRC repeats of human BRCA2 and highlighted sites 1008, 1225, and 1426 (Figure 5A). Surprisingly, all three sites of positive selection lie adjacent to a hydrophobic motif (FxxA) known to mediate interactions with Rad51 (Figure 5A red box). Since the co-crystal structure of the BRCA2 BRC4 in complex with Rad51 is available, we mapped these three sites to their analogous positions in BRC4 and found that they are in close proximity to the Rad51 binding interface (Figure 5B, PDB: 1N0W) [19]. The clustering of these residues near this interface might provide a clue to the driver of natural selection at these sites.

## Discussion

Nearly all known cases of recurrent positive selection in primate genomes involve genes in one of three categories: 1) immunity, 2) environmental perception (such as odorant and taste receptors), or 3) sexual selection and mate-choice [21,22]. This is due to the fact that ever-changing external stimuli (i.e. pathogens, environmental odors/tastes, etc.) drive the selection of new allelic variants. For example, immunity factors that are constantly challenged by pathogens exhibit some of the most striking signatures of positive selection seen in primate genomes [23-28]. Here, immunity genes will experience positive selection for protein-altering mutations that improve recognition of a relevant pathogen. Conversely, the pathogen will counter-evolve to escape detection, again placing selective pressure on the host population for new mutations that improve the immunity protein. This cycle can repeat itself indefinitely, resulting in an ever-escalating host-virus arms race. Therefore, it is surprising to see that *BRCA1* and *BRCA2*, genes that do not classically fit into any of the three categories listed above, are evolving in a similar manner to these highly adaptive immunity genes. In addition to the two described here, other DNA repair genes have also been shown to evolve under positive selection [29,30], but the driver behind this unusual finding remains to be identified.

An intense battle exists between host DNA repair machinery and viruses, and we propose that this could contribute to the evolutionary signatures documented here. Many viruses are known to interact with the DNA repair machinery and cell cycle regulators [31,32]. One fundamental issue is that the free ends of viral genomes are exposed, in contrast to the host’s DNA, which is capped by telomeres. Despite this, many viruses need to access the nucleus where the host’s DNA repair machinery recognizes these un-capped viral genome ends as “damaged” cellular DNA, activating the DNA damage response. In order for productive infection to proceed, viruses must actively thwart these host repair pathways. For example, DNA repair proteins interfere with the adenovirus lifecycle by concatenating the ends of newly synthesized viral DNA, inhibiting efficient packaging into viral progeny [33]. In turn, adenovirus has evolved a way around this blockade by encoding proteins that mislocalize or degrade the specific host factors involved. Depending on the virus involved, host DNA repair factors can also be hijacked to facilitate viral replication. For instance, herpes simplex virus-1 simultaneously activates DNA repair constituents that aid in viral genome replication [34,35] and counteracts those that do not [36,37]. Human immunodeficiency virus 1 is also known to activate the DNA damage response and manipulate cell cycle checkpoints through the actions of its accessory protein Vpr [38,39]. Additionally, several studies have shown that specific DNA repair proteins play critical roles in retroviral genome integration [40-43] while others seem to decrease the efficiency of infection [44-46].

One can imagine that these and other viruses that access the nucleus during replication could feasibly interact with BRCA1 or BRCA2, driving the selection of variants that ultimately lead to decreased susceptibility to infection. However, it is possible that variant alleles selected for this purpose would have detrimental consequences to protein function in the context of host DNA repair. Most of the deleterious *BRCA1* and *BRCA2* variants characterized thus far introduce stop codons or frame-shifts that result in premature truncation of the protein, the consequences of which manifest as cancer at relatively early ages. The effects of non-synonymous point mutations, such as those documented here, might be expected to be much more subtle. The effects of subtle mutations are more difficult to assess because the resulting genomic instability may only be realized later in life and can be confounded by other genetic or environmental influences. We therefore propose a hypothesis where viruses are driving the intriguingly rapid rate of evolution seen in *BRCA1* and *BRCA2*, potentially giving rise to antagonistic pleiotropy. This would be analogous to the malaria and sickle cell anemia trade-off that is well documented [47].

## Conclusions

The BRCA1 and BRCA2 proteins play key roles in the repair of damage to chromosomal DNA. We have expanded the analysis of the evolution of these genes, showing that both have been subject to recurrent positive selection during simian primate speciation. Although the force or forces driving the diversifying selection of these genes is unknown, the result is that the sequence of these proteins has been altered in humans and our closest living relatives. It remains to be seen whether this is an instance of antagonistic pleiotropy, where positive selection driven by one force causes functional consequences in another context, potentially the formation of cancers [48].

## Methods

### Non-human primate samples

Of the 44 chimpanzee samples evaluated in this study, 34 were obtained from the Chimpanzee Biomedical Research Resource (NIH8U42OD011197-13), which is supported through a cooperative agreement with the National Institutes of Health (NIH). This NIH-supported colony is housed at the MD Anderson Cancer Center’s Michale E. Keeling Center for Comparative Medicine and Research (KCCMR) in Bastrop, TX. The origins of the chimpanzees comprising the KCCMR colony are highly diverse with only a few closely related (siblings/offspring) animals in the colony (Additional file 4). Blood from 34 chimpanzees was collected directly into PAXgene Blood RNA Tubes (PreAnalytix) at the same time other blood samples were obtained as part of the prescheduled annual veterinary exam for each animal. Another 10 chimpanzee genomic DNA samples were purchased from Coriell (Additional file 5).

All 44 rhesus macaque samples evaluated in this study were obtained from animals housed at the KCCMR in collaboration with researchers at this institution. The colony at the KCCMR is a closed breeding colony comprised of approximately 980 rhesus macaques of Indian-origin that originated from a colony of 286 founder animals in 1988 (degree of relatedness can be found in Additional file 6). Blood from these animals was collected directly into PAXgene Blood RNA Tubes (PreAnalytix) at the same time other blood samples were obtained as part of the prescheduled annual veterinary exam for each animal.

Bonobo genomic DNA samples were obtained from the integrated primate biomaterials and information resource (IPBIR) of the Coriell Institute or extracted from blood samples obtained from the Columbus zoo and the Language Research Center, Georgia State University. All seven individuals are unrelated (Additional file 7).

The remaining non-human primate samples were acquired as cell lines purchased from the Coriell Institute under a U.S. Fish and Wildlife Service permit (sources and unique identifiers are listed in Additional file 8). This study was approved by the University of Texas at Austin Institutional Review Board.

### Primate *BRCA1* and *BRCA2* sequencing

Human *BRCA1* and *BRCA2* coding sequences were obtained from GenBank (accession number NM 007294 and NM 000059, respectively). *BRCA1* and *BRCA2* sequences from chimpanzee, gorilla, orangutan, rhesus macaque, and marmoset were obtained using the BLAT alignment tool on the UCSC genome database (http://genome.ucsc.edu/). For the remaining 18 primate sequences, primary or immortalized cell lines were grown in standard media supplemented with 15% fetal bovine serum at 37°C and 5% CO_2_. Cells were collected and RNA was extracted using the AllPrep DNA/RNA kit (QIAGEN). cDNA libraries were generated using SuperScript III First-Strand Synthesis Kit (Invitrogen) using oligo dT or random hexamer primers. PCR products were generated using PCR SuperMix High Fidelity (Invitrogen) and directly sequenced or cloned into pCR4 for sequencing. Primers used for PCR and sequencing can be found in Additional files 9, 10, 11 and 12. These sequences have been deposited in GenBank (accession numbers KM017616-KM017652).

Blood from rhesus macaque and chimpanzee individuals was collected in PAXgene Blood RNA Tubes (PreAnalytiX). RNA was extracted using the PAXgene Blood miRNA Kit (QIAGEN) and genomic DNA was obtained using the AllPrep DNA/RNA kit (QIAGEN). *BRCA1* Exon 11 was amplified from extracted genomic DNA (chimpanzee, bonobo, and rhesus macaque) using PCR SuperMix High Fidelity (Invitrogen) and sequenced. Details on PCR and sequencing primers can be found in Additional file 9 and 10.

### PAML analysis

A multiple sequence alignment was generated for *BRCA1* and *BRCA2* using ClustalX2.1 [49]. The alignments are straight-forward with only a few small indels (Additional files 1 and 2). Gene sequences at each ancestral node were reconstructed using the codeml program in PAML 4.3 [50]. dN/dS values along each branch of the phylogenetic tree were calculated using the free-ratio model. Substitution counts given along specified branches are the estimates made in the free ratio model, but were also calculated by directly comparing the predicted ancestral and the known extant sequences and counting differences manually. Both methods yielded the same values. The one-ratio and two-ratio models were performed as described previously [51]. To detect selection, multiple alignments were fit to the NSsites models M1a (null model, codon values of dN/dS are fit into two site classes, one with value between 0 and 1, and one fixed at dN/dS = 1), M2a (positive selection model, similar to M1a but with an extra codon class of dN/dS > 1), M7 (null model, codon values of dN/dS fit to a beta distribution bounded between 0 and 1), M8a (null model, similar to M7 except with an extra fixed codon class at dN/dS = 1), and M8 (positive selection model, similar to M7 but with an extra class of dN/dS > 1). Model fitting was performed with multiple seed values for dN/dS (ω) and assuming either the f61 or f3x4 model of codon frequencies [52]. Likelihood ratio tests were performed to assess whether permitting some codons to evolve under positive selection gives a significantly better fit to the data than models where positive selection is not allowed [53,54]. These different model comparisons represent different trade-offs between power and accuracy [55]. In all cases the positive selection model was a significantly better fit (p < 0.05), and individual codons assigned to the dN/dS > 1 class with high posterior probabilities (P > 0.85 by Bayes Emperical Bayes [56]) were analyzed. The crystal structure was obtained from the RCSB Protein Data Bank (http://www.pdb.org) and residues under positive selection were mapped using MacPyMol (http://www.pymol.org).

### Hardy-weinberg equilibrium test

Single nucleotide polymorphisms (SNPs) were annotated for each bonobo, chimpanzee, and rhesus macaque individual. Allele frequencies were calculated for each SNP and tested for departure from Hardy-Weinberg equilibrium (http://www.oege.org) [57]. Chi squared values were calculated using 1 degree of freedom. A p-value (after Bonferroni correction) < 0.0056, 0.0056, and 0.0042 for bonobos, chimpanzees, and rhesus macaque, respectively, was considered statistically significant.

### Ethics

No new human data was generated or analyzed in this study.

## Abbreviations

BIC: Breast Cancer Information Core; SNP: Single Nucleotide Polymorphism.

## Competing interests

The authors declare that they have no competing interests.

## Authors’ contributions

DIL sequenced genes, carried out molecular genetic studies, analyzed data, and wrote the manuscript; RMM sequenced genes and analyzed data; UQL sequenced genes; ACS provided primate materials and performed statistical tests; GKW oversaw the collection of primate materials and edited the manuscript; AMD sequenced genes and edited the manuscript; SLS conceived the study, participated in its design and coordination, and edited the manuscript. All authors read and approved the final manuscript.

## Supplementary Material

Additional file 1 **Alignment of *BRCA1* sequences.** description – alignment of *BRCA1* sequences used in the PAML analyses. Click here for file

Additional file 2 **Alignment of *BRCA2* sequences.** description – alignment of *BRCA2* sequences used in the PAML analyses. Click here for file

Additional file 3 **Evolution of *BRCA2* over the course of primate speciation.** dN/dS values for each branch of the primate phylogeny were calculated using the free-ratio model in PAML [13]. Branches exhibiting dN/dS values > 1 are shown in bold italics. Dashes (-) represent branches where zero synonymous substitutions are predicted to have occurred. On these branches, dS = 0 and dN/dS can therefore not be calculated. In these instances, the numbers of non-synonymous (N) and synonymous (S) substitutions predicted to have occurred along each branch are indicated in parentheses (N:S). Of these, branches that experienced 4 or more non-synonymous changes are italicized. Click here for file

Additional file 4 **Degree of relatedness in *Pan troglodyte* (chimpanzee) individuals.** description – sex, age, and relatedness of chimpanzee individuals used in this study. Click here for file

Additional file 5 **Sources and unique identifiers of *Pan troglodyte* genomic DNA used to generate *BRCA1* exon 11 sequences.** description – sources and unique identifiers of chimpanzee genomic DNA used in this study. Click here for file

Additional file 6 **Degree of relatedness in *Macaca mulatta *** **(rhesus macaque) individuals.** description – relatedness of rhesus macaque individuals used in this study. Click here for file

Additional file 7 ***Pan paniscus* (bonobo) individuals information.** description – sex and sources of bonobo samples used in this study. Click here for file

Additional file 8 **Sources and unique identifiers of cell lines used to generate primate cDNA libraries and sequences.** description – sources and unique identifiers of cell lines used in this study. Click here for file

Additional file 9 **Primers used for *BRCA1* amplification and sequencing.** description – Primers used to amplify and sequence *BRCA1*. Click here for file

Additional file 10 **Sequences of primers used for BRCA1 sequencing.** description – sequences of primers used to amplify and sequence *BRCA1*. Click here for file

Additional file 11 **Primers used for *BRCA2* amplification and sequencing.** description – primers used to amplify and sequence *BRCA2*. Click here for file

Additional file 12 **Sequences of primers used for *BRCA2* sequencing.** description – sequences of primers used to amplify and sequence *BRCA2*. Click here for file
