# Supplementary material for: Rapid evolution of BRCA1 and BRCA2 in humans and other primates
Source: BMC Evol Biol. 2014 Jul 11;14:155. doi: 10.1186/1471-2148-14-155 (PMC4106182; doi:10.1186/1471-2148-14-155)
Supplement: Additional file 1 — Alignment of BRCA1 sequences. description – alignment of BRCA1 sequences used in the PAML analyses. [file 1471-2148-14-155-S1.pdf]

BRCA1 CLUSTAL 2.1 multiple sequence alignment

```
Human                  ATGGATTTATCTGCTCTTCGCGTTGAAGAAGTACAAAATGTCATTAATGCTATGCAGAAA
Rhesus                 ATGGATTTATCTGCTGTTCGCGTTGAAGAAGTACAAAATGTCATTAATGCTATGCAGAAA
CEMacaque              ATGGATTTATCTGCTGTTCGCGTTGAAGAAGTACAAAATGTCATTAATGCTATGCAGAAA
BMangabey              ATGGATTTATCTGCTGTTCGCGTTGAAGAAGTACAAAATGTCATTAATGCTATGCAGAAA
OliveBaboon            ATGGATTTATCTGCTGTTCGCGTTGAAGAAGTACAAAATGTCATTAATGCTATGCAGAAA
Talapoin               ATGGATTTATCTGCTGTTCGCGTTGAAGAAGTACAAAATGTCATTAATGCTATGCAGAAA
WolfsGuenon            ATGGATTTATCTGCTGTTCGCGTTGAAGAAGTACAAAATGTCATTAATGCTATGCAGAAA
Colobus                ATGGATTTATCTGCTGTTCGCGTTGAAGAAGTACAAAATGTCATTAATGCTATGCAGAAA
Chimpanzee             ATGGATTTATCTGCTCTTCGCGTTGAAGAAGTACAAAATGTCATTAACGCTATGCAGAAA
Bonobo                 ATGGATTTATCTGCTCTTCGCGTTGAAGAAGTACAAAATGTCATTAATGCTATGCAGAAA
Gorilla                ATGGATTTATCTGCTCTTCGCGTTGAAGAAGTACAAAATGTCATTAATGCTATGCAGAAA
Orangutan              ATGGATTTATCTGCTGTTCGCGTTGAAGAAGTACAAAATGTCATTAATGCTATGCAGAAA
BorneoOrangutan        ATGGATTTATCTGCTGTTCGCGTTGAAGAAGTACAAAATGTCATTAATGCTATGCAGAAA
PileatedGibbon         ATGGATTTATCTGCTGTTCGCGTTGAAGAAGTACAAAATGTCATTAATGCTATGCAGAAA
AgileGibbon            ATGGATTTATCTGCTGTTCGCGTTGAAGAAGTACAAAATGTCATTCATGCTATGCAGAAA
WHGibbon               ATGGATTTATCTGCTGTTCGCGTTGAAGAAGTACAAAATGTCATTAATGCTATGCAGAAA
Siamang                ATGGATTTATCTGCTGTTCGCGTTGAAGAAGTACAAAATGTCATTAATGCTATGCAGAAA
RCGibbon               ATGGATTTATCTGCTGTTCGCGTTGAAGAAGTACAAAATGTCATTAATGCTATGCAGAAA
WCGibbon               ATGGATTTATCTGCTGTTCGCGTTGAAGAAGTACAAAATGTCATTAATGCTATGCAGAAA
Marmoset               ATGGATTTATCTGCTGTTCATGTTGAAGAAGTACAAAATGTCCTTAATGCTATGCAGAAA
SqMnky                 ATGGATTTATCTGCTGTTCGTGTTGAAGAAGTGCAAAATGTCCTTAATGCTATGCAGAAA
TitiMonkey             ATGGATTTATCTGCTGTTCGCGTTGAAGAAGTACAAAATGTCCTTAATGCTATGCAGAAA
HowlerMk               ATGGATTTATCTGCTGTTCGTGTTGAAGAAGTACAAAATGTCCTTAATGCTATGCAGAAA
                       *************** ***  *********** ********* ** * ************

Human                  ATCTTAGAGTGTCCCATCTGTCTGGAGTTGATCAAGGAACCTGTCTCCACAAAGTGTGAC
Rhesus                 ATCTTAGAGTGTCCAATCTGTCTGGAGTTGATCAAGGAACCTGTCTCCACAAAGTGTGAC
CEMacaque              ATCTTAGAGTGTCCAATCTGTCTGGAGTTGATCAAGGAACCTGTCTCCACAAAGTGTGAC
BMangabey              ATCTTAGAGTGTCCAATCTGTCTGGAGTTGATCAAGGAACCTGTCTCCACAAAGTGTGAC
OliveBaboon            ATCTTAGAGTGTCCAATCTGTCTGGAGTTGATCAAGGAACCTGTCTCCACAAAGTGTGAC
Talapoin               ATCTTAGAGTGTCCAATCTGTCTGGAGTTGATCAAGGAACCTGTCTCCACAAAGTGTGAC
WolfsGuenon            ATCTTAGAGTGTCCAATCTGTCTGGAGTTGATCAAGGAACCTGTCTCCACAAAGTGTGAC
Colobus                ATCTTAGAGTGTCCAATCTGTCTGGAGTTGATCAAGGAACCTGTCTCCACAAAGTGTGAC
Chimpanzee             ATCTTAGAGTGTCCCATCTGTCTGGAGTTGATCAAGGAACCTGTCTCCACAAAGTGTGAC
Bonobo                 ATCTTAGAGTGTCCCATCTGTCTGGAGTTGATCAAGGAACCTGTCTCCACAAAGTGTGAC
Gorilla                ATCTTAGAGTGTCCCATCTGTCTGGAGTTGATCAAGGAACCTGTCTCCACAAAGTGTGAC
Orangutan              ATCTTAGAGTGTCCCATCTGTCTGGAGTTGATCAAGGAACCTGTCTCCACAAAGTGTGAC
BorneoOrangutan        ATCTTAGAGTGTCCCATCTGTCTGGAGTTGATCAAGGAACCTGTCTCCACAAAGTGTGAC
PileatedGibbon         ATCTTAGAGTGTCCCATCTGTCTGGAGTTGATCAAGGAACCTGTCTCCACAAAGTGTGAC
AgileGibbon            ATCTTAGAGTGTCCCATCTGTCTGGAGTTGATCAAGGAACCTGTCTCCACAAAGTGTGAC
WHGibbon               ATCTTAGAGTGTCCCATCTGTCTGGAGTTGATCAAGGAACCTGTCTCCACAAAGTGTGAC
Siamang                ATCTTAGAGTGTCCCATCTGTCTGGAGTTGATCAAGGAACCTGTCTCCACAAAGTGTGAC
RCGibbon               ATCTTAGAGTGTCCCATCTGTCTGGAGTTGATCAAGGAACCTGTCTCCACAAAGTGTGAC
WCGibbon               ATCTTAGAGTGTCCCATCTGTCTGGAGTTGATCAAGGAACCTGTCTCCACAAAGTGTGAC
Marmoset               ATCTTAGAGTGTCCAATCTGTCTGGAGTTGATCAAGGAACCTGTCTCCACAAAGTGTGAC
SqMnky                 ATCTTAGAGTGTCCAATCTGTCTGGAATTGATCAAGGAACCTGTCTCCACAAAGTGTGAC
TitiMonkey             ACCTTAGAGTGTCCAATTTGTCTGGAGTTGATCAAGGAACCTGTCTCCCCAAAATGTGAC
HowlerMk               ATCTTAGAGTGTCCAATCTGTCTGGAGTTGATCAAGGAACCTGTCTCCACAAAGTGTGAC
                       * ************ ** ******** ********************* **** ******

Human                  CACATATTTTGCAAATTTTGCATGCTGAAACTTCTCAACCAGAAGAAAGGGCCTTCACAG
Rhesus                 CACATATTTTGCAGATTTTGCATGCTGAAACTTCTCAACCAGAAGAAAGGGCCTTCACAG
CEMacaque              CACATATTTTGCAGATTTTGCATGCTGAAACTTCTCAACCAGAAGAAAGGGCCTTCACAG
BMangabey              CACATATTTTGCAGATTTTGCATGCTGAAACTTCTCAACCAGAAGAAAGGGCCTTCACAG
OliveBaboon            CACATATTTTGCAGATTTTGCATGCTGAAACTTCTCAACCAGAAGAAAGGGCCTTCACAG
Talapoin               CACATATTTTGCAAATTTTGCATGCTGAAACTTCTCAACCAGAAGAAAGGGCCTTCACAG
WolfsGuenon            CACATATTTTGCAAATTTTGCATGCTGAAACTTCTCAACCAGAAGAAAGGGCCTTCACAG
Colobus                CACATATTTTGCAGATTTTGCATGCTGAAACTTCTCAACCAGAAGAAAGGGCCTTCACAG
Chimpanzee             CACATATTTTGCAAATTTTGCATGCTGAAACTTCTCAACCAGAAGAAAGGGCCTTCACAG
Bonobo                 CACATATTTTGCAAATTTTGCATGCTGAAACTTCTCAACCAGAAGAAAGGGCCTTCACAG
Gorilla                CACATATTTTGCAAATTTTGCATGCTGAAACTTCTCAACCAGAAGAAAGGGCCTTCACAA
Orangutan              CACATATTTTGCAAGTTTTGCATGCTGAAACTTCTCAACCAGAAGAAAGGGCCTTCACAG
BorneoOrangutan        CACATATTTTGCAAATTTTGCATGCTGAAACTTCTCAACCAGAAGAAAGGGCCTTCACAG
PileatedGibbon         CACATATTTTGCAAATTTTGCATGCTGAAACTTCTCAACCAGAAGAAAGGGCCTTCACAG
AgileGibbon            CACATATTTTGCAAATTTTGCATGCTGAAACTTCTCAACCAGAAGAAAGGGCCTTCACAG
WHGibbon               CACATATTTTGCAAATTTTGCATGCTGAAACTTCTCAACCAGAAGAAAGGGCCTTCACAG
Siamang                CACATATTTTGCAAATTTTGCATGCTGAAACTTCTCAACCAGAAGAAAGGGCCTTCACAG
RCGibbon               CACATATTTTGCAAATTTTGCATGCTGAAACTTCTCAACCAGAAGAAAGGGCCTTCACAG
WCGibbon               CACATATTTTGCAAATTTTGCATGCTGAAACTTCTCAACCAGAAGAAAGGGCCTTCACAG
Marmoset               CACATATTTTGCAAATTTTGCATGCTGAAACTTCTTAACCAGAAGAAAGGGCCTTCACAG
```

```
SqMnky              CACATATTTTGCAAATTTTGCATGCTGAAACTTCTTAACCAGAAGAAAGGGCCTTCACAG
TitiMonkey          CACATATTTTGCAAATTTTGCATGCTGAAACTACTTAACCAGAAGAAAGGGCCTTCACAG
HowlerMk            CACATATTTTGCAAATTTTGCATGCTGAAACTTCTTAACCAGAAGAAAGGGCCTTCACAG
                    *************  ***************** ** *********************** 

Human               TGTCCTTTATGTAAGAATGATATAACCAAAAGGAGCCTACAAGAAAGTACGAGATTTAGT
Rhesus              TGTCCTTTGTGTAAGAATGATATAACCAAAAGAAGCCTACAAGAAAGTACAAGATTTAGT
CEMacaque           TGTCCTTTGTGTAAGAATGATATAACCAAAAGAAGCCTACAAGAAAGTACAAGATTTAGT
BMangabey           TGTCCTTTGTGTAAGAATGATATAACCAAAAGAAGCCTACAAGAAAGTACAAGATTTAGT
OliveBaboon         TGTCCTTTGTGTAAGAATGATATAACCAAAAGAAGCCTACAAGAAAGTACAAGATTTAGT
Talapoin            TGTCCTTTGTGTAAGAATGATATAACCAAAAGAAGCCTACAAGAAAGTACAAGATTTAGT
WolfsGuenon         TGTCCTTTGTGTAAGAATGATATAACCAAAAGAAGCCTACAAGAAAGTACAAGATTTAGT
Colobus             TGTCCTTTGTGTAAGAATGATATAACCAAAAGAAGCCTACAAGAAAGTACAAGATTTAGT
Chimpanzee          TGTCCTTTATGTAAGAATGATATAACCAAAAGGAGCCTACAAGAAAGTACGAGATTTAGT
Bonobo              TGTCCTTTATGTAAGAATGATATAACCAAAAGGAGCCTACAAGAAAGTACGAGATTTAGT
Gorilla             TGTCCTTTATGTAAGAATGATATAACCAAAAGGAGCCTACAAGAAAGTACGAGATTTAGT
Orangutan           TGTCCTTTATGTAAGAATGATATAACCAAAAGGAGCCTACAAGAAAGTACGAGATTTAGT
BorneoOrangutan     TGTCCTTTATGTAAGAATGATATAACCAAAAGGAGCCTACAAGAAAGTACGAGATTTAGT
PileatedGibbon      TGTCCTTTATGTAAGAATGATATAACCAAAAGAAGCCTACAAGAAAGTACGAGATTTAGT
AgileGibbon         TGTCCTTTATGTAAGAATGATATAACCAAAAGAAGCCTACAAGAAAGTACGAGATTTAGT
WHGibbon            TGTCCTTTATGTAAGAATGATATAACCAAAAGAAGCCTACAAGAAAGTACGAGATTTAGT
Siamang             TGTCCTTTATGTAAGAATGATATAACCAAAAGAAGCCTACAAGAAAGTACGAGATTTAGT
RCGibbon            TGTCCTTTATGTAAGAATGATATAACCAAAAGAAGCCTACAAGAAAGTACGAGATTTAGT
WCGibbon            TGTCCTTTATGTAAGAATGATATAACCAAAAGAAGCCTACAAGAAAGTACGAGATTTAGT
Marmoset            TGTCCTTTGTGTAAGAATGATATAACCAAAAGAAGCCTACAGGAAAGTACAAGATTTAGT
SqMnky              TGTCCTTTGTGTAAGAATGATATAACCAAAAGAAGCCTACAGGAAAGTACAAGATTTAGT
TitiMonkey          TGTCCTTTGTGTAAGACTGGTATAACCAAAAGAAGCCTACAGGAAAGTACAAGATTTAGT
HowlerMk            TGTCCTTTGTGTAAGAATGATATAACCAAAAGAAGCCTACAGGAAAGTACAAGATTTAGT
                    ******** ******* ** ************ ******** ******** *********

Human               CAACTTGTTGAAGAGCTATTGAAAATCATTTGTGCTTTTCAGCTTGACACAGGTTTGGAG
Rhesus              CAACTTGTTGAAGAGCTATTGAAAATCATTCATGCTTTTCAGCTTGACACAGGTTTGCAG
CEMacaque           CAACTTGTTGAAGAGCTATTGAAAATCATTCATGCTTTTCAGCTTGACACAGGTTTGCAG
BMangabey           CAACTTGTTGAAGAGCTATTGAAAATCATTCATGCTTTTCAGCTTGACACAGGTTTGCAG
OliveBaboon         CAACTTGTTGAAGAGCTATTGAAAATCATTCATGCTTTTCAGCTTGACACAGGTTTGCAG
Talapoin            CAACTTGTTGAAGAGCTATTGAAAATCATTCATGCTTTTCAGCTTGACACAGGTTTGCAG
WolfsGuenon         CAACTTGTTGAAGAGCTATTGAAAATCATTCATGCTTTTCAGCTTGACACAGGTTTGCAG
Colobus             CAACTTGTTGAAGAGCTATTGAAAATCATTCATGCTTTTCAGCTTGACACAGGTTTGCAG
Chimpanzee          CAACTTGTTGAAGAGCTATTGAAAATCATTTGTGCTTTTCAGCTTGACACAGGTTTGGAG
Bonobo              CAACTTGTTGAAGAGCTATTGAAAATCATTTGTGCTTTTCAGCTTGACACAGGTTTGGAG
Gorilla             CAACTTGTTGAAGAGCTATTGAAAATCATTTGTGCTTTTCAGCTTGACACAGGTTTGGAG
Orangutan           CAACTTGTAGAAGAGCTATTGAAAATCATTTGTGCTTTTCAGCTTGACACAGGTTTGCAG
BorneoOrangutan     CAACTTGTAGAAGAGCTATTGAAAATCATTTGTGCTTTTCAGCTTGACACAGGTTTGCAG
PileatedGibbon      CAACTTGTTGAAGAGCTATTGAAAATCATTTGTGCTTTTCAGCTTGACACAGGTTTGCAG
AgileGibbon         CAACTTGTTGAAGAGCTATTGAAAATCATTTGTGCTTTTCAGCTTGACACAGGTTTGCAG
WHGibbon            CAACTTGTTGAAGAGCTATTGAAAATCATTTGTGCTTTTCAGCTTGACACAGGTTTGCAG
Siamang             CAACTTGTTGAAGAGCTATTGAAAATCATTTGTGCTTTTCAGCTTGACACAGGTTTGCAG
RCGibbon            CAACTTGTTGAAGAGCTATTGAAAATCATTTGTGCTTTTCAGCTTGACACAGGTTTGCAG
WCGibbon            CAACTTGTTGAAGAGCTATTGAAAATCATTTGTGCTTTTCAGCTTGACACAGGTTTGCAG
Marmoset            CAACTTGTTGAAGAACTATTGAAAATCATTCACGCTTTTGAGCTTGACACGGGTTTGCAG
SqMnky              CAACTTGTTGAAGAACTATTGAAAATCATTCACGCTTTTGAGCTTGACACAGGTTTGCAG
TitiMonkey          CAACTTGTTGAAGAACTATTGAAAATCATTCATGCTTTTGAGCTTGACACAGGTTTGCAG
HowlerMk            CAACTTGTTGAAGAACTATTGAAAATCATTCACGCTTTTGAGCTTGACACAGGTTTGCAG
                    ******** ***** ***************   ****** ********** ****** **

Human               TATGCAAACAGCTATAATTTTGCAAAAAAGGAAAATAACTCTCCTGAACATCTAAAAGAT
Rhesus              TTTGCAAACAGTTATAATTTTGCAAAAAAGGAAAATCACTCTCCTGAACATCTAAAAGAT
CEMacaque           TTTGCAAACAGTTATAATTTTGCAAAAAAGGAAAATCACTCTCCTGAACATCTAAAAGAT
BMangabey           TTTGCAAACAGTTATAATTTTGCAAAAAAGGAAAATCACTCTCCTGAACATCTAAAAGAT
OliveBaboon         TTTGCAAACAGTTATAATTTTGCAAAAAAGGAAAATCACTCTCCTGAACATCTAAAAGAT
Talapoin            TTTGCAAACAGTTATAATTTTGCAAAAAAAGAAAATCACTCTCCTGAACATCTAAAAGAT
WolfsGuenon         TTTGCAAACAGTTATAATTTTGCAAAAAAGGAAAATCACTCTCCTGAACATCTAAAAGAT
Colobus             TTTGCAAACAGTTATAATTTTGCAAAAAAGGAAAATAACTCTCCTGAACATCTAAAAGAT
Chimpanzee          TATGCAAACAGTTATAATTTTGCAAAAAAGGAAAATAACTCTCCTGAACATCTAAAAGAT
Bonobo              TATGCAAACAGTTATAATTTTGCAAAAAAGGAAAATAACTCTCCTGAACATCTAAAAGAT
Gorilla             TATGCAAACAGTTATAATTTTGCAAAAAAGGAAAATAACTCTCCTGAACATCTAAAAGAT
Orangutan           TATGCAAACAGTTATAATTTTGCAAAAAAGGAAAATAACTCTCCTGAACATCTAAAAGAT
BorneoOrangutan     TATGCAAACAGTTATAATTTTGCAAAAAAGGAAAATAACTCTCCTGAACATCTAAAAGAT
PileatedGibbon      TATGCAAACAGTTATAATTTTGCAAAAAAGGAAAATAACTCTCCTGAACATCTAAAAGAT
AgileGibbon         TATGCAAACAGTTATAATTTTGCAAAAAAGGAAAATAACTCTCCTGAACATCTAAAAGAT
WHGibbon            TATGCAAACAGTTATAATTTTGCAAAAAAGGAAAATAACTCTCCTGAACATCTAAAAGAT
Siamang             TATGCAAACAGTTATAATTTTGCAAAAAAGGAAAATAACTCTCCTGAACATCTAAAAGAT
RCGibbon            TATGCAAACAGTTATAATTTTGCAAAAAAGGAAAATAACTCTCCTGAACATCTAAAAGAT
```

```
WCGibbon            TATGCAAACAGTTATAATTTTGCAAAAAAGGAAAATAACTCTCCTGAACATCTAAAAGAT
Marmoset            TTTGCAAACAGTTATAATTTTGCCAAAAAGGAAAATAACTCTCCTGAACATCTAAAAGAT
SqMnky              TTTGCAAACAGTTATAATTTTGCAAAAAAGGAAAATAACTCTCCTGAACATCTAAAAGAT
TitiMonkey          TTTGCAAACAGTTATAATTTTGCAAAAAAGGAAAATAACTCTCCTCAACATCTAAAAGAT
HowlerMk            TTTGCAAACAGTCATAATTTTGCAAAAAAGGAAAATAACTCTCCTGAACATCTAAAAGAT
                    * *********  ********** ***** ****** ******** **************

Human               GAAGTTTCTATCATCCAAAGTATGGGCTACAGAAACCGTGCCAAAAGACTTCTACAGAGT
Rhesus              GAAGTTTCTATCATCCAAAGTATGGGCTACAGAAACCGTGCCAAAAGACTTCTACAGAGT
CEMacaque           GAAGTTTCTATCATCCAAAGTATGGGCTACAGAAACCGTGCCAAAAGACTTCTACAGAGT
BMangabey           GAAGTTTCTATCATCCAAAGTATGGGCTACAGAAACCGTGCCAAAAGACTTCTACAGAGT
OliveBaboon         GAAGTTTCTATCATCCAAAGTATGGGCTACAGAAACCGTGCCAAAAGACTTCTACAGAGT
Talapoin            GAAGTTTCTATCATCCAAAGTATGGGCTACAGAGACCGTGCCAAAAGACTTCTACAGAGT
WolfsGuenon         GAAGTTTCTGTCATCCAAAGTATGGGCTACAGAGACCGTGCCAAAAGACTTCTACAGAGT
Colobus             GAAGTTTCTCTCATCCAAAGTATGGGCTACAGAAACCGTGCCAGAAGACTTCTACAGAGT
Chimpanzee          GAAGTTTCTATCATCCAAAGTATGGGCTACAGAAACCGTGCCAAAAGACTTCTACAGAGT
Bonobo              GAAGTTTCTATCATCCAAAGTATGGGCTACAGAAACCGTGCCAAAAGACTTCTACAGAGT
Gorilla             GAAGTTTCTATCATCCAAAGTACGGGCTACAGAAGCCGTGCCAAAAGACTTCTACAGAGT
Orangutan           GAAGTTTCTATCATCCAAAGTATGGGCTACAGAAATCGTGCCAAAAGACTTCTACAGAGT
BorneoOrangutan     GAAGTTTCTATCATCCAAAGTATGGGCTACAGAAATCGTGCCAAAAGACTTCTACAGAGT
PileatedGibbon      GAAGTTTCTATCATCCAAAGTATGGGCTACAGAAACCGTGCCAAAAGACTTCTACAGAGT
AgileGibbon         GAAGTTTCTATCATCCAAAGTATGGGCTACAGAAACCGTGCCAAAAGACTTCTACAGAGT
WHGibbon            GAAGTTTCTATCATCCAAAGTATGGGCTACAGAAACCGTGCCAAAAGACTTCTACAGAGT
Siamang             GAAGTTTCTATCATCCAAAGTATGGGCTACAGAAACCGTGCCAAAAGACTTCTACAGAGT
RCGibbon            GAAGTTTCTATCATCCAAAGTATGGGCTACAGAAACCGTGCCAAAAGACTTCTACAGAGT
WCGibbon            GAAGTTTCTATCATCCAAAGTATGGGCTACAGAAACCGTGCCAAAAGACTTCTACAGAGT
Marmoset            GAATTTTCTATCATCCAAAGTATGGGCTACAGAAATCGTGCCAAAAGACTTCGACAGAGT
SqMnky              GAATTTTCTATCATCCAAAGTATGGGCTACAGAAATCGTGCCAAAAGACTTCGACAAAGT
TitiMonkey          GAATTTTCTATCATCCAAAGTATGGGATACAGAAATCGTGCCAAAAGACTTCGACAGAGT
HowlerMk            GAATTTTCTATCATCCAAAGTATGGGCTACAGAAATCGTGCCAAAAGACTTCGACAGAGT
                    *** ***** ************ *** ******   ******* ******** *** ***

Human               GAACCCGAAAATCCTTCCTTGCAGGAAACCAGTCTCAGTGTCCAACTCTCTAACCTTGGA
Rhesus              GAACCCGAAAATCCTTCCTTGCAGGAAACCAGTCTCAGTGTCCCACTCTCTAACCTTGGA
CEMacaque           GAACCCGAAAATCCTTCCTTGCAGGAAACCAGTCTCAGTGTCCCACTCTCTAACCTTGGA
BMangabey           GAACCCGAAAATCCTTCCTTGCAGGAAACCAGTCTCAGTGTCCCACTCTCTAACCTTGGA
OliveBaboon         GAACCCGAAAATCCTTCCTTGCAGGAAACCAGTCTCAGTGTCCCACTCTCTAACCTTGGA
Talapoin            GAACCCGAAAATCCTTCCTTGCAGGAAACCAGTCTCAGTGTCCCACTCTCTAACCTTGGA
WolfsGuenon         GAACCCGAAAATCCTTCCTTGCAGGAAACCAGTCTCAGTGTCCCATTCTCTAACCTTGGA
Colobus             GAACCCGAAAATCCTTCCTTGCAGGAAACCAGTCTCAGTGTCCCACTCTCTAACTTTGGA
Chimpanzee          GAACCTGAAAATCCTTCCTTGCAGGAAACCAGTCTCAGTGTCCAACTCTCTAACCTTGGA
Bonobo              GAACCTGAAAATCCTTCCTTGCAGGAAACCAGTCTCAGTGTCCAACTCTCTAACCTTGGA
Gorilla             GAACCCGAAAATCCTTCCTTGCAGGAAACCGGTCTCAGTGTCCAAGTCTCTAACCTTGGA
Orangutan           GAACCCGAAAATCCTTCCTTGCAGGAAACCAGTCCCAGTGTCCAACTCTCTAACCTTGGA
BorneoOrangutan     GAACCCGAAAATCCTTCCTTGCAGGAAACCAGTCCCAGTGTCCAACTCTCTAACCTTGGA
PileatedGibbon      GAA------AATCCTTCCTTGCAGGAAACCAGTTTCAGTGTCCAACTCTCTAACCTCGGA
AgileGibbon         GAA------AATCCTTCCTTGCAGGAAACCAGTTTCAGTGTCCAACTCTCTAACCTCGGA
WHGibbon            GAA------AATCCTTCCTTGCAGGAAACCAGTTTCAGTGTCCAACTCTCTAACCTCGGA
Siamang             GAA------AATCCTTCCTTGCAGGAAACCAGTTTCAGTGTCCAACTCTCTAACCTCGGA
RCGibbon            GAACCTGAAAATCCTTCCTTGCAGGAAACCAGTTTCAGTGTCCAACTCTCTAACCTTGGA
WCGibbon            GAACCTGAAAATCCTTCCTTGCAGGAAACCAGTTTCAGTGTCCAACTCTCTAACCTTGGA
Marmoset            GAACCCCAAAATCCTACCTTGCAGGAAACCAATCTCAGTGTCCAGCTCTCTAACCCTGGA
SqMnky              GAACCCCAAAATCCTACCTTGCAGGAAACCAGTCTCAGTGTCCAGCTCTCTAACCCTGGA
TitiMonkey          GAACCCGAAAATCCTACCTTGCAGGAAACCAGTCTCAGTGTCCAGCTCGCTAACCCTGGA
HowlerMk            GAACCCGAAAATCCTTCTTTGCAGGAAACGAGTCTCAGTGTCCAACTCTCTAACCCTGGA
                    ***      ****** * ***********   *  ********   ** *****   ***

Human               ACTGTGAGAACTCTGAGGACAAAGCAGCGGATACAACCTCAAAAGACGTCTGTCTACATT
Rhesus              ATTGTGAGAACTCTGAGGACAAAGCAGCAGATACAACCTCAAAAGAAGTCTGTCTACATT
CEMacaque           ATTGTGAGAACTCTGAGGACAAAGCAGCAGATACAACCTCAAAAGAAGTCTGTCTACATT
BMangabey           ATTGTGAGAACTCTGAGGACAAAGCAGCGGATACAACCTCAAAAGAAGTCTGTCTACATT
OliveBaboon         ATTGTGAGAACTCTGAGGACAAAGCAGCGGATACAACCTCAAAAGAAGTCTGTCTACATT
Talapoin            ATTGTGAGAACTCTGAGGACAAAGCAGCGGATACAACCTCAAAAGAAGTCTGTCTACATT
WolfsGuenon         ATTGTGAGAACTCTGAGGACAAAGCAGCGGATACAACCTCAAAAGAAGTCTGTCTACATT
Colobus             ATTGTGAGAACTCTGAGGACAAAGCAGCGGATACAACCTCAAAAGAAGTCTGTCTACATT
Chimpanzee          ACTGTGAGAACTCTGAGGACAAAGCAACGGATACAACCTCAAAAGAAGTCTGTCTACATT
Bonobo              ACTGTGAGAACTCTGAGGACAAAGCAGCGGATACAACCTCAAAAGAAGTCTGTCTACATT
Gorilla             ACTGTGAGAACTCTGAGGACAAAGCAGCGGATACAACCTCAAAAGAAGTCTGTCTACATT
Orangutan           ACTGTGAGAACTCTGAGGACAAAGCAGCGGATACAACCTCAAAAGAAGTCTGTTTACATT
BorneoOrangutan     ACTGTGAGAACTCTGAGGACAAAGCAGCGGATACAACCTCAAAAGAAGTCTGTTTACATT
PileatedGibbon      ACTGTGAGAACTCTGAGGACAAAGCAGCGGATACAACCTCAAAAGAAGTCTGTCTACATT
AgileGibbon         ACTGTGAGAACTCTGAGGACAAAGCAGCGGATACAACCTCAAAAGAAGTCTGTCTACATT
WHGibbon            ACTGTGAGAACTCTGAGGACAAAGCAGCGGATACAACCTCAAAAGAAGTCTGTCTACATT
```

```
Siamang              ACTGTGAGAACTCTGAGGACAAAGCAGCGGATACAACCTCAAAAGAAGTCTGTCTACATT
RCGibbon             ACTGTGAGAACTCTGAGGACAAAGCAGCAGATACAACCTCAAAAGAAGTCTGTCTACATT
WCGibbon             ACTGTGAGAACTCTGAGGACAAAGCAGCAGATACAACCTCAAAAGAAGTCTGTCTACATT
Marmoset             ATTGTGAGATCTCTGAGGACAAAGCAGTGGATACAACCTCAAAATAAGTCTGTCTACATT
SqMnky               ATTGTGAGATCGCTGAGGACAAAGCAGTGGATACAGCCTCAAAATACGTCTGTCTACATT
TitiMonkey           ATTGTGAGATCTCTGAGGACAAAGCAGCGGATACAACTTCAAAATAAATCTGTCTACATT
HowlerMk             ATTGTGAGATCTCTGAGGACAAAGCAACGGATACAACCTCAAAATAAGTCTGTCTACATT
                     * ******* * **************   ****** * ****** *  ***** ******

Human                GAATTGGGATCTGATTCTTCTGAAGATACCGTTAATAAGGCAACTTATTGCAGTGTGGGA
Rhesus               GAACTGGGATCTGATTCTTCTGAAGATACGGTTAATAAGGCAACTTATTGCAGTGTGGGA
CEMacaque            GAACTGGGATCTGATTCTTCTGAAGATACGGTTAATAAGGCAACTTATTGCAGTGTGGGA
BMangabey            GAACTGGGATCTGATTCTTCTGAAGATACGGTTAATAAGGCAACTTATTGCAGTGTGGGA
OliveBaboon          GAACTGGGATCTGATTCTTCTGAAGATACGGTTAATAAGGCAACTTATTGCAGTGTGGGA
Talapoin             GAACTGGGATCTGATTCTTCTGAAGATACGGTTAATAAGGCAACTTATTGCAGTGTGGGA
WolfsGuenon          GAACTGGGATCTGATTCTTCTGAAGATACGGTTAATAAGGCAACTTATTGCAGTGTAGGA
Colobus              GAACTGGGATCTGATTCTTCTGAAGATACGGTTAATAAAGCAACTTATTGCAGTGTGGGA
Chimpanzee           GAATTGGGATCTGATTCTTCTGAAGATACCGTTAATAAGGCAACTTATTGCAGTGTGGGA
Bonobo               GAATTGGGATCTGATTCTTCTGAAGATACCGTTAATAAGGCAACTTATTGCAGTGTGGGA
Gorilla              GAATTGGGATCTGATTCTTCTGAAGATACCGTTAATAAGGCAACTTATTGCAGTGTGGGA
Orangutan            GAATTAGGATCTGATTCTTCTGAAGATACGGTTAATAAGGCAACTTATTGCAGTGTGGGA
BorneoOrangutan      GAATTGGGATCTGATTCTTCTGAAGATACGGTTAATAAGGCAACTTATTGCAGTGTGGGA
PileatedGibbon       GAATTGGGATCTGATTCTTCTGAAGATACGGTTAATAAGGCAACTTATTGCAGTGTGGGA
AgileGibbon          GAATTGGGATCTGATTCTTCTGAAGATACGGTTAATAAGGCAACTTATTGCAGTGTGGGA
WHGibbon             GAATTGGGATCTGATTCTTCTGAAGATACGGTTAATAAGGCAACTTATTGCAGTGTGGGA
Siamang              GAATTGGGATCTGATTCTTCTGAAGATACGGTTAATAAGGCAACTTATTGCAGTGTGGGA
RCGibbon             GAATTGGGATCTGATTCTTCTGAAGATACAGTTAATAAGGCAACTTATTGCAGTGTGGGA
WCGibbon             GAATTGGGATCTGATTCTTCTGAAGATACAGTTAATAAGGCAACTTATTGCAGTGTGGGA
Marmoset             GACTTGGGATCTGATTCTTCTGAAGATACAATTAATAAGAAAACTTACTGCAGTGTGGGA
SqMnky               GACTTGGGATCTGATTCTTCTGAAGATACAATTAATAAGAAAACTTACTGCAGTGTGGGA
TitiMonkey           GACTTGGGATCTGATTCTTCTGAAGATACAATTAATAAGAAAACTTACTGCAGTGTGGGA
HowlerMk             GACTTGGGATCTGATTCTTCTGAAGATACAGTTAATAAGAAAACTTACTGCAGCGTGGGA
                     **  * ***********************  *******   ****** ***** ** ***

Human                GATCAAGAATTGTTACAAATCACCCCTCAAGGAACCAGGGATGAAATCAGTTTGGATTCT
Rhesus               GATCAAGAATTGTTACAAATCACCCCTCAAGGAACCAGGGATGAAACCAGTTTGGATTCT
CEMacaque            GATCAAGAATTGTTACAAATCACCCCTCAAGGAACCAGGGATGAAACCAGTTTGGATTCT
BMangabey            GATCAAGAATTGTTACAAATCACCCCTCAAGGAACCAGGGATGAAACCAGTTTGGATTCT
OliveBaboon          GATCAAGAATTGTTACAAATCACCCCTCAAGGAACCAGGGATGAAACCAGTTTGGATTCT
Talapoin             GATCAAGAATTGTTACAAATCACCCCTCAAGGAACCAGGGATGAAACCAGTTTGGATTCT
WolfsGuenon          GATCAAGAATTGTTACAAATCACCCCTCAAGGAACCAGGGATGAAACCAGTTTGGATTCT
Colobus              GATCAAGAATTGTTACAAATCACCCCTCAAGGAACCAGGGATGAAACCAGTTTGGATTCT
Chimpanzee           GATCAAGAATTGTTACAAATCACCCCTCAAGGAACCAGGGATGAAATCAGTTTGGATTCT
Bonobo               GATCAAGAATTGTTACAAATCACCCCTCAAGGAACCAGGAATGAAATCAGTTTGGATTCT
Gorilla              GATCAAGAATTGTTACAAATCACCCCTCAAGGAACCAGGGATGAAATCAGTTTGGATTCT
Orangutan            GATCAAGAATTGCTACAAATCACCCCTCAAGGAACCAGCGATGAAATCAGTTTGGATTCT
BorneoOrangutan      GATCAAGAATTGCTACAAATCACCCCTCAAGGAACCAGCGATGAAATCAGTTTGGATTCT
PileatedGibbon       GATCAAGAATTGTTACAAATCACCCCTCAAGGAACCAGGGATGAAATCAGTTTGGATTCT
AgileGibbon          GATCAAGAATTGTTACAAATCACACCTCAAGGAACCAGGGATGAAATCAGTTTGGATTCT
WHGibbon             GATCAAGAATTGTTACAAATCACCCCTCAAGGAACCAGGGATGAAATCAGTTTGGATTCT
Siamang              GATCAAGAATTGTTACAAATCACCCCTCAAGGAACCAGGGATGAAATCAGTTTGGATTCT
RCGibbon             GATCAAGAATTGTTACAAATCACCCCTCAAGGAACCAGGGATGAAATCAGTTTGGATTCT
WCGibbon             GATCAAGAATTGTTACAAATCACCCCTCAAGGAACCAGGGATGAAATCAGTTTGGATTCT
Marmoset             GATCAAGAATTGTTACAAATCACCCCTCAAGGAACCAGGGATGAAACCAGTTTGGATTCT
SqMnky               GATCAAGAATTGTTTCAAATCACCCCTCAAGGAACCAGGGATGAAACCAGTTTGGATTCT
TitiMonkey           GATCAAGAATTGTTACAAATCACCCCTCAAGGAACCAGGGATGAAACCAGTTTGGATTCT
HowlerMk             GATCAAGAATTGTTACAAATCACCCCTCAAGGAACCAGGGATGAAACCAGTTTGAATTCT
                     ************ * ******** **************  ****** ******* *****

Human                GCAAAAAAGGCTGCTTGTGAATTTTCTGAGACGGATGTAACAAATACTGAACATCATCAA
Rhesus               GCAAAAAAGGCTGCTTGTGAATTTTCTGAGAAGGATATAACAAATACTGAACATCATCAG
CEMacaque            GCAAAAAAGGCTGCTTGTGAATTTTCTGAGAAGGATATAACAAATACTGAACATCATCAG
BMangabey            GCAAAAAAGGCTGCTTGTGAATTTTCTGAGAAGGATATAACAAATACTGAACATCATCAG
OliveBaboon          GCAAAAAAGGCTGCTTGTGAATTTTCTGAGAAGGATATAACAAATACTGAACATCATCAG
Talapoin             GCAAAAAAGGCTGCTTGTGAATTTTCTGAGAAGGATATAACAAATACTGAACATCATCAG
WolfsGuenon          GCAAAAAAGGCTGCTTGTGAATTTTCTGAGAAGGATATAACAAATACTGAACATCATCAG
Colobus              GCAAAAAAGGCTGCTTGTGAATTTTCTGAGAAGGATATAACAAATACTGAACATCATCAG
Chimpanzee           GCAAAAAAGGCTGCTTGTGAATTTTCTGAGACGGATGTAACAAATACTGAACATCATCAA
Bonobo               GCAAAAAAGGCTGCTTGTGAATTTTCTGAGACGGATGTAACAAATACTGAACATCATCAA
Gorilla              GCAAAAAAGGCTGCTTGTGAATTTTCTGAGACGGATGTAACGAATACTGAACATCATCAA
Orangutan            GCAAAAAAGGCTGCTTGTGAATTTTCTGAGACAGATGTAACAAATACTGAACATCATCAA
BorneoOrangutan      GCAAAAAAGGCTGCTTGTGAATTTTCTGAGACAGATGTAACAAATACTGAACATCATCAA
PileatedGibbon       GCAAAAAAGGCTGCTTGTGAATTTTCTGAGACGGATGTAACAAATACTGAACATCAACAA
```

```
AgileGibbon          GCAAAAAAGGCTGCTTGTGAATTTTCTGAGACGGATGTAACAAATACTGAACATCAACAA
WHGibbon             GCAAAAAAGGCTGCTTGTGAATTTTCTGAGACGGATATAACAAATACTGAACATCAACAA
Siamang              GCAAAAAAGGCTGCTTGTGAATTTTCTGAGACGGATGTAACAAATACTGAACATCAACAA
RCGibbon             GCAAAAAAGGCTGCTTGTGAATTTTCTGAGACGGATGTAACAAATACTGAACATCAACAA
WCGibbon             GCAAAAAAGGCTGCTTGTGAATTTTCTGAGACGGATGTAACAAATACTGAACATCAACAA
Marmoset             GCAAAAAAGGCTCCTTGTGAGTTTTCTGAGAAGGATATAACAAATACTGAACATCATCAA
SqMnky               GCAAAAAAGGCTCCTTGTGAGTTTTCTGAGAAGGATATAACAAATACTGAACATCATCAA
TitiMonkey           GCAAAAAAGGCTCCTTGTGAGTTTTCTGAGAAGGATATAACAAATACTGAACATCATCAA
HowlerMk             GCAAAAAAGGCTCCTTGTGAATTTTCTGAGAAGGATATAACAAATACTGAACATCATCAA
                     ************ ******* **********  *** **** ************** **

Human                CCCAGTAATAATGATTTGAACACCACTGAGAAGCGTGCAGCTGAGAGGCATCCAGAAAAG
Rhesus               TCCAGTAATAATGATTTGAACACCACTGAGAAACATGCAACTGAGAGGCATCCAGAAAAG
CEMacaque            TCCAGTAATAATGATTTGAACACCACTGAGAAACATGCAACTGAGAGGCATCCAGAAAAG
BMangabey            TCCAGTAATAATGATTTGAACACCACTGAGAAACATGCAACTGAGAGGCATCCAGAAAAG
OliveBaboon          TCCAGTAATAATGATTTGAACACCACTGAGAAACATGCAACTGAGAGGCATCCAGAAAAG
Talapoin             TCCAATAATAATGATTTGAACACCACTGAGAAACATGCAACTGAGAGGCATCCAGAAAAG
WolfsGuenon          TCCAGTAATAATGATTTGAACACCACTGAGAAACATGCAACTGAGAGGCATCCAGAAAAG
Colobus              TCCAGTAATAATGATTTGAATACCACTGAGAAGCATGCAACTGAGAGGCATCCAGAAAAG
Chimpanzee           CCCAGTAATAATGATTTGAACACCACTGAGAAGCGTGCAACTGAGAGGCATCCAGAAAAG
Bonobo               CCCAGTAATAATGATTTGAACACCACTGAGAAGCGTGCAACTGAGAGGCATCCAGAAAAG
Gorilla              CCCAGTAATAATGATTTGAACACCACTGAGAAGCGTGCAACTGAGAGGCATCCAGAAAAG
Orangutan            CCCAGTAATAACGATTTGAACACCACTGAGAAGCGTGCAACTGAGAGGCATCCAGAAAAG
BorneoOrangutan      CCCAGTAATAACGATTTGAACACCACTGAGAAGCGTGCAACTGAGAGGCATCCAGAAAAG
PileatedGibbon       CCCAGTAATAACGATTTGAACACCACTGAGAAGTGTGCAACTGAGAGGCATCCAGAAAAG
AgileGibbon          CCCAGTAATAACGATTTGAACACCACTGAGAAGTGTGCAACTGAGAGGCATCCAGAAAAG
WHGibbon             CCCAGTAATAACGATTTGAACACCACTGAGAAGTGTGCAACTGAGAGGCATCCAGAAAAG
Siamang              CCCAGTAATAACGATTTGAACACCACTGAGAAGCGTGCAACTGAGAGGCATCCAGAAAAG
RCGibbon             CCCAGTAATAACGATTTGAACACCACTGAGAAGCGTGCAACTGAGAGGCATCCAGAAAAG
WCGibbon             CCCAGTAATAACGATTTGAACACCACTGAGAAGCGTGCAACTGAGAGGCATCCAGAAAAG
Marmoset             TCCAGTAGTAACAATTTGAACACCACTGAGAAGCATGCAACTGAGAGGCATCCAGAAAAG
SqMnky               TCCAGTAATAACAATTTGAACACCACTGAGAAGCATGCAACTGAGAGGCATCCAGAAAAG
TitiMonkey           TCCAGTAATAACAATTTGAACACCACTGAGAAGCATGCAACTGAGAGGCATCCAGAAAAG
HowlerMk             TCCAGTAATAATGATTTGAACACCACTGAGAAGCATGCAACTGAGAGGCATCCAGAAAAG
                      *** ** ***  ******* ***********   **** ********************

Human                TATCAGGGTAGTTCTGTTTCAAACTTGCATGTGGAGCCATGTGGCACAAATACTCATGCC
Rhesus               TATCAGGGTAGTTCTGTTTCAAACTTGCATGTGGAGCCATGTGGCACAAATACTCATGCC
CEMacaque            TATCAGGGTAGTTCTGTTTCAAACTTGCATGTGGAGCCATGTGGCACAAATACTCATGCC
BMangabey            TATCAGGGTAGTTCTGTTTCAAACTTGCATGTGGAGCCATGTGGCACAAATACTCATGCC
OliveBaboon          TATCAGGGTAGTTCTGTTTCAAACTTGCATGTGGAGCCATGTGGCACAAATACTCATGCC
Talapoin             TATCAGGGTAGTTCTGTTTCAAACTTGCATGTGGAGCCATGTGGCACAAATACTCATGCC
WolfsGuenon          TATCAGGGTAGTTCTGTTTCAAACTTGCATGTGGAGCCATGTGGCACAAATACTCATGCC
Colobus              TATCAGGGTAGTTCTGTTTCAAACTTGCATGTGGAGCCATGTGGCACAAATACTCATGCC
Chimpanzee           TATCAGGGTAGTTCTGTTTCAAACTTGCATGTGGAGCCATGTGGCACAAATACTCATGCC
Bonobo               TATCAGGGTAGTTCTGTTTCAAACTTGCATGTGGAGCCATGTGGCACAAATACTCATGCC
Gorilla              TATCAGGGTAGTTCTGTTTCAAACTTGCATGTGGAGCCATGTGGCACAAATACTCATGCC
Orangutan            TATCAGGGTAGTTCTGTTTCAAACTTGCATGTGGAGCCATGTGGCACAAATACTCATGCC
BorneoOrangutan      TATCAGGGTAGTTCTGTTTCAAACTTGCATGTGGAGCCATGTGGCACAAATACTCATGCC
PileatedGibbon       TATCAGGGTAGTTCTGTTTCAAACTTGCATGTGGAGCCATGTGGCACAAATACTCATGCC
AgileGibbon          TATCAGGGTAGTTCTGTTTCAAACTTGCATGTGGAGCCATGTGGCACAAATACTCATGCC
WHGibbon             TATCAGGGTAGTTCTGTTTCAAGCTTGCATGTGGAGCCATGTGGCACAAATACTCATGCC
Siamang              TATCAGGGTAGTTCTGTTTCAAACTTGCATGTGGAGCCATGTGGCACAAATACTCATGCC
RCGibbon             TATCAGGGTAGTTCTGTTTCAAACTTGCATGTGGAGCTATGTGGCACAAATACTCATGCC
WCGibbon             TATCAGGGTAGTTCTGTTTCAAACTTGCATGTGGAGCTATGTGGCACAAATACTCATGCC
Marmoset             TATCAGAGTAGTTCTGTTTCAAACTTGCATGTGGAGCCATGTGGCACAAATACTCATGCC
SqMnky               TATCAGGGTAGTTCTGTTCCAAACTTGCATGTGGAGCCATGTGGCACAAATACTCATGCC
TitiMonkey           TATCAGGGGAGTTCTGTTTCAAACTTGCATGTGGAGCCATGTGGCACAAATACTCATGCC
HowlerMk             TGTCAGGGTAGTTCTGTTTCAAACTTGCATGTGGAGCCATGTGGCACAAATACTCATGCC
                     * **** * ********* *** ************** **********************

Human                AGCTCATTACAGCATGAGAACAGCAGTTTATTACTCACTAAAGACAGAATGAATGTAGAA
Rhesus               AGCTCATTACAGCATGAGAAC---AGTTTATTACTCACTAAAGACAGAATGAATGTAGAA
CEMacaque            AGCTCATTACAGCATGAGAAC---AGTTTATTACTCACTAAAGACAGAATGAATGTAGAA
BMangabey            AGCTCATTACAGCATGAGAAC---AGTTTATTACTCACTAAAGACAGAATGAATGTAGAA
OliveBaboon          AGCTCATTACAGCATGAGAAC---AGTTTATTACTCACTAAAGACAGAATGAATGTAGAA
Talapoin             AGCTCATTACAGCATGAGAAC---AGTTTATTACTCACTAAAGACAGAATGAATGTAGAA
WolfsGuenon          AGCTCATTACAGCATGAGAAC---AGTTTATTACTCACTAAAGACAGAATGAATGTAGAA
Colobus              AGCTCATTACAGCATGAGAACAGCAGTTTATTACTCACTAAAGACAGAATGAATGTAGAA
Chimpanzee           AGCTCATTACAGCATGAGAACAGCAGTTTATTACTCACTAAAGACAGAATGAATGTAGAA
Bonobo               AGCTCATTACAGCATGAGAACAGCAGTTTATTACTCACTAAAGACAGAATGAATGTAGAA
Gorilla              AGCTCATTACAGCATGAGAACAGCAGTTTATTACTCACTAAAGACAGAATGAATGTAGAA
Orangutan            AGCTCATTACAGCATGAGAACAGCAGTTTATTACTCACTAAAGACAGAATGAATGTAGAA
```

```
BorneoOrangutan         AGCTCATTACAGCATGAGAACAGCAGTTTATTACTCACTAAAGACAGAATGAATGTAGAA
PileatedGibbon          AGCTCATTACAGCATGAGAACAGCAGTTTATTACTCACTAAAGACAGAATGAATGTAGAA
AgileGibbon             AGCTCATTACAGCATGAGAACAGCAGTTTATTACTCACTAAAGACAGAATGAATGTAGAA
WHGibbon                AGCTCATTACAGCATGAGAACAGCAGTTTATTACTCACTAAAGACAGAATGAATGTAGAA
Siamang                 AGCTCATTACAGCATGAGAACAGCAGTTTATTACTCACTAAAGACAGAATGAATGTAGAA
RCGibbon                AGCTCATTACAGCATGAGAACAGCAGTTTATTACTCACTAAAGACAGAATGAATGTAGAA
WCGibbon                AGCTCATTACAGCATGAGAACAGCAGTTTATTACTCACTAAAGACAGAATGAATGTAGAA
Marmoset                AGCTCATTATGGCATGAGAACAGCAGTTTATTACTCACTAAAGACAGACTGAATGTAGAA
SqMnky                  AGCTCATTACAGCATGAGAACAGCAGTTTATTACTCACTAAAGACAGACTGAATGTAGAA
TitiMonkey              AGCTCATTACAGCATGAGAACAGCAGTTTATTACTCACTAAAGACAGACTGAATGTAGAA
HowlerMk                AGCTCATTACAGCATGAGAACAGCAGTTTGTTACTCACTAAAGACAGACTGAATGTAGAA
                        *********  **********   ***** ****************** ***********

Human                   AAGGCTGAATTCTGTAATAAAAGCAAACAGCCTGGCTTAGCAAGGAGCCAACATAACAGA
Rhesus                  AAGGCTGAATTCTGTAATAAAAGCAAACAGCCTGGCTTGGCAAGGAGCCAACATAACAGA
CEMacaque               AAGGCTGAATTCTGTAATAAAAGCAAACAGCCTGGCTTGGCAAGGAGCCAACATAACAGA
BMangabey               AAGGCTGAATTCTGTAATAAAAGCAAACAGCCTGGCTTGGCAAGGAGCCAACATAACAGA
OliveBaboon             AAGGCTGAATTCTGTAATAAAAGCAAACAGCCTGGCTTGGCAAGGAGCCAACATAACAGA
Talapoin                AAGGCTGAATTCTGTAATAAAAGCAAACAGCCTGGCTTGGCAAGGAGCCAACATAACAGA
WolfsGuenon             AAGGCTGAATTCTGTAATAAAAGCAAACAGCCTGGCTTGGCAAGGAGCCAACATAACAGA
Colobus                 AAGGCTGAATTCTGTAATAAAAGCAAACAGCCTGGCTTAGCAAGGAGCCAACATAACAGA
Chimpanzee              AAGGCTGAATTCTGTAATAAAAGCGAACAGCCTGGCTTAGCAAGGAGCCAACATAACAGA
Bonobo                  AAGGCTGAATTCTGTAATAAAAGCAAACAGCCTGGCTTAGCAAGGAGCCAACATAACAGA
Gorilla                 AAGGCTGAATTCTGTAATAAAAACAAACAGCCTGGCTTAGCAAGGAGCCAACATAACAGA
Orangutan               AAGGCTGAATTCTGTAATAAAAGCAAACAGCCTGGCTTAGCAAGGAGCCAACATAACAGA
BorneoOrangutan         AAGGCTGAATTCTGTAATAAAAGCAAACAGCCTGGCTTAGCAAGGAGCCAACATAACAGA
PileatedGibbon          AAGGCTGAATTCTGTAATAAAAGCAAACAGCCTGGCTTAGCAAGGAGCCAACATAACAGA
AgileGibbon             AAGGCTGAATTCTGTAATAAAAGCAAACAGCCTGGCTTAGCAAGGAGCCAACATAACAGA
WHGibbon                AAGGCTGAATTCTGTAATAAAAGCAAACAGCCTGGCTTAGCAAGGAGCCAACATAACAGA
Siamang                 AAGGCTGAATTCTGTAATAAAAGCAAACAGCCTGGCTTAGCAAGGAGCCAACATAACAGA
RCGibbon                AAGGCTGAATTCTGTAATAAAAGCAAACAGCCTGGCTTAGCAAGGAGCCAACATAACAGA
WCGibbon                AAGGCTGAATTCTGTAATAAAAGCAAACAGCCTGGCTTAGCAAGGAGCCAACATAACAGA
Marmoset                AAGGCTGAATTCTGTAATAAAAGCAAACAGCCTGGCTTAGCAAGGAGCCAACATAACAGA
SqMnky                  AAGGCTGAATTCTGTAATAAAAGCAAACAGCCTGGCTTAGCAAGGAGCCAACATAACAGA
TitiMonkey              AAGGCTGAATTCTGTAATAAAAGCAAACAGTCTGACTTAGCAAGGAGCCAACATAACAGA
HowlerMk                AAGGCTGAATTCTGTAATAAAAGCAAACAGCCTGGCTTAGCAAGGAGCCAACATAACAGA
                        ********************** * ***** *** *** *********************

Human                   TGGGCTGGAAGTAAGGAAACATGTAATGATAGGCGGACTCCCAGCACAGAAAAAAAGGTA
Rhesus                  TGGACTGGAAGTAAGGAAACATGTAATGATAGGCAGACTCCCAGCACAGAGAAAAAGGTA
CEMacaque               TGGACTGGAAGTAAGGAAACATGTAATGATAGGCAGACTCCCAGCACAGAGAAAAAGGTA
BMangabey               TGGACTGGAAGTAAGGAAACATGTAATGATAGGCAGACTCCCAGCACAGAGAAAAAGGTA
OliveBaboon             TGGACTGGAAGTAAGGAAACATGTAATGATAGGCAGACTCCCAGCACAGAGAAAAAGGTA
Talapoin                TGGATTGGAAGTAAGGAAACATGTAATGATAGGCAGACTCCCAGCACAGAGAAAAAGGTA
WolfsGuenon             TGGACTGGAAGTAAGGAAACATGTAATGATAGGCAGACTCCCAGCACAGAGAAAAAGGTA
Colobus                 TGGACTGGAAGTAAGGAAACATGTAATGATAGGCAGACTCCCAGCACAGAGAAAAAGGCA
Chimpanzee              TGGGCTGGAAGTAAGGAAACATGTAATGATAGGCGGACTCCCAGCACAGAAAAAAAGGTA
Bonobo                  TGGGCTGGAAGTAAGGAAACATGTAATGATAGGCGGACTCCCAGCACAGAAAAAAAGGTA
Gorilla                 TGGGCTGGAAGTAAGGAAACATGTAATGATAGGCGGACTCCCAGCACAGAAAAAAAGGTA
Orangutan               TGGGCTGGAAGTAAGGAAACATGTAATGATAGGCAGACTCCCAGCACAGAAAAAAAGGTA
BorneoOrangutan         TGGGCTGGAAGTAAGGAAACATGTAATGATAGGCAGACTCCCAGCACAGAAAAAAAGGTA
PileatedGibbon          TGGGCTGGAAGTAAGGAAACATGTAATGATAGGCAGACTCCCAGCACAGAGAAAAAGGTA
AgileGibbon             TGGGCTGGAAGTAAGGAAACATGTAATGATAGGCAGACTCCCAGCACAGAGAAAAAGGTA
WHGibbon                TGGGCTGGAAGTAAGGAAACATGTAATGATAGGCAGACTCCCAGCACAGAGAAAAAGGTA
Siamang                 TGGGCTGGAAGTAAGGAAACATGTAATGATAGGCAGACTCCCAGCACAGAGAAAAAGGTA
RCGibbon                TGGGCTGGAAGTAAGGAAACATGTAATGATAGGCAGACTCCCAGCACAGAGAAAAAGGTA
WCGibbon                TGGGCTGGAAGTAAGGAAACATGTAATGATAGGCAGACTCCCAGCACAGAGAAAAAGGTA
Marmoset                TGGGCTGAAAGTGAGGAAACGTGTAATGATAGGCAGACTCCCAGCACAGAGAAAAAGGTA
SqMnky                  TGGGCTGAAAGTGAGGAAACATGTAATGATAGGCAGACTCCCAGCACAGAGAAAAAGGTA
TitiMonkey              TGGGCTGAAAGTGAGGAAACATGTAATGATAGGCAGACTCCTAGCACAGAGAAAAAGGTA
HowlerMk                TGGGCTGAAAGTGAGGAAACATGTAATGATAGGCAGACTCCCAGCACAGAGAAAAAGGTA
                        ***  ** **** ******* ************* ****** ******** ******* *

Human                   GATCTGAATGCTGATCCCCTGTGTGAGAGAAAAGAATGGAATAAGCAGAAACTGCCATGC
Rhesus                  GATCTGAATGCTAATGCCCTGTATGAGAGAAAAGAATGGAATAAGCAAAAACTGCCATGC
CEMacaque               GATCTGAATGCTAATGCCCTGTATGAGAGAAAAGAATGGAATAAGCAAAAACTGCCATGC
BMangabey               GATCTGAATGCTAATGCCCTGTATGAGAGAAAAGAATGGAATAAGCAAAAACTGCCATGC
OliveBaboon             GATCTGAATGCTAATGCCCTGTATGAGAGAAAAGAATGGAATAAGCAAAAACTGCCATGC
Talapoin                GATCTGAATGCTGATGCCCTGTATGAGAGAAAAGAATGGAATAAGCAAAAACTGCCATGC
WolfsGuenon             GATCTGAATGCTGATGCCCTGTATGAGAGAAAAGAATGGAATAAGCAAAAACTGCCATGC
Colobus                 GATCTGAATGCTGATACCCTGTATGAGAGAAAAGAAAGGAATAAGCAGAAACTGCCATGC
Chimpanzee              GATCTGAATGCTGATCCCCTGTGTGAGAGAAAAGAATGGAATAAGCAGAAACTGCCATGC
Bonobo                  GATCTGAATGCTGATCCCCTGTGTGAGAGAAAAGAATGGAATAAGCAGAAACTGCCATGC
```

```
Gorilla                 GATCTGAATGCTGATCCCCTGTGTGAGAGAAACGAATGGAATAAGCAGAAACTGCCATGC
Orangutan               GACCTGAATGCTGATCCCCTGTGTGAGAGAAAAGAATGGAATAAGCAGAAACTGCCATGC
BorneoOrangutan         GACCTGAATGCTCATCCCCTGTGTGAGAGAAAAGAATGGAATAAGCAGAAACTGCCATGC
PileatedGibbon          GATCTGAATGCTGATCCCCTGTGTGAGAGAAAAGAATGGAATAAGCAGAAACTGCCATGC
AgileGibbon             GATCTGAATGCTGATCCCCTGTGTGAGAGAAAAGAATGGAATAAGCAGAAACTGCCATGC
WHGibbon                GATCTGAATGCTGATCCCCTGTGTGAGAGAAAAGAATGGAATAAGCAGAAACTGCCATGC
Siamang                 GATCTGAATGCTGATCCCCTGTGTGAGAGAAAAGAATGGAATAAGCAGAAACTGCCATGC
RCGibbon                GATCTGAATGCTGATCCCCTGTGTGAGAGAAAAGAATGGAATAAGCAGAAACTGCCATGC
WCGibbon                GATCTGAATGCTGATCCCCTGTGTGAGAGAAAAGAATGGAATAAGCAGAAACTGCCATGC
Marmoset                GATCTGGATGCTGATCCCCTGCATGGGAGAAAAGAATGGAATGAGCAGAAACCTCCATGC
SqMnky                  GATCTGGATGCTGATCCCCTGCATGGGAGAAAAGAATGGAATAAGCAGAAACCTCCATGC
TitiMonkey              GATCTGGATGCTGATCCCCTGTATGGGAGAAAAGAATGGAATAAGCAGAAACCTCCATGC
HowlerMk                GATCTGGATGCTGATCCCCTGCATGGGAGAAAAGAATGGAATAAGCAGAAACCTCCGTGC
                        ** *** ***** ** *****  ** ****** *** ***** **** ****  ** ***

Human                   TCAGAGAATCCTAGA---GATACTGAAGAT---GTTCCTTGGATAACACTAAATAGCAGC
Rhesus                  TCTGAGAATCCTAGA---GACGCTGAAGAT---GTTCCTTGGATAACACTAAATAGCAGC
CEMacaque               TCTGAGAATCCTAGA---GACGCTGAAGAT---GTTCCTTGGATAACACTAAATAGCAGC
BMangabey               TCTGAGAATCCTAGA---GACACTGAAGATGATGTTCCTTGGATAACACTAAATAGCAGC
OliveBaboon             TCTGAGAATCCTAGA---GACACTGAAGAT---GTTCCTTGGATAACACTAAATAGCAGC
Talapoin                TCTGAGAATCCTAGA---GACACTGAAGAT---GTTCCTTGGATAACACTAAATAGCAGC
WolfsGuenon             TCTGAGAATCCTAGA---GACACTGAAGAT---GTTCCTTGGATAACACTAAATAGCAGC
Colobus                 TCTGAGAATCCTAGA---GACACTGAAGAT---GTTCCTTGGATAACACTGAATAGCAGC
Chimpanzee              TCAGAGAATCCTAGA---GATACTGAAGAT---GTTCCTTGGATAACACTAAATAGCAGC
Bonobo                  TCAGAGAATCCTAGA---GATACTGAAGAT---GTTCCTTGGATAACACTAAATAGCAGC
Gorilla                 TCAGAGAATCCTAGA---GATACTGAAGAT---GTTCCTTGGATAACACTAAATAGCAGC
Orangutan               TCAGAGAATCCTAGA---GATACTGAAGAT---GTTCCTTGGATAACACTAAATAGCAGC
BorneoOrangutan         TCAGAGAATCCTAGA---GATACTGAAGAT---GTTCCTTGGATAACACTAAATAGCAGC
PileatedGibbon          TCAGAGAATCTTACA---GATACTGAAGAT---GTTCCTTGGATAACACTAAATAGCAGC
AgileGibbon             TCAGAGAATCCTACA---GATACTGAAGAT---GTTCCTTGGATAACACTAAATAGCAGC
WHGibbon                TCAGAGAATCCTACA---GATACTGAAGAT---GTTCCTTGGATAACACTAAATAGCAGC
Siamang                 TCAGAGAATCCTACA---GATACTGAAGAT---GTTCCTTGGATAACACTAAATAGCAGC
RCGibbon                TCAGAGAATCCTACA---GATACTGAAGAT---GTTCCTTGGATAACACTAAATAGCAGC
WCGibbon                TCAGAGAATCCTACA---GATACTGAAGAT---GTTCCTTGGATAACACTAAATAGCAGC
Marmoset                TCTGAGAAGCCTAGAGATGATACTGAAGAT---GTTGCTTGGTTAACACTAAATAGCAGC
SqMnky                  TCTGAGAATTCTAATAATGATACTGAAGAT---GTTGCTTGGATGACACTAAATAGCAGC
TitiMonkey              TCTGAGAATCCTAGAGATGATACTGAAGAT---ATTGCTTGGATAACACTAAATAGCAGC
HowlerMk                TCTGAGAATCCTAGAGATGATACTGAAGAT---GTTGCTTGGATAACGCTAAATAGCAGC
                        ** *****   **     **  ********    ** ***** * ** ** *********

Human                   ATTCAGAAAGTTAATGAGTGGTTTTCCAGAAGTGATGAACTGTTAGGTTCTGATGACTCA
Rhesus                  ATTCAGAAAGTTAATGAGTGGTTTTCCAGAAGTGATGAACTGTTAAGTTCTGATGACTCA
CEMacaque               ATTCAGAAAGTTAATGAGTGGTTTTCCAGAAGTGATGAACTGTTAAGTTCTGATGACTCA
BMangabey               ATTCAGAAAGTTAATGAGTGGTTTTCCAGAAGTGATGAACTGTTAAGTTCTGATGACTCA
OliveBaboon             ATTCAGAAAGTTAATGAGTGGTTTTCCAGAAGTGATGAACTGTTAAGTTCTGATGACTCA
Talapoin                ATTCAGAAAGTTAATGAGTGGTTTTCCAGAAGTGATGAACTGTTAAGTTCTGATGACTCA
WolfsGuenon             ATTCAGAAAGTTAATGAGTGGTTTTCCAGAAGTGATGAACTGTTAAGTTCTGATGACTCA
Colobus                 ATTCAGAAAGTTAATGAGTGGTTTTCCAGAAGTGATGAACTGTTAAGTTCTGATGACTCA
Chimpanzee              ATTCAGAAAGTTAATGAGTGGTTTTCCAGAAGTGATGAACTGTTAGGTTCTGATGACTCA
Bonobo                  ATTCAGAAAGTTAATGAGTGGTTTTCCAGAAGTGATGAACTGTTAGGTTCTGATGACTCA
Gorilla                 ATTCAGAAAGTTAATGAGTGGTTTTCCAGAAGTGATGAACTGTTAGGTTCTGATGACTCA
Orangutan               ATTCAGAAAGTTAATGAGTGGTTTTCCAGAAGTGACGAACTGTTAGGTTCTGATGACTCA
BorneoOrangutan         ATTCAGAAAGTTAATGAGTGGTTTTCCAGAAGTGACGAACTGTTAGGTTCTGATGACTCA
PileatedGibbon          ATTCAGAAAGTTAATGAGTGGTTTTCCAGAAGTGATGAACTGTTAAATTCTGATGACTCA
AgileGibbon             ATTCAGAAAGTTAATGAGTGGTTTTCCAGAAGTGATGAACTGTTAAATTCTGATGACTCA
WHGibbon                ATTCAGAAAGTTAATGAGTGGTTTTCCAGAAGTGATGAACTGTTAAATTCTGATGACTCA
Siamang                 ATTCAGAAAGTTAATGAGTGGTTTTCCAGAAGTGATGAACTGTTAAGTTCTGATGACTCA
RCGibbon                ATTCAGAAAGTTAATGAGTGGTTTTCCAGAAGTGATGAACTGTTAAGTTCTGATGACTCA
WCGibbon                ATTCAGAAAGTTAATGAGTGGTTTTCCAGAAGTGATGAACTGTTAAGTTCTGATGACTCA
Marmoset                ATTCAGAAAGTTAATGAGTGGTTTTCCAGAAGTGATGAACTGTTAACTTCTGATGACTCA
SqMnky                  ATTCAGAAAGTTAATGAGTGGTTTTCCAGAAGTGATGAACTGTTAACTTCTGATGACTCA
TitiMonkey              ATTCAGAAAGTTAATGAGTGGTTTTCCAGAAGTGATGAACTGTTAACTTCTGATGACTCA
HowlerMk                ATTCAGAAAGTTAATGAGTGGTTTTCCAGAAGTGATGAACTGTTAACTTCTGATGACTCA
                        *********************************** *********  *************

Human                   CATGATGGGGAGTCTGAATCAAATGCCAAAGTAGCTGATGTATTGGACGTTCTAAATGAG
Rhesus                  CATGATGGGGGGTCTGAATCAAATGCCAAAGTAGCTGATGTATTGGACGTTCTAAATGAG
CEMacaque               CATGATGGGGGGTCTGAATCAAATGCCAAAGTAGCTGATGTATTGGACGTTCTAAATGAG
BMangabey               CATGATGGGGGGTCTGAATCAAATGCCAAAGTAGCTGATGTATTGGACATTCTAAATGAG
OliveBaboon             CATGATGGGGGGTCTGAATCAAATGCCAAAGTAGCTGATGTATTGGACGTTCTAAATGAG
Talapoin                CATGATGGGGGGTCTGAATCAAATGCCAAAGTAGCTGATGTATTGGACGTTCTAAATGAG
WolfsGuenon             CATGATGGGGGGTCTGAATCAAATGCCAAAGTAGCTGATGTATTGGACGTTCTAAATGAG
Colobus                 CATGATGGGGGTTCTGAATCAAATGCCAAAGTAGCTGATGTATTGGATGTTCTGAATGAG
```

```
Chimpanzee          CATGATGGGGGGTCTGAATCAAATGCCAAAGTAGCTGATGTATTGGACGTTCTAAATGAG
Bonobo              CATGATGGGGGGTCTGAATCAAATGCCAAAGTAGCTGATGTATTGGACGTTCTAAATGAG
Gorilla             CATGATGGGGGGTCTGAATCAAATGCCAAAGTAGCTGATGTATTGGACGTTCTAAATGAG
Orangutan           CATGATGGGAGGTCTGAATCAAATGCCAAAGTAGCGGATGTATTGGACGTTCTAAATGAG
BorneoOrangutan     CATGATGGGAGGTCTGAATCAAATGCCAAAGTAGCGGATGTATTGGACGTTCTAAATGAG
PileatedGibbon      CATGATGGGGGGTCTGAATCAAATGCCAAAGTAGCTGATGTATTGGACGTTCTAAATGAG
AgileGibbon         CATGATGGGGGGTCTGAATCAAATGCCAAAGTAGCTGATGTATTGGACGTTCTAAATGAG
WHGibbon            CATGATGGGGGGTCTGAATCAAATGCCAAAGTAGCTGATGTATTGGACGTTCTAAATGAG
Siamang             CATGATGGGGGGTCTGAATCAAATGCCAAAGTAGCTGATGTATTGGACGTTCTAAATGAG
RCGibbon            CATGATGGGGGGTCTGAATCAAATGCCAAAGTAGCTGATGTATTGGACGTTCTAAATGAG
WCGibbon            CATGATGGGGGGTCTGAATCAAATGCCAAAGTAGCTGATGTATTGGACGTTCTAAATGAG
Marmoset            CATGACGAGGGGTCTGAATCAAATGCCAAAGTAGCTGAAGCATTGGAAGTTCTAAATGAA
SqMnky              CATGACGAGGGGTCTGAATCAAATGCCAAAGTAGCTGAAGCATTGGAAGTTCTAAATGAG
TitiMonkey          CATGATGGGGGGTCTGAATCAAATGCCAAAGTAGCTGAAGCATTGGAAGTTCTAAATGAG
HowlerMk            CATGATGGGGGGTCTGAATCAAATGCCAAAGTAGCTGAAGCATTGGAAGTTCTAAATGAG
                    ***** * *   *********************** ** * ******  **** *****

Human               GTAGATGAATATTCTGGTTCTTCAGAGAAAATAGACTTACTGGCCAGTGATCCTCATGAG
Rhesus              GTAGATGAATATTCTGGTTCTTCAGAGAAAATAGACTTACTGGCCAGTGATCCTCATGAG
CEMacaque           GTAGATGAATATTCTGGTTCTTCAGAGAAAATAGACTTACTGGCCAGTGATCCTCATGAG
BMangabey           GTAGATGAATATTCTGGTTCTTCAGAGAAAATAGACTTACTGGCCAGTGATCCTCATGAG
OliveBaboon         GTAGATGAATATTCTGGTTCTTCAGAGAAAATAGACTTACTGGCCAGTGATCCTCATGAG
Talapoin            GTAGATGAATATTCTGGTTCTTCAGAGAAAATAGACTTACTGGCCAGTGATCCTCATGAG
WolfsGuenon         GTAGATGAATATTCTGGTTCTTCAGAGAAAATAGACTTACTGGCCAGTGATCCTCATGAG
Colobus             GTAGATGAATATTCTGGTTCTTCAGAGAAAATAGACTTACTGGCCAGTGATCCTCATGAG
Chimpanzee          GTAGATGAATATTCTGGTTCTTCAAAGAAAATAGACTTACTGGCCAGCGATCCTCATGAG
Bonobo              GTAGATGAATATTCTGGTTCTTCAGAGAAAATAGACTTACTGGCCAGCGATCCTCATGAG
Gorilla             GTAGATGAATATTCTGGTTCTTCAGAGAAAATAGACTTACTGGCCAGTGATCCTCATGAG
Orangutan           GTAGATGAATATTCTGGTTCTTCAGAGAAAATAGACTTACTGGCCAGTGATCCTCATGAG
BorneoOrangutan     GTAGATGAATATTCTGGTTCTTCAGAGAAAATAGACTTATTGGCCAGTGATCCTCATGAG
PileatedGibbon      GTAGATGAATATTCTGGTTCTTCAGAGAAAATAGACTTACTGGCCAGTGATCCTCATGAG
AgileGibbon         GTAGATGAATATTCTGGTTCTTCAGAGAAAATAGACTTACTGGCCAGTGATCCTCATGAG
WHGibbon            GTAGATGAATATTCTGGTTCTTCAGAGAAAATAGACTTACTGGCCAGTGATCCTCATGAG
Siamang             GTAGATGAATATTCTGGTTCTTCAGAGAAAATAGACTTACTGGCCAGTGATCCTCATGAG
RCGibbon            GTAGACGAATATTCTGGTTCTTCAGAGAAAATAGACTTACTGGCCAGTGATCCTCATGAG
WCGibbon            GTAGACGAATATTCTGGTTCTTCAGAGAAAATAGACTTACTGGCCAGTGATCCTCATGAG
Marmoset            GTAGATGGATATTCTAGTTCTTCAGAAAAAATAGACTTACTGGCCAGTGATCCTCATGAT
SqMnky              GTAGATGGATATTCTAGTTCTTCAGAGAAAATAGACTTACTGGCCAGTGATCCTCATGAT
TitiMonkey          GTAGATGGATATTCTAGTTCTTCAGAGAAAATAGACTTACTGGCCAGTGATCCTCATGAT
HowlerMk            GTAGATGGATATTCTAGTTCTTCAGAGAAAATAGACTTACTGGCCAGTGATCCTCATGAT
                    ***** * ******* ******** * ************ ******* ***********

Human               GCTTTAATATGTAAAAGTGAAAGAGTTCACTCCAAATCAGTAGAGAGTAATATTGAAGAC
Rhesus              CCTTTAATATGTAAAAGTGAAAGAGTTCACTCCAGTTCAGTAGAGAGTAATATTAAAGAC
CEMacaque           CCTTTAATATGTAAAAGTGAAAGAGTTCACTCCAGTTCAGTAGAGAGTAATATTAAAGAC
BMangabey           CCTTTAATATGTAAAAGTGAAAGAGTTCACTCCAGTTCAGTAGAGATTAATATTAAAGAC
OliveBaboon         CCTTTAATATGTAAAAGTGAAAGAGTTCACTCCAGTTCAGTAGAGATTAATATTAAAGAC
Talapoin            CCTTTAATATGTAAAAGTGAAAGAGTTCACTCCAGTTCAGTAGAGAGTAGTATTGAAGAC
WolfsGuenon         CCTTTAATATGTAAAAGTGAAAGAGTTCACTCCAGTTCAGTAGAGCGTAGTATTGAAGAC
Colobus             CCTTTAATATGTAAAAGTGAAAGAGTTCACTCCAGTTCAGTAGAGAGTAATATTGAAGAC
Chimpanzee          GCTTTAATATGTAAAAGTGAAAGAGTTCACTCCAAATCAGTAGAGAGTAATACTGAAGAC
Bonobo              GCTTTAATATGTAAAAGTGAAAGAGTTCACTCCAAATCAGTAGAGAGTAATACTGAAGAC
Gorilla             GCTTTAATATGTAAAAGTGAAAGAGTTCACTCCAAATCAGTAGAGAGTAATATTGAAGAC
Orangutan           GCTTTAATTTGTAAAAGTGAAAGAGTTCACTCCAAATCAGTAGAGAGTAATATTGAAGAC
BorneoOrangutan     GCTTTAATTTGTAAAAGTGAAAGAGTTCACTCCAAATCAGTAGAGAGTAATATTGAAGAC
PileatedGibbon      TCTTTAATATGTAAAAGTGAAAGAGTTCACTCCAAATCAGTAGAGAGTAATATTGAAGAC
AgileGibbon         TCTTTAATATGTAAAAGTGAAAGAGTTCACTCCAAATCAGTAGAGAGTAATATTGAAGAC
WHGibbon            TCTTTAATATGTAAAAGTGAAAGAGTTCACTCCAAATCAGTAGAGAGTAATATTGAAGAC
Siamang             TCTTTAATATGTAAAAGTGAAAGAGTTCACTCCAAATCAGTAGAGAGTAATATTGAAGAC
RCGibbon            TCTTTAATATGTAAAAGTGAAAGAGTTCACTCCAAATCAGTAGAGAGTAATATTGAAGAC
WCGibbon            TCTTTAATATGTAAAAGTGAAAGAGTTCACTCCAAATCAGTAGAGAGTAATATTGAAGAC
Marmoset            GCTTTAATATGTAAAAGTGAAAGAGTTCATTGCAAATCAGTAGAGAGTAGTATTGAAGAT
SqMnky              GCTTCAATATGTAAAAGTGAAAGAGTTCACTGCAAATCAGTAGAGAGTAGTATTGAAGAT
TitiMonkey          CCTTTAATATGTAAAAGTGAAAGAGTTCACTGCATATCAGTAGAGAGTAGGATTGAAGAT
HowlerMk            CATTTGATATGTAAAAGTGAAAGAGTTCACTGCAAATCAGTAGAGAGTAGTATTGAAGAT
                      **  ** ******************** * **  *********  **  * * ****

Human               AAAATATTTGGGAAAACCTATCGGAAGAAGGCAAGCCTCCCCAACTTAAGCCATGTAACT
Rhesus              AAAATATTTGGGAAAACCTATCGGAGGAAGGCAAACCTTCCCAATTTAAGCCATGTAACT
CEMacaque           AAAATATTTGGGAAAACCTATCGGAGGAAGGCAAACCTTCCCAATTTAAGCCATGTAACT
BMangabey           AAAATATTTGGGAAAACCTATCGGAGGAAGGCAAACCGTCCCAATTTAAGCCATGTAACT
OliveBaboon         AAAATATTTGGGAAAACCTATCGGAGGAAGGCAAACCTTCCCAATTTAAGCCATGTAACT
Talapoin            AAAATATTTGGGAAAACCTATCGGAGGAAGGCAAACCTTCCCAATTTAAGCCATGTAACT
```

```
WolfsGuenon          AAAATATTTGGGAAAACCTATCGGAGGAAGGCAAACTTTCCCAATTTAAGCCATGTAACT
Colobus              AAAATATTTGGGAAAACCTATCGGAGGAAGGCAAACCTTCCCAATTTAAGCCATGTAACT
Chimpanzee           AAAATATTTGGGAAAACCTATCGGAGGAAGGCAAGCCTCCCCAACTTAAGCCATGTAACT
Bonobo               AAAATATTTGGGAAAACCTATCGGAGGAAGGCAAGCCTCCCCAACTTAAGCCATGTAACT
Gorilla              AAAATATTTGGGAAAACCTATCGGAGGAAGGCAAGCCTCCCCAGCTTAAGCCATGTAACT
Orangutan            AAAATATTTGGGAAAACCTATCGGAGGAAGGCAAGCCTCCCCAACTTAAGCCATGTAACT
BorneoOrangutan      AAAATATTTGGGAAAACCTATCGGAGGAAGGCAAGCCTCCCCAACTTAAGCCATGTAACT
PileatedGibbon       AAAATATTTGGGAAAACCTATCGGAGGAAGGCAAGCCTCCCCAACTTAAGCCATGTAACT
AgileGibbon          AAAATATTTGGGAAAACCTATCGGAGGAAGGCAAGCCTCCCCAACTTAAGCCATGTAACT
WHGibbon             AAAGTATTTGGGAAAACCTATCGGAGGAAGGCAAGCCTCCCCAACTTAAGCCATGTAACT
Siamang              AAAATATTTGGGAAAACCTATCGGAGGAAGGCAAGCCTCCCCAACTTAAGCCATGTAACT
RCGibbon             AAAATATTTGGGAAAACCTATCGGAGGAAGGCAAGCCTCCCCAACTTAAGCCATGTAACT
WCGibbon             AAAATATTTGGGAAAACCTATCGGAGGAAGGCAAGCCTCCCCAACTTAAGCCATGTAACT
Marmoset             AAAATATTTGGGAAAACCTATCGGAGGAAGGCAAGCCTCCCGAATTTGAGCCATGTAACT
SqMnky               AAAATATTTGGGAAAACCTATCGGAGGAAGGCAAGCCTCCCTAACTTGAGCCATGAAACT
TitiMonkey           AAAATATTTGGGAAAACCTATCGGAGGAAGGCAAGCCTCTCTAACTCAAGCCATGTAACT
HowlerMk             AAAATATTTGGGAAAACCTATCGGAGGAAGGCAAGCCTCCCTAACTTGAGCCACGTAACT
                     *** ********************* ******** *    * *  *  ***** * ****

Human                GAAAATCTAATTATAGGAGCATTTGTTACTGAGCCACAGATAATACAAGAGCGTCCCCTC
Rhesus               GAAAATCTAATTATAGGAGCACTTGTTACTGAGTCACAGATAATGCAAGAGCGTCCCCTC
CEMacaque            GAAAATCTAATTATAGGAGCACTTGTTACTGAGTCACAGATAATGCAAGAGCGTCCCCTC
BMangabey            GAAAATCTAATTATAGGAGCACTTGTTACTGAGTCAGAAATAATGCAAGAGCGTCCCCTC
OliveBaboon          GAAAATCTAATTATAGGAGCACTTGTTACTGAGTCAGAAATAATGCAAGAGCGTCCCCTC
Talapoin             GAAAATCTAATTATAGGAGCACTTGTTACTGAGTCACAGATAATGCAAGAGCGTCCCCTC
WolfsGuenon          GAAAATCTAATTATAGGAGCACTTGTTACTGAGTCACAGATAATGCAAGAGCGTCCCCTC
Colobus              GAAAATCTAATTATAGGAGCACTTGTTACTGAGTCACAGATAATGCAAGAGCGTCCCCTC
Chimpanzee           GAAAATCTAATTATAGGAGCATTTGTTACTGAGCCACAGATAATACAAGAGCGTCCCCTC
Bonobo               GAAAATCTAATTATAGGAGCATTTGTTACTGAGCCACAGATAATACAAGAGCGTCCCCTC
Gorilla              GAAAATCTAATTATAGGAGCATTTGTTACTGAGCCACAGATAATACAAGAGCGTCCCCTC
Orangutan            GAAAATCTAATTATAGGAGCATTTGTTACTGAGCCACAGATAATACAAGAGCGTCCCCTC
BorneoOrangutan      GAAAATCTAATTATAGGAGCATTTGTTACTGAGCCACAGATAATACAAGAGCGTCCCCTC
PileatedGibbon       GAAAATCTAATTATAGGAGCATTTGTTACTGAGCCACAGATAATACAAGAGCGTCCCCTC
AgileGibbon          GAAAATCTAATTATAGGAGCATTTGTTACTGAGCCACAGATAATACAAGAGCGTCCCCTC
WHGibbon             GAAAATCTAATTATAGGAGCATTTGTTACTGAGCCACAGATAATACAAGAGCGTCCCCTC
Siamang              GAAAATCTAATTATAGGAGCATTTGTTACTGAGCCACAGATAATACAAGAGCGTCCCCTC
RCGibbon             GAAAATCTAATTATAGGAGCATTTGTTACTGAGCCACAGATAATACAAGAGCGTCCCCTC
WCGibbon             GAAAATCTAATTATAGGAGCATTTGTTACTGAGCCACAGATAATACAAGAGCGTCCCCTC
Marmoset             GAAAATCTAATTATAGGAGCATTTGTTACTGAACCACAGATAACACAAGAGCATCCTCTC
SqMnky               GAAAATCTAATTATAGGAGCACTTGTTACTGAGCCACAGATAACACAAGAGCATCCCTTC
TitiMonkey           GAAAATCTAATTATAGGAGCATTTGTTACTGAGCCACAGATAACACAAGAGCATCCCCTC
HowlerMk             GAAAATCTAATTATAGGAGCATTTGTTACTGAGCCACAGATAATACAAGAGCATCCTCTC
                     ********************* **********  ** * ****  ******* ***  **

Human                ACAAATAAATTAAAGCGTAAAAGGAGACCTACATCAGGCCTTCATCCTGAGGATTTTATC
Rhesus               ACAAATAAATTAAAGCGTAAAAGGAGAACTACATCAGGTCTTCATCCTGAGGATTTTATA
CEMacaque            ACAAATAAATTAAAGCGTAAAAGGAGAACTACATCAGGTCTTCATCCTGAGGATTTTATA
BMangabey            ACAAATAAATTAAAGCGTAAAAGGAGAACTACATCAGGTCTTCATCCTGAGGATTTTATA
OliveBaboon          ACAAATAAATTAAAGCGTAAAAGGAGAACTACATCAGGTCTTCATCCTGAGGATTTTATA
Talapoin             ACAAACAAATTAAAGCGTAAAAGGAGAACTACATCAGGTCTTCATCCTGAGGATTTTATA
WolfsGuenon          ACAAACAAATTAAAGCGTAAAAGGAGAACTACATCAGGTCTTCATCCTGAGGATTTTATA
Colobus              ACAAATAAATTAAAGCGTAAAAGGAGAACTACATCAGGTCTTCATCCTGAGGATTTTATA
Chimpanzee           ACAAATAAATTAAAGCGTAAAAGGAGAGCTACATCAGGCCTTCATCCTGAGGATTTTATC
Bonobo               ACAAATAAATTAAAGCGTAAAAGGAGAGCTACATCAGGCCTTCATCCTGAGGATTTTATC
Gorilla              ACAAATAAATTAAAGCGTAAAAGGAGAGCTACATCAGGCCTTCATCCTGAGGATTTTATC
Orangutan            ACAAATAAATTAAAGCGTAAAAGGAGAGCTACATCAGGCCTTCATCCTGAGGATTTTATC
BorneoOrangutan      ACAAATAAATTAAAGCGTAAAAGGAGAGCTACATCAGGCCTTCATCCTGAGGATTTTATC
PileatedGibbon       ACAAATAAATTAAAGCATAAAAGGAGAGCTACATCAGGCCTTCATCCTGAGGATTTTATC
AgileGibbon          ACAAATAAATTAAAGCATAAAAGGAGAGCTACATCAGGCCTTCATCCTGAGGATTTTATC
WHGibbon             ACAAATAAATTAAAGCATAAAAGGAGAGCTACATCAGGCCTTCATCCTGAGGATTTTATC
Siamang              ACAAATAAATTAAAGCATAAAAGGAGAGCTACATCAGGCCTTCATCCTGAGGATTTTATC
RCGibbon             ACAAATAAATTAAAGCATAAAAGGAGAGCTACATCAGGCCTTCATCCTGAGGATTTTATC
WCGibbon             ACAAATAAATTAAAGCATAAAAGGAGAGCTACATCAGGCCTTCATCCTGAGGATTTTATC
Marmoset             ACAAATAAATTAAAGCGTAAAAGGAGAGTTACATCAGGACTTCATCCTGAGGATTTTATC
SqMnky               ACAAATAAATTAAAGCGTAAAAGGAGAGTTACATCAGGACTTCATCCTGAGGATTTTATC
TitiMonkey           ACAAATAAATTAAAGCGTAAAAGGAGAGTTACATCAGGACTTCATCCTGAGGATTTTATC
HowlerMk             ACAAATAAATTAAAGCGTAAAAGGAGAGTTACATCAGGACTTCATCCTGAGGATTTTATC
                     ***** ********** **********  ********* ******************** 

Human                AAGAAAGCAGATTTGGCAGTTCAAAAGACTCCTGAAATGATAAATCAGGGAACTAACCAA
Rhesus               AAGAAAGCAGATTTGGCAGTTCAAAAGACTCCTGAAATAATAAATCAGGGAACTAACCAA
CEMacaque            AAGAAAGCAGATTTGGCAGTTCAAAAGACTCCTGAAATAATAAATCAGGGAACTAACCAA
BMangabey            AAGAAAGCAGATTTGGCAGTTCAAAAGACTCCTGAAATAATAAATCAGGGAACTAACCAA
```

```
OliveBaboon          AAGAAAGCAGATTTGGCAGTTCAAAAGACTCCTGAAATAATAAATCAGGGAACTAACCAA
Talapoin             AAGAAAGCAGATGTGGCAGTTCAAAAGACTCCTGAAATAATAAATCAGGGAACTAACCAA
WolfsGuenon          AAGAAAGCAGATTTGGCAGTTCAAAAGACTCCTGAAATAATTAATCAGGGAACTAACCAA
Colobus              AAGAAAGCAGATTTGGCAGTTCAAAAGACTCCTGAAATGATAAATCAGGGAACTAACCAA
Chimpanzee           AAGAAAGCAGATTTGGCAGTTCAAAAGACTCCTGAAATGATAAATCAGGGAACTAACCAA
Bonobo               AAGAAAGCAGATTTGGCAGTTCAAAAGACTCCTGAAATGATAAATCAGGGAACTAACCAA
Gorilla              AAGAAAGCAGATTTGGCAGTTCAAAAGACTCCTGAAATGATAAATCAGGGAACTAACCAA
Orangutan            AAGAAAGCAGATTTGGCAGTTCAAAAGACTCCTGAAATGATAAATCAGGGAACTAACCAA
BorneoOrangutan      AAGAAAGCAGATTTGGCAGTTCAAAAGACTCCTGAAATGATAAATCAGGGAACTAACCAA
PileatedGibbon       AAGAAAGCAGATTTGGCAGTTCAAAAGACTCCTGAAATGATAAATCAGGGAACTAACCAA
AgileGibbon          AAGAAAGCAGATTTGGTAGTTCAAAAGACTCCTGAAATGATAAATCAGGGAACTAACCAA
WHGibbon             AAGAAAGCAGATTTGGCAGTTCAAAAGACTCCTGAAATGATAAATCAGGGAACTAACCAA
Siamang              AAGAAAGCAGATTTGGCAGTTCAAAAGACTCCTGAAATGATAAATCAGGGAACTAACCAA
RCGibbon             AAGAAAGCAGATTTGGCAGTTCAAAAGACTCCTGAAATGATAAATCAGGGAACTAACCAA
WCGibbon             AAGAAAGCAGATTTGGCAGTTCAAAAGACTCCTGAAATGATAAATCAGGGAACTAACCAA
Marmoset             AAGAAAGCAGATTTGGCAGTTCAAAAGACTCCTGAAAAGATAAATCAGGGAACTAACCAA
SqMnky               AAGAAAGCAGATTTGGCAGTTCAAAAGACTCCTGAAAAGATAAATCAGGGAACTAACCAA
TitiMonkey           AACAAAGCAGATTCAACAGTTCAAAAGACTCCTGAAAAGATAAATCAGGGAACTAACCAA
HowlerMk             AAGAAAGCAGATTTGGCAGTTCAAAAGACTCCTGAAAAGATAAATCAGGGAACTAACCAA
                     ** *********     ********************  ** ******************

Human                ACGGAGCAGAATGGTCAAGTGATGAATATTACTAATAGTGGTCATGAGAATAAAACAAAA
Rhesus               ATGGAGCAGAATGGTCAAGTGATGAATATTACTAATAGTGCTCATGAGAATAAAACAAAA
CEMacaque            ATGGAGCAGAATGGTCAAGTGATGAATATTACTAATAGTGCTCATGAGAATAAAACAAAA
BMangabey            ATGGAGCAGAATGGTCAAGTGATGAATATTACTAATAGTGCTCATGAGAATAAAACAAAA
OliveBaboon          ATGGAGCAGAATGGTCAAGTGATGAATATTACTAATAGTGCTCATGAGAATAAAACAAAA
Talapoin             ATGGAGCAGAATGGTCAAGTGATGAATATTACTAATAGTGCTCATGAGAATAAAACAAAA
WolfsGuenon          ATGGAGCAGAATGATCAAGTGATGAATATTACTAATAGTGCTCATGAGAATAAAACAAAA
Colobus              ATGGAGCAGAATGGTCAAGTGATGAATATTACTAATAGTGCTCATGAGAATAAAACAAAA
Chimpanzee           ATGGAGCAGAATGGTCAAGTGATGAATATTACTAATAGTGGTCATGAGAATAAAACAAAA
Bonobo               ATGGAGCAGAATGGTCAAGTGATGAATATTACTAATAGTGGTCATGAGAATAAAACAAAA
Gorilla              ATGGAGCAGAATGGTCAAGTGATGAATATTACTAATAGTGGTCATGAGAATAAAACAAAA
Orangutan            ATGGAGCAGAATGGTCAAGTGATGAATATTACTAATAGTGGTCATGAGAATAAAACAAAA
BorneoOrangutan      ATGGAGCAGAATGGTCAAGTGATGAATATTACTAATAGTGGTCATGAGAATAAAACAAAA
PileatedGibbon       ATGGAGCAGAATGTTCAAGTGATGAATATTACTAATAGTGGTCATGAGAATAAAACAATA
AgileGibbon          ATGGAGCAGAATGTTCAAGTGATGAATATTACTAATAGTGGTCATGAGAATAAAACAATA
WHGibbon             ATGGAGCAGAATGTTCAAGTGATGAATATTACTAATAGTGGTCATGAGAATAAAACAATA
Siamang              ATGGAGCAGAATGTTCAAGTGATGAATATTACTAATAGTGGTCATGAGAATAAAACAATA
RCGibbon             ATGGAGCAGAATGTTCAAGTGATGAATATTACTAATAGTGGTCATGAGAATAAAACAATA
WCGibbon             ATGGAGCAGAATGTTCAAGTGATGAATATTACTAATAGTGGTCATGAGAATAAAACAATA
Marmoset             ATGGAGCGGAATGATCAAGTGATGAATATTACTAACAGTGGCCATGAGAATAAAACAAAA
SqMnky               ATGGAGCAGAATGATCAAGTGATGAATATTACTAACAGTGGTCATGAGAATAAAACAAAA
TitiMonkey           ATGGAGCAGAATGATCAAGTGATGAATATTACGAACAGTGGTCATGAGAATAAAACAAAA
HowlerMk             ACAGAGCGGAATGATCAAGTGATGAATATTACTAACAGTGGTCATGAGAATAAAACAAAA
                     *  **** ***** ****************** ** ****  **************** *

Human                GGTGATTCTATTCAGAATGAGAAAAATCCTAACCCAATAGAATCACTCGAAAAAGAATCT
Rhesus               GGTGATTCTATTCAGAATGAGAAAAATCCTAACGCAATAGAATCACTGGAAGAAGAATCT
CEMacaque            GGTGATTCTATTCAGAATGAGAAAAATCCTAACGCAATAGAATCACTGGAAGAAGAATCT
BMangabey            GGTGATTCTATTCAGAATGAGAAAAATCCTAACCCAATAGAATCACTGGAAGAAGAATCT
OliveBaboon          GGTGATTCTATTCAGAATGAGAAAAATCCTAACCCAATAGAATCACTGGAAGAAGAATCT
Talapoin             GGTGATTCTATTCAGAATGAGAAAAATCCTAACCCAATAGAATCACTGGAAGAAGAATCT
WolfsGuenon          GGTGATTCTATTCAGAATGAGAAAAATCCTAACCCAATAGAATCACTGGAAGAAGAATCT
Colobus              GGTGATTCTATTCAGAATGAGAAAAATCCTGACCCAATAGAATCACTGGAAGAAGAATCT
Chimpanzee           GGTGATTCTATTCAGAATGAGAAAAATCCTAACCCAATAGAATCACTCGAAAAAGAATCT
Bonobo               GGTGATTCTATTCAGAATGAGAAAAATCCTAACCCAATAGAATCACTCGAAAAAGAATCT
Gorilla              GGTGATTCTATTCAGAATGAGAAAAATCCTAACCCAATAGAATCACTAGAAAAAGAATCT
Orangutan            GGTGATTCTATTCAGAATGAGAAAAATCCTAACCCAATAGAATCACTCGAAAAAGAATCT
BorneoOrangutan      GGTGATTCTATTCAGAATGAGAAAAATCCTAACCCAATAGAATCACTCGAAAAAGAATCT
PileatedGibbon       CGTGATTCTATTCAGAATGAGAAAAATCCTAACCCAATAGAATCACTCGAAAAAGAATCT
AgileGibbon          CGTGATTCTATTCAGAATGAGAAAAATCCTAACCCAATAGAATCACTCGAAAAAGAATCT
WHGibbon             CGTGATTCTATTCAGAATGAGAAAAATCCTAACCCAATAGAATCACTCGAAAAAGAATCT
Siamang              CGTGATTCTATTCAGAATGAGAAAAATCCTAACCCAATAGAATCACTCGAAAAAGAATCT
RCGibbon             CGTGATTCTATTCAGAATGAGAAAAATCCTAACCCAATAGAATCACTCGAAAAAGAATCT
WCGibbon             CGTGATTCTATTCAGAATGAGAAAAATCCTAACCCAATAGAATCACTCGAAAAAGAATCT
Marmoset             GGTGATTCTATTCAGAATGAGAACAATCCTAACCCAGTAGAATCACTGGAAAAAGAATCT
SqMnky               GATGATTCTATTCAGAATGTGAACAATCCTAACCCAGTAGAATCACTGGAAAAAGAATCT
TitiMonkey           GGTGATTCTATTCAGAATGAGAACAATCCTAACCCAGTAGAATCACTGGAAAAAGAATCT
HowlerMk             GGTGATTCTATTCAGAATGAGAACAATCCTAACCCAGTAGAATCACTGGAAAAAGAATCA
                       ***************** *** ****** ** ** ********** *** ******* 

Human                GCTTTCAAAACGAAAGCTGAACCTATAAGCAGCAGTATAAGCAATATGGAACTCGAATTA
Rhesus               GCTTTCAAAACTAAAGCTGAACCTATAAGCAGCAGTATAAACAATATGGAACTAGAATTA
```

```
CEMacaque              GCTTTCAAAACTAAAGCTGAACCTATAAGCAGCAGTATAAACAATATGGAACTAGAATTA
BMangabey              GCTTTCAAAACTAAAGCTGAACCTATAAGCAGCAGTATAAACAATATGGAACTAGAATTA
OliveBaboon            GCTTTCAAAACTAAAGCTGAACCTATAAGCAGCAGTATAAACAATATGGAACTAGAATTA
Talapoin               GCTTTCAAAACTAAAGCTGAACCTATAAGCAGCAGTATAAACAATATGGAACTAGAATTA
WolfsGuenon            GCTTTCAAAACTAAAGCTGAACCTATAAGCAGCAGTATAAACAATATGGAACTAGAATTA
Colobus                GCTTTCAAAACTAAAGCTGAACCTATAAGCAGCAGTATAAACAATATGGAACTAGAATTA
Chimpanzee             GCTTTCAAAACGAAAGCTGAACCTATAAGCAGCGGTATAAGCAATATGGAACTCGAATTA
Bonobo                 GCTTTCAAAACGAAAGCTGAACCTATAAGCAGCAGTATAAGCAATATGGAACTCGAATTA
Gorilla                GCTTTCAAAACGAAAGCTGAACCTATAAGCAGCTGTATAAGCAATATGGAACTCGAATTA
Orangutan              GCTTTCAAAACAAAAGCTGAACCTATAAGCAGCAGTATAAGCAATATGGAACTCGAATTA
BorneoOrangutan        GCTTTCAAAACAAAAGCTGAACCTATAAGCAGCAGTATAAGCAATATGGAACTCGAATTA
PileatedGibbon         GCTTTCAAAGCGAAAGCTGAACCTATAAGCAGCAGTATAAGCAATATGGAACTCGAATTA
AgileGibbon            GCTTTCAAAGCGAAAGCTGAACCTATAAGCAGCAGTATAAGCAATATGGAACTCGAATTA
WHGibbon               GCTTTCAAAGCGAAAGCTGAACCTATAAGCAGCAGTATAAGCAATATGGAACTCGAATTA
Siamang                GCTTTCAAAGCGAAAGCTGAACCTATAAGCAGCAGTATAAGCAATATGGAACTCGAATTA
RCGibbon               GCTTTCAAAGCGAAAACTGAACCTATAAGCAGCAGTATAAGCAATATGGAACTCGAATTA
WCGibbon               GCTTTCAAAGCGAAAACTGAACCTATAAGCAGCAGTATAAGCAATATGGAACTCGAATTA
Marmoset               ---TTCAGAAGTAAAGCTGAACCTATAAGCAGTAGTATAAGCAATATGGAACTAGAATTA
SqMnky                 ---TTCAGAAGTAAAGCTGAACCTATAAGCAGTAGTATAAGCAATATGGAACAAGAATTA
TitiMonkey             ---TTCAGAAGTAAAGCTGAGCCTATAAGCAGTAGGATAAGCAATATGGAACTAGAATTA
HowlerMk               ---TTCAAAAGTAAAGCTGAACCTATAAGCAGTAGTATAAGCAATATGGAATTAGAATTG
                          **** *   *** **** ***********  * **** **********   *****

Human                  AATATCCACAATTCAAAAGCACCTAAAAAGAATAGGCTGAGGAGGAAGTCTTCTACCAGG
Rhesus                 AATATCCACAATTCAAAAGCACCTAAAAAAAATAGGCTGAGGAGGAAGTCTTCTACCAGG
CEMacaque              AATATCCACAATTCAAAAGCACCTAAAAAAAATAGGCTGAGGAGGAAGTCTTCTACCAGG
BMangabey              AATATCCACAATTCAAAAGCACCTAAGAAAAATAGGCTGAGGAGGAAGTCTTCTACCAGG
OliveBaboon            AATATCCACAATTCAAAAGCACCTAAGAAAAATAGGCTGAGGAGGAAGTCTTCTACCAGG
Talapoin               AATATCCACAATTCAAAAGCACCTAAAAAAAATAGACTGAGGAGGAAGTCTTCTACCAGG
WolfsGuenon            AATATCCACAATTCAAAAGCACCTAAAAAAAATAGGCTGAGGAGGAAGTCTTCTACCAGG
Colobus                AATATCCACAATTCAAAAGCACCTAAAAAGAATAGGCTGAGGAGGAAGTCTTCTACCAGG
Chimpanzee             AATATCCACAATTCAAAAGCACCTAAAAAGAATAGGCTGAGGAGGAAGTCTTCTACCAGG
Bonobo                 AATATCCACAATTCAAAAGCACCTAAAAAGAATAGGCTGAGGAGGAAGTCTTCTACCAGG
Gorilla                AATATCCACAATTCAAAAGCGCCTAAAAAGAATAGGCTGAGGAGGAAGTCTTCTACCAGG
Orangutan              AATATCCATAATTCAAAAGCACCTAAAAAGAATAGGCTGAGGAGGAAGTCTTCTACCAGG
BorneoOrangutan        AATATCCATAATTCAAAAGCACCTAAAAAGAATAGGCTGAGGAGGAAGTCTTCTACTAGG
PileatedGibbon         AATATCCACAATTCAAAAGCACCTAAAAAGAATAGGCTGAGGAGGAAGTCTTCTACCAGG
AgileGibbon            AATATCCACAATTCAAAAGCACCTAAAAAGAATAGGCTGAGGAGGAAGTCTTCTACCAGG
WHGibbon               AATATCCACAATTCAAAAGCACCTAAAAAGAATAGGCTGAGGAGGAAGTCTTCTACCAGG
Siamang                AATATCCACAATTCAAAAGCACCTAAAAAGAATAGGCTGAGGAGGAAGTCTTCTACCAGG
RCGibbon               AATATCCACAATTCAAAAGCACCTAAAAAGAATAGGCTGAGGAGGAAGTCTTCTACCAGG
WCGibbon               AATATCCACAATTCAAAAGCACCTAAAAAGAATAGGCTGAGGAGGAAGTCTTCTACCAGG
Marmoset               AATATCCACAATTCCAAAGCATCTAAAAAGAATAGGCTGAGGAGGAAGTCTTCTACCAGG
SqMnky                 AATATCCACAATTCCAAAGCATCTAAAAAGAATAGGCTGAGGAGGAAGTCTTCCACCAGG
TitiMonkey             AATGTCCACAATTCCAAAGCATCTAAAAAGAATAGGCTGAGGAGGAAGTCTTCTACCAGG
HowlerMk               AATGTCCACAATTCCAAAGCATCTAAAAAGAATAGGCTGAGAAGGAAGTCTTCTACCAGG
                       *** **** ***** *****  **** ** ***** ***** *********** ** ***

Human                  CATATTCATGCGCTTGAACTAGTAGTCAGTAGAAATCTAAGCCCACCTAATTGTACTGAA
Rhesus                 CATATTCATGCGCTTGAACTAGTAGTCAGTAGAAATCTAAGCCCACCTAATTGTACTGAA
CEMacaque              CATATTCATGCGCTTGAACTAGTAGTCAGTAGAAATCTAAGCCCACCTAATTGTACTGAA
BMangabey              CATATTCATGCGCTTGAACTAGTAGTCAGTAGAAATCTAAGCCCACCTAATTGTACTGAA
OliveBaboon            CATATTCATGCGCTTGAACTAGTAGTCAGTAGAAATCTAAGCCCACCTAATTGTACTGAA
Talapoin               CATATTCATGCGCTTGAACTAGTAGTCAGTAGAAATCTAAGCCCACCTAATTGTACTGAA
WolfsGuenon            CATATTCATGCGCTTGAACTAGTAGTCAGTAGAAATCTAAGCCCACCTAATTGTACTGAA
Colobus                CATATTCATGCGCTTGAACTAGTAGTCAGTAGAAATCTAAGCCCACCTAATTGTACTGAA
Chimpanzee             CATATTCATGCGCTTGAACTAGTAGTCAGTAGAAATCTAAGCCCACCTAATTGTACTGAA
Bonobo                 CATATTCATGCGCTTGAACTAGTAGTCAGTAGAAATCTAAGCCCACCTAATTGTACTGAA
Gorilla                CATATTCATGCGCTTGAACTAGTAGTCAGTAGAAATCTAAGCCCACCTAATTGTACTGAA
Orangutan              CATATTCATGCGCTTGAACTAGTAGTCAGTAGAAATCTAAGCCCACCTAATTGTACTGAA
BorneoOrangutan        CATATTCATGCGCTTGAACTAGTAGTCAGTAGAAATCTAAGCCCACCTAATTGTACTGAA
PileatedGibbon         CATATTCATGCGCTTGAACTAGTAGTCAGTAGAAATCTAAGTCCACCTAATTGTACTGAA
AgileGibbon            CATATTCATGCGCTTGAACTAGTAGTCAGTAGAAATCTAAGTCCACCTAATTGTACTGAA
WHGibbon               CATATTCATGCGCTTGAACTAGTAGTCAGTAGAAATCTAAGTCCACCTAATTGTACTGAA
Siamang                CATATTCATGCGCTTGAACTAGTAGTCAGTAGAAATCTAAGTCCACCTAATTGTACTGAA
RCGibbon               CATATTCATGCGCTTGAACTAGTTGTCAGTAGAAATCTAAGTCCACCTAATTGTACTGAA
WCGibbon               CATATTCATGCGCTTGAACTAGTAGTCAGTAGAAATCTAAGTCCACCTAATTGTACTGAA
Marmoset               CATATTCATGAGCTTGAATTAGTAGTCAGTAGAAATCTAAGCCCACCTAATTATACTGAA
SqMnky                 CATATTCATGAGCTTGAATTAGTAGTCAGTAGAAATCTAAGCCCACCTAATTATACTGAA
TitiMonkey             CATATTCCTGAGCTTGAACTGGTAGTCAGTAGAAATCTAAGCCCACCTAATTATACTGAA
HowlerMk               CATATTCATGAGCTTGAACTAGTAGTCAGTAGAAATCTAAGCCCACCTAATTATACTGAA
                       ******* ** ******* * ** ***************** ********** *******
```

```
Human               TTGCAAATTGATAGTTGTTCTAGCAGTGAAGAGATAAAGAAAAAAAAGTACAACCAAATG
Rhesus              CTACAAATTGATAGTTGTTCTAGCAGTGAAGAGATAAAGAAAAAAAATTACAACCAAATG
CEMacaque           CTACAAATTGATAGTTGTTCTAGCAGTGAAGAGATAAAGAAAAAAAATTACAACCAAATG
BMangabey           CTACAAATTGATAGTTGTTCTAGCAGTGAAGAGATAAAGAAAAAAAATTACAACCAAATG
OliveBaboon         CTACAAATTGATAGTTGTTCTAGCAGTGAAGAGATAAAGAAAAAAAATTACAACCAAATG
Talapoin            CTACAAATTGATAGTTGTTCTAGCAGTGAAGAGATAAAGAAAAAAAATTACAACCAAGGG
WolfsGuenon         CTACAAATTGATAGTTGTTCTAGCAGTGAAGAGATAAAGAAAAAAAATTACAACCAAGTG
Colobus             CTACAAATTGATAGTTGTTCTAGCAGTGAAGAGATAAAGAAAAAAAATTACAACCAAATG
Chimpanzee          TTGCAAATTGATAGTTGTTCTAGCAGTGAAGAGATAAAGAAAAAAAAGTACAACCAAATG
Bonobo              TTGCAAATTGATAGTTGTTCTAGCAGTGAAGAGATAAAGAAAAAAAAGTACAACCAAATG
Gorilla             TTGCAAATTGATAGTTGTTCTAGCAGTGAAGAGATAAAGAAAAAAAAGTACAACCAAATG
Orangutan           TTGCAAATTGATAGTTGTTCTAGCAGTGAAGAGATAAAGAAAAAAAAATACAACCAAATG
BorneoOrangutan     TTGCAAATTGATAGTTGTTCTAGCAGTGAAGAGATAAAGAAAAAAAAATACAACCAAATG
PileatedGibbon      TTACAAATTGATAGTTGTTCTAGCAGTGAAGAGAAAAAGAAAAAAAAGTACAACCAAATG
AgileGibbon         TTACAAATTGATAGTTGTTCTAGCAGTGAAGAGAAAAAGAAAAAAAAGTACAACCAAATG
WHGibbon            TTACAAATTGATAGTTGTTCTAGCAGTGAAGAGAAAAAGAAAAAAAAGTACAACCAAATG
Siamang             TTACAAATTGATAGTTGTTCTAGCAGTGAAGAGAAAAAGAAAAAAAAGTACAACCAAATG
RCGibbon            TTACAAATTGATAGTTGTTCTAGCAGTGAAGAGAAAAAGAAAAAAAAGTACAACCAAATG
WCGibbon            TTACAAATTGATAGTTGTTCTAGCAGTGAAGAGAAAAAGAAAAAAAAGTACAACCAAATG
Marmoset            GTACAAATTGATAGTTGTTCTAGCAGTGAAGAGCTAAAGAAAAAAAATTACAACCAAATG
SqMnky              GTACAAATTGATAGTTGTTCTAGCAGTGAAGAGATAAAGAAAAAAAATTACAACCAAATG
TitiMonkey          GTACAAATTGATAATTGTTCTAGCAGTGAAGAGATAAAGGAAAAAAATTACAACCAAATG
HowlerMk            GTACAAATTGATAGTTGTTCTAGCAGTGAAGAGATAAAGAAAAAAAATTACAACCAAATG
                     * ********** *******************  **** ******* *********  *

Human               CCAGTCAGGCACAGCAGAAACCTACAACTCATGGAAGGTAAAGAACCTGCAACTGGAGCC
Rhesus              CCAGTCAGGCACAGCAGAAACCTACAACTCATGGAAGATAAAGAATCTGCAACTGGAGCC
CEMacaque           CCAGTCAGGCACAGCAGAAACCTACAACTCATGGAAGATAAAGAATCTGCAACTGGAGCC
BMangabey           CCAGTCAGGCACAGCAGAAACCTACAACTCATGGAAGATAAAGAATCTGCAACTGGAGCC
OliveBaboon         CCAGTCAGGCACAGCAGAAACCTACAACTCATGGAAGATAAAGAATCTGCAACTGGAGCC
Talapoin            CCAGTCAGGCACAGCAGAAACCTACAACTCATGGAAAATAAAGAATCTGCAACTGGAGCC
WolfsGuenon         CCAGTCAGGCACAGCAGAAACCTACAACTCATGGAAGATAAAGAATCTGCAACTGGAGCC
Colobus             CCAGTCAGGCACAGCAGAAACCTACAACTCATGGAAGATAAAGAATCTGCAACTGGAGCC
Chimpanzee          CCAGTCAGGCACAGCAGAAACCTACAACTCATGGAAGATAAAGAACCTGCAACTGGAGTC
Bonobo              CCAGTCAGGCACAGCAGAAACCTACAACTCATGGAAGATAAAGAACCTGCAACTGGAGTC
Gorilla             CCAGTCAGGCACAGCAGAAACCTACAGCTCATGGAAGATAAAGAACCTGCAACTGGAGCC
Orangutan           CCAGTCAGGCACAGCAGAAACCTACAACTCATGGAAGATAAAGAACCTGCAACTGGAGCC
BorneoOrangutan     CCAGTCAGGCACAGCAGAAACCTACAACTCATGGAAGATAAAGAACCTGCAACTGGAGCC
PileatedGibbon      CCAGTCAGGCACAGCAGAAACCTACAACTCATGGAAGATAAAGAACCTGCAACTGGAGCT
AgileGibbon         CCAGTCAGGCACAGCAGAAACCTACAACTCATGGAAGATAAAGAACCTGCAACTGGAGCT
WHGibbon            CCAGTCAGGCACAGCAGAAACCTACAACTCATGGAAGATAAAGAACCTGCAACTGGAGCT
Siamang             CCAGTCAGGCACAGCAGAAACCTACAACTCATGGAAGATAAAGAACCTGCAACTGGAGCT
RCGibbon            CCAGTCAGGCACAGCAGAAACCTACAACTCATGGAAGATAAAGAACCTGCAACTGGAGCT
WCGibbon            CCAGTCAGGCACAGCAGAAACCTACAACTCATGGAAGATAAAGAACCTGCAACTGGAGCT
Marmoset            CCAGTCAGGCACAGCAGAAAGCTACAACTCATGGAAGATAAAGAACGTTCAACTGGAGCC
SqMnky              CCAGTCAGGCACAGCAGAAAACCACAACTCGTGGAAGATAAAGAACATGCAACTGGAGCC
TitiMonkey          CCAGTCAGGCACAGCAGAAAGCTACAACTCATGGAAGATAAAGAACGTGCAACTGGAGCC
HowlerMk            CCAGTCAGGCACAGCAGAAAGCTACAACTCATGGAAGATAAAGAACGTGCAGCTAGAGCC
                    ******************** * *** *** *****  *******  * ** ** ***

Human               AAGAAGAGTAACAAGCCAAATGAACAGACAAGTAAAAGACATGACAGCGATACTTTCCCA
Rhesus              AAGAAGAGTAACAAGCCAAACGAACAGACAAGTAAAAGACATGCCAGTGATACTTTCCCA
CEMacaque           AAGAAGAGTAACAAGCCAAATGAACAGACAAGTAAAAGACATGCCAGTGATACTTTCCCA
BMangabey           AAGAAGAGTAACAAGCCAAATGAACAGACAAGTAAAAGACATGCCAGTGATACTTTCCCA
OliveBaboon         AAGAAGAGTAACAAGCCAAATGAACAGACAAGTAAAAGACATGCCAGTGATACTTTCCCA
Talapoin            AAGAAGAGTAACAAGCCAAATGAACAGACAAGTAAAAGACATGCCAGTGATACTTTCCCA
WolfsGuenon         AAAAAGAGTAACAAGCCAAATGAACAGACAAGTAAAAGACATGCCAGTGATACTTTCCCA
Colobus             AAGAAGAGTAACAAGCCAAATGAACAGACAAGTAAAAGACATGCCAGTGATACTTTCCCA
Chimpanzee          AAGAAGAGTAACAAGCCAAATGAACAGACAAGTAAAAGACATGACAGCGATACTTTCCCA
Bonobo              AAGAAGAGTAACAAGCCAAATGAACAGACAAGTAAAAGACATGACAGCGATACTTTCCCA
Gorilla             AAGAAGAGTAACAAGCCAAATGAACAGACAAGTAAAAGACATGACAGCGATACTTTCCCA
Orangutan           AAGAAGAGTAACAAGCCAAATGAACAGACAAGTAAAAGACATGACAGCGATACTTTCCCA
BorneoOrangutan     AAGAAGAGTAACAAGCCAAATCAACAGACAAGTAAAAGACATGACAGCGATACTTTCCCA
PileatedGibbon      AAGAAGAGTAACAAGCCAAATGAACAGACAAGTAAAAGACATGACAGCGATACTTTCCCA
AgileGibbon         AAGAAGAGTAACAAGCCAAATGAACAGACAAGTAAAAGACATGACAGCGATACTTTCCCA
WHGibbon            AAGAAGAGTAACAAGCCAAATGAACAGACAAGTAAAAGACATGACAGCGATACTTTCCCA
Siamang             AAGAAGAGTAACAAGCCAAATGAACAGACAAGTAAAAGACATGACAGCGATACTTTCCCA
RCGibbon            AAGAAGAGTAACAAGCCAAATGAACAGACAAGTAAAAGACCTGACAGCGATACTTTCCCA
WCGibbon            AAGAAGAGTAACAAGCCAAATGAACAGACAAGTAAAAGACCTGACAGCGATACTTTCCCA
Marmoset            AAAAAGAGTAGCAAGTCAAATGAACAA---AGTAAAAGACATGCCAGTGATACTTTCCCA
SqMnky              AAAAAGAGTAGCCAGCCAAATGAACAAACAAATAAAAGACATGCCAGTGATACTTTCCCA
TitiMonkey          AAAAAGAGTAGCAAGCCAAATGAACAAACAAGTAAAAGACATGCCAGTGATACTTTTCCA
HowlerMk            AAAAAGAGTAGCAAGCCAAATGAACAAACAAGTAAAAGACATGCCAGTGATACTTTCCCA
```

```
                       ** ******* * ** ****  ****    * ******** ** *** ******** ***

Human                  GAGCTGAAGTTAACAAATGCACCTGGTTCTTTTACTAAGTGTTCAAATACCAGTGAACTT
Rhesus                 GAACTGAAGTTAACAAAGGTACCTGGTTCTTTTACTAACTGTTCAAATACTAGTGAACTT
CEMacaque              GAACTGAAGTTAACAAAGGTACCTGGTTCTTTTACTAACTGTTCAAATACTAGTGAACTT
BMangabey              GAACTGAAGTTAACAAAGGTACCTGGTTCTTTTACTAACTGTTCAAATACTAGTGAACTT
OliveBaboon            GAACTGAAGTTAACAAAGGTACCTGGTTCTTTTACTAACTGTTCAAATACTAGTGAACTT
Talapoin               GAACTGAAGTTAACAAAGGTACCTGGTTCTTTTACTAACTGTTCAAATACTAGTGAACTT
WolfsGuenon            GAACTGAAGTTAACAAAGGTACCTGGTTCTTTTACTAACTGTTCAAATACTAGTGAACTT
Colobus                GAACTGAAGTTAGCAAAGGTACCTCGTTCTTTTACTAACTGTTCAAATACTAGTGAACTT
Chimpanzee             GAGCTGAAGTTAACAAATGCACCTGGTTCTTTTACTAACTGTTCAAATACCAGTGAACTT
Bonobo                 GAGCTGAAGTTAACAAATGCACCTGGTTCTTTTACTAACTGTTCAAATACCAGTGAACTT
Gorilla                GAGCTGAAGTTAACAAATGCACCTGGTTCTTTTACTAACTGTTCAAATACCAGTGAACTT
Orangutan              GAGCTGAAGTTAACAAATGCACCTGGTTCTTTTACTAACTGTTCAAATACCAGTGAGCTT
BorneoOrangutan        GAGCTGAAGTTAACAAATGCACCTGGTTCTTTTACTAACTGTTCAAATACCAGTGAGCTT
PileatedGibbon         GAGCTGAAGTTAACAAATGCACCTGGTTCTTTTACTAACTGTTCAAATACCAGTGAACTT
AgileGibbon            GAGCTGAAGTTAACAAATGCACCTGGTTCTTTTACTAACTGTTCAAATACCAGTGAACTT
WHGibbon               GAGCTGAAGTTAACAAATGCACCTGGTTCTTTTACTAACTGTTCAAATACCAGTGAACTT
Siamang                GAGCTGAAGTTAACAAATGCACCTGGTTCTTTTACTAACTGTTCAAATACCAGTGAACTT
RCGibbon               GAGCTGAAGTTAACAAATGCACCTGGTTCTTTCACTAACTGTTCAAATACCAGTGAACTT
WCGibbon               GAGCTGAAGTTAACAAATGCACCTGGTTCTTTCACTAACTGTTCAAATACCAGTGAACTT
Marmoset               GAACTGAGGTTAACAAACATACCTGATTCTTTTACTAACTGTTCAAATACTAATGAACTT
SqMnky                 GAGCCGAGGTTAACAAACATACCTGGTTCTTTTACTAACTGTTCAAATACTAATGAACTT
TitiMonkey             GAACTGAGGTTAACAAACATACCTGGTTCTTTTACTAACTGTCCAAATACTGATGAACTT
HowlerMk               GAACTGAGGTTAACAAACATACCTGGTTCTTTTACTAACTGTTCAAATACTAATGAATTT
                       ** * ** **** ****   ****  ****** ***** *** *******   ***  **

Human                  AAAGAATTTGTCAATCCTAGCCTTCCAAGAGAAGAAAAAGAAGAGAAACTAGAAACAGTT
Rhesus                 AAAGAATTTGTCAATCCTAGCCTTTCAAGAGAAGAAAAAGAAGAGAAACTAGAAACAGTT
CEMacaque              AAAGAATTTGTCAATCCTAGCCTTTCAAGAGAAGAAAAAGAAGAGAAACTAGAAACAGTT
BMangabey              AAAGAATTTGTCAATCCTAGCCTTTCAAGAGAAGAAAAAGAAGAGAAACTAGAAACAGTT
OliveBaboon            AAAGAATTTGTCAATCCTAGCCTTTCAAGAGAAGAAAAAGAAGAGAAACTAGAAACAGTT
Talapoin               AAAGAATTTGTCAATCCTAGCCTTTCAAGAGAAGAAAAAGAAGAGAAACTAGAAACAGTT
WolfsGuenon            AAAGAATTTGTCAATCCTAGCCTTTCAAGAGAAGAAAAAGAAGATAAACTAGAAACAGTT
Colobus                AAAGAATTTGTCAATCCTAGCCTTTCAAGAGAAGAAAAAGAAGAGAAACTAGAAACAGTT
Chimpanzee             AAAGAATTTGTCAATCCTAGCCTTCCAAGAGAAGAAAAAGAAGAGAAACTAGAAACAGTT
Bonobo                 AAAGAATTTGTCAATCCTAGCCTTCCAAGAGAAGAAAAAGAAGAGAAACTAGAAACAGTT
Gorilla                AAAGAATTTGTCAATCCTAGCCTTCCAAGAGAAGAAAAAGAAGAGAAACTAGAAACAGTT
Orangutan              AAAGAATTTGTCAATCCTAGCCTTCCAAGAGAAGAAAAAGAAGAGAAACTAGGAACAGTT
BorneoOrangutan        AAAGAATTTGTCAATCCTAGCCTTCCAAGAGAAGAAAAAGAAGAGAAACTAGGAACAGTT
PileatedGibbon         AAAGAATTTGTCAATCCTAGCCTTCCAAGAGAAGAAAAAGAAGAGAAACTAGAAACAGTT
AgileGibbon            AAAGAATTTGTCAATCCTAGCCTTCCAAGAGAAGAAAAAGAAGAGAAACTAGAAACAGTT
WHGibbon               AAAGAATTTGTCAATCCTAGCCTTCCAAGAGAAGAAAAAGAAGAGAAACTAGAAACAGTT
Siamang                AAAGAATTTGTCAATCCTAGCCTTCCAAGAGAAGAAAAAGAAGAGAAACTAGAAACAGTT
RCGibbon               AAAGAATTTGTCAATCCTAGCCTTCCAAGAGAAGAAAAAGAAGAGAAACTAGAAACAGTT
WCGibbon               AAAGAATTTGTCAATCCTAGCCTTCCAAGAGAAGAAAAAGAAGAGAAACTAGAAACAGTT
Marmoset               AAAGAATTTGTCAATCCTAGCCTTCCAAGAGAAGAAAAAGAAGAGAAACTAGAAACAGTT
SqMnky                 AAAGAATTTGTCAATCGTAGCCTTCCAAGAGAAGAAAAAGAAGAGAAACCAGAAACAGTT
TitiMonkey             AAAGAATTTGTCAATCCTAGTCTTCCAAGAGAAGAAAAAGAAGAGAAACTAGAAACAGTT
HowlerMk               AAAGAATTTGTCAATCCTAGCCTTCCAAGAGAACAAACAGAAGAGAAACTAGAAACAGTT
                       **************** *** *** ******** *** ****** **** ** *******

Human                  AAAGTGTCTAATAATGCTGAAGACCCCAAAGATCTCATGTTAAGTGGAGAAAGGGTTTTG
Rhesus                 AAAGTGTCTAATAATGCCAAAGACCCCAAAGATCTCATGTTAAGTGGAGAAAGGGTTTTA
CEMacaque              AAAGTGTCTAATAATGCCAAAGACCCCAAAGATCTCATGTTAAGTGGAGAAAGGGTTTTA
BMangabey              AAAGTGTCTAATAATGCCAAAGACCCCAAAGATCTCATGTTAAGTGGAGAAAGGGTTTTA
OliveBaboon            AAAGTGTCTAATAATGCCAAAGACCCCAAAGATCTCATGTTAAGTGGAGAAAGGGTTTTA
Talapoin               AAAGTGTCTAATAATGCCAAAGACCCCGAAGATCTCATGTTAAGTGGAGAAAGGGTTTTA
WolfsGuenon            AAAGTGTCTAATAATGCCAAAGACCCTGAAGATCTCATGTTAAGTGGAGAAAGCGTTTTA
Colobus                AAAGTATCTAATAATGCCAAAGCCCCCAAAGATCTCATGTTAAGTGGAGAAAGGGTTTTA
Chimpanzee             AAAGTGTCTAATAATGCCGAAGACCCCAAAGATCTCATGTTAAGTGGAGAAAGGGTTTTG
Bonobo                 AAAGTGTCTAATAATGCCGAAGACCCCAAAGATCTCATGTTAAGTGGAGAAAGGGTTTTG
Gorilla                AAAGTGTCTAATAATGCCGAAGACCCCAAAGATCTCATGTTAAGTGGAGAAAGGGTTTTG
Orangutan              AAAGTGTCTAATAATGCCAAAGACCCCAAAGATCTCATGTTAAGTGGAGAAAGGGTTTTG
BorneoOrangutan        AAAGTGTCTAATAATGCCAAAGACCCCAAAGATCTCATGTTAAGTGGAGAAAGGGTTTTG
PileatedGibbon         AAAGTGTCTAATAATGCCAAAGACCCCAAAGATCTCATGTTAAGTGGAGAAAGGGTTTTG
AgileGibbon            AAAGTGTCTAATAATGCCAAAGACCCCAAAGATCTCATGTTAAGTGGAGAAAGGGTTTTG
WHGibbon               AAAGTGTCTAATAATGCCAAAGACCCCAAAGATCTCATGTTAAGTGGAGAAAGGGTTTTG
Siamang                AAAGTGTCTAATAATGCCAAAGACCCCAAAGATCTCATGTTAAGTGGAGAAAGGGTTTTG
RCGibbon               AAAGTGTCTAATAATGCCAAAGACCCCAAAGATCTCATGTTAAGTGGAGAAAGGGTTTTG
WCGibbon               AAAGTGTCTAATAATGCCAAAGACCCCAAAGATCTCATGTTAAGTGGAGAAAGGGTTTTG
Marmoset               AAACTGTCTAATGATGCCAAAGACCCCAATGATCTCATGTTAAGTGGAGAAAGTGTTTTG
SqMnky                 AAACTGTCTAATGATGCCAGAGACCCCAAAGATCTCATGTTAAGTGGAGAAAGTGTTTTG
```

```
TitiMonkey            AAACTGTCTAATGATGCCAAAGACCCCAAAGATCTCATGTTAAGTGGAGAAAGTGTTTTG
HowlerMk              AAACTGTCTAATAATGCCAAAGACCCCAAAGATCTCATGTTAAGTGGAGAAAGTGTTTTG
                      *** * ****** ****   ** ***  * *********************** *****

Human                 CAAACTGAAAGATCTGTAGAGAGTAGCAGTATTTCATTGGTACCTGGTACTGATTATGGC
Rhesus                CAAACTGAAAGATCTGTAGAGAGTAGCAGTATTTCATTGGTACCTGATACCGATTATGGC
CEMacaque             CAAACTGAAAGATCTGTAGAGAGTAGCAGTATTTCATTGGTACCTGGTACCGATTATGGC
BMangabey             CAAACTGAAAGATCTGTAGAGAGTAGCAGTATTTCATTGGTACCTGGTACCGATTATGGC
OliveBaboon           CAAACTGAAAGATCTGTAGAGAGTAGCAGTATTTCATTGGTACCTGGTACCGATTATGGC
Talapoin              CAAACTGAAAGATCTGTAGAGAGTAGCAGTATTTCATTGGTACCTGGTACCGATTATGGC
WolfsGuenon           CAAACTGAAAGATCTGTAGAGAGTAGCAGTATTTCATTGGTACCTGGTACCGATTATGGC
Colobus               CAAACTGAAAGATCTGTAGAGAGTAGCAGTATTTCATTGGTACCTGGTACTGATTATGGC
Chimpanzee            CAAACTGAAAGATCTGTAGAGAGTAGCAGTATTTCATTGGTACCTGGTACTGATTATGGC
Bonobo                CAAACTGAAAGATCTGTAGAGAGTAGCAGTATTTCATTGGTACCTGGTACTGATTATGGC
Gorilla               CAAACTGAAAGATCTGTAGAGAGTAGCAGTATTTCATTGGTACCTGGTACTGATTATGGC
Orangutan             CAAACTGAAAGATCTGTAGAGAGTAGCAGTATTTCATTGGTACCTGGTACTGATTATGGC
BorneoOrangutan       CAAACTGAAAGATCTGTAGAGAGTAGCAGTATTTCATTGGTACCTGGTACTGATTATGGC
PileatedGibbon        CAAACTGAAAGATCTGTAGAGAGTAGCAGTATTTCATTGGTACCTGGTACTGATTATGGC
AgileGibbon           CAAACTGAAAGATCTGTAGAGAGTAGCAGTATTTCATTGGTACCTGGTACTGATTATGGC
WHGibbon              CAAACTGAAAGATCTGTAGAGAGTAGCAGTATTTCATTGGTACCTGGTACTGATTATGGC
Siamang               CAAACTGAAAGATCTGTAGAGAGTAGCAGTATTTCATTGGTACCTGGTACTGATTATGGC
RCGibbon              CAAACTGAAAGATCTGTAGAGAGTAGCAGTATTTCATTGGTACCTGGTACTGATTATGGC
WCGibbon              CAAACTGAAAGATCTGTAGAGAGTAGCAGTATTTCATTGGTACCTGGTACTGATTATGGC
Marmoset              CAAATTGAAAGATCTGTAGAGAGTAGCAGTATTTCATTGGTACCTGGTACTGATTATGGC
SqMnky                CAAATTGAAAGATCTGTAGAGAGTAGCAGTATTTCATTGGTACCTGGTACTGATTATGGC
TitiMonkey            CAAATTGAAAGATCTGTAGAGAGTAGCAGTATTTCATTGGTACCTGGTACTGATTATGGC
HowlerMk              CAAATTGAAAGATCTGTAGAGAGTAGCAGTATTTCGTTGATACCTGGTACTGATTATGGC
                      **** ****************************** *** ****** *** *********

Human                 ACTCAGGAAAGTATCTCGTTACTGGAAGTTAGCACTCTAGGGAAGGCAAAAACAGAACCA
Rhesus                ACTCAGGAAAGTATCTCATTACTGGAAGTTAGCACTCTAGGGAAGGCAAAAACAGAACGA
CEMacaque             ACTCAGGAAAGTATCTCATTACTGGAAGTTAGCACTCTAGGGAAGGCAAAAACAGAACGA
BMangabey             ACTCAGGAAAGTATCTCATTACTGGAAGTTAGCACTCTAGGGAAGGCAAAAACAGAACTA
OliveBaboon           ACTCAGGAAAGTATCTCATTACTGGAAGTTAGCACTCTAGGGAAGGCAAAAACAGAACTA
Talapoin              ACTCAGGAAAGTATCTCATTACTGGAAGTTAGCACTCTAGGGAAGGCAAAAACAGAACGA
WolfsGuenon           ACTCAGGAAAGTATCTCATTACTGGAAGTTAGCACTCTAGGGAAGGCAAAAACAGAACGA
Colobus               ACTCAGGAAAGTATCTCATTACTGGAAGTTAGCACTCTAGGGAAAGCAAAAACAGAACAA
Chimpanzee            ACTCAGGAAAGTATCTCGTTACTGGAAGTTAGCACTCTAGGGAAGGCAAAAACAGAACCA
Bonobo                ACTCAGGAAAGTATCTCGTTACTGGAAGTTAGCACTCTAGGGAAGGCAAAAACAGAACCA
Gorilla               ACTCAGGAAAGTATCTCGTTACTGGAAGTTAGCACTCTAGGGAAGGCAAAAACAGAACCA
Orangutan             ACTCAGGAAAGTATCTCGTTACTGGAAGTTAGCACTCTAGGGAAGGCAAAAACAGAACCA
BorneoOrangutan       ACTCAGGAAAGTATCTCGTTACTGGAAGTTAGCACTCTAGGGAAGGCAAAAACAGAACCA
PileatedGibbon        ACTCAGGAAAGTATCTCGTTACTGGAAGTTAGCACTCTAGGGAAGGCAAAAACAGAACCA
AgileGibbon           ACTCAGGAAAGTATCTCGTTACTGGAAGTTAGCACTCTAGGGAAGGCAAAAACAGAACCA
WHGibbon              ACTCAGGAAAGTATCTCGTTACTGGAAGTTAGCACTCTAGGGAAGGCAAAAACAGAACCA
Siamang               ACTCAGGAAAGTATCTCGTTACTGGAAGTTAGCACTCTAGGGAAGGCAAAAACAGAACCA
RCGibbon              ACTCAGGAAAGTATCTCGTTGCTGGAAGTTAGCACTCTAGGGAAGGCAAAAACAGAACCA
WCGibbon              ACTCAGGAAAGTATCTCGTTGCTGGAAGTTAGCACTCTAGGGAAGGCAAAAACAGAACCA
Marmoset              ACTCAGGAAAGTATCTCATTACTGGAAGTTAGCACTCTAGGGAAGGCAAAAACAGAACCA
SqMnky                ACTCAGGAAAGTATCTCATTACTGGAAGTTAGCACTCTAGGGAAGGCAAAAACAGAACCA
TitiMonkey            ACTCAGGAAAGTATCTCATTACTGGAAGTTAGCACTCCAGGGAAGGCAAAAACAGAACCA
HowlerMk              ACTCAGGAAAGTATCTCATTACTGGAAGTTAGCACTCTGGGGAAGGCAAAAACAGAACCA
                      ***************** ** ****************  ***** ************* *

Human                 AATAAATGTGTGAGTCAGTGTGCAGCATTTGAAAACCCCAAGGGACTAATTCATGGTTGT
Rhesus                AATAAATGTATGAGTCAGTGTGCAGCATTTGAAAACCCCAAGGAACTAATTCATGGTTGT
CEMacaque             AATAAATGTATGAGTCAGTGTGCAGCATTTGAAAACCCCAAGGAACTAATTCATGGTGGT
BMangabey             AATAAATGTATGAGTCAGTGTGCAGCATTTGAAAACTCCAAGGAACTAATTCATGGTTGT
OliveBaboon           AATAAATGTATGAGTCAGTGTGCAGCATTTGAAAACTCCAAGGAACTAATTCATGGTTGT
Talapoin              AATAAATGTATGAGTCAGTGTGCAGCATTTGAAAACCCCAAGGAACTAATTCATGGTTGT
WolfsGuenon           AATAAATGTATGAGTCAGTGTGCAGCATTTGAAAACCCCAAGAAACTAATTCATGGTTGT
Colobus               AATAAATGTGTGAGTCAGTGTGCAGCATTTGAAAACCCCAAGGAACTAATTCATGGTTGT
Chimpanzee            AATAAATGTGTGAGTCAGTGTGCAGCATTTGAAAACCCCAAGGGACTAATTCATGGTTGT
Bonobo                AATAAATGTGTGAGTCAGTGTGCAGCATTTGAAAACCCCAAGGGACTAATTCATGGTTGT
Gorilla               CATAAATGTGTGAGTCAGTGTGCAGCATTTGAAAACCCCAAGGGACTAATTCATGGTTGT
Orangutan             AATAAATGTGTGAGTCAGTGTGCAGCATTTGAAAACCCCAAGGAACTAATTCATGGTTGT
BorneoOrangutan       AATAAATGTGTGAGTCAGTGTGCAGCATTTGAAAACCCCAAGGAACTAATTCATGGTTGT
PileatedGibbon        AATAAATGTGTGAGTCAGTGTGCAGCATTTGAAAACCCCAAGGAACTAATTCATGGTTGT
AgileGibbon           AATAAATGTGTGAGTCAGTGTGCAGCATTTGAAAACCCCAAGGAACTAATTCATGGTTGT
WHGibbon              AATAAATGTGTGAGTCAGTGTGCAGCATTTGAAAACCCCAAGGAACTAATTCATGGTTGT
Siamang               AATAAATGTGTGAGTCAGTGTGCAGCATTTGAAAACCCCAAGGAACTAATTCATGGTTGT
RCGibbon              AATAAATGTGTGAGTCAGTGTGCAGCATTTGAAAACCCCAAGGAACTAATTCATGGTTGT
WCGibbon              AATAAATGTGTGAGTCAGTGTGCAGCATTTGAAAACCCCAAGGAACTAATTCATGGTTGT
```

```
Marmoset            AATAAATGTGTGAGTCAGTGTGCAGCATTTGAAAACCCCAAGGAACTAATTCATGGTGGT
SqMnky              AATAAATGTGTGAGCCAGTGTGCAGCATTTGAAAACCCCAAGGAACTAATTCATGGTTGT
TitiMonkey          AAGAAATGTGTGAGTCAATGTGGAGCATTTGAAAACCCCAAGGAACTAATTCGTGGTTGT
HowlerMk            AATAAATGTGTGAGTCAGTGTGCAGCATTTGAAAACCCCAAGGAACTAATTCATGGTTGT
                     * ****** **** ** **** ************* *****  ******** **** **

Human               TCCAAAGATAATAGAAATGACACAGAAGGCTTTAAGTATCCATTGGGACATGAAGTTAAC
Rhesus              TCTGAAGATACTAGAAATGACACAGAAGGCTTTAAGTATCCATTGGGAAGTGAAGTTAAC
CEMacaque           TCTGAAGATACTAGAAATGACACAGAAGGCTTTAAGTATCCATTGGGAAGTGAAGTTAAC
BMangabey           TCTGAAGATACTAGAAATGACACAGAAGGCTTTAAGTATCCATTGGGACGTGAAGTTAAC
OliveBaboon         TCTGAAGATACTAGAAATGACACAGAAGGCTTTAAGTATCCATTGGGACGTGAAGTTAAC
Talapoin            TCTGAAGATACTAGAAATGACACAGAAGGCTTTAAGTATCCATTGGGACATGAAGTTAAC
WolfsGuenon         TCTGAAGATACTAGAAATGACACAGAAGGCTTTAAGTATCCATTGGGACATGAAGTTAAC
Colobus             TCTGAAGATACTAGAAATGACACAGAAGGCTTTAAGTATCCATTGGGAGGTGATGTTAAC
Chimpanzee          TCCAAAGATACTAGAAATGACACAGAAGGCTTTAAGTATCCATTGGGACATGAAGTTAAC
Bonobo              TCCAAAGATACTAGAAATGACACAGAAGGCTTTAAGTATCCATTGGGACATGAAGTTAAC
Gorilla             TCCAAAGATACTAGAAATGACACAGAAGGCTTTAAGTATCCATTGGGACATGAAGTTAAC
Orangutan           TTCAAAGATACTAGAAATGACACAGAAGGGTTTAAGTATCCATTGGGACATGAAGTTAAC
BorneoOrangutan     TTCAAAGATACTAGAAATGACACAGAAGGCTTTAAGTATCCATTGGGACATGAAGTTAAC
PileatedGibbon      TCCAAAGATACTAGAAATGACACAGAAGGCTTTAAGTATCCATTGGGACATGAAGTTAAC
AgileGibbon         TCCAAAGATACTAGAAATGACACAGAAGGCTTTAAGTATCCATTGGGACATGAAGTTAAC
WHGibbon            TCCAAAGATACTAGAAATGACACAGAAGGCTTTAAGTATCCATTGGGACATGAAGTTAAC
Siamang             TCCAAAGATACTAGAAATGACACAGAAGGCTTTAAGTACCCATTGGGACATGAAGTTAAC
RCGibbon            TCCAAAGATACTAGAAATGACACAGAAGGCTTTAAGTATCCATTGGGACATGAAGTTAAC
WCGibbon            TCCAAAGATACTAGAAATGACACAGAAGGCTTTAAGTATCCATTGGGACATGAAGTTAAC
Marmoset            TCTAAAGATACTAGAAATGGCACAGAAGGCTTTAAGTATCCATTGGGACCTGAAGTTAAC
SqMnky              TCTAAAGATACTAGAAATGGCACAGAAGGCTTTAAGTATCCGTTGGGACCTGAAGTTAAC
TitiMonkey          TCTAAAGATACTAGAAATGGCACAGAAGGCTCTAAGTATCCGTTGGGACCTGAAGTTAAC
HowlerMk            TCTAAAGATACTAGAAATGGCACAGAAGGCTTGAAGTATCCATTGGGACCTGAAGTTAAC
                    *   ****** ******** ********* *  ***** ** ******  *** ******

Human               CACAGTCGGGAAACAAGCATAGAAATGGAAGAAAGTGAACTTGATGCTCAGTATTTGCAG
Rhesus              CACAGTCAGGAAACAAGCATAGAAATAGAAGAAAGTGAACTTGATACTCAGTATTTGCAG
CEMacaque           CACAGTCAGGAAACAAGCATAGAAATAGAAGAAAGTGAACTTGATACTCAGTATTTGCAG
BMangabey           CACAGTCAGGAAACAAGCATAGAAATAGAAGAAAGTGAACTTGATACTCAGTATTTGCAG
OliveBaboon         CACAGTCAGGAAACAAGCATAGAAATAGAAGAAAGTGAACTTGATACTCAGTATTTGCAG
Talapoin            CACAGTCAGGAAACAAGCATAGAAATAGAAGAAAGTGAACTTGATACTCAGTATTTGCAG
WolfsGuenon         CACAGTCAGGAAACAAGCATAGAAATAGAAGAAAGTGAACTTGATACTCAGTATTTGCAG
Colobus             CACAGTCAGGAAACAAGCATAGAAATAGAAGAAAGTGAACTTGATACTCAGTATTTGCAG
Chimpanzee          CACAGTCGGGAAACAAGCATAGAAATGGAAGAAAGTGAACTTGATGCTCAGTATTTGCAG
Bonobo              CACAGTCGGGAAACAAGCATAGAAATGGAAGAAAGTGAACTTGATGCTCAGTATTTGCAG
Gorilla             CACAGTCGGGAAACAAGCATAGAAATGGAAGAAAGTGAACTTGATGCTCAGTATTTGCAG
Orangutan           CACAGTCAGGAAACAAGCATAGAAATGGAAGAAAGTGAACTTGATACTCAGTATTTGCAG
BorneoOrangutan     CACAGTCAGGAAACAAGCATAGAAATGGAAGAAAGTGAACTTGATACTCAGTATTTGCAG
PileatedGibbon      CACAGTCAGGAAACAAGCATAGAAATGGAAGAAAGTGAACTTGATACTCAGTATTTGCAG
AgileGibbon         CACAGTCAGGAAACAAGCATAGAAATGGAAGAAAGTGAACTTGATACTCAGTATTTGCAG
WHGibbon            CACAGTCAGGAAACAAGCATAGAAATGGAAGAAAGTGAACTTGATACTCAGTATTTGCAG
Siamang             CACAGTCAGGAAACAAGCATAGAAATGGAAGAAAGTGAACTTGATACTCAGTATTTGCAG
RCGibbon            CACAGTCAGGAAACAAGCATAGAAATGGAAGAAAGTGAACTTGATACTCAGTATTTGCAG
WCGibbon            CACAGTCAGGAAACAAGCATAGAAATGGAAGAAAGTGAACTTGATACTCAGTATTTGCAG
Marmoset            TACAGTCAGGAAACAAGCATAGATATGGAAGAAAGTGAACTTGATACTCAATATTTGCAG
SqMnky              TACAGTCAGGAAACAAGCATGGATATGGAAGAAAGTGAACTTGATACTCAATATTTGCAG
TitiMonkey          TGCAGTCAGAAAACAAACATAGATATGGAAGAAAGTGAACTTGATACTCAGTATTTGCAG
HowlerMk            TACAGTCAGGAAACAAGCATAGATATGAGAGAAAGTGAACTTGATACTCAATATTTGCAG
                      ***** * ****** *** ** **   **************** **** *********

Human               AATACATTCAAGGTTTCAAAGCGCCAGTCATTTGCTCCGTTTTCAAATCCAGGAAATGCA
Rhesus              AATACATTCAAGGTTTCAAAGCGCCAGTCCTTTGCTCTGTTTTCAAATCCAGGAAATCCA
CEMacaque           AATACATTCAAGGTTTCAAAGCGCCAGTCCTTTGCTCTGTTTTCAAATCCAGGAAATCCA
BMangabey           AATACATTCAAGGTTTCAAAGCGCCAGTCCTTTGCTCTGTTTTCAAATCCAGGAAATCCA
OliveBaboon         AATACATTCAAGGTTTCAAAGCGCCAGTCCTTTGCTCTGTTTTCAAATCCAGGAAATCCA
Talapoin            AATACATTCAAGGTTTCAAAGCGCCAGTCCTTTGCTCTGTTTTCAAATCCAGGAAATCCA
WolfsGuenon         AATACATTCAAGGTTTCAAAGCGCCAGTCCTTTGCTCTGTTTTCAAATCCAGGAAATCCA
Colobus             AATACATTCAAGGTTTCAAAGCGCCAGTCCTTTGCTCTGTTTTCAAATCCAGGAAATCCA
Chimpanzee          AATACATTCAAGGTTTCAAAGCGCCAGTCATTTGCTCTGTTTTCAAATCCAGGAAATCCA
Bonobo              AATACATTCAAGGTTTCAAAGCGCCAGTCATTTGCTCTGTTTTCAAATCCAGGAAATCCA
Gorilla             AATACATTCAAGGTTTCAAAGCGCCAGTCATTTGCTCTGTTTTCAAATCCAGGAAATCCA
Orangutan           AATACATTCAAGGTTTCAAAGCGCCAGTCATTTGCTCTGTTTTCAAATCCAGGAAATCCA
BorneoOrangutan     AATACATTCAAGGTTTCAAAGCGCCAGTCATTTGCTCTGTTTTCAAATCCAGGAAATCCA
PileatedGibbon      AATACATTCAAGGTTTCAAAGCGCCAGTCATTTGCTCTGTTTTCAAATCCAGGAAATCCA
AgileGibbon         AATACATTCAAGGTTTCAAAGCGCCAGTCATTTGCTCTGTTTTCAAATCCAGGAAATCCA
WHGibbon            AATACATTCAAGGTTTCAAAGCGCCAGTCATTTGCTCTGTTTTCAAATCCAGGAAATCCA
Siamang             AATACATTCAAGGTTTCAAAGCGCCAGTCATTTGCTCTGTTTTCAAATCCAGGAAATCCA
```

```
RCGibbon              AATACATTCAAGGTTTCAAAGCGCCAGTCATTTGCTCTGTTTTCAAATCCAGGAAATCCA
WCGibbon              AATACATTCAAGGTTTCAAAGCGCCAGTCATTTGCTCTGTTTTCAAATCCAGGAAATCCA
Marmoset              AATACATTCAAGGTTTCAAAGCGCCAGTCATTTGCTCTGTTTTCAAATCCAGGAAATCCA
SqMnky                AATACATTCAAGGTTTCAAAGCGCCAGTCATTTGCTCTGTTTTCAAATCCAGGAAATCCA
TitiMonkey            AATACATTCAAGGTTTCAAAGCGCCAGTCATTTGCTCTGTTTTCAAATCCAGGAAATTCA
HowlerMk              AATACATTCAAGGTTTCAAAGCGCCAGTCATTTGCTCTGTTTTCAAATCCAGGAAATCCA
                      ***************************** ******* ******************* **

Human                 GAAGAGGAATGTGCAACATTCTCTGCCCACTCTGGGTCCTTAAAGAAACAAAGTCCAAAA
Rhesus                GAAGAGGAATGTGCAACATTCTCTGCCCACTCTAGGTCCTTAAAGAAACAAAGTCCAAAA
CEMacaque             GAAGAGGAATGTGCAACATTCTCTGCCCACTCTAGGTCCTTAAAGAAACAAAGTCCAAAA
BMangabey             GAAGAGGAATGTGCAACATTCTCTGCCCACTCTAGGTCCTTAAAGAAACAAAGTCCAAAA
OliveBaboon           GAAGAGGAATGTGCAACATTCTCTGCCCACTCTAGGTCCTTAAAGAAACAAAGTCCAAAA
Talapoin              GAAGAGGAATGTGTAACATTCTCTGCCCACTCTAGGTCCTTAAAGAAACAAAGTCCAAAA
WolfsGuenon           GAAGAGGAATGTGTAACATTCTCTGCCCACTCTAGGTCCTTAAAGAAACAAAGTCTAAAA
Colobus               GAAGAGGAATGTGCAACATTCTCTGCCCACTCTAAGTCCTTAAAGAAACAAAGTCCAAAA
Chimpanzee            GAAGAGGAATGTGCAACATTCTCTGCCCACTGTAGGTCCTTAAAGAAACAAAGTCCAAAA
Bonobo                GAAGAGGAATGTGCAACATTCTCTGCCCACTGTAGGTCCTTAAAGAAACAAAGTCCAAAA
Gorilla               GAAGAGGAATGTGCAACATTCTCTGCCCACTCTAGGTCCTTAAAGAAACAAAGTCCAAAA
Orangutan             GAAGAGGAATGTGCAACATTCTCTGCCCACTCTAGGTCCTTAAAGAAACAAAGTCCAAAA
BorneoOrangutan       GAAGAGGAATGTGCAACATTCTCTGCCCACTCTAGGTCCTTAAAGAAACAAAGTCCAAAA
PileatedGibbon        GAAGAGGAATGTGCAACATTCTCTGCCCAGTCTAGGTCCTTAAAGAAACGAAGTCCAAAA
AgileGibbon           GAAGAGGAATGTGCAACATTCTCTGCCCAGTCTAGGTCCTTAAAGAAACGAAGTCCAAAA
WHGibbon              GAAGAGGAATGTGCAACATTCTCTGCCCAGTCTAGGTCCTTAAAGAAACGAAGTCCAAAA
Siamang               GAAGAGGAATGTGCAACATTCTCTGCCCAGTCTAGGTCCTTAAAGAAACGAAGTCCAAAA
RCGibbon              GAAGAGGAATATGCAACATTCTCTGCCCAGTCTAGGTCCTTAAAGAAACGAAATCCAAAA
WCGibbon              GAAGAGGAATATGCAACATTCTCTGCCCAGTCTAGGTCCTTAAAGAAACGAAATCCAAAA
Marmoset              GAAAAGGAATGTGCAACATTCTCTGCCCACTCTGGACCCTTAAAGAAACAAAGTCCAAAG
SqMnky                GAAAAGGAATGTGCAGCATTCTCTGCCCACTCCAGGTCCTTAAAGAAACAAAGTCCAAAG
TitiMonkey            GAAAAGGAATGTGCAACATTCTCTGCCCACTCTGGGTCCTTAAAGAAACAAAGTCCAAAG
HowlerMk              GAAAAGGAATGTGCAACATTCTCTGCCTGCTCTGGGTCCTTAAAGAAACAAAGTCCAAAG
                      *** ****** ** * ***********   *      ************ ** ** ***

Human                 GTCACTTTTGAATGTGAACAAAAGGAAGAAAATCAAGGAAAGAATGAGTCTAATATCAAG
Rhesus                GTTACTTCTGAATGTGAACAAAAGGAAGAAAATCAAGGAAAGAAAGAGTCTAATATCAAG
CEMacaque             GTTACTTCTGAATGTGAACAAAAGGAAGAAAATCAAGGAAAGAAAGAGTCTAATATCAAG
BMangabey             GTTACTTCTGAATGTGAACAAAAGGAAGAAAATCAAGGAAAGAAAGAGTCTAATATCAAG
OliveBaboon           GTTACTTCTGAATGTGAACAAAAGGAAGAAAATCAAGGAAAGAAAGAGTCTAATATCAAG
Talapoin              GTTACTTCTGAATGTGAACAAAAGGAAGAAAATCAAGGAAAGAAAGAGTCTAATATCAAG
WolfsGuenon           GTTACTTCTGAATGTGAACAAAAGGAAGAAAATCAAGGAAAGAAAGAGTCTAATATCAAG
Colobus               GTTACTTCTGAATGTGAACAAAAGGAAGAAAATCAAGGAAAGAAAGAGTCTAATATCAAG
Chimpanzee            GTCACTTTTGAACGTGAACAAAAGGAACAAAATCAAGGAAAGAATGAGTCTAATATCAAG
Bonobo                GTCACTTTTGAACGTGAACAAAAGGAACAAAATCAAGGAAAGAATGAGTCTAATATCAAG
Gorilla               GTCACTTTTGAATGTGAACAAAAGGAAGAAAATCAAGGAAAGAATGAGTCTAATATCAAG
Orangutan             GTCACTTTTGAATGTGAACAAAAGGAAGAAAATCAAGGAAAGAATGAGTCTAATATCAAG
BorneoOrangutan       GTCACTTTTGAATGTGAACAAAAGGAAGAAAATCAAGGAAAGAATGAGTCTAATATCAAG
PileatedGibbon        GTCACTTTTGAATGTGAACAAAAGGAAGAAAATCAAGGAAAGAATAAGTCTAATATCAAG
AgileGibbon           GTCACTTTTGAATGTGAACAAAAGGAAGAAAATCAAGGAAAGAATAAGTCTAATATCAAG
WHGibbon              GTCACTTTTGAATGTGAACAAAAGGAAGAAAATCAAGGAAAGAATAAGTCTAATATCAAG
Siamang               GTCACTTTTGAATGTGAACAAAAGGAAGAAAATCAAGGAAAGAATAAGTCTAATATCAAG
RCGibbon              GTCACTTTTGAATGTGAACAAAAGGAAGAAAATCAAGGAAAGAATAAGTCTAATATCAAG
WCGibbon              GTCACTTTTGAATGTGAACAAAAGGAAGAAAATCAAGGAAAGAATAAGTCTAATATCAAG
Marmoset              GTCACTCTTGAATGTGAACAAAAGGAAGAAAATCAAGGAGAGAAAGAGTCTAATAGCGAG
SqMnky                GTCACTCTTGAATGTGAACAAAAGGAAGAAAATCAAGGAGAGAAAGAGTCTAATATCGAG
TitiMonkey            GTCACTCTTGAATGTGAACAAAAGGAAGAAAATCAAGGAGAGAAAGAGTCTAATATCGAG
HowlerMk              GTCACTCCTGAATGTGAACAAAAGGAAGAAAATCAAGGAGAGAAAGAGTCTAATATCGAG
                      ** ***  **** ************** *********** ****  ********* * **

Human                 CCTGTACAGACAGTTAATATCACTGCAGGCTTTCCTGTGGTTGGTCAGAAAGATAAGCCA
Rhesus                CCTGTACAGACAGTTAATATCACTGCAGGCTTTTCTGTGGTTTGTCAGAAAGATAAACCA
CEMacaque             CCTGTACAGACAGTTAATATCACTGCAGGCTTTTCTGTGGTTTGTCAGAAAGATAAGCCA
BMangabey             CCTGTACAGACAGTTAATATCACTGCAGGCTTTTCTGTGGTTTGTCAGAAAGATAAGCCA
OliveBaboon           CCTGTACAGACAGTTAATATCACTGCAGGCTTTTCTGTGGTTTGTCAGAAAGATAAGCCA
Talapoin              CCTGTACAGACAGTTAATATCACTGCAGGCTTTTCTGTGGTTTGTCAGAAAGATAAGCCA
WolfsGuenon           CCTGTACAGACAGTTAATATCACTGCAGGCTTTTCTGTGGTTTGTCAGAAAGATAAGCCA
Colobus               CCTGTACAGACAGTTAATATCACTGCAGGCTTTCCTGTGGTTTGTCAGAAAGATAAGCCA
Chimpanzee            CCTGTACAGACAGTTAATACCACTGCAGGCTTTCCTGTGGTTTGTCAGAAAGATAAGCCA
Bonobo                CCTGTACAGACAGTTAATATCACTGCAGGCTTTCCTGTGGTTTGTCAGAAAGATAAGCCA
Gorilla               CCTGTACAGACAGTTAATATCACTGCAGGCTTTCCTGTGGTTTGTCAGAAAGATAAGCCA
Orangutan             CCTGTACAGACAGCTAATATCACTGCAGGCTTTCCTGTGGTTTGTCAGAAAGATAAGCCA
BorneoOrangutan       CCTGTACAGACAGCTAATATCACTGCAGGCTTTCCTGTGGTTTGTCAGAAAGATAAGCCA
PileatedGibbon        CCTGTACAGACAGTTAATATCTCTGCAGGCTTTCCTGTGGTTTGTCAGAAAGATAAGCCA
AgileGibbon           CCTGTACAGACAGTTAATATCTCTGCAGGCTTTCCTGTGGTTTGTCAGAAAGATAAGCCA
```

```
WHGibbon               CCTGTACAGACAGTTAATATCTCTGCAGGCTTTCCTGTGGTTTGTCAGAAAGATAAGCCA
Siamang                CCTGTACAGACAGTTAATATCTCTGCAGGCTTTCCTGTGGTTTGTCAGAAAGATAAGCCA
RCGibbon               CCTGTACAGACAATTAATATCTCTGCAGGCTTTCCTGTGGTTTGTCAGAAAGATAAGCCA
WCGibbon               CCTGTACAGACAGTTAATATCTCTGCAGGCTTTCCTGTGGTTTGTCAGAAAGATAAGCCA
Marmoset               CCTGTAGAGACAGTTAACATCACTGCAGGCTTTCCTATGGTTTGTCAGAAAGATAAGCCA
SqMnky                 CCTGTAGAGACAGTTAATATCACTGCAGGCATTCCTATGGTTTGTCAGAAAGATAAGCCA
TitiMonkey             CCTGTAGAGACAGTTAATATCACTGCAGCCTTTCCTATGGTTTGTCAGAAAGACAAGCCA
HowlerMk               CTTGTAGAGACAGTTAATACCACTGCAGGCTTTCCTATGGTTTGTCAGAAAGATAAGCCA
                       * **** *****  *** * * ****** * ** ** ***** ********** ** ***

Human                  GTTGATAATGCCAAATGTAGTATCAAAGGAGGCTCTAGGTTTTGTCTATCATCTCAGTTC
Rhesus                 GTTGATAATGCCAAATGTAGTATCAAAGGAGGCTCTAGGTTTTGTCTATCATCTCAGTTC
CEMacaque              GTTGATAATGCCAAATGTAGTATCAAAGGAGGCTCTAGGTTTTGTCTATCATCTCAGTTC
BMangabey              GTTGATAATGCCAAATGTAGTATCAAAGGAGGCTCTAGGTTTTGTCTATCATCTCAGTTC
OliveBaboon            GTTGATAATGCCAAATGTAGTATCAAAGGAGGCTCTAGGTTTTGTCTATCATCTCAGTTC
Talapoin               GTTGATAATGCCAAATGTAGTATCAAAGGAGGCTCTAGGTTTTGTCTATCATCTCAGTTC
WolfsGuenon            GTTGATAATGCCAAATGTAGTATCAAAGGAGGCTCTAGGTTTTGTCTATCATCTCAGTTC
Colobus                GTTGATAATGCCAAATGTAGTATCAAAGGAGGCTCTAGGTTTTGTCTATCATCTCAGTTC
Chimpanzee             GTTGATTATGCCAAATGTAGTATCAAAGGAGGCTCTAGGTTTTGTCTATCATCTCAGTTC
Bonobo                 GTTGATTATGCCAAATGTAGTATCAAAGGAGGCTCTAGGTTTTGTCTATCATCTCAGTTC
Gorilla                GTTGATTATGCCAAATGTAGTATCAAAGGAGGCTCTAGGTTTTGTCTATCATCTCAGTTC
Orangutan              GTTGATTATGCCAAATGTAGTATCAAAGGAGGCTCTAGGTTTTGTCTATCATCTCAGTTC
BorneoOrangutan        GTTGATTATGCCAAATGTAGTATCAAAGGAGGCTCTAGGTTTTGTCTATCATCTCAGTTC
PileatedGibbon         GTTGATTATGCCAAATGTAGTATCAAAGGAGGCTCTAGGTTTTGTCTATCATCTCAGTTC
AgileGibbon            GTTGATTATGCCAAATGTAGTATCAAAGGAGGCTCTAGGTTTTGTCTATCATCTCAGTTC
WHGibbon               GTTGATTATGCCAAATGTAGTATCAAAGGAGGCTCTAGGTTTTGTCTATCATCTCAGTTC
Siamang                GTTGATTATGCCAAATGTAGTATCAAAGGAGGCTCTAGGTTTTGTCTATCATCTCAGTTC
RCGibbon               GTTGATTATGCCAAATGTAGTATCAAAGGAGGCTCTAGGTTTTGTCTATCATCTCAGTTC
WCGibbon               GTTGATTATGCCAAATGTAGTATCAAAGGAGGCTCTAGGTTTTGTCTATCATCTCAGTTC
Marmoset               GTTGATTATGCCAAAGGT---ATTGAAGGAGGCTCTGGGCTTTGTCTATCATCTCAGTTC
SqMnky                 GTTGACTATGCCAAATGT---ATCGAAGGAGGCTCTAGGCTTTGTCTATCATCTCAGTTC
TitiMonkey             GTTGATTATGCCAAATGT---ATCGAAGGAGGCTCTAGGCTTTGTCTGTCATATCAGTTC
HowlerMk               GTTGATTATGCCAAATGT---ATCGAAGGAGGCTCTAGGCTTTGTCTATCATCTCAGTTC
                       *****  ******** **   **   *********** ** ******* **** *******

Human                  AGAGGCAACGAAACTGGACTCATTACTCCAAATAAACATGGACTTTTACAAAACCCATAT
Rhesus                 AGAGGCAACGAAACTGGACTCATTACTCCAAATAAACATGGACTTTTACAAAACCCATAC
CEMacaque              AGAGGCAACGAAACTGGACTCATTACTCCAAATAAACATGGACTTTTACAAAACCCATAC
BMangabey              AGAGGCAACGAAACTGGACTCATTACTCCAAATAAACATGGACTTTTACAAAACCCATAC
OliveBaboon            AGAGGCAACGAAACTGGACTCATTACTCCAAATAAACATGGACTTTTACAAAACCCATAC
Talapoin               AGAGGCAACGAAACTGGACTCATTACTCCAAATAAACATGGACTTTTACAAAACCCATAC
WolfsGuenon            AGAGGCAACGAAACTGGACTCATTACTCCAAATAAACATGGACTTTTACAAAACCCATAC
Colobus                AGAGGCAACGAAACTGGACTCATTACTCCAAATAAACATGGACTTTTACAAAACCCATAC
Chimpanzee             AGAGGCAACGAAACTGGACTCATTACTCCAAATAAACATGGACTTTTACAAAACCCATAT
Bonobo                 AGAGGCAACGAAACTGGACTCATTACTCCAAATAAACATGGACTTTTACAAAACCCATAT
Gorilla                AGAGGCAACGAAACTGGACTCATTACTCCAAATAAACATGGACTTTTACAAAACCCATAT
Orangutan              AGAGGCAACGAAACTGGACTCATTACTCCAAATAAACATGGACTTTCACAAAACCCATAT
BorneoOrangutan        AGAGGCAACGAAACTGGACTCATTACTCCAAATAAACATGGACTTTCACAAAACCCATAT
PileatedGibbon         AGAGGCAACAAAACTGGACTCATTACTCCAAATAAACATGGACTTTTACAAAACCCATAT
AgileGibbon            AGAGGCAACAAAACTGGACTCATTACTCCAAATAAACATGGACTTTTACAAAACCCATAT
WHGibbon               AGAGGCAACAAAACTGGACTCATTACTCCAAATAAACATGGACTTTTACAAAACCCATAT
Siamang                AGAGGCAACAAAACTGGACTCATTACTCCAAATAAACATGGACTTTTACAAAACCCATAT
RCGibbon               AGAGGCAACAAAACTGGACTCATTACTCCAAATAAACATGGACTTTTACAAAACCCATAT
WCGibbon               AGAGGCAACAAAACTGGACTCATTACTCCAAATAAACATGGACTTTTACAAAACCCATAT
Marmoset               AGAGGCAATGAAACTGGACTCATTATTCCAAATAAACATGGACTTTTACAAAACCCATAT
SqMnky                 AGAGGCAATGAAACTGAACTCATTACTCCAAATAAACATGGACTTTTACAAAACTCGTAT
TitiMonkey             AGAGGCAATGAAACTGGACTCATTATTCCAAATAAACATGGACTTTTACAAAACCCGTGT
HowlerMk               AGAGGCAACGAAACTGGACTCATTATTCCAAATAAACATGGACTTTTACAGAACCCATAT
                       ********  ****** ******** ******************** *** *** * *

Human                  CGTATACCACCACTTTTTCCCATCAAGTCATTTGTTAAAACTAAATGTAAGAAAAATCTG
Rhesus                 CATATACCACCACTTTTTCCTGTCAAGTCATTTGTTAAAACTAAATGTAACAAAAACCTG
CEMacaque              CATATACCACCACTTTTTCCTGTCAAGTCATTTGTTAAAACTAAATGTAACAAAAACCTG
BMangabey              CATATACCACCACTTTTTCCTGTCAAGTCATTTGTTAAAACTAAATGTAACAAAAACCTG
OliveBaboon            CATATACCACCACTTTTTCCTGTCAAGTCATTTGTTAAAACTAAATGTAACAAAAACCTG
Talapoin               CATATACCACCACTTTTTCCTGTCAAGTCATTTGTTAAAACTAAATGTAAGAAAAACCTG
WolfsGuenon            CATATACCACCACTTTTTCCTGTCAAGTCATTTGTTAAAACTAAATGTAAGAAAAACTTG
Colobus                CATATACCACCGCTTTTTCCTGTCAGGTCATTTGTTAAAACTAAATGTAAGAAAAACCTG
Chimpanzee             CATATACCACCACTTTTTCCCATCAAGTCATTTGTTAAAACTAAATGTAAGAAAAACCTG
Bonobo                 CATATACCACCACTTTTTCCCATCAAGTCATTTGTTAAAACTAAATGTAAGAAAAACCTG
Gorilla                CATATACCACCACTTTTTCCCATCAAGTCATTTGTTAAAACTAAATGTAAGAAAAACCTG
Orangutan              CATATACCACCACTTTTTCCCATCAAGTCATTTGTTAAAACTAAATGTAAGAAAAACCTG
BorneoOrangutan        CATATACCACCACTTTTTCCCATCAAGTCATTTGTTAAAACTAAATGTAAGAAAAACCTG
```

```
PileatedGibbon        CATATACCACCACTTTTTCCCATCAAGTCAATTGTTAAAACTAAATGTAAGAAAAACCTG
AgileGibbon           CATATACCACCACTTTTTCCCATCAAGTCAATTGTTAAAACTAAATGTAAGAAAAACCTG
WHGibbon              CATATACCACCACTTTTTCCCATCAAGTCAATTGTTAAAACTAAATGTAAGAAAAACCTG
Siamang               CATATACCACCACTTTTTCCCATCAAGTCAATTGTTAAAACTAAATGTAAGAAAAACCTG
RCGibbon              CATATACCACCACTTTTTCCCATCAAGTCAATTGTTAAAACTAAATGTAAGAAAAACCTG
WCGibbon              CATATACCACCACTTTTTCCCATCAAGTCAATTGTTAAAACTAAATGTAAGAAAAACCTG
Marmoset              CATATACCACCACTTATTCCCACCAGGTCATTCGTTAAAACTAAATATAAGAAAAACCTG
SqMnky                CATATACCACCACTTATTCCCACCAGGTCATTTATTGAAACTAAATGTAAGAAAAACCTG
TitiMonkey            CATATACCACCACTTATTCCCACCAGGTCATTTGTTAAAACTAAATGTAAGAAAAACCTG
HowlerMk              CATATGTCACCGCTTATTCCCACCAGGTCATTTGTTAAAACTAAATGTAAGAAAAACCTG
                      * ***  **** *** ****   ** **** *  ** ********* *** *****  **

Human                 CTAGAGGAAAACTTTGAGGAACATTCAATGTCACCTGAAAGAGAAATGGGAAATGAGAAC
Rhesus                CTAGAGGAAAACTCTGAGGAACATTCAGTGTCACCTGAAAGAGCAGTGGGAAACGAGAAC
CEMacaque             CTAGAGGAAAACTCTGAGGAACATTCAGTGTCACCTGAAAGAGCAGTGGGAAACGAGAAC
BMangabey             CTAGAGGAAAACTCTGAGGAACATTCAGTGTCACCTGAAAGAGCAGTGGGAAACGAGAAC
OliveBaboon           CTAGAGGAAAACTCTGAGGAACATTCAGTGTCACCTGAAAGAGCAGTGGGAAACGAGAAC
Talapoin              CTAGAGGAAAACTCTGAGGAACATTCAGTGTCACCCGAAAGAGCAGTGGGAAACGAGAAC
WolfsGuenon           CTAGAGGAAAACTCTGAGGAACATTCAGTGTCACCCGAAAGAGCAGTGGGAAACGAGAAC
Colobus               CTAGAGGAAAACTCTGAGGAACATTCAGTGTCACCTGAAAGAGCAGTAGGAAACGAGAAC
Chimpanzee            CTAGAGGAAAACTTTGAGGAACATTCAATGTCACCTGAAAGAGAAATGGGAAATGAGAAC
Bonobo                CTAGAGGAAAACTTTGAGGAACATTCAATGTCACCTGAAAGAGAAATGGGAAATGAGAAC
Gorilla               CTAGAGGAAAACTTTGAGGAACATTCAATGTCACCTGAAAGAGAAATGGGAAATGAGAAC
Orangutan             CTAGAGGAAAACTCTGAGGAACATTCAATGTCACCTGAAAGAGAAATGGGAAACGAGAAC
BorneoOrangutan       CTAGAGGAAAACTCTGAGGAACATTCAATGTCACCTGAAAGAGAAATGGGAAACGAGAAC
PileatedGibbon        CTAGAGGAAAACTCTGAGGAACATTCAATGTCACCTGAAAGAGAAATGGGAAACGAGAAC
AgileGibbon           CTAGAGGAAAACTCTGAGGAACATTCAATGTCACCTGAAAGAGAAATGGGAAACGAGAAC
WHGibbon              CTAGAGGAAAACTCTGAGGAACATTCAATGTCACCTGAAAGAGAAATGGGAAACGAGAAC
Siamang               CTAGAGGAAAACTCTGAGGAACATTCAATGTCACCTGAAAGAGAAATGGGAAACGAGAAC
RCGibbon              CTAGAGGAAAACTCTGAGGAACATTCAATGTCACCTGAAAGAGAAATGGGAAACGAGAAC
WCGibbon              CTAGAGGAAAACTCTGAGGAACATTCAATGTCACCTGAAAGAGAAATGGGAAACGAGAAC
Marmoset              CTAGAGGAAAACTCTGAAGAACATTCAATGTCACCTGAAAGAGCAATGGGAAACAAGAAC
SqMnky                CTAGAGGAAAACTCTGAGGAACATTCAGTGTCATCTGAAAGAGCAATGGGAAACAAGAAC
TitiMonkey            CTAGAGGAAAACTCTGAGGAACATTCAATGTCACCTGGAAGAGCAATGGGAAACAAGAAC
HowlerMk              CTAGAAGAAAACTCTGAGGAACATTCAATGTCACCTGAAAGAGCAATGGGAAACAAGAAC
                      ***** ******* *** ********* ***** * * ***** * * *****  *****

Human                 ---ATTCCAAGTACAGTGAGCACAATTAGCCGTAATAACATTAGAGAAAATGTTTTTAAA
Rhesus                ATCATTCCAAGTACAGTGAGCACAATTAGCCATAATAACATTAGAGAAAATGCTTTTAAA
CEMacaque             ATCATTCCAAGTACAGTGAGCACAATTAGCCATAATAACATTAGAGAAAATGCTTTTAAA
BMangabey             ATCATTCCAAGTACAGTGAGCACAATTAGCCATAATAACATTAGAGAAAATGCTTTTAAA
OliveBaboon           ATCATTCCAAGTACAGTGAGCACAATTAGCCATAATAACATTAGAGAAAATGCTTTTAAA
Talapoin              ATCATTCCAAGTACAGTGAGCACAATTAGCCGTAATAACATTAGAGAAAATGCTTTTAAA
WolfsGuenon           ATCATTCCAAGTACAGTGAGCACAACTGGCCATAATAACATTAGAGAAAATGCTTTTAAA
Colobus               ATCATTCCAAGTGCAGTGAGCACAATTAGCCATAATAACATTAGAGAAAATGCTTTTAAA
Chimpanzee            ---ATTCCAAGTACAGTGAGCACAATTAGCCGTAATAACATTAGAGAAAATGTTTTTAAA
Bonobo                ---ATTCCAAGTACAGTGAGCACAATTAGCCGTAATAACATTAGAGAAAATCTTTTTAAA
Gorilla               ---ATTCCAAGTACAGTGAGCACAATTAGCCGTAATAACATTAGAGAAAATGTTTTTAAA
Orangutan             ---ATTCCAAGTACAGTGAGCATAATTAGCCGTAATAACATTAGAGAAAATGTTTTTAAA
BorneoOrangutan       ---ATTCCAAGTACAGTGAGCATAATTAGCCGTAATAACATTAGAGAAAATGTTTTTAAA
PileatedGibbon        ---ATTCCAAGTACAGTGAGCACAATTAGCCGTAATAACATTAGAGAAAGTGCTTTTAAA
AgileGibbon           ---ATTCCAAGTACAGTGAGCACAATTAGCCGTAATAACATTAGAGAAAGTGCTTTTAAA
WHGibbon              ---ATTCCAAGTACAGTGAGCACAATTAGCCGTAATAACATTAGAGAAAGTGCTTTTAAA
Siamang               ---ATTCCAAGTACAGTGAGCACAATTAGCCGTAATAACATTAGAGAAAGTGCTTTTAAA
RCGibbon              ---ATTCCAAGTACAGTGAGCACAATTAGCCGTAATAACATTAGAGAAAGTGCTTTTAAA
WCGibbon              ---ATTCCAAGTACAGTGAGCACAATTAGCCGTAATAACATTAGAGAAAGTGCTTTTAAA
Marmoset              ATCATTCCAAGTACAGTGAGCACAATTAGCCACAATAAC---AGAGAAAATGCTTTTAAA
SqMnky                ATCATTCCAAGTACAGTGAGCACAGTTAGCCATAATAAC---AGAGAAAAAGCTTTTAAA
TitiMonkey            ATCATTCCAAGTACAGTGAGCACAATTAGCCATAATAAC---AGAGAAAATGCTTTTAAA
HowlerMk              ATCATTCCAAGTACAGTGAGCACAATTAGCCATAATAAC---AGAGAAAATGCTTTTAAA
                         ********* ********* *  * ***  ******   *******    *******

Human                 GAAGCCAGCTCAAGCAATATTAATGAAGTAGGTTCCAGTACTAATGAA------------
Rhesus                GAAGCCAGCTCGAGCAATATTAATGAAGTAGGTTCCAGTACTAATGAA------------
CEMacaque             GAAGCCAGCTCGAGCAATATTAATGAAGTAGGTTCCAGTACTAATGAA------------
BMangabey             GAAGCCAGCTGGAGCAATATTAATGAAGTAGGTTCCAGTACTAATGAA------------
OliveBaboon           GAAGCCAGCTGGAGCAATATTAATGAAGTAGGTTCCAGTACTAATGAA------------
Talapoin              GAAGCCAGCTCGAGCAATATTAATGAAGTAGGTTCCAGTACTAATGAA------------
WolfsGuenon           AAAGCCAGCTCGAGCAATATTAATGAAGTAGGTTCCAGTACTAATGAA------------
Colobus               GGAGCCAGCTCGAGCAATAGTAATGAAGTAGGTTCCAGTACTAATGAA------------
Chimpanzee            GAAGCCAGCTCAAGCAATATTAATGAAGTAGGTTCCAGTACTAATGAA------------
Bonobo                GAAGCCAGCTCAAGCAATATTAATGAAGTAGGTTCCAGTACTAATGAA------------
Gorilla               GAAGCCAGCTCAAGCAATATTAATGAAGTAGGTTCCAGTACTAATGAA------------
```

```
Orangutan              GAAGCCAGCTCAAGCAATATTAATGAAGTAGGTTCCAGTACTAATGAA------------
BorneoOrangutan        GAAGCCAGCTCAAGCAATATTAATGAAGTAGGTTCCAGTACTAATGAA------------
PileatedGibbon         GAAGCCAGCTCAAGCAATATTAATGAAGTAGGTTCCAGTACTAATGAA------------
AgileGibbon            GAAGCCAGCTCAAGCAATATTAATGAAGTAGGTTCCAGTACTAATGAA------------
WHGibbon               GAAGCCAGCTCAAGCAATATTAATGAAGTAGGTTCCAGTACTAATGAA------------
Siamang                GAAGCCAGCTCAAGCAATATTAATGAAGTAGGTTCCAGTACTAATGAA------------
RCGibbon               GAAGCCAGCTCAAGCAATATTAATGAAGTAGGTTCCAGTACTAATGAA------------
WCGibbon               GAAGCCAGCTCAAGCAATATTAATGAAGTAGGTTCCAGTACTAATGAA------------
Marmoset               GAAACCAGCTCAAGCAGTATTAATGAAGTAGGTTCCAGTACTAATGAA------------
SqMnky                 GAAACCAGCTCAAGCAGTATTAATGAAGTAGGTTCCAGTACTAATGAA------------
TitiMonkey             GAAACCAGCTCAAGCAGTATTAATGAAGTAGGTTCCAGTACTAATGAA------------
HowlerMk               GAAACCAGCTCAAGCAGTATTTATGAAGTAGGTTCCAGTACTAATGAAGCAGGTTCTAGT
                         * ******  **** ** * **************************

Human                  ---------GTGGGCTCCAGTATTAATGAAATAGGTTCCAGTGATGAAAACATTCAAGCA
Rhesus                 ---------GTGGGCTCCAGTATTAATGAAGTAGGTCCCAGTGATGAAAACATTCAAGCA
CEMacaque              ---------GTGGGCTCCAGTATTAATGAAGTAGGTTCCAGTGATGAAAACATTCAAGCA
BMangabey              ---------GTGGGCTCCAGTATTAATGAAGTAGGTTCCAGTGATGAAAACATTCAAGCA
OliveBaboon            ---------GTGGGCTCCAGTATTAATGAAGTAGGTTCCAGTGATGAAAACATTCAAGCA
Talapoin               ---------GTGGGCTCCAGTATTAATGAAGTAGGTTCCAGTGATGAAAACATTCAAGCA
WolfsGuenon            ---------GTGGGCTCCAGTATTAATGAAGTAGGTTCCAGTGATGAAAACATTCAAGCA
Colobus                ---------GTGGGCTCCAGTATTAATGAAGTAGGTTCCAGTGATGAAAACATTCAGGCA
Chimpanzee             ---------GTGGGCTCCAGTATTAATGAAGTAGGTTCCAGTGATGAAAACATTCAAGCA
Bonobo                 ---------GTGGGCTCCAGTATTAATGAAGTAGGTTCCAGTGATGAAAACATTCAAGCA
Gorilla                ---------GTGGGCTCCAGTATTAATGAAGTAGGTTCCAGTGATGAAAACATTCAAGCA
Orangutan              ---------GTGGGCTCCAGTATTAATGAAGTAGGTTCCAGTGATGAAAACATTCAAGCA
BorneoOrangutan        ---------GTGGGCTCCAGTATTAATGAAGTAGGTTCCAGTGATGAAAACATTCAAGCA
PileatedGibbon         ---------GTGGGCTCCAGTATTAATGAAGTAGGTTCCAGTGATGAAAACATTCAAGCA
AgileGibbon            ---------GTGGGCTCCAGTATTAATGAAGTAGGTTCCAGTGATGAAAACATTCAAGCA
WHGibbon               ---------GTGGGCTCCAGTATTAATGAAGTAGGTTCCAGTGATGAAAACATTCAAGCA
Siamang                ---------GTGGGCTCCAGTATTAATGAAGTAGGTTCCAGTGATGAAAACATTCAAGCA
RCGibbon               ---------GTGGGCTCCAGTATTAATGAAGTAGGTTCCAGTGATGAAAACATTCAGGCA
WCGibbon               ---------GTGGGCTCCAGTATTAATGAAGTAGGTTCCAGTGATGAAAACATTCAGGCA
Marmoset               ---------GTAGGCTCCAGTATTAATGAAGTAGGTTCCAGTGATGAAAACATTCAAGCA
SqMnky                 ---------GTAGGCTCCAGTATTAATGAAGTAGGTTCCAGTGATGAAAACATTCAAGCA
TitiMonkey             ---------GTAGGTTCTAGTACTAATGAAGTAGGTTCCAGTGATGAAAACATTCAAGCA
HowlerMk               ACTAATGAAGTAGGCTCCAGTATTAATGAAGTAGGTTCCAGTGATGAAAACATTCAAGCA
                                ** ** ** **** ******* ***** ******************* ***

Human                  GAACTAGGTAGAAACAGAGGGCCAAAATTGAATGCTATGCTTAGATTAGGGGTTTTGCAA
Rhesus                 GAACTAGGTAGAAACAGAGGGCCAAAATTGAATGCTGTGCTTAGATTAGGGCTTTTGCAA
CEMacaque              GAACTAGGTAGAAACAGAGGGCCAAAATTGAATGCTGTGCTTAGATTAGGGCTTTTGCAA
BMangabey              GAACTAGGTAGAAACAGAGGGCCAAAATTGAATGCTGTGCTTAGATTAGGGCTTTTGCAA
OliveBaboon            GAACTAGGTAGAAACAGAGGGCCAAAATTGAATGCTGTGCTTAGATTAGGGCTTTTGCAA
Talapoin               GAACTAGGTAGAAACAGAGGGCCAAAATTGAATGCTGTGCTTAGATTAGGGCTTTTGCAA
WolfsGuenon            GAACTAGGTAGAAACAGAGGGCCAAAATTGAATGCTGTGCTTAGATTAGGGCTTTTGCAA
Colobus                GAACTAGGTAGAAACAGAGGGCCACAATTGAATGCTGTGCTTAGATTAGGGCTTTTGCAA
Chimpanzee             GAACTAGGTAGAAACAGAGGGCCAAAATTGAATGCTATGCTTAGATTAGGGGTTTTGCAA
Bonobo                 GAACTAGGTAGAAACAGAGGGCCAAAATTGAATGCTATGCTTAGATTAGGGGTTTTGCAA
Gorilla                GAACTAGGTAGAAACAGAGGGCCAAAATTGAATGCTATGCTTAGATTAGGGGTTTTGCAA
Orangutan              GAACTAGGTAGAAGCAGAGGGCCAAAATTGAATGCTGTGCTTAGATTAGGGGTTTTGCAA
BorneoOrangutan        GAACTAGGTAGAAGCAGAGGGCTAAAATTGAATGCTATGCTTAGATTAGGGGTTTTGCAA
PileatedGibbon         GAACTAGGTAGAAACAGAGGGCCAAAATTGAATGCTATGCTTAGATTAGGGGTTTTGCAA
AgileGibbon            GAACTAGGTAGAAACAGAGGGCCAAAATTGAATGCTATGCTTAGATTAGGGGTTTTGCAA
WHGibbon               GAACTAGGTAGAAACAGAGGGCCAAAATTGAATGCTATGCTTAGATTAGGGGTTTTGCAA
Siamang                GAACTAGGTAGAAACAGAGGGCCAAAATTGAATGCTATGCTTAGATTAGGGGTTTTGCAA
RCGibbon               GAACTAGGTAGAAACAGAGGGCCAAAATTGAATGCTATGCTTAGATTAGGGGTTTTGCAA
WCGibbon               GAACTAGGTAGAAACAGAGGGCCAAAATTGAATGCTATGCTTAGATTAGGGGTTTTGCAA
Marmoset               GAGATAGGTAGAAACAGAAGGCCAAAATTGAATGCTATGCTTAGATTAGAGCTTTTGCAA
SqMnky                 GAGATGGGTAGAAACAGAAGGCCAAAACTGAATGCTATGCTTAGATTAGGGCTTTTGCAA
TitiMonkey             GAGATAGGTAGAAACAGAAGGCCAAAACTGAATGCTATGCTTAGATTAGGGCTTTTGCAA
HowlerMk               GAGCTAGGTAGAAACAGAAGGCCAAAATTGAATGCTATGCTTAGATTAGGGCTTTTGCAA
                       **  * ******* **** *** * ** ******** ************ * ********

Human                  CCTGAGGTCTATAAACAAAGTCTTCCTGGAAGTAATTGTAAGCATCCTGAAATAAAAAAG
Rhesus                 CCTGAGGTCTGTAAACAAAGTCTTCCTATAAGTAATTGTAAGCATCCTGAAATAAAAAAG
CEMacaque              CCTGAGGTCTGTAAACAAAGTCTTCCTATAAGTAATTGTAAGCATCCTGAAATAAAAAAG
BMangabey              CCTGAGGTCTGTAAACAAAGTCTTCCTATAAGTAATTGTAAGCATCCTGAAATAAAAAAG
OliveBaboon            CCTGAGGTCTGTAAACAAAGTCTTCCTATAAGTAATTGTAAGCATCCTGAAATAAAAAAG
Talapoin               CCTGAGGTCTGTAAACAAAGTCTTCCTATAAGTAATTGTAAGCATCCTGAAATAAAAAAG
WolfsGuenon            CCTGAGGTCTGTAAACAAAGTCTTCCTATAAGTAATTGTAAGCATCCTGAAATAAAAAAG
Colobus                CCTGAGGTCTGTAAACAAAGTCTTCCTATAAGTAATTGTAAGCATCCTGAAATAAAAAAG
Chimpanzee             CCTGAGGTCTATAAACAAAGTCTTCCTGGAAGTAATTGTAAGCATCCTGAAATAAAAAAG
```

```
Bonobo                 CCTGAGGTCTATAAACAAAGTCTTCCTGGAAGTAATTGTAAGCATCCTGAAATAAAAAAG
Gorilla                CCTGAGGTCTATAAACAAAGTCTTCCTGGAAGTAATTGTAAGCATCCTGAAATAAAAAAG
Orangutan              CCTGAGGTCTATAAACAAAGTTTTCCTGGAAGTAATGGTAAGCATCCTGAAATAAAAAAG
BorneoOrangutan        CCTGAGGTCTATAAACAAAGTTTTCCTGGAAGTAATGGTAAGCATCCTGAAATAAAAAAG
PileatedGibbon         CCTGAGGTCTATAAACAAAGTCTTCCTAGAAGTAATTGTAAGCATCCTGAAATAAAAAGG
AgileGibbon            CCTGAGGTCTATAAACAAAGTCTTCCTAGAAGTAATTGTAAGCATCCTGAAATAAAAAGG
WHGibbon               CCTGAGGTCTATAAACAAAGTCTTCCTAGAAGTAATTGTAAGCATCCTGAAATAAAAAGG
Siamang                CCTGAGGTCTATAAACAAAGTCTTCCTAGAAGTAATTGTAAGCATCCTGAAATAAAAAGA
RCGibbon               CCTGAGGTCTATAAACAAAGTCTTCCTAGAAGTAATTGTAAGCATCCTGAAATAAAAAGG
WCGibbon               CCTGAGGTCTATAAACAAAGTCTTCCTAGAAGTAATTGTAAGCATCCTGAAATAAAAAGG
Marmoset               CCTGAGATGTATAAGCAAAGTCTTCCTATAAGTAATTGTAAACATCCTGACACTAAAAAG
SqMnky                 CCTGAGATTTATAAGCAAAGTCTTCCTATAAGTGATTGTAAACATCCTGAAATTAAAAAG
TitiMonkey             CCTGAGATTTATAAGCAAAGTCTTCCTATAAGTAATTGTAAACATCCTGAAATTAAAAAG
HowlerMk               CCTGAGATTTGTAAGCAAAGTCTTCCTATAAGTGATTGTAAACATCCTGAAATTAAAAAG
                       ****** * * *** ****** *****  **** ** **** ******** *  ****

Human                  CAAGAATATGAAGAAGTAGTTCAGACTGTTAATACAGATTTCTCTCCATATCTGATTTCA
Rhesus                 CAAGAACATGAAGAATTAGTTCAGACTGTTAATACAGACTTCTCTCCATGTCTGATTTCA
CEMacaque              CAAGAACATGAAGAATTAGTTCAGACTGTTAATACAGACTTCTCTCCATGTCTGATTTCA
BMangabey              CAAGAACATGAAGAATTAGTTCAGACTGTTAATACAGACTTCTCTCCATGTCTGATTTCA
OliveBaboon            CAAGAACATGAAGAATTAGTTCAGACTGTTAATACAGACTTCTCTCCATGTCTGATTTCA
Talapoin               CAAGAACATGAAGAAATAGTTCAGACTGTTAATACAGACTTCTCTCCATGTCTGATTTCA
WolfsGuenon            CAAGAACATGAAGAAATAGTTCAGACTGTTAATACAGACTTCTCTCCATGTCTGATTTCA
Colobus                CAAGAACATGAAGAAGTAGTTCAGACTGTTAATACAGACTTCTCTCCATGTCTGATTTCA
Chimpanzee             CAAGAATATGAAGAAGTAGTTCAGACTGTTAATACAGATTTCTCTCCATGTCTGATTTCA
Bonobo                 CAAGAATATGAAGAAGTAGTTCAGACTGTTAATACAGATTTCTCTCCATGTCTGATTTCA
Gorilla                CAAGAATATGAAGAAGTAGTTCAGACTGTTAATACAGATTTCTCTCCATGTCTGATTTCA
Orangutan              CAAGAATATGAAGAAGTACTTCAGACTGTTAATACAGACTTCTCTCCATGTCTGATTTCA
BorneoOrangutan        CAAGAATATGAAGAAGTACTTCAGACTGTTAATACAGACTTCTCTCCATGTCTGATTTCA
PileatedGibbon         CAAGAATATGAAGAAGTAGTTCAGACTGTTAATACAGACTTCTCTCCATGTGTGATTTCA
AgileGibbon            CAAGAATATGAAGAAGTAGTTCAGACTGTTAATACAGACTTCTCTCCATGTGTGATTTCA
WHGibbon               CAAGAATATGAAGAAGTAGTTCAGACTGTTAATACAGACTTCTCTCCATGTGTGATTTCA
Siamang                CAAGAATATGAAGAAGTAGTTCAGACTGTTAATACAGACTTCTCTCCATGTGTGATTTCA
RCGibbon               CAAGAATATGAAGAAGTAGTTCAGACTGTTAATACAGACTTCTCTCCATGTGTGATTTCA
WCGibbon               CAAGAATATGAAGAAGTAGTTCAGACTGTTAATACAGACTTCTCTCCATGTGTGATTTCA
Marmoset               CAAGAACATGAA---GTAGTTCAGACTGTTAATACAGACCTCTCTCCATGTCTGATTTCA
SqMnky                 CAGGAACATGAAGAA---GTTCAGACTGTTAATACAGACCTCTCTCCATGTCTGATTTCA
TitiMonkey             CAAGAACATGAAGAAGTAGTTCAGACTGTCAATACAGACCTCTCTCCATGTCTGATTTCA
HowlerMk               CAAGAACATGAAGAAGTAGTTCAGACTGTTAATACAGACGTCTCTCTATGTCTGATTTCA
                       ** *** *****      *********** ********  ****** ** * ********

Human                  GATAACTTAGAACAGCCTATGGGAAGTAGTCATGCATCTCAGGTTTGTTCTGAGACACCT
Rhesus                 GATAACCTAGAACAGCCTATGGGAAGTAGTCATGCGTCTGAGGTTTGTTCTGAGACTCCT
CEMacaque              GATAACCTAGAACAGCCTATGGGAAGTAGTCATGCGTCTGAGGTTTGTTCTGAGACTCCT
BMangabey              GATAACCTAGAACAGCCTATGGGAAGTAGTCATGCGTCTGAGGTTTGTTCTGAGACTCCT
OliveBaboon            GATAACCTAGAACAGCCTATGGGAAGTAGTCATGCGTCTGAGGTTTGTTCTGAGACTCCT
Talapoin               GATAACCTAGAACAGCCTATGGGAAGTAGTCATGCGTCTGAGGTTTGTTCTGAGACTCCT
WolfsGuenon            GATAACCTAGAACAGCCTATGGGAAGTAGTCATGCGTCTGAGGTTTGTTCTGAGACTCCT
Colobus                GATAACCTAGAACAGCCTATGGGAAGTAGTCATGTGTCTGAGGTTTGTTCTGAGACTCCT
Chimpanzee             GATAACTTAGAACAGCCTATGGGAAGTAGTCATGCATCTCAGGTTTGTTCTGAGACACCT
Bonobo                 GATAACTTAGAACAGCCTATGGGAAGTAGTCATGCATCTCAGGTTTGTTCTGAGACACCT
Gorilla                GATAACTTAGAACAGCCTATGGGAAGTAGTCATGCATCTCAGGTTTGTTCTGAGACACCT
Orangutan              GATAACCTAGAACAGCCTATGAGAAGTAGTCATGCATCTCAGGTTTGTTCTGAGACACCT
BorneoOrangutan        GATAACCTAGAACAGCCTATGAGAAGTAGTCATGCATCTCAGGTTTGTTCTGAGACACCT
PileatedGibbon         GATAACCTAGAACAGCCTATGGGAAGTAGTCATGCATCTCAGGTTTGTTCTGAGACACCT
AgileGibbon            GATAACCTAGAACAGCCTATGGGAAGTAGTCATGCATCTCAGGTTTGTTCTGAGACACCT
WHGibbon               GATAACCTAGAACAGCCTATGGGAAGTAGTCATGCATCTCAGGTTTGTTCTGAGACACCT
Siamang                GATAACCTAGAACAGCCTATGGGAAGTAGTCATGCATCTCAGGTTTGTTCTGAGACACCT
RCGibbon               GATAACCTAGAACAGCCTATGGGAAGTAGTCATGCATCTCAGGTTTGTTCTGAGACACCT
WCGibbon               GATAACCTAGAACAGCCTATGGGAAGTAGTCATGCATCTCAGGTTTGTTCTGAGACACCT
Marmoset               GATAACGTAGAACAGCATATGGGAAGCAGTCATACATGTCAGGTTTGTTCTGAGACACCT
SqMnky                 GATAACCTAGAACAGCATGTGGGAAGCAGTCATACATCTCAGGTTTGTTCCGAGACACCT
TitiMonkey             GATAATCTAGAACAGCATATGGGAAACAGTCATACATCTCAGGTTTGTTCTGAGACACCT
HowlerMk               TATAACCTAGAACAGCATATGGGAAGCAGTCATACATCTCAGGTTTGTTCTGAGACACCT
                        ****  ********* * ** ***  ******   * * ********** ***** ***

Human                  GATGACCTGTTAGATGATGGTGAAATAAAGGAAGATACTAGTTTTGCTGAAAATGACATT
Rhesus                 GATGATCTGTTAGATGATGGTGAAATAAAGGAAGATACTAGTTTTGCTGCAAATGACATT
CEMacaque              GATGATCTGTTAGATGATGGTGAAATAAAGGAAGATACTAGTTTTGCTGAAAATGACATT
BMangabey              GATGATCTGTTAGATGATGGTGAAATAAAGGAAGATACTAGTTTTGCTGAAAATGACATT
OliveBaboon            GATGATCTGTTAGATGATGGTGAAATAAAGGAAGATACTAGTTTTGCTGAAAATGACATT
Talapoin               GATGATCTGTTAGATGATGGTGAAATAAAGGAAGATACTAGTTTTGCTGAAAATGACATT
WolfsGuenon            GATGATCTGTTAGATGATGGTGAAATAAAGGAAGATACTAGTTTTGCTGAAAATGACATT
```

```
Colobus                GATGACCTGTTAGATGATGGTGAAATAAAGGAAGATACTAGTTTTGCTGAAAATGACATT
Chimpanzee             GATGACCTGTTAGATGATGGTGAAATAAAGGAAGATACTAGTTTTGCTGAAAATGACATT
Bonobo                 GATGACCTGTTAGATGATGGTGAAATAAAGGAAGATACTAGTTTTGCTGAAAATGACATT
Gorilla                GATGACCTGTTAGATGATGGTGAAATAAAGGAAGATACTAGTTTTGCTAAAAATGACATT
Orangutan              AATGACCTGTTAGATGATGGTGAAATAAAGGAAGATACTAGTTTTGCTGAAAATGACATT
BorneoOrangutan        GATGACCTGTTAGATGATGGTGAAATAAAGGAAGATACTAGTTTTGCTGAAAATGACATT
PileatedGibbon         GATGACCTGCTAGATGATGGTGAAATAAAGGAAGATACTAGTTTTGCTGAAAATGACATT
AgileGibbon            GATGACCTGCTAGATGATGGTGAAATAAAGGAAGATACTAGTTTTGCTGAAAATGACATT
WHGibbon               GATGACCTGCTAGATGATGGTGAAATAAAGGAAGATACTAGTTTTGCTGAAAATGACATT
Siamang                GATGACCTGCTAGATGATGGTGAAATAAAGGAAGATACTAGTTTTGCTGAAAATGACATT
RCGibbon               GATGACCTGTTAGATGATGGTGAAATAAAGGAAGATACTAGTTTTGCTGAAAATGACATT
WCGibbon               GATGACCTGTTAGATGATGGTGAAATAAAGGAAGATACTAGTTTTGCTGAAAATGACATT
Marmoset               GAGGACCTGTTAGATGATGGTGAAATAAAGGAAGATACTAGTTTTGCTGAATATGGCATT
SqMnky                 GACGACCTGTCAGATGAAGGTGAAATAAAGGAAGATACTAGTTTTGCTGAATATGGCAGT
TitiMonkey             GACGACCTGTTAGATGATGGTGAAATAAAGGAAGATACTGGTTTTGCTGAATATGGCATT
HowlerMk               GACAACCTGTTAGATGATGGTGAAATAAAGGAAGATACTAGTTTTGCTGAATATGGCATT
                        *  * ***  ****** ********************* ********  * *** ** *

Human                  AAGGAAAGTTCTGCTGTTTTTAGCAAAAGCGTCCAGAAAGGAGAGCTTAGCAGGAGTCCT
Rhesus                 AAGGAGAGTTCTGCTGTTTTTAGCAAAAGCATCCAGAGAGGAGAGCTCAGCAGGAGCCCT
CEMacaque              AAGGAGAGTTCTGCTGTTTTTAGCAAAAGCATCCAGAGAGGAGAGCTCAGCAGGAGCCCT
BMangabey              AAGGAGAGTTCTGCTGTTTTTAGCAAAAGCGTCCAGAGAGGAGAGCTCAGCAGGAGCCCT
OliveBaboon            AAGGAGAGTTCTGCTGTTTTTAGCAAAAGCGTCCAGAGAGGAGAGCTCAGCAGGAGCCCT
Talapoin               AAGGAGAGTTCTGCTGTTTTTAGCAAAAGTGTCCAGAGAGGAGAGCTCAGCAGGAGCCCT
WolfsGuenon            AAGGAGAGTTCTGCTGTTTTTAGCAAAAGCGTCCAGAGAGGAGAGCTCAGCAGGAGCCCT
Colobus                AAGGAGAGTTCTGCTGTTTTTAGCAAAAGCGTCCAGAGAGGAGAGCTCAGCAGGAGCCCT
Chimpanzee             AAGGAAAGTTCTGCTGTTTTTAGCAAAAGCGTCCAGAGAGGAGAGCTTAGCAGGAGTCCT
Bonobo                 AAGGAAAGTTCTGCTGTTTTTAGCAAAAGCGTCCAGAGAGGAGAGCTTAGCAGGAGTCCT
Gorilla                AAGGAAAGTTCTGCTGTTTTTAGCAAAAACGTCCAGAGAGGAGAGCTTAGCAGGAGTCCT
Orangutan              AAGGAAAGTTCTGCTGTTTTTAGCAAAAGCGTCCAGAGAGGAGAGCTTAGCAGGAGTCCT
BorneoOrangutan        AAGGAAAGTTCTGCTGTTTTTAGCAAAAGCGTCCAGAGAGGAGAGCTTAGCAGGAGTCCT
PileatedGibbon         AAGGAAAGTTCTGCTGTTTTTAGCAAAAGCGTCCAGAGAGGAGAGCTTAGCAGGAGTCCT
AgileGibbon            AAGGAAAGTTCTGCTGTTTTTAGCAAAAGCGTCCAGAGAGGAGAGCTTAGCAGGAGTCCT
WHGibbon               AAGGAAAGTTCTGCTGTTTTTAGCAAAAGCGTCCAGAGAGGAGAGCTTAGCAGGAGTCCT
Siamang                AAGGAAAGTTCTGCTGTTTTTAGCAAAAGCGTCCAGAGAGGAGAGCTTAGCAGAAGTCCT
RCGibbon               AAGGAAAGTTCTGCTGTTTTTAGCAAAAGCGTCCAGAGAGGAGAGCTTAGCAGGAGTCCT
WCGibbon               AAGGAAAGTTCTGCTGTTTTTAGCAAAAGCGTCCAGAGAGGAGAGCTTAGCAGGAGTCCT
Marmoset               AAGGAAACTTCTGCTGTTTTTAGCAAAAGTGTCCAGAGAGGAGAGCTCAGCAGGAGCCCT
SqMnky                 AAGGAGACTTCTGCTATTTTTAGCAAAAGTGTCCAGAGAGGAGAGCTCAGCAGGAGCCCT
TitiMonkey             AAGGAGACTTCTGCTGTTTTTAGCAAAAGTGTCCAGAGAGGAGAGCTCAGCAGGAGCCCT
HowlerMk               AAGGAGACTTCTACTGTTTTTAGCAAAAGTGTCCAGAGAGGAGAGCTCAGCAGGAGCCCT
                       ***** * **** ** ************   ****** ********* ***** ** ***

Human                  AGCCCTTTCACCCATACACATTTGGCTCAGGGTTACCGAAGAGGGGCCAAGAAATTAGAG
Rhesus                 AGCCCTTTCACCCATACACATTTAGCTCAGGGTTACCAAAAAGAGGCCAAGAAATTAGAG
CEMacaque              AGCCCTTTCACCCATACACATTTAGCTCAGGGTTACCGAAAAGAGGCCAAGAAATTAGAG
BMangabey              AGCCCTTTCACCCATACACATTTGGCTCAGGGTTACCAAAAAGGGGCCAAGAAATTAGAG
OliveBaboon            AGCCCTTTCACCCATACACATTTGGCTCAGGGTTACCGAAAAGGGGCCAAGAAATTAGAG
Talapoin               AGCCCTTTCACCCATACACATTTGGCTCAGGGTTACCGAAGAGGGGCCAAGAAATTAGAG
WolfsGuenon            AGCCCTTTCACCTATACACATTTGGCTCAGGGTTACCGAAGAGGGGCCAAGAAATTAGAG
Colobus                AGCCCTTTCACCCATACACATTTGGCTCAGGGTTACCGAAGAGGGGCCAAGAAATTAGAG
Chimpanzee             AGCCCTTTCACCCATACACATTTGGCTCAGGGTTACCGAAGAGGGGCCAAGAAATTAGAG
Bonobo                 AGCCCTTTCACCCATACACATTTGGCTCAGGGTTACCGAAGAGGGGCCAAGAAATTAGAG
Gorilla                AGCCCTTTCACCCATACACATTTGGCTCAGGGTTACCGAAGAGGGGCCAAGAAATTAGAG
Orangutan              AGCCCTTTCACCCATACACATTTGGCTCAGGGTTACCGAAGAGGGGCCAAGAAATTAGAG
BorneoOrangutan        AGCCCTTTCACCCATACACATTTGGCTCAGGGTTACCGAAGAGGGGCCAAGAAATTAGAG
PileatedGibbon         AGCCCTTTCACCCATACACATTTGGCTCAGGGTTACCGAAGAGGGGCCAAGAAATTAGAG
AgileGibbon            AGCCCTTTCACCCATACACATTTGGCTCAGGGTTACCGAAGAGGGGCCAAGAAATTAGAG
WHGibbon               AGCCCTTTCACCCATACACATTTGGCTCAGGGTTACCGAAGAGGGGCCAAGAAATTAGAG
Siamang                AGCCCTTTCACCCATACACATTTGGCTCAGGGTTACCGAAGAGGGGCCAAGAAATTAGAG
RCGibbon               AGCCCTTTCACCCACACACATTTGGCTCAGGGTTACCGAAGAGGGGCCAAGAAATTAGAG
WCGibbon               AGCCCTTTCACCCACACACATTTGGCTCAGGGTTACCGAAGAGGGGCCAAGAAATTAGAG
Marmoset               AGCCCTTTCACCCATACGCATTTGGCTCAGGTTTACCAAAGAGGGGCCAAGAAATTAGAG
SqMnky                 AGCCCTTTCACCCATACACATTTGGCTCAGGTTTACCAAAGAGGGACCAAGAAATTAGAG
TitiMonkey             TGCCCTTTCACCCAAACACATTTGGCTCAGGTTTACCAAAGAGGGGCCAAGAAATTAGAG
HowlerMk               AGCCCTTTCACCCATACACATTTGGCTCAGGTTTACCAAAGAGGGGCCAAGAAATTAGAG
                        *********** * ** ***** ******* ***** ** ** * **************

Human                  TCCTCAGAAGAGAACTTATCTAGTGAGGATGAAGAGCTTCCCTGCTTCCAACACTTGTTA
Rhesus                 TCCTCAGAAGAGAACTTATCTAGTGAGGATGAAGAGCTTCCCTGCTTCCAACACTTGTTA
CEMacaque              TCCTCAGAAGAGAACTTATCTAGTGAGGATGAAGAGCTTCCCTGCTTCCAACACTTGTTA
BMangabey              TCCTCAGAAGAGAACTTATCTAGTGAGGATGAAGAGCTTCCCTGCTTCCAACACTTGTTA
OliveBaboon            TCCTCAGAAGAGAACTTATCTAGTGAGGATGAAGAGCTTCCCTGCTTCCAACACTTGTTA
```

```
Talapoin              TCCTCAGAAGAGAACTTATCTAGTGAGGATGAAGAGCTTCCCTGCTTCCAACACTTATTA
WolfsGuenon           TCCTCAGAAGAGAACTTATCTAGTGAGGATGAAGAGCTTCCCTGCTTCCAACACTTATTA
Colobus               TCCTCGGAAGAGAACTTATCTAGTGAGGATGAAGAGCTTCCCTGCTTCCAACACTTGTTA
Chimpanzee            TCCTCAGAAGAGAACTTATCTAGTGAGGATGAAGAGCTTCCCTGCTTCCAACACTTGTTA
Bonobo                TCCTCAGAAGAGAACTTATCTAGTGAGGATGAAGAGCTTCCCTGCTTCCAACACTTGTTA
Gorilla               TCCTCAGAAGAGAACTTATCTAGTGAGGATGAAGAGCTTCCCTGCTTCCAACACTTGTTA
Orangutan             TCCTCAGAAGAGAACTTATCTAGTGAGGATGAAGAGCTTCCCTGCTTCCAACACTTGTTA
BorneoOrangutan       TCCTCAGAAGAGAACTTATCTAGTGAGGATGAAGAGCTTCCCTGCTTCCAACACTTGTTA
PileatedGibbon        TCCTCAGAAGAGAACTTATCTAGTGAGGATGAAGAGCTTCCCTGCTTCCAACACTTGTTA
AgileGibbon           TCCTCAGAAGAGAACTTATCTAGTGAGGATGAAGAGCTTCCCTGCTTCCAACACTTGTTA
WHGibbon              TCCTCAGAAGAGAACTTATCTAGTGAGGATGAAGAGCTTCCCTGCTTCCAACACTTGTTA
Siamang               TCCTCAGAAGAGAACTTATCTAGTGAGGATGAAGAGCTTCCCTGCTTCCAACACTTGTTA
RCGibbon              TCCTCAGAAGAGAACTTATCTAGTGAGGATGAAGAGCTTCCCTGCTTCCAACACTTGTTA
WCGibbon              TCCTCAGAAGAGAACTTATCTAGTGAGGATGAAGAGCTTCCCTGCTTCCAACACTTGTTA
Marmoset              TCCTCGGAAGAGAACTTATCTAGTGAGGAAGAAGAGCTTCCCTGCTTCCAACACTTGTTA
SqMnky                TCCTCGGAAGAGAACTTATCTAGTGAGGAAGAGGAGCTTCCCTGCTTCCAACACTTGTTA
TitiMonkey            TCCTCGGAAGAAAACTTACCTAGTGAGGAAGAAGAGCTTCCCTGCTTCCAACACTTGTTA
HowlerMk              TCCTCGGAAGAGAATTTATCTAGTGAGGAAGAAGAGCTTCCCTGCTTCCAGCACTTGTTA
                      ***** ***** ** *** ********** ** ***************** ***** ***

Human                 TTTGGTAAAGTAAACAATATACCTTCTCAGTCTACTAGGCATAGCACCGTTGCTACCGAG
Rhesus                TTTGGTAAAGTAAGCAATATACCTTCTCAGACTACTAGGCATAGCACTGTTGCTACCGAG
CEMacaque             TTTGGTAAAGTAAGCAATATACCTTCTCAGACTACTAGGCATAGCACTGTTGCTACCGAG
BMangabey             TTTGGTAAAGTAAGCAATATACCTTCTCAGACTACTAGGCATAGCACTGTTGCTACCGAG
OliveBaboon           TTTGGTAAAGTAAGCAATATACCTTCTCAGACTACTAGGCATAGCACTGTTGCTACCGAG
Talapoin              TTTGGTAAAGTAAGCAATATACCTTCTCAGACTACTAGGCATAGCACTGTTGCTACCGAG
WolfsGuenon           TTTGGTAAAGTAAGCAATATACCTTCTCAGACTACTAGGCATAGCACTGTTGCTACCGAG
Colobus               TTTGGTAAAGTAAGCAATATACCTTCTCAGATTACTAGGCATAGCACTGTTGCTACCGAG
Chimpanzee            TTTGGTAAAGTAAGCAATATACCTTCTCAGTCTACTAGGCATAGCACCGTTGCTACCGAG
Bonobo                TTTGGTAAAGTAAGCAATATACCTTCTCAGTCTACTAGGCATAGCACCGTTGCTACCGAG
Gorilla               TTTGGTAAAGTAAGCAATATACCTTCTCAGTCTACTAGGCATAGCACCGTTGCTACCGAG
Orangutan             TTTGGTAAAGTAAGCAATATACCTTCTCAGTCTACTAGGCATAGCACCGTTGCTACCGAG
BorneoOrangutan       TTTGGTAAAGTAAGCAATATACCTTCTCAGTCTACTAGGCATAGCACCGTTGCTACTGAG
PileatedGibbon        TTTGGTAAAGTAAGCAATATACCTTCTCAGTCTACCAGGCATAGCACCGTTGCTACCGAG
AgileGibbon           TTTGGTAAAGTAAGCAATATACCTTCTCAGTCTACCAGGCATAGCACCGTTGCTACCGAG
WHGibbon              TTTGGTAAAGTAAGCAATATACCTTCTCAGTCTACCAGGCATAGCACCATTGCTACCGAG
Siamang               TTTGGTAAAGTAAGCAATATACCTTCTCAGTCTACCAGGCATAGCACCGTTGCTACCGAG
RCGibbon              TTTGGTAAAGTAAGCAATATACCTTCTCAGTCTACTAGGCATAGCACCGTTGCTACCGAG
WCGibbon              TTTGGTAAAGTAAGCAATATACCTTCTCAGTCTACTAGGCATAGCACCGTTGCTACCGAG
Marmoset              TTTGGTAAAGTAAGCAATACACCT---CAGTCTACCAGGCATAACACTGTTGCTATTGAG
SqMnky                TTTGGTAAAGTAAGCAATACACCT---CAGTCTACCAGGCATAGCACTGTTGCTACTGAG
TitiMonkey            TTTGGTAAAGTAAGCAATACACCT---CAGTCTACCAGGCATAGTACTGTTGCTACTGAG
HowlerMk              TTTGGTAAAGTAAGCAATACACCT---CAGTCTACCAGGCATAGCACTTCTGCTGCTGAG
                      ************* ***** ****   ***  *** *******  **   ****   ***

Human                 TGTCTGTCTAAGAACACAGAGGAGAATTTATTATCATTGAAGAATAGCTTAAATGACTGC
Rhesus                TGTCTGTCTAAGAACACAGAGGAGAATTTATTATCATTGAAGAATAGCTTAACTGACTGT
CEMacaque             TGTCTGTCTAAGAACACAGAGGAGAATTTATTATCATTGAAGAATAGCTTAACTGACTGT
BMangabey             TGTCTGTCTAAGAACACAGAGGAGAATTTATTATCATTGAAGAATAGCTTAACTGACTGT
OliveBaboon           TGTCTGTCTAAGAACACAGAGGAGAATTTATTATCATTGAAGAATAGCTTAACTGACTGT
Talapoin              TGTCTGTCTAAGAACACAGTGGAGAATTTATTATCATTGAAGAATAGCTTAACTGACTGT
WolfsGuenon           TGTCTGTCTAAGACCACAGTGGAGAATTTATTATCATTGAAGAATAGCTTAACTGACTGT
Colobus               TGTCTGTCTAAGAACACAGAGGAGAATTTGTTATCATTGAAGAATAGCTTAACTGACTGT
Chimpanzee            TGTCTGTCTAAGAACACAGAGGAGAATTTATTATCATTGAAGAATAGCTTAAATGACTGC
Bonobo                TGTCTGTCTAAGAACACAGAGGAGAATTTATTATCATTGAAGAATAGCTTAAATGACTGC
Gorilla               TGTCTGTCTAAGAACACAGAGGAGAATTTATTATCATTGAAGAATAGCTTAAATGACTGC
Orangutan             TGTCTGTCTAAGAACACAGAGGAGAATTTATTATCATTGAAGAATAGCTTAAATGACTAC
BorneoOrangutan       TGTCTGTCTAAGAACACAGAGGAGAATTTATTATCATTGAAGAATAGCTTAAATGACTAC
PileatedGibbon        TGTCTGTCTAAGAACACAGAGGAGAATTTATTATCACTGAAGAATAGCTTAAATGACTGC
AgileGibbon           TGTCTGTCTAAGAACACAGAGGAGAATTTATTATCACTGAAGAATAGCTTAAATGACTGC
WHGibbon              TGTCTGTCTAAGAACACAGAGGAGAATTTATTATCACTGAAGAATAGCTTAAATGACTGC
Siamang               TGTCTGTCTAAGAACACAGAGGAGAATTTATTATCACTGAAGAATAGCTTAAATGACTGC
RCGibbon              TGTCTGTCTAAGAACACAGAGGAGAATTTATTATCACTGAAGAATAGCTTAAATGACTGC
WCGibbon              TGTCTGTCTAAGAACACAGAGGAGAATTTATTATCACTGAAGAATAGCTTAAATGACTGC
Marmoset              TGCCTGTCTAAGAACACACAGGAGAATTTATTATCATTGAAGAGTAGCTTAAATGACTGC
SqMnky                TGTCTGTCTAAGAACACGGAGGAGAATTTATTATCATTGAAGAGTAGTTTAAATGACTGC
TitiMonkey            TGTCTGTCTAAGAACATGGAGGAGAATTTATTATCATTGAAGAGTAGCTTAAATGACTGC
HowlerMk              TGTCTGTCTAAGAACACAGAGGAGAATTTATTATCATTGAAGAGTAGCTTAAGTGACTGC
                      ** ********** **    ********* ****** ****** *** **** *****

Human                 AGTAACCAGGTAATATTGGCAAAGGCATCTCAGGAACATCACCTTAGTGAGGAAACAAAA
Rhesus                AGTAACCAGGTAATATTGGCAAAGGCATCCCAGGAACATCACCTTAGTGAGGAAACAAAA
CEMacaque             AGTAACCAGGTAATATTGGCAAAGGCATCCCAGGAACATCACCTTAGTGAGGAAACAAAA
```

```
BMangabey              AGTAACCAGGTAATATTGTCAAAGGCATCCCAGGAACATCACCTTAGTGAGGAAACAAAA
OliveBaboon            AGTAACCAGGTAATATTGTCAAAGGCATCCCAGGAACATCACCTTAGTGAGGAAACAAAA
Talapoin               AGTAACCAGGTAATGTTGGCAACGGCATCCCAGGAACATCACCTTAGTGAGGAAACAAAA
WolfsGuenon            AGTAACCAGGTAATATTGGCAAAGGCATCCCAGGAACATCACCTTAGTGAGGAAACAAAA
Colobus                AGTAACCAGGTAATATTAGCAAAGGCATCCCAGGAACATCACCTTAGTGAGGAAACAAAA
Chimpanzee             AGTAACCAGGTAATATTGGCAAAGGCATCTCAGGAACATCACCTTAGTGAGGAAACAAAA
Bonobo                 AGTAACCAGGTAATATTGGCAAAGGCATCTCAGGAACATCACCTTAGTGAGGAAACAAAA
Gorilla                AGTAACCAAGTAATATTGGCAAAGACATCTCAGGAACATCACCTTAGTGAGGAAACAAAG
Orangutan              AGTAACCAGGTAATATTGGTAAAGGCATCCCAGGAACATCACCTTAGTGAGGAAACAAAA
BorneoOrangutan        AGTAACCAGGTAATATTGGTAAAGGCATCCCAGGAACATCACCTTAGTGAGGAAACAAAA
PileatedGibbon         AGTAACCAGGTAATATTGGCAAAGGCATCCCAGGAACATCACCTTAGTGAGGAAACAAAA
AgileGibbon            AGTAACCAGGTAATATTGGCAAAGGCATCCCAGGAACATCACCTTAGTGAGGAAACAAAA
WHGibbon               AGTAACCAGGTAATATTGGCAAAGGCATCCCAGGAACATCACCTTAGTGAGGAAACAAAA
Siamang                AGTAACCAGGTAATATTGGCAAAGGCATCCCAGGAACATCACCTTAGTGAGGAAACAAAA
RCGibbon               AGTAACCAGGTAATATTGGCAAAGGCATCCCAGGAACATCACCTTAGTGAGGAAACAAAA
WCGibbon               AGTAACCAGGTAATATTGGCAAAGGCATCCCAGGAACATCACCTTAGTGAGGAAACAAAA
Marmoset               AGTAACCAGGTAATATTGGCAAAGGCATCCCAGGAACATCACCTTAGTGAGGAAACAAAA
SqMnky                 AGTAACCAGGTAATATTGGCAAAGGCATCCCAGGAACATCACCTTAGTGAGGAAACAAAA
TitiMonkey             AGTAACCAGGTAATATTGGCAAAGGCATCCCAGGAACATCACCTTAGTGAGGAAACAAAA
HowlerMk               AGTAACCAGGTAATATTGGCAAAGGCATCCCAGGAACATTACCTTAGTGAGGAAACAAAA
                       ******** ***** **   ** * **** ********* *******************

Human                  TGTTCTGCTAGCTTGTTTTCTTCACAGTGCAGTGAATTGGAAGACTTGACTGCAAATACA
Rhesus                 TGTTCTGGTAGCTTGTTTTCTTCACAGTGCAGTGAACTGGAAGACTTGACTGCAAATACA
CEMacaque              TGTTCTGGTAGCTTGTTTTCTTCACAGTGCAGTGAACTGGAAGACTTGACTGCAAATACA
BMangabey              TGTTCTGGTAGCTTGTTTTCTTCACAGTGCAGTGAACTGGAAGACTTGACTGCAAATACA
OliveBaboon            TGTTCTGGTAGCTTGTTTTCTTCACAGTGCAGTGAACTGGAAGACTTGACTGCAAATACA
Talapoin               TGTTCTGGTAGCTTGTTTTCTTCACAGTGCAGTGAACTGGAAGACTTGACTGCACATACA
WolfsGuenon            TGTTCTGGTAGCTTGTTTTCTTCACAGTACAGTGAACTGGAAGACTTGACTGCAAATACA
Colobus                GGTTCTGGTAGCTTGTTTTCTTCACAGTGCAGTGAACTGGAAGACTTGACTGCAAATACA
Chimpanzee             TGTTCTGCTAGCTTGTTTTCTTCACAGTGCAGTGAATTGGAAGACTTGACTGCAAATACA
Bonobo                 TGTTCTGCTAGCTTGTTTTCTTCACAGTGCAGTGAATTGGAAGACTTGACTGCAAATACA
Gorilla                TGTTCTGCTAGCTTGTTTTCTTCACAGTGCAGTGAATTGGAAGACTTGACTGCAAATACA
Orangutan              TGTTCTGCTAGCTTGTTTTCTTCACAGTGCAGTGAACTGGAAGACTTGACTGCAAATACA
BorneoOrangutan        TGTTCTGCTAGCTTGTTTTCTTCACAGTGCAGTGAACTGGAAGACTTGACTGCAAATACA
PileatedGibbon         TGTTCTGGTAGCTTGTTTTCTTCACAGTGCAGTGAACTGGAAGACTTGACCGCAAATACA
AgileGibbon            TGTTCTGGTAGCTTGTTTTCTTCACAGTGCAGTGAACTGGAAGACTTGACCGCAAATACA
WHGibbon               TGTTCTGGTAGCTTGTTTTCTTCACAGTGCAGTGAACTGGAAGACTTGACCGCAAATACA
Siamang                TGTTCTGGTAGCTTGTTTTCTTCACAGTGCAGTGAACTGGAAGACTTGACTGCAAATACA
RCGibbon               TGTTCTGGTAGCTTGTTTTCTTCACAGTGCAGTGAACTGGAGGACTTGACCGCAAATACA
WCGibbon               TGTTCTGGTAGCTTGTTTTCTTCACAGTGCAGTGAACTGGAAGACTTGACCGCAAATACA
Marmoset               TGTTCTGGTAGCTTGTTTTCTTCACAGTGCAGTGAACTGGAAGACTTGACTACAAATACA
SqMnky                 TGTTCTGGTAGCTTGTTTTCTTCACAGTGCAGTGAACTGGAAGACTTGACTACAAATACA
TitiMonkey             TGTTCTGGTAGCTTGTTTTCTTCACAGTGCAGTGAACTGGAAGACTTGACTACAAATACA
HowlerMk               TGTTCTGGTAGCTTGTTTTCTTCACAGTGCAGTGAACTGGAAGACTTAACTACAAATACA
                        ****** ******************** ******* **** ***** **  ** *****

Human                  AACACCCAGGATCCTTTCTTGATTGGTTCTTCCAAACAAATGAGGCATCAGTCTGAAAGC
Rhesus                 AACACCCAGGATCCTTTCTTGATTGGTTCTTCCAAACGAATGAGGCATCAGTCTGAAAGC
CEMacaque              AACACCCAGGATCCTTTCTTGATTGGTTCTTCCAAACGAATGAGGCATCAGTCTGAAAGC
BMangabey              AATACCCAGGATCCTTTCTTGATTGGTTCTTCCAAACGAATGAGGCATCAGTCTGAAAGC
OliveBaboon            AATACCCAGGATCCTTTCTTGATTGGTTCTTCCAAACGAATGAGGCATCAGTCTGAAAGC
Talapoin               AACACCCAGGATCCTTTCTTGATTGGTTCTTCCAAACGAATGAGGCATCAGTCTGAAAGC
WolfsGuenon            AACACCCAGGATCCTTTCTTGATTGGTTCTTCCAAACGAATGAGGCATCAGTCTGAAAGC
Colobus                AACACCCAGGATCCTTTCTTGATTGGTTCTTCCAAACGAATGAGGCATCAGTCTGAAAGC
Chimpanzee             AACACCCAGGATCCTTTCTTGATTGGTTCTTCCAAACAAATGAGGCATCAGTCTGAAAGC
Bonobo                 AACACCCAGGATCCTTTCTTGATTGGTTCTTCCAAACAAATGAGGCATCAGTCTGAAAGC
Gorilla                AACACCCAGGATCCTTTCTTGATTGGTTCTTCCAAACAAATGAGGCATCAGTCTGAAAGC
Orangutan              AACACCCAGGATCGTTTCTTCATTGGTTCTTCTAAACAAATGAGGCATCAGTCTGAAAGC
BorneoOrangutan        AACACCCAGGATCGTTTCTTCATTGGTTCTTCTAAACAAATGAGGCATCAGTCTGAAAGC
PileatedGibbon         AACACCCAGGATCCTTTCTTGATTGGTTCTTCCAAACAAATGAGGCATCATTCTGAAAGC
AgileGibbon            AACACCCAGGATCCTTTCTTGATTGGTTCTTCCAAACAAATGAGGCATCATTCTGAAAGC
WHGibbon               AACACCCAGGATCCTTTCTTGATTGGTTCTTCCAAACAAATGAGGCATCATTCTGAAAGC
Siamang                AACACCCAGGATCCTTTCTTGATTGGTTCTTCCAAACAAATGAGGCATCATTCTGAAAGC
RCGibbon               AACACCCAGGATCCTTTCTTGATTGGTTCTTCCAAACAAATGAGGCATCATTCTGAAAGC
WCGibbon               AACACCCAGGATCCTTTCTTGATTGGTTCTTCCAAACAAATGAGGCATCATTCTGAAAGC
Marmoset               AACACCCAGGATCCTTTCTTGATTGATTCTTCCAAACAAAAGAGGCATCAGTCTGAAAGC
SqMnky                 AACACCCAGGATCCTTTCTTGATTGATTCTTCCAAACGAATGAGGCATCAGTCTGGAAGC
TitiMonkey             AACACCCAGGATCCTTTCTTGATTGATCCTTCCAAACGAATGAGGCATCAGTCTGAAAGC
HowlerMk               AACACCCAAGATCCTTTCTTGATCGATTCTTCCAAACGAATGAGGCGTCAGTCTGAAAGC
                       ** ***** **** ****** ** * * **** **** ** ***** *** **** ****

Human                  CAGGGAGTTGGTCTGAGTGACAAGGAATTGGTTTCAGATGATGAAGAAAGAGGAACGGGC
```

```
Rhesus                 CAGGGAGTTGGTCTGAGTGACAAGGAATTGGTTTCAGATGATGAAGAAAGGGGAACGGGC
CEMacaque              CAGGGAGTTGGTCTGAGTGACAAGGCATTGGTTTCAGATGATGAAGAAAGGGGAACGGGC
BMangabey              CAGGGAGTTGGTCTGAGTGACAAGGAATTGGTTTCAGATGATGAAGAAAGGGGAACGGGC
OliveBaboon            CAGGGAGTTGGTCTGAGTGACAAGGAATTGGTTTCAGCTGATGAAGAAAGGGGAACAGGC
Talapoin               CAGGGAGTTGGTCCGAGTGACAAGGAATTGGTTTCAGATGATGAAGAAAGGGGAACGGGC
WolfsGuenon            CAGGGAGTTGGTCTGAGTGACAAGGAATTGGTTTCAGATCATGAAGAAAGGGGAACGGGC
Colobus                CAGGGAGTTGGTCTGAGTGACAAGGAATTGGTTTCAGATGATGAAGAAAGGGGAACAGGC
Chimpanzee             CAGGGAGTTGGTCTGAGTGACAAGGAATTGGTTTCAGATGATGAAGAAAGAGGAACGGGC
Bonobo                 CAGGGAGTTGGTCTGAGTGACAAGGAATTGGTTTCAGATGATGAAGAAAGAGGAACGGGC
Gorilla                CAGGGAGTTGGTCTGAGTGACAAGGAATTGGTTTCAGATGATGAAGAAAGAGGAACGGGC
Orangutan              CAGGGAGTTGGTCTGAGTGACAAGGAATTGGTTTCAGATGATGAAGAAAGAGGAACGGAC
BorneoOrangutan        CAGGGAGTTGGTCTGAGTGACAAGGAATTGGTTTCAGATGATGAAGAAAGAGGAACGGAC
PileatedGibbon         CAGGGAGTTGGTCTGAGTGACAAGGAATTGGTTTCAGGTGATGAAGAAAGAGGACCAGGC
AgileGibbon            CAGGGAGTTGGTCTGAGTGACAAGGAATTGGTTTCAGGTGATGAAGAAAGAGGACCAGGC
WHGibbon               CAGGGAGTTGGTCTGAGTGACAAGGAATTGGTTTCAGGTGATGAAGAAAGAGGACCAGGC
Siamang                CAGGGAGTTGGTCTGAGTGACAAGGAATTGGTTTCAGGTGATGAAGAAAGAGGACCAGGC
RCGibbon               CAGGGAGTTGGTCTGAGTGACAAGGAATTGGTTTCAGGTGATGAAGAAAGAGGAACAGGC
WCGibbon               CAGGGAGTTGGTCTGAGTGACAAGGAATTGGTTTCAGGTGATGAAGAAAGAGGAACAGGC
Marmoset               CAGGGAGTTGGTCTGAGTGACAAGGAATTGGATTCAGATGATGAAGAAAGGGGAATAGAC
SqMnky                 CAGGGAGTTGGTCTGAGTGACAAGGAATTGGTTTCAGATGATGAAGAAAGGGGAAAAGAT
TitiMonkey             CAGGGAGTTGGTCTGAGTGACAAGGAATTGGTTTCAGATGATGAAGAAAGGGGAACAGAC
HowlerMk               CAGGGAGTTGGTCTGAGTGACAAGGAATTGGTTTCAGATGATGAAGAAAGGGGAACAGAC
                       ************* *********** ***** ***** * ********** ***   *

Human                  TTGGAAGAAAATAATCAAGAAGAGCAAAGCATGGATTCAAACTTAGGTGAAGCAGCATCT
Rhesus                 TTGGAAGAAGATAATCAAGAAGAGCAAAGTGTGGATTCAAACTTAGGTGAAGCAGCATCC
CEMacaque              TTGGAAGAAGATAATCAAGAAGAGCAAAGTGTGGATTCAAACTTAGGTGAAGCAGCATCC
BMangabey              TTGGAAGAAGATAATCAAGAAGAGCAAAGTGTGGATTCAAACTTAGGTGAAGCAGCATCC
OliveBaboon            TTGGAAGAAGATAATCAAGAAGAGCAAAGTGTGGATTCAAACTTAGGTGAAGCAGCATCC
Talapoin               TTGGAAGAAGATAATCAAGAAGAGCAAAGTGTGGATTCAAACTTAGGTGAAGCAGCATCC
WolfsGuenon            TTGGAAGAAGATAATCAAGAAGAGCAAAGTGTGGATTCAAACTTAGGTGAAGCAGCATCC
Colobus                TTGGAAGAAGATAATCCAGAAGAGCAAAGTGTGGATTCAAACTTAGGTGAAGCAGCATCC
Chimpanzee             TTGGAAGAAAATAATCAAGAAGAGCAAAGCATGGATTCAAACTTAGGTGAAGCAGCATCT
Bonobo                 TTGGAAGAAAATAATCAAGAAGAGCAAAGCATGGATTCAAACTTAGGTGAAGCAGCATCT
Gorilla                TTGGAAGAAAATAATCAAGAAGAGCAAAGCATGGATTCAAACTTAGGTGAAGCAGCATCT
Orangutan              TTGGAAGAAAATAATCAAGAAGAGCAAGGCGTGGATTCAAACTTAGGTGAAGCAGCATCT
BorneoOrangutan        TTGGAAGAAAATAATCAAGAAGAGCAAGGCGTGGATTCAAACTTAGGTGAAGCAGCATCT
PileatedGibbon         TTGGAAGAAAATAATCAAGAAGAGCAAAGCGTGGATTCAAACTTAGGTGAAGCAGCATCT
AgileGibbon            TTGGAAGAAAATAATCAAGAAGAGCAAAGCGTGGATTCAAACTTAGGTGAAGCAGCATCT
WHGibbon               TTGGAAGAAAATAATCAAGAAGAGCAAAGCGTGGATTCAAACTTAGGTGAAGCAGCATCT
Siamang                TTGGAAGAAAATAATCAAGAAGAGCAAAGCGTGGATTCAAACTTAGGTGAAGCAGCATCT
RCGibbon               TTGGAAGAAAATAATCAAGAAGAGCAAAGCGTGGATTCAAACTTAGGTGAAGCAGCATCT
WCGibbon               TTGGAAGAAAATAATCAAGAAGAGCAAAGCGTGGATTCAAACTTAGGTGAAGCAGCATCT
Marmoset               TTGGAAGAAGATAATCAAGAAGAGCAAAGTATGGATTCGAACTTAGGTGAAGCAGCATTT
SqMnky                 TTGGAAGAAGATAATCAAGAAGAGCAAAGTATGGATTCAAACTTAGGTGAAGCAGCATTT
TitiMonkey             TTGGAAGAAGATAATCAAGAAGAGCAAAGTGTGGATTCAAACTTAGGTGAAGCAGCAGTG
HowlerMk               TTGGAAGAAGATAATCAAGAAGAGCAAAGTATGGATTCAAACTTAGGTGAAGCAGCATCT
                       ********* ****** ********** *  ******* ******************

Human                  GGGTGTGAGAGTGAAACAAGCGTCTCTGAAGACTGCTCAGGGCTATCCTCTCAGAGTGAC
Rhesus                 GGGTATGAGAGTGAAACAAGCGTCTCTGAAGACTGCTCAAGGCTCTCCTCTCAGAGCGAA
CEMacaque              GGGTATGAGAGTGAAACAAGCGTCTCTGAAGACTGCTCAAGGCTCTCCTCTCAGAGCGAA
BMangabey              GGGTATGAGAGTGAAACAAGCGTCTCTGAAGACTGCTCAAGGCTCTCCTCTCAGAGCGAA
OliveBaboon            GGGTATGAGAGTGAAACAAGCGTCTCTGAAGACTGCTCAAGGCTCTCCTCTCAGAGCGAA
Talapoin               GGGTATGAGAGTGAAACAAGCGTCTCTGAAGACTGCTCAAGGCTCTCCTCTCAGAGCGAA
WolfsGuenon            GGGTATGAGAGTGAAACAAGCGTCTCTGAAGACTGCTCAAGGCTCTCCTCTCAGAGCGAA
Colobus                GGGTATGAGAGTGAAACAAGCGTCTCTGAAGACTGCTCAAGGCTCTCCTCTCAGAGCGAA
Chimpanzee             GGGTGTGAGAGTGAAACAAGCGTCTCTGAAGACTGCTCAGGGCTATCCTCTCAGAGTGAC
Bonobo                 GGGTGTGAGAGTGAAACAAGCGTCTCTGAAGACTGCTCAGGGCTATCCTCTCAGAGTGAC
Gorilla                GGGTGTGAGAGTGAAACAAGCGTCTCTGAAGACTGCTCAGGGCTATCCTCTCAGAGTGAC
Orangutan              GGGTATGAGAGTGAAACAAGCGTCTCTGAAGACTGCTCAGGGCTATCCTCTCAGAGTGAC
BorneoOrangutan        GGGTATGAGAGTGAAACAAGCGTCTCTGAAGACTGCTCAGGGCTATCCTCTCAGAGTGAC
PileatedGibbon         GGGTATGCAAGTGAAACAAGCGTCTCTGAAGACTGCTCAGGGCTATCCTCTCAGAGTGAC
AgileGibbon            GGGTATGCAAGTGAAACAAGCGTCTCTGAAGACTGCTCAGGGCTATCCTCTCAGAGTGAC
WHGibbon               GGGTATGCAAGTGAAACAAGCGTCTCTGAAGACTGCTCAGGGCTATCCTCTCAGAGTGAC
Siamang                GGGTATGCAAGTGAAACAAGCGTCTCTGAAGACTGCTCAGGGCTATCCTCTCAGAGTGAC
RCGibbon               GGGTATGCGAGTGAAACAAGCGTCTCTGAAGACTGCTCAGGGCTATCCTCTCAGAGTGAC
WCGibbon               GGGTATGCGAGTGAAACAAGCGTCTCTGAAGACTGCTCAGGGCTATCCTCTCAGAGTGAC
Marmoset               GGGTATGAGAGTGAAACAAGCATCACTGAAGACTGCTCAGGGCTATCCTCTCAGAGTGAT
SqMnky                 GGGTACGAGAGTGAAACAAGCATCACTGAAGACTGTTCAGGGCTATCCTCTCAGAGCGAT
TitiMonkey             GGGTATGAGAGTGAAACAAGCATCACTGAAGACTGCTCAGGGCTATCCTCTCAGAGCGAT
HowlerMk               GGGTATGAAAGTGAAACAAGCATCACTGAAGACTGCTCAGGGCTACCCTCTCAGAGCGAT
                       ****  *  ************ ** ********** *** ****  ********** **
```

```
Human              ATTTTAACCACTCAGCAGAGGGATACCATGCAACATAACCTGATAAAGCTCCAGCAGGAA
Rhesus             ATTTTAACCACTCAGCAGAGGGATACCATGCAAGATAACCTAATAAAGCTCCAGCAGGAA
CEMacaque          ATTTTAACCACTCAGCAGAGGGATACCATGCAAGATAACCTAATAAAGCTCCAGCAGGAA
BMangabey          ATTTTAACCACTCAGCAGAGGGATACCATGCAAGATAACCTAATAAAGCTCCAGCAGGAA
OliveBaboon        ATTTTAACCACTCAGCAGAGGGATACCATGCAAGATAACCTAATAAAGCTCCAGCAGGAA
Talapoin           ATTTTAACCACTCAGCAGAGGGATACTATGCAAGATAACCTAATAAAGCTCCAGCAGGAA
WolfsGuenon        ATTTTAACCACTCAGCAGAGGGATACCATGCAAGATAACCTAATAAAGCTCCAGCAGGAA
Colobus            ATTTTAACCACTCAGCAGAGGGATACCATGCAAGATAACCTAATAAAGCTCCAGCAGGAA
Chimpanzee         ATTTTAACCACTCAGCAGAGGGATACCATGCAAGATAACCTGATAAAGCTCCAGCAGGAA
Bonobo             ATTTTAACCACTCAGCAGAGGGATACCATGCAAGATAACCTGATAAAGCTCCAGCAGGAA
Gorilla            ATTTTAACCACTCAGCAGAGGGATACCATGCAAGATAACCTGATAAAGCTCCAGCAGGAA
Orangutan          ATTTTAACCACTCAGCAGAGGGATACCATGCAAGATAACCTGATAAAGCTCCAGCAGGAA
BorneoOrangutan    ATTTTAACCACTCAGCAGAGGGATACCATGCAAGATAACCTGATAAAGCTCCAGCAGGAA
PileatedGibbon     ATTTTAACCACTCAGCAGAGGGATACCATGCAAGATAACCTGATAAAGCTCCAGCAGGAA
AgileGibbon        ATTTTAACCACTCAGCAGAGGGATACCATGCAAGATAACCTGATAAAGCTCCAGCAGGAA
WHGibbon           ATTTTAACCACTCAGCAGAGGGATACCATGCAAGATAACCTGATAAAGCTCCAGCAGGAA
Siamang            ATTTTAACCACTCAGCAGAGGGATACCATGCAAGATAACCTGATAAAGCTCCAGCAGGAA
RCGibbon           ATTTTAACCACTCAGCAGAGGGATACCATGCAAGATAACCTGATAAAGCTCCAGCAGGAA
WCGibbon           ATTTTAACCACTCAGCAGAGGGATACCATGCAAGATAACCTGATAAAGCTCCAGCAGGAA
Marmoset           ATTTTAACCACTCAGCAGAGGGATACCATGCAAGACAATCTGATAAAGCTCCAGCAGGAA
SqMnky             ATTTTAACCACTCAGCAGAGGGATACCATGCAAGATAATCTGATAAAGCTCCAGCAGGAA
TitiMonkey         ATTTTAACCACTCAGCAGAGGGATACCATGCAAGATAATCTGATAAAGCTCCAGCAGGAA
HowlerMk           ATTTTAACCACTCAGCAGAGGGATACCATGCAAGATAATCTGATAAAGCTCCAGCAGGAA
                   ************************** ****** * ** ** ******************

Human              ATGGCTGAACTAGAAGCTGTGTTAGAACAGCATGGGAGCCAGCCTTCTAACAGCTACCCT
Rhesus             ATGGCTGAACTAGAAGCTGTGTTAGAACAGCACGGAAGCCAGCCTTCTAACAGCTACCCT
CEMacaque          ATGGCTGAACTAGAAGCTGTGTTAGAACAGCACGGAAGCCAGCCTTCTAACAGCTACCCT
BMangabey          ATGGCTGAACTAGAAGCTGTGTTAGAACAGCACGGAAGCCAGCCTTCTAACAGCTACCCT
OliveBaboon        ATGGCTGAACTAGAAGCTGTGTTAGAACAGCACGGAAGCCAGCCTTCTAACAGCTACCCT
Talapoin           ATGGCTGAACTAGAAGCTGTGTTAGAACAGCACGGAAGCCAGCCTTCTAACAGCTACCCT
WolfsGuenon        ATGGCTGAACTAGAAGCTGTGTTAGAACAGCACGGAAGCCAGCCTTCTAACAGCTACCCT
Colobus            ATGGCTGAACTAGAAGCTGTGTTAGAACAGCATGGAAGCCAGCCTTCTAACAGCTACCCT
Chimpanzee         ATGGCTGAACTAGAAGCTGTGTTAGAACAGCATGGGAGCCAGCCTTCTAACAGCTACCCT
Bonobo             ATGGCTGAACTAGAAGCTGTGTTAGAACAGCATGGGAGCCAGCCTTCTAACAGCTACCCT
Gorilla            ATGGCTGAACTAGAAGCTGTGTTAGAACAGCATGGGAGCCAGCCTTCTAACAGCTACCCT
Orangutan          ATGGCTGAACTAGAAGCTGTGTTAGAACAGCATGGGAGCCAGCCTTCTAACAGCTACCCT
BorneoOrangutan    ATGGCTGAACTAGAAGCTGTGTTAGAACAGCATGGGAGCCAGCCTTCTAACAGCTACCCT
PileatedGibbon     ATGGCTGAACTAGAAGCTGTGTTAGAACAGCATGGGAGCCAGCCTTCTCACAGCTACCCT
AgileGibbon        ATGGCTGAACTAGAAGCTGTGTTAGAACAGCATGGGAGCCAGCCTTCTCACAGCTACCCT
WHGibbon           ATGGCTGAACTAGAAGCTGTGTTAGAACAGCATGGGAGCCAGCCTTCTCACAGCTACCCT
Siamang            ATGGCTGAACTAGAAGCTGTGTTAGAACAGCATGGGAGCCAGCCTTCTCACAGCTACCCT
RCGibbon           ATGGCTGAACTAGAAGCTGTGTTAGAACAGCATGGGAGCCAGCCTTCTCACAGCTACCCT
WCGibbon           ATGGCTGAACTAGAAGCTGTGTTAGAACAGCATGGGAGCCAGCCTTCTCACAGCTACCCT
Marmoset           ATGGCTGAACTAGAAGCTGTGTTAGAACAGCATGGGAGCCAGCCTTCTCACAGCTCCCCT
SqMnky             ATGGCTGAACTAGAAGCTGTGTTAGAACAGCATGGGAGCCAGCCTTCTCACAGCTCCCCT
TitiMonkey         ATGGCTGAACTAGAAGCTGTGTTAGAACAGCATGGGAGCCAGCCTTCTCACAGCTCCCCT
HowlerMk           ATGGCTGAACTAGAAGCTGTGTTAGAACAGCATGGGAGCCAGCCTTCTCACAGTTCCCCT
                   ******************************** ** ************ **** * ****

Human              TCCATCATAAGTGACTCTTCTGCCCTTGAGGACCTGCGAAATCCAGAACAAAGCACATCA
Rhesus             TCCATCATAACTGACTCTTCTGCCCTTGAGGACCTGCGAAATCCAGAACAAAGCACATCA
CEMacaque          TCCATCATAACTGACTCTTCTGCCCTTGAGGACCTGCGAAATCCAGAACAAAGCACATCA
BMangabey          TCCATCATAACTGACTCTTCTGCCCTTGAGGACCTGCGAAATCCAGAACAAAGCACATCA
OliveBaboon        TCCATCATAACTGACTCTTCTGCCCTTGAGGACCTGCGAAATCCAGAACAAAGCACATCA
Talapoin           TCCATCATAACTGACTCTTCTGCCCTTGAGGACCTGCGAAATCCAGAACAAAGCACATCA
WolfsGuenon        TCCATCATAACTGACTCTTCTGCCCTTGAGGACCTGCGAAATCCAGAACAAAGCACATCA
Colobus            TCCATTGTAACTGACTCTTCTGCCCTTGAGGACCTACGAAATCCAGAACAAAGCACATCA
Chimpanzee         TCCATCATAAGTGACTCTTCTGCCCTTGAGGACCTGCAAAATCCAGAACAAAGCACATCA
Bonobo             TCCATCATAAGTGACTCTTCTGCCCTTGAGGACCTGCAAAATCCAGAACAAAGCACATCA
Gorilla            TCCATCATAAGTGACTCTTCTGCCCTTGAGGACCTGCGAAATCCAGAACAAAGCACATCA
Orangutan          TCCATCATAAGTGACTCTTCTGCCCTTGAGGACCTGCGAAATCCAGAACAAAGCACATCA
BorneoOrangutan    TCCATCATAAGTGACTCTTCTGCCCTTGAGGACCTGCGAAATCCAGAACAAAGCACATCA
PileatedGibbon     CCCATCATAAGTGACTCTTCTGCCCTTGAGGACCTGCAAAATCCAGAACAAAGCACATCA
AgileGibbon        CCCATCATAAGTGACTCTTCTGCCCTTGAGGACCTGCAAAATCCAGAACAAAGCACATCA
WHGibbon           CCCATCATAAGTGACTCTTCTGCCCTTGAGGACCTGCAAAATCCAGAACAAAGCACATCA
Siamang            CCCATCATAAGTGACTCTTCTGCCCTTGAGGACCTGCAAAATCCAGAACAAAGCACATCA
RCGibbon           TCCATCATAAGTGACTCTTCTGCCCTTGAGGACCTGCAAAATCCAGAACAAAGCACATCA
WCGibbon           TCCATCATAAGTGACTCTTCTGCCCTTGAGGACCTGCAAAATCCAGAACAAAGCACATCA
Marmoset           TCCGTCATAACTGTCTCTTCTGCCCCTGAGGACCTTCAAAATCCAGAACAAAGCACATCA
SqMnky             TCCGTCATAACTGACTCTTCTGCCCTTGAGGACCTTCGAAATCCAGAACAAAGCACATCA
TitiMonkey         TCTGTCATAACTGACGCTTCTGTCCTTGAGGACCTTCAAAACCCAGAACAAAGCACATCA
```

```
HowlerMk                 TCCATCATAACTGACTCTTCTACCTTTGAGTACCTTCGAAATCCGAAACAAAGCACATCA
                          *  *  *** ** * *****  *  **** **** * *** **  **************

Human                    GAAAAAGCAGTATTAACTTCACAGAAAAGTAGTGAATACCCTATAAGCCAGAATCCAGAA
Rhesus                   GAAAAAGCAGTATTAACTTCACAGAAAAGTAGTGAGTATCCTATAAACCAGAATCCAGAA
CEMacaque                GAAAAAGCAGTATTAACTTCACAGAAAAGTAGTGAGTATCCTATAAACCAGAATCCAGAA
BMangabey                GAAAAAGCAGTATTAACTTCACAGAAAAGTAGTGAATATCCTATAAACCAGAATCCAGAA
OliveBaboon              GAAAAAGCAGTATTAACTTCACAGAAAAGTAGTGAATATCCTATAAACCAGAATCCAGAA
Talapoin                 GAAAAAGCAGTATTAACTTCACAGAAAAGTAGTGAATATCCTATAAACCAGAATCCAGAA
WolfsGuenon              GAAAAAGCAGTATTAACTTCACAGAAAAGTAGTGAATATCCTATAAACCAGAATCCAGAA
Colobus                  GAAAAAGCAGTATTAACTTCACAGAAAAGTAGTGAATATCCTATAAGCCAGAATCCAGAA
Chimpanzee               GAAAAAGCAGTATTAACTTCACAGAAAAGTAGTGAATACCCTATAAGCCAGAATCCAGAA
Bonobo                   GAAAAAGCAGTATTAACTTCACAGAAAAGTAGTGAATACCCTATAAGCCAGAATCCAGAA
Gorilla                  GAAAAAGCAGTATTAACTTCACAGAAAAGTAGTGAATACCCTATAAGCCAGAATCCAGAA
Orangutan                GAAAAAGCAGTATTAACTTCACAGAAAAGTAGTGAATACCCTATAAGCCAGAATCCAGAA
BorneoOrangutan          GAAAAAGCAGTATTAACTTCACAGAAAAGTAGTGAATACCCTATAAGCCAGAATCCAGAA
PileatedGibbon           GAAAAAGCAGTATTAACTTCACAGAAAAGTAGTGCATACCCTATAAGCCAGAATCCAGAA
AgileGibbon              GAAAAAGCAGTATTAACTTCACAGAAAAGTAGTGCATACCCTATAAGCCAGAATCCAGAA
WHGibbon                 GAAAAAGCAGTATTAACTTCACAGAAAAGTAGTGCATACCCTATAAGCCAGAATCCAGAA
Siamang                  GAAAAAGCAGTATTAACTTCACAGAAAAGTAGTGCATACCCTATAAGCCAGAATCCAGAA
RCGibbon                 GAAAAAGCAGTATTAACTTCACAGAAAAGTAGTGCATACCCTATAAGCCAGAATCCAGAA
WCGibbon                 GAAAAAGCAGTATTAACTTCACAGAAAAGTAGTGCATACCCTATAAGCCAGAATCCAGAA
Marmoset                 GAAAAAGCAGTATTAACTTCACAGAAAAGTAGTGAATATCCTATAAGCCAGAATCCAGAA
SqMnky                   GAAAAAGCAGTATTAACTTCACAGAAAAATAGTGAATATCGTATAAGCCAGAATCCAGAA
TitiMonkey               GAAAAAGCAGTATTAACTTCACAGAAAAGTAGTGAATATCCTATAAGCCAGATTCCAGAA
HowlerMk                 GAAAAAGCAGTATTAACTTCACAGAAAAGTAGTGAATATCCTATAAGCCAGAATCCAGAA
                         **************************** *****  ** * ***** ***** *******

Human                    GGCCTTTCTGCTGACAAGTTTGAGGTGTCTGCAGATAGTTCTACCAGTAAAAATAAAGAA
Rhesus                   GGCCTTTCTGCTGACAAGTTCGAGGTATCTGCAGATAGTTCTACCAGTAAAAATAAAGAA
CEMacaque                GGCCTTTCTGCTGACAAGTTCGAGGTATCTGCAGATAGTTCTACCAGTAAAAATAAAGAA
BMangabey                GGCCTTTCTGCTGACAAGTTCGAGGTATCTGCAGATAGTTCTACCAGTAAAAATAAAGAA
OliveBaboon              GGCCTTTCTGCTGACAAGTTCGAGGTATCTGCAGATAGTTCTACCAGTAAAAATAAAGAA
Talapoin                 GGCCTTTCTGCTGACAAGTTCGAGGTATCTGCAGATAGTTCTACCAGTAAAAATAAAGAA
WolfsGuenon              GGCCTTTCTGCTGACAAGTTCGAGGTATCTGCAGATAGTTCTACCAGTAAAAATAAAGAA
Colobus                  GGCCTTTCTGCTGACAAGTTCAAGGTATCTGCAGATAGTTCTACCAGTAAAAATAAAGAA
Chimpanzee               GGCCTTTCTGCTGACAAGTTTGAGGTGTCTGCAGATAGTTCTACCAGTAAAAATAAAGAA
Bonobo                   GGCCTTTCTGCTGACAAGTTTGAGGTGTCTGCAGATAGTTCTACCAGTAAAAATAAAGAA
Gorilla                  GGCCTTTCTGCTGACAAGTTTGAGGTGTCTGCAGATAGTTCTACCAGTAAAAATAAAGAA
Orangutan                GGCCTTTCTGCTGACAAGTTTGAGGTGTCTGCAGATAGTTCAACCAATAAAAATAAAGAA
BorneoOrangutan          GGCCTTTCTGCTGACAAGTTTGAGGTGTCTGCAGATAGTTCAACCAATAAAAATAAAGAA
PileatedGibbon           GGCCTTTTTGCTGACAAGTTTGAGGTGTCTGCAGATAGTTCTACCAGTAAAAATAAAGAA
AgileGibbon              GGCCTTTTTGCTGACAAGTTTGAGGTGTCTGCAGATAGTTCTACCAGTAAAAATAAAGAA
WHGibbon                 GGCCTTTTTGCTGACAAGTTTGAGGTGTCTGCAGATAGTTCTACCAGTAAAAATAAAGAA
Siamang                  GGCCTTTTTGCTGACAAGTTTGAGGTGTCTGCAGATAGTTCTACCAGTAAAAATAAAGAA
RCGibbon                 GGCCTTTTTGCTGACAAGTTTGAGGTGTCTGCAGATAGTTCTACCAGTAAAAATAAAGAA
WCGibbon                 GGCCTTTTTGCTGACAAGTTTGAGGTGTCTGCAGATAGTTCTACCAGTAAAAATAAAGAA
Marmoset                 GGCCTTTCTGCTGACAAGTTTGAGGAGTCTTCAGATAGTTCTACCAATAAAAATAAAGAA
SqMnky                   GGCCTTTCTGCTGACAAGCTTGAGGTGTCTCCAGATAGTTCTACCAGTAAAAATAAAGAA
TitiMonkey               TGCCTTTCTGCTGACAAGTTCAAGGTGTCTCCAGATAGTTCTACCAGTAAAAATAAAGAA
HowlerMk                 GGCCTTTCTGCTGACAAGTTCGAGATGTCTCCAGATAGTTCTACCAGTAAAAATGAAGAA
                          ****** ********** *  **   *** ********** **** ******* *****

Human                    CCAGGAGTGGAAAGGTCATCCCCTTCTAAATGCCCATCATTAGATGATAGGTGGTACATG
Rhesus                   CCAGGAGTGGAAAGGTCATCCCCTTCTAAATGCCAGTCATTAGAGGATAGGTGGTATGTG
CEMacaque                CCAGGAGTGGAAAGGTCATCCCCTTCTAAATGCCAGTCATTAGAGGATAGGTGGTATGTG
BMangabey                CCAGGAGTGGAAAGGTCATCCCCTTCTAAATGCCAGTCATTAGAGGATAGGTGGTATGTG
OliveBaboon              CCAGGAGTGGAAAGGTCATCCCCTTCTAAATGCCAGTCATTAGAGGATAGGTGGTATGTG
Talapoin                 CCAGGAGTGGAAAGGTCATCCCCTTCTAAATGCCAGTCATTAGAGGATAGGTGGTATGTG
WolfsGuenon              CCAGGAGTGGAAAGGTCATCCCCTTCTAAATGCCAGTCATTAGAGGATAGGTGGTATGTG
Colobus                  CCAGGAGTGGAAAGGTCATCCCCTTCTAAATGCCAGTCGTTAGAGGATAGGTGGTATGTG
Chimpanzee               CCAGGAGTGGAAAGGTCATCCCCTTCTAAATGCCCATCATTAGATGATAGGTGGTACATG
Bonobo                   CCAGGAGTGGAAAGGTCATCCCCTTCTAAATGCCCATCATTAGATGATAGGTGGTACATG
Gorilla                  CCAGGAGTGGAAAGGTCATCCCCTTCTAAATGCCCATCATTAGATGATAGGTGGTACATG
Orangutan                CCAGGAGTGGAAAGGTCATCCCCTTCTAAATGCCCGTCATTAGATGATAGGTGGTACATG
BorneoOrangutan          CCAGGAGTGGAAAGGTCATCCCCTTCTAAATGCCCGTCATTAGATGATAGGTGGTACCTG
PileatedGibbon           CCAGGAGTGGAAAGGTCATCCCCTTCTAAATGCCAGTCATTAGATGATAGGTGGTACATG
AgileGibbon              CCAGGAGTGGAAAGGTCATCCCCTTCTAAATGCCAGTCATTAGATGATAGGTGGTACATG
WHGibbon                 CCAGGAGTGGAAAGGTCATCCCCTTCTAAATGCCAGTCATTAGATGATAGGTGGTACATG
Siamang                  CCAGGAGTGGAAAGGTCATCCCCTTCTAAATGCCAGTCATTAGATGATAGGTGGTACATG
RCGibbon                 CCAGGAGTGGAAAGGTCATCCCCTTCTAAATGCCAGTCATTAGATGATAGGTGGTACATG
WCGibbon                 CCAGGAGTGGAAAGGTCATCCCCTTCTAAATGCCAGTCATTAGATGATAGGTGGTACATG
Marmoset                 CCAGGAGTGGAAAGGTCATCCCCTTCTAAATGCCAGTCATTAGATGATAGGTGGTGCGTG
```

```
SqMnky                 CCAGGAGTGGAAAGGTCATCCCCTTCCAAATGCCAGTCATTAGATGATAGGTGGTGCATG
TitiMonkey             CCAGGAGTGGAAAGGTCATCCCCTTCTAAATGCCAGTCATTAGATGATAGGTGGTGCGTG
HowlerMk               CCAGGAGTGGAAAGATCATCCCCTTCTAAATGCCAGCCATTAGATGATAGGTGGTGCGTG
                       ************** *********** *******   * ***** **********   **

Human                  CACAGTTGCTCTGGGAGTCTTCAGAATAGAAACTACCCATCTCAAGAGGAGCTCATTAAG
Rhesus                 CACAGTAGCTCTGGGAGTCTTCAGAATGGAAATTACCCATCTCAAGAGGAGCTCATTAAG
CEMacaque              CACAGTAGCTCTGGGAGTCTTCAGAATGGAAATTACCCATCTCAAGAGGAGCTCATTAAG
BMangabey              CACAGTAGCTCTGGGAGTCTTCAGAATGGAAATTACCCATCTCAAGAGGAGCTCATTAAG
OliveBaboon            CACAGTAGCTCTGGGAGTCTTCAGAATGGAAATTACCCATCTCAAGAGGAGCTCATTAAG
Talapoin               CACAGAAGCTCTGGGAGTCTTCAGAATAGAAATTACCCATCTCAAGAGGAGCTCATTAAG
WolfsGuenon            CACAGTAGCTCTGGGAGTCTTCAGAATGGAAATTACCCATCTCAAGAGGAGCTCATTAAG
Colobus                CACAGTAGCTCTGGGAGTCTTCAGAATGGAAACTACCCATCTCAAGAGGAGCTCATTAAG
Chimpanzee             CACAGTTGCTCTGGGAGTCTTCAGAATAGAAACTACCCATCTCAAGAGGAGCTCATTAAG
Bonobo                 CACAGTTGCTCTGGGAGTCTTCAGAATAGAAACTACCCATCTCAAGAGGAGCTCATTAAG
Gorilla                CACAGTTGCTCTGGGAGTCTTCAGAATAGAAACTACCCATCTCAAGAGGAGCTCATTAAG
Orangutan              CACAGTTGCTCTGGGAGTCTTCAGAATGGAAACTACCCATCTCAAGAGGAGCTCATTAAG
BorneoOrangutan        CACAGTTGCTCTGGGAGTCTTCAGAATGGAAACTACCCATCTCAAGAGGAGCTCATTAAG
PileatedGibbon         CACAGTTGCTCTGGGAGTCTTCAGAATGGAAACTACCCATCTCAAGAGGAGCTCATTAAG
AgileGibbon            CACAGTTGCTCTGGGAGTCTTCAGAATGGAAACTACCCATCTCAAGAGGAGCTCATTAAG
WHGibbon               CACAGTTGCTCTGGGAGTCTTCAGAATGGAAACTACCCATCTCAAGAGGAGCTCATTAAG
Siamang                CACAGTTGCTCTGGGAGTCTTCAGAATGGAAACTACCCATCTCAAGAGGAGCTCATTAAG
RCGibbon               CACAGTTGCTCTGGGAGTCTTCAGAATGGAAACTACCCATCTCAAGAGGAGCTCATTAAG
WCGibbon               CACAGTTGCTCTGGGAGTCTTCAGAATGGAAACTACCCATCTCAAGAGGAGCTCATTAAG
Marmoset               CACAGTTGCTCTGGGAGTCTTCAGAATGGAAACTCCCATCTCAAGAGGAGCTCATTAAG
SqMnky                 CACAGTTGCTCTGGGAGTCTTCAGAATGGAAACTACCCATCTCAAGAGGAGCTTATTAAG
TitiMonkey             CACGGTTGCTCTGGGAGTCTTCTGAATGGAAACTACCCATCTCAAGAGGAGCTCATTAAG
HowlerMk               CACAGTTGCTCTGGGAGTCTTCAGAATGGAAACTACCCATCTCAAGAGGAGCTCATTAAG
                       *** *  *************** **** **** * ****************** ******

Human                  GTTGTTGATGTGGAGGAGCAACAGCTGGAAGAGTCTGGGCCACACGATTTGACGGAAACA
Rhesus                 GTTGTTGATGTGGAGACACAACAACTGGAAAAGTCTGGGCCACACGATTTGATGGAACCA
CEMacaque              GTTGTTGATGTGGAGACGCAACAACTGGAAAAGTCTGGGCCACACGATTTGATGGAACCA
BMangabey              GTTGTTGATGTGGAGACGCAACAACTGGAAAAGTCTGGGCCACACAATTTGATGGAACCA
OliveBaboon            GTTGTTGATGTGGAGACGCAACAACTGGAAAAGTCTGGGCCACACAATTTGATGGAACCA
Talapoin               GTTGTTGATGTGGAGAAGCAACGACTGGAAAAGTCTGGGCCACACGATTTGATGGAACCA
WolfsGuenon            GTTGTTGATGTGGAGAAGCAACAACTGGAAAAGTCTGGGCCACACGATTTGATGGAACCA
Colobus                GTTGTTGATATGGAGGAGCAACAACTGGAAAAGTCTGGGCCACACAATTTGATGGAACCA
Chimpanzee             GTTGTTGATGTGGAGGAGCAACAGCTGGAAGAGTCTGGGCCACACGATTTGACGGAAACA
Bonobo                 GTTGTTGATGTGGAGGAGCAACAGCTGGAAGAGTCTGGGCCACACGATTTGACGGAAACA
Gorilla                GTTGTTGATGTGGAGGAGCAACAGCTGGAAGAGTCTGGGCCACACGATTTGACGGAAACA
Orangutan              GTTGTTGATGTGGAGAAGCAACAGCTGGAAGAGTCTGGGCCACACGATTTGACGGAACCA
BorneoOrangutan        GTTGTTGATGTGGAGAAGCAACAGCTGGAAGAGTCTGGGCCACACGATTTGACGGAACCA
PileatedGibbon         GTTGTTGATGTGGAGGAGCAACAGCTGGAAGAGTCTGGGCCACACGATTTGACGGAACCA
AgileGibbon            GTTGTTGATGTGGAGGAGCAACAGCTGGAAGAGTCTGGGCCACACGATTTGACGGAACCA
WHGibbon               GTTGTTGATGTGGAGGAGCAACAGCTGGAAGAGTCTGGGCCACACGATTTGACGGAACCA
Siamang                GTTGTTGATGTGGAGGAGCAACAGCTGGAAGAGTCTGGGCCACACGATTTGACGGAACCA
RCGibbon               GTTGTTGATGTGGAGGAGCAACAGCTGGAAGAGTCTGGGCCACACGATTTGACGGAACCA
WCGibbon               GTTGTTGATGTGGAGGAGCAACAGCTGGAAGAGTCTGGGCCACACGATTTGACGGAACCA
Marmoset               GTT---GATGTGGAGGAGCAACAACTGGATAAGTCTGGGCCCCATGATTTGATGGAACCA
SqMnky                 GTT---GATATGGAGGAGCAACAACTGGAAAAGTCTGGGCCACATGATTTAATGGAACCA
TitiMonkey             GTT---GATGTGGAGGAGCAACAACTGGAAAAGTCTGGGCCACATGATTTAATGAAACCA
HowlerMk               GTT---GATGTGGAGGAGCAACAACTGGAAAAGTTTGAGCCACATGATTTAATGGAACCA
                       ***   *** *****   ****  *****  *** ** *** **  **** * * ** **

Human                  TCTTACTTGCCAAGGCAAGATCTAGAGGGAACCCCTTACCTGGAATCTGGAATCAGCCTC
Rhesus                 TCTTACTTGCCAAGGCAAGATCTAGACGGAACCCCTTACCTGGAATCTGGAATCAGTCTC
CEMacaque              TCTTACTTGCCAAGGCAAGATCTAGAGGGAACCCCTTACCTGGAATCTGGAATCAGTCTC
BMangabey              TCTTACTTGCCAAGGCAAGATCTAGAGGGAACCCCTTACCTGGAATCTGGAATCAGTCTC
OliveBaboon            TCTTACTTGCCAAGGCAAGATCTAGAGGGAACCCCTTACCTGGAATCTGGAATCAGTCTC
Talapoin               TCTTACTTGCCAAGGCAAGATCTAGAGGGAACCCCTTACCTGGAATCTGGAATCAGTCTC
WolfsGuenon            TCTTACTTGCCAAGGCAAGATCTAGAGGGAACCCCTTACCTAGAATCTGGAATCAGTCTC
Colobus                TCTTACTTGCCAAGGCAAGATCTAGAGGGAACACCTTACCTGGAATCTGGAATCAGTCTC
Chimpanzee             TCTTACTTGCCAAGGCAAGATCTAGAGGGAACCCCTTACCTGGAATCTGGAATCAGCCTC
Bonobo                 TCTTACTTGCCAAGGCAAGATCTAGAGGGAACCCCTTACCTGGAATCTGGAATCAGCCTC
Gorilla                TCTTACTTGCCAAGGCAAGATCTAGAGGGAACCCCTTACCTGGAATCTGGAATCAGCCTC
Orangutan              TCTTACTTGCCAAGGCAAGATCTAGAGGGAACCCCTTACCTGGAATCTGGAATCAGCCTC
BorneoOrangutan        TCTTACTTGCCAAGGCAAGATCTAGAGGGAACCCCTTACCTGGAATCTGGAATCAGCCTC
PileatedGibbon         TCTTACTTGCCAAGGCAAGATCTAGAGGGAACCCCTTACTTGGAATCTGGAATCAGCCTC
AgileGibbon            TCTTACTTGCCAAGGCAAGATCTAGAGGGAACCCCTTACTTGGAATCTGGAATCAGCCTC
WHGibbon               TCTTACTTGCCAAGGCAAGATCTAGAGGGAACCCCTTACTTGGAATCTGGAATCAGCCTC
Siamang                TCTTACTTGCCAAGGCAAGATCTAGAGGGAACCCCTTACTTGGAATCTGGAATCAGCCTC
RCGibbon               TCTTACTTGCCAAGGCAAGATCTAGAGGGAACCCCTTACTTGGAATCTGGAATCAGCCTC
```

```
WCGibbon             TCTTACTTGCCAAGGCAAGATCTAGAGGGAACCCCTTACTTGGAATCTGGAATCAGCCTC
Marmoset             TCTTACTTGCCAAGGCAAGATCTAGAGGGAACCCCTTACCTAGAATCTGGAATCAGCCTC
SqMnky               TCTTACTTGCCAAGGCACGATCTAGAGGGAACCCCTTACCTAGAATCTGGAATCAGCCTC
TitiMonkey           CCTTACTTGCCAAGGCAAGATCTAGAGGGAACCCCTTACCTAGAATCTGGAATCAGCCTC
HowlerMk             TCTTACTTGCCAAGGCAAAATCTAGAGGGAACCCCTTACCTAGAATCTGGAATCAGCCTC
                      ****************  ******* ***** ****** * ************** ***

Human                TTCTCTGATGACCCTGAATCTGATCCTTCTGAAGACAGAGCCCCAGAGTCAGCTCGTGTT
Rhesus               TTCTCTGATGACCCTGAATCTGATCCTTCCGAAGACAGAGCCCCAGAGTCAGCTCATGTT
CEMacaque            TTCTCTGATGACCCTGAATCTGATCCTTCCGAAGACAGAGCCCCAGAGTCAGCTCATGTT
BMangabey            TTCTCTGATGACCCTGAATCTGATCCGTCCGAAGACAGAGCCCCAGAGTCAGCTCATGTT
OliveBaboon          TTCTCTGATGACCCTGAATCTGATCCATCCGAAGACAGAGCCCCAGAGTCAGCTCATGTT
Talapoin             TTCTCTGATGACCCTGAATCTGATCCTTCTGAAGACAGAGCCCCAGAGTCAGCTCATGTT
WolfsGuenon          TTCTCTGATGACCCTGAATCTGATCCTTCTGAAGACAGAGCCCCAGAGTCAGCTCATGTT
Colobus              TTCTCTGATGACCCTGAATCTGATCCTTCTGAAGACAGAGCCCTAGAGTCAGCTCATGTT
Chimpanzee           TTCTCTGATGACCCTGAATCTGATCCTTCTGAAGACAAAGCCCCAGAGTCAGCTCATGTT
Bonobo               TTCTCTGATGACCCTGAATCTGATCCTTCTGAAGACAAAGCCCCAGAGTCAGCTCATGTT
Gorilla              TTCTCTGATGACCCTGAATCTGATCCTTCTGAAGACAGAGCCCCAGAGTCAGCTCATGTT
Orangutan            TTCTCTGATGACCCTGAATCTGATCCTTCTGAAGACAGAGCCCCAGAGTCAGCTCATGTT
BorneoOrangutan      TTCTCTGATGACCCTGAATCTGATCCTTCTGAAGACAGAGCCCCAGAGTCAGCTCATGTT
PileatedGibbon       TTCTCTGATGACTCTGAATCTGATCCTTCGGCAGACAGAGCCCCAGAGTCAGCTCATGTT
AgileGibbon          TTCTCTGATGACTCTGAATCTGATCCTTCGGCAGACAGAGCCCCAGAGTCAGCTCATGTT
WHGibbon             TTCTCTGATGACTCTGAATCTGATCCTTCGGCAGACAGAGCCCCAGAGTCAGCTCATGTT
Siamang              TTCTCTGATGACTCTGAATCTGATCCTTCGGCAGACAGAGCCCCAGAGTCAGCTCATGTT
RCGibbon             TTCTCTGATGACTCTGAATCTGATCCTTCGGCAGACAGAGCCCCAGAGTCAGCTCATGTT
WCGibbon             TTCTCTGATGACTCTGAATCTGATCCTTCGGCAGACAGAGCCCCAGAGTCAGCTCATGTT
Marmoset             TTCTCTGATGACCCTGAATCTGATCCTTCTGAAGACAGAGCCCCAGAATCATCCCATGCT
SqMnky               TTCTCTGATGACCCTGAATCTGATCCTTCTGAAGACAGAGCCCCAGAATCATCTCATGCT
TitiMonkey           TTCTCTGATGACCCTGAATCTGATCCTTCTGAAGACAGAGCCCCAGAATCATCTCATGCT
HowlerMk             TTCTCTGATGACCCTGAATCTGATCCTTCTGAAGACAGAGCCCCAGAATCATCTCATGCT
                     ************ ************* ** * ***** ***** *** *** * * ** *

Human                GGCAACATACCATCTTCAACCTCTGCATTGAAAGTTCCCCAATTGAAAGTTGCAGAATCT
Rhesus               GGCAGCATACCATCTTCAACCTCTGCATTGAAAGTACCCCAGTGGCAAGTTGCAGAATCT
CEMacaque            GGCAGCATACCATCTTCAACCTCTGCATTGAAAGTACCCCAGTGGCAAGTTGCAGAATCT
BMangabey            GGCAGCATACCATCTTCAACCTCTGCATTGAAAGTACCCCAGTGGCAAGTTGCAGAATCT
OliveBaboon          GGCAGCATACCATCTTCAACCTCTGCATTGAAAGTACCCCAGTGGCAAGTTGCAGAATCT
Talapoin             GGCAGCATACCATCTTCAACCTCTGCATTGAAAGTACCCCAGTGGCAAGTTGCAGAATCT
WolfsGuenon          GGCAGCATACCATCTTCAACCTCTGCATTGAAAGTACCCCAGTGGCAAGTTGCAGAATCT
Colobus              GGCAGCATACCATCTTCAACCTCTGCATTGAAAGTACCCCAGTGGCAAGTTGCAAAATCT
Chimpanzee           GGCAACATACCATCTTCAACCTCTGCATTGAAAGTTCCCCAATTGAAAGTTGCAGAATCT
Bonobo               GGCAACATACCATCTTCAACCTCTGCATTGAAAGTTCCCCAATTGAAAGTTGCAGAATCT
Gorilla              GGCAACATACCATCTTCAACCTCTGCATTGAAAGTTCCCCAATTGAAAGTTGCAGAATCT
Orangutan            GGCAGCATACCATCTTCAACCTCTGCATTGAAAGTACCCCAATTGAAAGTTGCAGAATCT
BorneoOrangutan      GGCAGCATACCATCTTCAACCTCTGCATTGAAAGTACCCCAATTGAAAGTTGCAGAATCT
PileatedGibbon       GGCAATATACCATCTTCAACCTCTGCATTGAAAGTACCCCAGTTGAAAGTTGCTGAATCT
AgileGibbon          GGCAATATACCATCTTCAACCTCTGCATTGAAAGTACCCCAGTTGAAAGTTGCTGAATCT
WHGibbon             GGCAATATACCATCTTCAACCTCTGCATTGAAAGTACCCCAGTTGAAAGTTGCTGAATCT
Siamang              GGCAATATACCATCTTCAACCTCTGCATTGAAAGTACCCCAGTTGAAAGTTGCTGAATCT
RCGibbon             GGCAATATACCATCTTCAACCTCTGCATTGCAAGTACCCCAGTTGAAAGTTGCTGAATCT
WCGibbon             GGCAATATACCATCTTCAACCTCTGCATTGAAAGTACCCCAGTTGAAAGTTGCTGAATCT
Marmoset             GGCAGCATACCATCTTCAACCTCTACATTGAAAGTACCCCAGTTTCAAGTTGCAGAATCT
SqMnky               GGCAGCATACCATCTTCAACCTCTGCATTGAAAGTACCCCAGTTTCAAGTTGCAGAATGT
TitiMonkey           GGCAGCATACCATCTTCAACCTCTATATTGAAAGTACCCCAATTTCAAGTTGCAGAATCT
HowlerMk             GGCAGCATACCATCTTCAACTTCTACATTGAAAGTACCCCAATTTCAAGTTGCAGAATCT
                     ****  ************** ***  **** **** ***** *   *******  *** *

Human                GCCCAGAGTCCAGCTGCTGCTCATACTACTGATACTGCTGGGTATAATGCAATGGAAGAA
Rhesus               GCCCAGAGTCCAGCTGCTGCTCATAATACTAATACTGCTGGGTATAATGCAATGGAAGAA
CEMacaque            GCCCAGAGTCCAGCTGCTGCTCATAATACTAATACTGCTGGGTATAATGCAATGGAAGAA
BMangabey            GCCCAGAGTCCAGCTGCTGCTCATAATACTAATACTGCTGGGTATAATGCAATGGAAGAA
OliveBaboon          GCCCAGAGTCCAGCTGCTGCTCATAATACTAATACTGCTGGGTATAATGCAATGGAAGAA
Talapoin             GCCCAGAGTCCAGCTGCTGCTCATAATACTAATACTGCTGGGTATAATGCAATGGAAGAA
WolfsGuenon          GCCCAGAGTCCAGCTGCTGCTCATAATACTAATACTGCTGGGTATAATGCAATGGAAGAA
Colobus              GCCCAGAGTCCAGCTGCTGCTCATAATACTAATACTGCTGAGTATAATGCAATGGAAGAA
Chimpanzee           GCCCAGAGTCCAGCTGCTGCTCATACTACTAATACTGCTGGGTATAATGCAATGGAAGAA
Bonobo               GCCCAGAGTCCAGCTGCTGCTCATACTACTAATACTGCTGGGTATAATGCAATGGAAGAA
Gorilla              GCCCAGAGTCCAGCTGCTGCTCATACTACTAATACTGCTGGGTATCATGCAATGGAAGAA
Orangutan            GCCCAGAGTCCAGCTGCTGCTCAGACTACTAATACTGCTGGGTATAATGCGATGGAAGAA
BorneoOrangutan      GCCCAGAGTCCAGCTGCTCCTCAGACTACTAATACTGCTGGGTATAATGCGATGGAAGAA
PileatedGibbon       GCCCAGAGTCCAGCTGCTGCTCATACTACTAATACTGCTGGGTATAATGCAATGGAAGAA
AgileGibbon          GCCCAGAGTCCAGCTGCTGCTCATACTACTAATACTGCTGGGTATAATGCAATGGAAGAA
WHGibbon             GCCCAGAGTCCAGCTGCTGCTCATACTACTAATACTGCTGGGTATAATGCAATGGAAGAA
```

```
Siamang             GCCCAGAGTCCAGCTGCTGCTCATACTACTAATACTGCTGGGTATAATGCAATGGAAGAA
RCGibbon            GCCCAGAGTCCAGCTGCTGCTCATACTACTAATACTGCTGGGTATAATGCAATGGAAGAA
WCGibbon            GCCCAGAGTCCAGCTGCTGCTCATACTACTAATACTGCTGGGTATAATGCAATGGAAGAA
Marmoset            GCCAAGAGTCTAGCTGCTGCTCATACTACTAATACTGCTGGGTGTAATGCAATGGAAGAA
SqMnky              GCCAAGAGTCTAGCTGCTGCTCATACTACTAATACTGCTGGGTGTAATGCAGTGGATGAA
TitiMonkey          GCCAAGAGTCTAGCTGCTGCTCATATTACTAATACTGCTAGGTGTAATGCAATGGAAGAA
HowlerMk            GCCAAGAGTCTAGCTGCTGCTCATAATACTAATACTGCTGGGTGTAATGCAATGGAAGAA
                    *** ****** ******* **** * **** ********  ** * ****  **** ***

Human               AGTGTGAGCAGGGAGAAGCCAGAATTGACAGCTTCAACAGAAAGGGTCAACAAAAGAATG
Rhesus              AGTGTGAGCAGGGAGAATCCGAAATTGACAGCTTCAACAGAAAGGGTCAACAAAAGAATG
CEMacaque           AGTGTGAGCAGGGAGAATCCGAAATTGACAGCTTCAACAGAAAGGGTCAACAAAAGAATG
BMangabey           AGTGTGAGCAGGGAGAAGCCAAAATTGACAGCTTCAACAGAAAGGGTCAATGAAAGAATG
OliveBaboon         AGTGTGAGCAGGGAGAAGCCAAAATTGACAGCTTCAACAGAAAGGGTCAACGAAAGAATG
Talapoin            AGTGTGAGCAGGGAGAAGCCAAAATTGACAGCTTCAACAGAAAGGGTCAACAAAAGAATG
WolfsGuenon         AGTGTGAGCAGGGAGAAGCCAAAATTGACAGCTTTAACAGAAAGGGTCAACAAAAGAATG
Colobus             AGTGTGAGCAGGGAGAAGCCAAAATTGACAGCTTCAACAGAAAGGGTCAACAAAAGAATG
Chimpanzee          AGTGTGAGCAGGGAGAAGCCAGAATTGACAGCTTCAACAGAAAGGGTCAACAAAAGAATG
Bonobo              AGTGTGAGCAGGGAGAAGCCAGAATTGACAGCTTCAACAGAAAGGGTCAACAAAAGAATG
Gorilla             AGTGTGAGCAGGGAGAAGCCAGAATTGACAGCTTCAACAGAAAGGGTCAACAAAAGAATG
Orangutan           AGTGTGAGCAGGGAAAAGCCAGAATTGACAGCTTCAACAGAAAGGGTCAACAAAAGAATG
BorneoOrangutan     AGTGTGAGCAGGGAAAAGCCAGAATTGACAGCTTCAACAGAAAGGGTCAACAAAAGAATG
PileatedGibbon      AGTGTGAGCAGGGAGAAGCCAGAATTGACAGCTTCAACAGAAAGGGTCAACAAAAGAATG
AgileGibbon         AGTGTGAGCAGGGAGAAGCCAGAATTGACAGCTTCAACAGAAAGGGTCAACAAAAGAATG
WHGibbon            AGTGTGAGCAGGGAGAAGCCAGAATTGACAGCTTCAACAGAAAGGGTCAACAAAAGAATG
Siamang             AGTGTGAGCAGGGAGAAGCCAGAATTGACAGCTTCAACAGAAAGGGTCAACAAAAGAATG
RCGibbon            AGTGTGAGCAGGGAGAAGCCAGAATTGACAGCTTCAACAGAAAGGGTCAACAAAAGAATG
WCGibbon            AGTGTGAGCAGGGAGAAGCCAGAATTGACAGCTTCAACAGAAAGGGTCAACAAAAGAATG
Marmoset            AATGTGAGCAGGCAGAAGCCAGAACTGGCAGCTTCAACAGAAAGGGTCAATAAAAGAAAG
SqMnky              AGTGTGAGCAGGAAGAAGCCAGAACTGGCAGCTTCAACAGAAAGGGTCAACAAAAGAATG
TitiMonkey          AGTGTGAGCAGGAAGAAGCCAGAATTGGCAGCTTCAACAGAAAGGGTCAACAAAAAAATG
HowlerMk            AGTATGAGCAGGAAGAAGCCAGAATTGGCAGCTTCAACAGAAAGAGTCAACAAAAGAATG
                    * * ******** * ** **  ** ** ****** ********* *****  *** ** *

Human               TCCATGGTGGTGTCTGGCCTGACCCCAGAAGAATTTATGCTCGTGTACAAGTTTGCCAGA
Rhesus              TCCTTGGTGGTGTCTGGCCTGACCCCAGAAGAATTTATGCTCGTGTACAAGTTTGCCAGA
CEMacaque           TCCTTGGTGGTGTCTGGCCTGACCCCAGAAGAATTTATGCTCGTGTACAAGTTTGCCAGA
BMangabey           TCCTTGGTGGTGTCTGGCCTGACCCCAGAAGAATTTATGCTCGTGTACAAGTTTGCCAGA
OliveBaboon         TCCTTGGTGGTGTCTGGCCTGACCCCAGAAGAATTTATGCTCGTGTACAAGTTTGCCAGA
Talapoin            TCCTTGGTGGTGTCTGGCCTGACCCCAGAAGAATTTATGCTCGTGTACAAGTTTGCCAGA
WolfsGuenon         TCCTTGGTGGTGTCTGGCCTGACCCCAGAAGAATTTATGCTCGTGTATAAGTTTGCCAGA
Colobus             TCCTTGGTGGTGTCTGGCCTGACCCCAGAAGAATTTATGCTCGTGTACAAGTTTGCCAGA
Chimpanzee          TCCATGGTGGTGTCTGGCCTGACCCCAGAAGAATTTATGCTCGTGTACAAGTTTGCCAGA
Bonobo              TCCATGGTGGTGTCTGGCCTGACCCCAGAAGAATTTATGCTCGTGTACAAGTTTGCCAGA
Gorilla             TCCATGGTGGTGTCTGGCCTGACCCCAGAAGAATTTATGCTCGTGTACAAGTTTGCCAGA
Orangutan           TCCATGGTGGTGTCTGGCCTGACCCCAGAAGAATTTATGCTCGTGTACAAGTTTGCCAGA
BorneoOrangutan     TCCATGGTGGTGTCTGGCCTGACCCCAGAAGAATTTATGCTCGTGTACAAGTTTGCCAGA
PileatedGibbon      TCCATGGTGGTGTCTGGCCTGACCCCAGAAGAATTTATGCTCGTGTACAACTTTGCCAGA
AgileGibbon         TCCATGGTGGTGTCTGGCCTGACCCCAGAAGAATTTATGCTCGTGTACAACTTTGCCAGA
WHGibbon            TCCATGGTGGTGTCTGGCCTGACCCCAGAAGAATTTATGCTCGTGTACAACTTTGCCAGA
Siamang             TCCATGGTGGTGTCTGGCCTGACCCCAGAAGAATTTATGCTCGTGTACAACTTTGCCAGA
RCGibbon            TCCATGGTGGTGTCTGGCCTGACCCCAGAAGAATTTATGCTCGTGTACAACTTTGCCAGA
WCGibbon            TCCATGGTGGTGTCTGGCCTGACCCCAGAAGAATTTATGCTCGTGTACAACTTTGCCAGA
Marmoset            TCCATGGTGGTGTCAGGCTTGACCCCAGAAGAATTAGTGCTCGTGTACAAGTTTGCCAGA
SqMnky              TCCATGGTAGTGTCAGGCTTGACCCCAGAAGAATTTGTGCTCGTGTACAAGTTTGCCAGA
TitiMonkey          TCCATGGTGGTATCAGGCTTGACTCCAGAAGAATTTGTGATCGTGTACAAGTTTGCCAGA
HowlerMk            TCCATGGTGGTGTCAGGCTTGACCCCAGAAGAATTTGTGCTCGTGTACAAGTTTGCCAGA
                    *** **** ** ** *** **** ***********  ** ******* ** *********

Human               AAACACCACATCACTTTAACTAATCTAATTACTGAAGAGACTACTCATGTTGTTATGAAA
Rhesus              AGATACCACATCGCTTTAACTAATCTAATTAGTGAAGAGACTACTCATGTCGTTATGAAA
CEMacaque           AGATACCACATCGCTTTAACTAATCTAATTAGTGAAGAGACTACTCATGTCGTTATGAAA
BMangabey           AGATACCACATCGCTTTAACTAATCTAATTAGTGAAGAGACTACTCATGTCGTTATGAAA
OliveBaboon         AGATACCACATCGCTTTAACTAATCTAATTAGTGAAGAGACTACTCATGTCGTTATGAAA
Talapoin            AGATACCACATCGCTTTAACTAATCTAATTACTGAAGAGACTACTCATGTCGTTATGAAA
WolfsGuenon         AGATACCACATCGCTTTAACTAATCTAATTACTGAAGAGACTACTCATGTCGTTATGAAA
Colobus             AGATACCACATCGCTTTAACTAATCTAATTACTGAAGAGACTACTCATGTTGTTATGAAA
Chimpanzee          AAACACCACATCACTTTAACTAATCTAATTACTGAAGAGACTACTCATGTTGTTATGAAA
Bonobo              AAACACCACATCACTTTAACTAATCTAATTACTGAAGAGACTACTCATGTTGTTATGAAA
Gorilla             AAACACCACATCACTTTAACTAATCTAATTACTGAAGAGACTACTCATGTTGTTATGAAA
Orangutan           AAACACCACATCACTTTAACTAATCTAATTACTGAAGAGACTACTCATGTTGTTATGAAA
BorneoOrangutan     AAACACCACATCACTTTAACTAATCTAATTACTGAAGAGACTACTCATGTTGTTATGAAA
PileatedGibbon      AAACACCACATCACTTTAACTAATCTAATTACTGAAGAGACTACTCATGTTGTTATGAAA
```

```
AgileGibbon              AAACACCACATCACTTTAACTAATCTAATTACTGAAGAGACTACTCATGTTGTTATGAAA
WHGibbon                 AAACACCACATCACTTTAACTAATCTAATTACTGAAGAGACTACTCATGTTGTTATGAAA
Siamang                  AAACACCACATCACTTTAACTAATCTAATTACTGAAGAGACTACTCATGTTGTTATGAAA
RCGibbon                 AAACACCACATCACTTTAACTAATCTAATTACTGAAGAGACTACTCATGTTGTTATGAAA
WCGibbon                 AAACACCACATCACTTTAACTAATCTAATTACTGAAGAGACTACTCATGTTGTTATGAAA
Marmoset                 CAACACCACATCACTTTGACTAATCAAATTACTGAAGAGACTACTCATGTTGTTATGAAA
SqMnky                   AAACACCACATCACTTTAACTAATCAAATTACTGAAGAGACTACTCATGTTGTTATGAAA
TitiMonkey               AAACACCACATCACTTTAACTAATCAAATTACTGAAGAGACTACTCATGTTGTTATGAAA
HowlerMk                 AAACACCGCATCGCTTTAACTAATCAAATTACTGAAGAGACTACTCATGTTGTTATGAAA
                           * *** **** **** ******* ***** ****************** *********

Human                    ACAGATGCTGAGTTTGTGTGTGAACGGACACTGAAATATTTTCTAGGAATTGCGGGAGGA
Rhesus                   ACAGATGCTGAGTTTGTGTGTGAACGGACGCTGAAATATTTTCTGGGAATTGCGGGAGGA
CEMacaque                ACAGATGCTGAGTTTGTGTGTGAACGGACGCTGAAATATTTTCTGGGAATTGCGGGAGGA
BMangabey                ACAGATGCTGAGTTTGTGTGTGAACGGACGCTGAAATATTTTCTGGGAATTGCGGGAGGA
OliveBaboon              ACAGATGCTGAGTTTGTGTGTGAACGGACGCTGAAATATTTTCTGGGAATTGCGGGAGGA
Talapoin                 ACAGATGCTGAGTTTGTGTGTGAACGGACGCTGAAATATTTTCTGGGAATTGCGGGAGGA
WolfsGuenon              ACAGATGCTGAGTTTGTGTGTGAACGGACGCTGAAATATTTTCTGGGAATTGCGGGAGGA
Colobus                  ACAGATGCTGAGTTTGTGTGTGAACGGACGCTGAAATATTTTCTGGGAATTGCGGGAGGA
Chimpanzee               ACAGATGCTGAGTTTGTGTGTGAACGGACACTGAAATATTTTCTAGGAATTGCGGGAGGA
Bonobo                   ACAGATGCTGAGTTTGTGTGTGAACGGACACTGAAATATTTTCTAGGAATTGCGGGAGGA
Gorilla                  ACAGATGCTGAGTTTGTGTGTGAACGGACACTGAAATATTTTCTAGGAATTGCGGGAGGA
Orangutan                ACAGATGCTGAGTTTGTGTGTGAACGGACACTGAAATATTTTCTAGGAATTGCGGGAGGA
BorneoOrangutan          ACAGATGCTGAGTTTGTGTGTGAACGGACACTGAAATATTTTCTAGGAATTGCGGGAGGA
PileatedGibbon           ACAGATGCTGAGTTTGTGTGTGAACGGACACTGAAATATTTCCTAGGAATTGCGGGAGGA
AgileGibbon              ACAGATGCTGAGTTTGTGTGTGAACGGACACTGAAATATTTCCTAGGAATTGCGGGAGGA
WHGibbon                 ACAGATGCTGAGTTTGTGTGTGAACGGACACTGAAATATTTCCTAGGAATTGCGGGAGGA
Siamang                  ACAGATGCTGAGTTTGTGTGTGAACGGACACTGAAATATTTCCTAGGAATTGCAGGAGGA
RCGibbon                 ACAGATGCTGAGTTTGTGTGTGAACGGACACTGAAATATTTCCTAGGAATTGCGGGAGGA
WCGibbon                 ACAGATGCTGAGTTTGTGTGTGAACGGACACTGAAATATTTCCTAGGAATTGCGGGAGGA
Marmoset                 ACAGATGCTGAGTTTGTGTGTGAACGGACACTGAAATACTTTCTGGGAATTGCAGGAGGA
SqMnky                   ACAGATGCCGAGTTTGTGTGTGAACGGACACTGAAATATTTTCTGGGAATTGCAGGAGGA
TitiMonkey               ACAGATGCCGAGTTTGTGTGTGAACGGACGCTGAAATATTTTCTGGGAATTGCAGGAGGA
HowlerMk                 ACAGATGCCGAGTTTGTGTGTGAACGGACACTGAAATATTTTCTGGGAATTGCAGGAGGA
                         ******** ******************** ******** ** ** ******** ******

Human                    AAATGGGTAGTTAGCTATTTCTGGGTGACCCAGTCTATTAAAGAAAGAAAAATGCTGAAT
Rhesus                   AAATGGGTAGTTAGCTATTTCTGGGTGACCCAGTCTATTAAAGAAAGAAAAATGCTGAAT
CEMacaque                AAATGGGTAGTTAGCTATTTCTGGGTGACCCAGTCTATTAAAGAAAGAAAAATGCTGAAT
BMangabey                AAATGGGTAGTTAGCTATTTCTGGGTGACCCAGTCTATTAAAGAAAGAAAAATGCTGAAT
OliveBaboon              AAATGGGTAGTTAGCTATTTCTGGGTGACCCAGTCTATTAAAGAAAGAAAAATGCTGAAT
Talapoin                 AAATGGGTAGTTAGCTATTTCTGGGTGACCCAGTCTATTAAAGAAAGAAAAATGCTGAAT
WolfsGuenon              AAATGGGTAGTTAGCTATTTCTGGGTGACCCAGTCTATTAAAGAAAGAAAAATGCTGAAT
Colobus                  AAATGGGTAGTTAGCTATTTCTGGGTGACCCAGTCTATTAAAGAAAGAAAAATGCTGAAT
Chimpanzee               AAATGGGTAGTTAGCTATTTCTGGGTGACCCAGTCTATTAAAGAAAGAAAAATGCTGAAT
Bonobo                   AAATGGGTAGTTAGCTATTTCTGGGTGACCCAGTCTATTAAAGAAAGAAAAATGCTGAAT
Gorilla                  AAATGGGTAGTTAGCTATTTCTGGGTGACCCAGTCTATTAAAGAAGGAAAAATGCTGAAT
Orangutan                AAATGGGTAGTTAGCTATTTCTGGGTGACCCAGTCTATTAAAGAAAGAAAAATGCTGAAT
BorneoOrangutan          AAATGGGTAGTTAGCTATTTCTGGGTGACCCAGTCTATTAAAGAAAGAAAAATGCTGAAT
PileatedGibbon           AAATGGGTAGTTAGCTATTTCTGGGTGACCCAGTCTATTAAAGAAAGAAAAATGCTGAAT
AgileGibbon              AAATGGGTAGTTAGCTATTTCTGGGTGACCCAGTCTATTAAAGAAAGAAAAATGCTGAAT
WHGibbon                 AAATGGGTAGTTAGCTATTTCTGGGTGACCCAGTCTATTAAAGAAAGAAAAATGCTGAAT
Siamang                  AAATGGGTAGTTAGCTATTTCTGGGTGACCCAGTCTATTAAAGAAAGAAAAATGCTGAAT
RCGibbon                 AAATGGGTAGTTAGCTATTTCTGGGTGACCCAGTCTATTAAAGAAAGAAAAATGCTGAAT
WCGibbon                 AAATGGGTAGTTAGCTATTTCTGGGTGACCCAGTCTATTAAAGAAAGAAAAATGCTGAAT
Marmoset                 AAATGGGTAGTTAGCTATTTCTGGGTGACCCAGTCTATTAAAGAAAGAAAAATGCTGAAT
SqMnky                   AAATGGGTAGTTAGCTATTTCTGGGTGACCCAGTCTATTAAAGAAAGAAAAATGCTGAAT
TitiMonkey               AAATGGGTAGTTAGCTATTTCTGGGTGACCCAGTCTGTTAAAGAAAGAAAAATGCTGAAT
HowlerMk                 AAATGGGTAGTTAGCTATTTCTGGGTGACCCAGTCTATTAAAGAAAGAAAAATGCTGAAT
                         ************************************ ******** **************

Human                    GAGCATGATTTTGAAGTCAGAGGAGATGTGGTCAATGGAAGAAACCACCAAGGTCCAAAG
Rhesus                   GAGCATGATTTTGAAGTCAGAGGAGATGTGGTCAATGGAAGAAACCACCAAGGTCCAAAG
CEMacaque                GAGCATGATTTTGAAGTCAGAGGAGATGTGGTCAATGGAAGAAACCACCAAGGTCCAAAG
BMangabey                GAGCATGATTTTGAAGTCAGAGGAGATGTGGTCAATGGAAGAAACCACCAAGGTCCAAAG
OliveBaboon              GAGCATGATTTTGAAGTCAGAGGAGATGTGGTCAATGGAAGAAACCACCAAGGTCCAAAG
Talapoin                 GAGCATGATTTTGAAGTCAGAGGAGATGTGGTCAATGGAAGAAACCACCAAGGTCCAAAG
WolfsGuenon              GAGCATGATTTTGAAGTCAGAGGAGATGTGGTCAATGGAAGAAACCACCAAGGTCCAAAG
Colobus                  GAGCATGATTTTGAAGTCAGAGGAGATGTGGTCAATGGAAGAAACCACCAAGGTCCAAAG
Chimpanzee               GAGCATGATTTTGAAGTCAGAGGAGATGTGGTCAATGGAAGAAACCACCAAGGTCCAAAG
Bonobo                   GAGCATGATTTTGAAGTCAGAGGAGATGTGGTCAATGGAAGAAACCACCAAGGTCCAAAG
Gorilla                  GAGCATGATTTTGAAGTCAGAGGAGATGTGGTCAATGGAAGAAACCACCAAGGTCCAAAG
Orangutan                GAGCATGATTTTGAAGTCAGAGGAGATGTGGTCAATGGAAGAAACCACCAAGGTCCAAAG
```

```
BorneoOrangutan       GAGCATGATTTTGAAGTCAGAGGAGATGTGGTCAATGGAAGAAACCACCAAGGTCCAAAG
PileatedGibbon        GAGCATGATTTTGAAGTCAGAGGAGATGTGGTCAATGGAAGAAACCACCAAGGTCCAAAG
AgileGibbon           GAGCATGATTTTGAAGTCAGAGGAGATGTGGTCAATGGAAGAAACCACCAAGGTCCAAAG
WHGibbon              GAGCATGATTTTGAAGTCAGAGGAGATGTGGTCAATGGAAGAAACCACCAAGGTCCAAAG
Siamang               GAGCATGATTTTGAAGTCAGAGGAGATGTGGTCAATGGAAGAAACCACCAAGGTCCAAAG
RCGibbon              GAGCATGATTTTGAAGTCAGAGGAGATGTGGTCAATGGAAGAAACCACCAAGGTCCAAAG
WCGibbon              GAGCATGATTTTGAAGTCAGAGGAGATGTGGTCAATGGAAGAAACCACCAAGGTCCAAAG
Marmoset              GAGCATGATTTTGAAGTCAGAGGAGATGTGGTCAATGGAAGAAACCACCAAGGTCCAAGA
SqMnky                GAGCATGATTTTGAAGTCAGAGGAGATGTGGTCAATGGAAGAAACCACCAAGGTCCAAGG
TitiMonkey            GAGCATGATTTTGAAGTCAGAGGAGATGTGGTCAATGGAAGAAACCACCAAGGTCCAAGG
HowlerMk              GAGCATGATTTTGAAGTCAGAGGAGATGTGGTCAATGGAAGAAACCACCAAGGTCCAAGG
                      **********************************************************

Human                 CGAGCAAGAGAATCCCAGGACAGAAAGATCTTCAGGGGGCTAGAAATCTGTTGCTATGGG
Rhesus                CGAGCAAGAGAATCCCCAGACAGAAAGATCTTCAGGGGGCTAGAAATCTGTTGTTATGGG
CEMacaque             CGAGCAAGAGAATCCCCAGACAGAAAGATCTTCAGGGGGCTAGAAATCTGTTGTTATGGG
BMangabey             CGAGCAAGAGAATCCCCAGACAGAAAGATCTTCAGGGGGCTAGAAATCTGTTGTTATGGG
OliveBaboon           CGAGCAAGAGAATCCCCAGACAGAAAGATCTTCAGGGGGCTAGAAATCTGTTGTTATGGG
Talapoin              CGAGCAAGAGAATCCCCAGACAGAAAGATCTTCAGGGGGCTAGAAATCTGTTGTTATGGG
WolfsGuenon           CGAGCAAGAGAATCCCCAGACAGAAAGATCTTCAGGGGGCTAGAAATCTGTTGTTATGGG
Colobus               CGAGCAAGAGAATCCCCGGACAGAAAGATCTTCAGGGGGCTAGAAATCTGTTGCTATGGG
Chimpanzee            CGAGCAAGAGAATCCCAGGACAGAAAGATCTTCAGGGGGCTAGAAATCTGTTGCTATGGG
Bonobo                CGAGCAAGAGAATCCCAGGACAGAAAGATCTTCAGGGGGCTAGAAATCTGTTGCTATGGG
Gorilla               CGAGCAAGAGAATCCCAGGACAGAAAGATCTTCAGGGGGCTAGACATCTGTTGCTATGGG
Orangutan             CGAGCAAGAGAATCTCAGGACAGAAAGATCTTCAGGGGGCTAGAAATCTGTTGCTATGGG
BorneoOrangutan       CGAGCAAGAGAATCTCAGGACAGAAAGATCTTCAGGGGGCTAGAAATCTGTTGCTATGGG
PileatedGibbon        CGAGCAAGAGAATCCCAGGACAGAAAGATCTTCAGAGGGCTAGAAATCTGTTGCTATGGG
AgileGibbon           CGAGCAAGAGAATCCCAGGACAGAAAGATCTTCAGAGGGCTAGAAATCTGTTGCTATGGG
WHGibbon              CGAGCAAGAGAATCCCAGGACAGAAAGATCTTCAGAGGGCTAGAAATCTGTTGCTATGGG
Siamang               CGAGCAAGAGAATCCCAGGACAGAAAGATCTTCAGAGGGCTAGAAATCTGTTGCTATGGG
RCGibbon              CGAGCAAGAGAATCCCAGGACAGAAAGATCTTCAGAGGGCTAGAAATCTGTTGCTATGGG
WCGibbon              CGAGCAAGAGAATCCCAGGACAGAAAGATCTTCAGAGGGCTAGAAATCTGTTGCTATGGG
Marmoset              CGAGCAAGAGAATCCCAGGGCAGAAAGATCTTCAGGGGCCTGGAAATATGTTGCTATGGG
SqMnky                CGAGCAAGAGAATCCCAGGGCAAAAAGATCTTCAGGGGCCTAGAAATCTGTTGTTATGGG
TitiMonkey            CGAGCAAGAGAATCCCAGGGCAGAAAGATCTTCAGGGGCCTGGAAATCTGTTGCTATGGG
HowlerMk              CGAGCAAGAGAATCCCAGGGCAGAAAGATCTTCAGGGGCCTAGAAATCTGTTGCTATGGG
                      ************** *  * ** ************ ** ** ** ** ***** ******

Human                 CCCTTCACCAACATGCCCACAGATCAACTGGAATGGATGGTACAGCTGTGTGGTGCTTCT
Rhesus                CCCTTCACCAACATGCCCACAGATCAACTGGAGTGGATGGTACAGCTTTGTGGTGCTTCT
CEMacaque             CCCTTCACCAACATGCCCACAGATCAACTGGAGTGGATGGTACAGCTTTGTGGTGCTTCT
BMangabey             CCCTTCACCAACATGCCCACAGATCAACTGGAGTGGATGGTACAGCTTTGTGGTGCTTCT
OliveBaboon           CCCTTCACCAACATGCCCACAGATCAACTGGAGTGGATGGTACAGCTTTGTGGTGCTTCT
Talapoin              CCCTTCACCAACATGCCCACAGATCAACTGGAGCGGATGGTACAGCTGTGTGGTGCTTCT
WolfsGuenon           CCCTTCACCAACATGCCCACAGATCAACTGGAGTGGATGGTACAGCTGTGTGGTGCTTCT
Colobus               CCCTTCACCAACATGCCCACAGATCAACTGGAATGGATGGTAGAGCTGTGTGGTGCTTCT
Chimpanzee            CCCTTCACCAACATGCCCACAGATCAACTGGAATGGATGGTACAGCTGTGTGGTGCTTCT
Bonobo                CCCTTCACCAACATGCCCACAGATCAACTGGAATGGATGGTACAGCTGTGTGGTGCTTCT
Gorilla               CCCTTCACCAACATGCCCACAGATCAACTGGAATGGATGGTACAGCTGTGTGGTGCTTCT
Orangutan             CCCTTCACCAACATGCCCACAGATCAACTGGAATGGATAGTACAGCTGTGTGGTGCTTCT
BorneoOrangutan       CCCTTCACCAACATGCCCACAGATCAACTGGAATGGATAGTACAGCTGTGTGGTGCTTCT
PileatedGibbon        CCCTTCACCAACATGCCCACAGATCAACTGGAGTGGATGGTACAGCTGTGTGGTGCTTCT
AgileGibbon           CCCTTCACCAACATGCCCACAGATCAACTGGAGTGGATGGTACAGCTGTGTGGTGCTTCT
WHGibbon              CCCTTCACCAACATGCCCACAGATCAACTGGAGTGGATGGTACAGCTGTGTGGTGCTTCT
Siamang               CCCTTCACCAACATGCCCACAGATCAACTGGAGTGGATGGTACAGCTGTGTGGTGCTTCT
RCGibbon              CCCTTCACCAACATGCCCACAGATCAACTGGAGTGGATGGTACAGCTGTGTGGTGCTTCT
WCGibbon              CCCTTCACCAACATGCCCACAGATCAACTGGAGTGGATGGTACAGCTGTGTGGTGCTTCT
Marmoset              CCGTTTACCAATATGCCCACAGATCAACTGGAGTGGATGGTGCAGCTGTGTGGGGCTTCT
SqMnky                CCCTTTACCAACATGCCCACAGATCAACTGGAGTGGATGGTGCAGCTGTGTGGGGCTTCT
TitiMonkey            CCCTTTACCAACATGCCCACAGATCAACTGGAGTGGATGGTGCAGCTGTGTGGGGCTTCT
HowlerMk              CCCTTTACCAACATGCCCACAGATCAACTGGAGTGGATGGTGCAGCTGTGTGGGGCTTCT
                      ** ** ***** ********************  **** **  **** ***** ******

Human                 GTGGTGAAGGAGCTTTCATCATTCACCCTTGGCACAGGTGTCCACCCAATTGTGGTTGTG
Rhesus                GTGGTGAAGGAGCTTTCATCATTCACCCTTGGCACAGGTTTCCACCCAATTGTGGTTGTG
CEMacaque             GTGGTGAAGGAGCTTTCATCATTCACCCTTGGCACAGGTTTCCACCCAATTGTGGTTGTG
BMangabey             GTGGTGAAGGAGCTTTCATCATTCACCCTTGGCACAGGTTTCCACCCAATTGTGGTTGTG
OliveBaboon           GTGGTGAAGGAGCTTTCATCATTCACCCTTGGCACAGGTTTCCACCCAATTGTGGTTGTG
Talapoin              GTGGTGAAGGAGCTTTCATCATTCACCCTTGGCACAGGTTTCCACCCAATTGTGGTTGTG
WolfsGuenon           GTGGTGAAGGAGCTTTCATCATTCACCCTTGGCACAGGTTTCCACCCAATTGTGGTTGTA
Colobus               GTGGTGAAGGAGCTTTCATCATTCACCCTTGGCACAGGTTTCCACCCAGTTGTGGTTGTG
Chimpanzee            GTGGTGAAGGAGCTTTCATCATTCACCCTTGGCACAGGTGTCCACCCAATTGTGGTTGTG
Bonobo                GTGGTGAAGGAGCTTTCATCATTC---------ACAGGTGTCCACCCAATTGTGGTTGTG
```

```
Gorilla             GTGGTGAAGGAGCTTTCATCATTCACCCTTGGCACAGGTGTCCACCCAATTGTGGTTGTG
Orangutan           GTGGTGAAGGAGCTTTCATCATTCACCCTTGGCACAGGTGTCCACCCAATTGTGGTTGTG
BorneoOrangutan     GTGGTGAAGGAGCTTTCATCATTCACCCTTGGCACAGGTGTCCACCCAATTGTGGTTGTG
PileatedGibbon      GTGGTGAAGGAGCTTTCATCATTCACCCTTGGCACAGGTGTCCACCCAATTGTGGTTGTG
AgileGibbon         GTGGTGAAGGAGCTTTCATCATTCACCCTTGGCACAGGTGTCCACCCAGTTGTGGTTGTG
WHGibbon            GTGGTGAAGGAGCTTTCATCATTCACCCTTGGCACAGGTGTCCACCCAATTGTGGTTGTG
Siamang             GTGGTGAAGGAGCTTTCATCATTCACCCTTGGCACAGGTGTCCACCCAATTGTGGTTGTG
RCGibbon            GTGGTGAAGGAGCTTTCATCATTCACCCTTGGCACAGGTGTCCACCCAATTGTGGTTGTG
WCGibbon            GTGGTGAAGGAGCTTTCATCATTCACCCTTGGCACAGGTGTCCACCCAATTGTGGTTGTG
Marmoset            GTGGTGAAGGAGCTTTCATCATTCACCCTTGGCACAGGTATCCACGCAGTTGTGGTTGTG
SqMnky              GTGGTGAAGGAGCTTTCGTCATTCACCCTTGGCACAGGTATCCGTGCAGTTGTGGTTGTG
TitiMonkey          GTGGTGAAGGAGTTTTCATCATTCACCCTTGGCACAAGTATCCACGCAATTGTGGTTGTG
HowlerMk            GTGGTGAAGGAGCTTTCATCATTCACCCTTGGCACAGGTATCCACGCAATTGTGGTTGTG
                    ************ **** ******         *** ** ***   ** ********** 

Human               CAGCCAGATGCCTGGACAGAGGACAATGGCTTCCATGCAATTGGGCAGATGTGTGAGGCA
Rhesus              CAGCCAGATGCCTGGACAGAGGACAATGGCTTCCATGCAATTGGGCAGATGTGTGAGGCA
CEMacaque           CAGCCAGATGCCTGGACAGAGGACAATGGCTTCCATGCAATTGGGCAGATGTGTGAGGCA
BMangabey           CAGCCAGATGCCTGGACAGAGGACAATGGCTTCCATGCAATTGGGCAGATGTGTGAGGCA
OliveBaboon         CAGCCAGATGCCTGGACAGAGGACAATGGCTTCCATGCAATTGGGCAGATGTGTGAGGCA
Talapoin            CAGCCAGATGCCTGGACAGAGGACAATGGCTTCCATGCAATTGGGCAGATGTGTGAGGCA
WolfsGuenon         CAGCCAGATGCCTGGACAGAGGACAATGGCTTCCATGCAATTGGGCAGATGTGTGAGGCA
Colobus             CAGCCAGATGCCTGGACAGAGGACAATGGCTTCCATGCAATTGGGCAGATGTGTGAGGCA
Chimpanzee          CAGCCAGATGCCTGGACAGAGGACAATGGCTTCCATGCAATTGGGCAGATGTGTGAGGCA
Bonobo              CAGCCAGATGCCTGGACAGAGGACAATGGCTTCCATGCAATTGGGCAGATGTGTGAGGCA
Gorilla             CAGCCAGATGCCTGGACAGAGGACAATGGCTTCCATGCAATTGGGCAGATGTGTGAGGCA
Orangutan           CAGCCAGATGCCTGGACAGAGGACAATGGCTTCCATGCAATTGGGCAGATGTGTGAGGCA
BorneoOrangutan     CAGCCAGATGCCTGGACAGAGGACAATGGCTTCCATGCAATTGGGCAGATGTGTGAGGCA
PileatedGibbon      CAGCCAGATGCCTGGACAGAGGACAATGGCTTCCATGCAATTGGGCAGATGTGTGAGGCA
AgileGibbon         CAGCCAGATGCCTGGACAGAGGACAATGGCTTCCATGCAATTGGGCAGATGTGTGAGGCA
WHGibbon            CAGCCAGATGCCTGGACAGAGGACAATGGCTTCCATGCAATTGGGCAGATGTGTGAGGCA
Siamang             CAGCCAGATGCCTGGACAGAGGACAATGGCTTCCATGCAATTGGGCAGATGTGTGAGGCA
RCGibbon            CAGCCAGATGCCTGGACAGAGGACAATGGCTTCCATGCAATTGGGCAGATGTGTGAGGCA
WCGibbon            CAGCCAGATGCCTGGACAGAGGACAATGGCTTCCATGCAATTGGGCAGATGTGTGAGGCA
Marmoset            CAGCCAGATGCCTGGACAGAGGACAACGGCTTACATGCAATTGGGCAGATGTGTGAGGCG
SqMnky              CAGCCAGATGCCTGGACAGAGGACAGTGGCTTCCATGCAATTGGGCAGATGTGTGAAGCA
TitiMonkey          CAGCCAGATGCCTGGACAGAGGACAATGGCTTCCATGCAATTGGGCAGATGTGTAAGGCA
HowlerMk            CAGCCAGATGCCTGGACAGAGGACAATGGCTTCCATGCAATTGGGCAGATGTGTGAGGCA
                    *************************  ***** ********************* * ** 

Human               CCTGTGGTGACCCGAGAGTGGGTGTTGGACAGTGTAGCACTCTACCAGTGCCAGGAGCTG
Rhesus              CCTGTGGTGACCCGAGAGTGGGTGTTGGACAGTGTAGCACTCTACCAGTGCCAGGAGCTG
CEMacaque           CCTGTGGTGACCCGAGAGTGGGTGTTGGACAGTGTAGCACTCTACCAGTGCCAGGAGCTG
BMangabey           CCTGTGGTGACCCGAGAGTGGGTGTTGGACAGTGTAGCACTCTACCAGTGCCAGGAGCTG
OliveBaboon         CCTGTGGTGACCCGAGAGTGGGTGTTGGACAGTGTAGCACTCTACCAGTGCCAGGAGCTG
Talapoin            CCTGTGGTGACCCGAGAGTGGGTGTTGGACAGTGTAGCACTCTACCAGTGCCAGGAGCTG
WolfsGuenon         CCTGTGGTGACCCGAGAGTGGGTGTTGGACAGTGTAGCACTCTACCAGTGCCAGGAGCTG
Colobus             CCTGTGGTGACCCGAGAGTGGGTGTTGGACAGTGTAGCACTCTACCAGTGCCAGGAGCTG
Chimpanzee          CCTGTGGTGACCCGAGAGTGGGTGTTGGACAGTGTAGCACTCTACCAGTGCCAGGAGCTG
Bonobo              CCTGTGGTGACCCGAGAGTGGGTGTTGGACAGTGTAGCACTCTACCAGTGCCAGGAGCTG
Gorilla             CCCGTGGTGACCCGAGAGTGGGTGTTGGACAGTGTAGCACTCTACCAGTGCCAGGAGCTG
Orangutan           CCTGTGGTGACCCGAGAGTGGGTGTTGGACAGTGTAGCACTCTACCAGTGCCAGGAGCTG
BorneoOrangutan     CCTGTGGTGACCCGAGAGTGGGTGTTGGACAGTGTAGCACTCTACCAGTGCCAGGAGCTG
PileatedGibbon      CCTGTGGTGACCCGAGAGTGGGTGTTGGACAGTGTAGCACTCTACCAGTGCCAGGAGCTG
AgileGibbon         CCTGTGGTGACCCGAGAGTGGGTGTTGGACAGTGTAGCACTCTACCAGTGCCAGGAGCTG
WHGibbon            CCTGTGGTGACCCGAGAGTGGGTGTTGGACAGTGTAGCACTCTACCAGTGCCAGGAGCTG
Siamang             CCTGTGGTGACCCGAGAGTGGGTGTTGGACAGTGTAGCACTCTACCAGTGCCAGGAGCTG
RCGibbon            CCTGTGGTGACCCGAGAGTGGGTGTTGGACAGTGTAGCACTCTACCAGTGCCAGGAGCTG
WCGibbon            CCTGTGGTGACCCGAGAGTGGGTGTTGGACAGTGTAGCACTCTACCAGTGCCAGGAGCTG
Marmoset            CCTGTGGTGACTCGAGAGTGGGTGTTGGACAGTGTAGCACTCTACCAGTGCCAGGAGCTG
SqMnky              CCTGTGGTAACTCGAGAGTGGGTGTTGGACAGTGTAGCACTCTACCAGTGCCAGGAGCTG
TitiMonkey          CCTGTGGTGACTCGAGAGTGGGTGTTGGACAGTGTAGCACTCTACCAGTGCCAGGAGCTG
HowlerMk            CCTGTGGTGACTCGAGAGTGGGTGTTGGACAGTGTAGCACTCTACCAGTGCCAGGAGCTG
                    ** ***** ** ************************************************

Human               GACACCTACCTGATACCCCAGATCCCCCACAGCCACTAC
Rhesus              GACACCTACCTGATACCCCAGATCCCCCACAGCCACTAC
CEMacaque           GACACCTACCTGATACCCCAGATCCCCCACAGCCACTAC
BMangabey           GACACCTACCTGATACCCCAGATCCCCCACAGCCACTAC
OliveBaboon         GACACCTACCTGATACCCCAGATCCCCCACAGCCACTAC
Talapoin            GATACCTACCTGATACCCCAGATCCCCCACAGCCACTAC
WolfsGuenon         GACACCTACCTGATACCCCAGATCCCCCACAGCCACTAC
Colobus             GATGCCTACCTGATACCCCAGATCCCCCACAGCCACTAC
```

```
Chimpanzee          GACACCTACCTGATACCCCAGATCCCCCACAGCCACTAC
Bonobo              GACACCTACCTGATACCTCAGATCCCCCACAGCCACTAC
Gorilla             GACACCTACCTGATACCCCAGATCCCCCACAGCCACTAC
Orangutan           GACACCTACCTGATACCCCAGATCCCCCACAGCCACTAC
BorneoOrangutan     GACACCTACCTGATACCCCAGATCCCCCACAGCCACTAC
PileatedGibbon      GATACCTACCTGATACCCCAGATCCCCCACAGCCACTAC
AgileGibbon         GATACCTACCTGATACCCCAGATCCCCCACAGCCACTAC
WHGibbon            GATACCTACCTGATACCCCAGATCCCCCACAGCCACTAC
Siamang             GATACCTACCTGATACCCCAGATCCCCCACAGCCACTAC
RCGibbon            GATACCTACCTGATACCCCAGATCCCCCACAGCCACTAC
WCGibbon            GATACCTACCTGATACCCCAGATCCCCCACAGCCACTAC
Marmoset            GACACCTACCTGATACCCCAGATCCCCCACAGCCACTAC
SqMnky              GACACCTACCTGATACCCCAGATCCCTCACAGCCACTAC
TitiMonkey          GACACCTACCTGATACCACAGATCCCCCACAGCCACTAC
HowlerMk            GACACCTACCTGATACCCCAGCTCCCCCACAGCCACTAC
                    **  ************* *** **** ************
```
