# Supplementary material for: Rapid evolution of BRCA1 and BRCA2 in humans and other primates
Source: BMC Evol Biol. 2014 Jul 11;14:155. doi: 10.1186/1471-2148-14-155 (PMC4106182; doi:10.1186/1471-2148-14-155)
Supplement: Additional file 2 — Alignment of BRCA2 sequences. description – alignment of BRCA2 sequences used in the PAML analyses. [file 1471-2148-14-155-S2.pdf]

BRCA2 Exon 11 CLUSTAL 2.1 multiple sequence alignment

```
Bonobo                  TTATTGCATTCTTCTGTCAAAAGAAGCTGTTCACAGAATGATTCTGAAGAACCAACTTTG
BorneoOrangutan         TTATTGCATTCTTCTGTCAAAAGAAGCTGTTCACAGAATGATTCTGAAGAACCAACTTTG
Chimpanzee              TTATTGCATTCTTCTGTCAAAAGAAGCTGTTCACAGAATGATTCTGAAGAACCAACTTTG
RedCheekedGibbon        TTATTGCATTCTTCTGTCAAAAGAAGCTGTTCACAGAATGATTCTGAAGAACCAACTTTG
Human                   TTATTGCATTCTTCTGTGAAAAGAAGCTGTTCACAGAATGATTCTGAAGAACCAACTTTG
AgileGibbon             TTATTGCATTCTTCTGTCAAAAGAAGCTGTTCACAGAATGATTCTGAAGAACCAACTTTT
Gorilla                 TTATTGCATTCTTCTGTCAAAAGAAGCTGTTCACAGAATGATTCTGAAGAACCAACTTTG
Howler                  TTATTGCATCCTTCTGTCCAAAGAAGCTGTTCACAGATTGATTCTGGAGAACCAACTTTG
Rhesus                  TTATTTCATTCTTCTGTCAAAAGAAGCTGTTCACAGAATTATTCTGAAGAACCAACTTTG
WolfsGuenon             TTATTTCATTCTTCTGTCAAAAGAAGCTGTTCACAGAATTATTCTGAAGAACCAACTTTG
LeafMonkey              TTATTTCATTCTTCTGTCAAAAGAAGCTGTTCACAGAATTATTTTGAAGAACCAACTTTG
BlackMangabey           TTATTTCATTCTTCTGTCGAAAGAAGCTGTTCACAGAATTATTCTGAAGAACCAACTTTG
PileatedGibbon          TTATTGCATTCTTCTGTCAAAAGAAGCTGTTCACAGAATGATTCTGAAGAACCAACTTTG
SquirrelMonkey          TTATTGCATCCTTCTGTCCAAAGAAATTGTTCACAGATTGATTCTGGAGAACCAACTTTG
Titi                    TTATTGCATCCTTCTGTCCAAAGAAACTATTCACAGATTGATTCTGGAGAACCAACTTTG
Orangutan               TTATTGCATTCTTCTGTCAAAAGAAGCTGTTCACAGAATGATTCTGAAGAACCAACTTTG
CEMacaque               TTATTTCATTCTTCTGTCAAAAGAAGCTGTTCACAGAATTATTCTGAAGAACCAACTTTG
Talapoin                TTATTTCATTCTTCTGTCAAAAGAAGCTGTTCACAGAATTATTCTGAAGAACCAACTTTG
WhiteHandedGibbon       TTATTGCATTCTTCTGTCAAAAGAAGCTGTTCACAGAATGATTCTGAAGAACCAACTTTG
Siamang                 TTATTGCATTCTTCTGTCAAAAGAAGCTGTTCACAGAATGATTCTGAAGAACCAACTTTG
WhiteCheekedGibbon      TTATTGCATTCTTCTGTCAAAAGAAGCTGTTCACAGAATGATTCTGAAGAACCAACTTTG
Colobus                 TTATTTCATTCTTCTGTCGAAAGAAGCTGTTCACAGAATTATTTTGAAGAACCAACTTTG
OliveBaboon             TTATTTCATTCTTCTGTCGAAAGAAGGTGTTCACAGAATTATTCTGAAGAACCAACTTTG
Marmoset                TTATTGCATCCTTCTGTCCAAAGAAACTGTTCACAGATTGATTCTGGAGAACCAACTTTG
                        ***** *** *******  ******  * ******** * *** ** ************

Bonobo                  TCCTTAACTAGCTCTTTTGGGACAATTCTGAGGAAATGTTCTAGAAATGAAACATGT---
BorneoOrangutan         TCCTTAACTAGCTCTTTTGGGACAATTCTGAGGAAATGTTCTAGAAATGAAACATGT---
Chimpanzee              TCCTTAACTAGCTCTTTTGGGACAATTCTGAGGAAATGTTCTAGAAATGAAACATGT---
RedCheekedGibbon        TCCTTAACCAGCTCTTTTGGGACAGTTCTGAGGAAATGTTCTAGAAATGAAACATGTTGT
Human                   TCCTTAACTAGCTCTTTTGGGACAATTCTGAGGAAATGTTCTAGAAATGAAACATGT---
AgileGibbon             TCCTTAACCAGCTCTTTTGGGACAATTCTGAGGAAATGTTCTAGAAATGAAACATGTTGT
Gorilla                 TCCTTAACTAGCTCTTTTGGGACAATTCTGAGGAAATGTTCTAGAAATGAAACATGT---
Howler                  TCCTTAACCAGCTCTTCTGGGACAATTCTGAGGAAATGTTCTAGAAATGAAACATGT---
Rhesus                  TCCTTAACCAGCTCTTTTGGGACAATTCTGAGGAAATGTTCTAGAAATGAAACATGTTCT
WolfsGuenon             TCCTTAACCAGCTCTTTTGGGACAATTCTGAGGAAATGTTCTAGAAATGAAACATGTTCT
LeafMonkey              TCCTTAACCAGCTCTTTTGGGACAATTCTGAGAAAATGTTCTAGAAATGCAACATGTTCT
BlackMangabey           TCCTTAACCAGCTCTTTTGGGACAATTCTGAGGAAATGTTCTAGAAATGAAACATGTTCT
PileatedGibbon          TCCTTAACCAGCTCTTTTGGGACAATTCTGAGGAAATGTTCTAGAAATGAAACATGTTGT
SquirrelMonkey          TCCTTAACCAGCTCTTCTGGGACAATTCTGAGGAAATGTTCTAGAAATGAAACATGT---
Titi                    TCCTTAACCAGCTCTTCTGGGACAATTCTGATGAAATGTTCTAGAAATGAAACATGT---
Orangutan               TCCTTAACTAGCTCTTTTGGGACAATTCTGAGGAAATGTTCTAGAAATGAAACATGT---
CEMacaque               TCCTTAACCAGCTCTTTTGGGACAATTCTGAGGAAATGTTCTAGAAATGAAACATGTTCT
Talapoin                TCCTTAACCAGCTCTTTTGGGACAATTCTGAGGAAATGTTCTAGAAATGAAACATGTTCT
WhiteHandedGibbon       TCCTTAACCAGCTCTTTTGGGACAATTCTGAGGAAATGTTCTAGAAATGAAACATGTTGT
Siamang                 TCCTTAACCAGCTCTTTTGGGACAATTCTGAGGAAATGTTCTAGAAATGAAACATGTTGT
WhiteCheekedGibbon      TCCTTAACCAGCTCTTTTGGGACAGTTCTGAGGAAATGTTCTAGAAATGAAACATGTTGT
Colobus                 TCCTTAACCAGCTCTTTTGGGACAATTCTGAGAAAATGTTGTAGAAATGAAACATGTTCT
OliveBaboon             TCCTTAACCAGCTCTTTTGGGACAATTCTGAGGAAATGTTCTAGAAATGAAACATGTTCT
Marmoset                TCCTTAACCAGCTCTTCTGGGACAATTCTGAGGAAATGTACTAGAAATGAAACATGTTGT
                        ******** ******* ******* ******  ******  ******** *******

Bonobo                  TCTAATAATACAGTAATCTCTCAGGATCTTGATTATAAAGAAGCAAAATGTAATAAGGAA
BorneoOrangutan         TCTAATAATACAGTAATCTCTCAGGATCTTGATTATAAAGAAGCAAAATGTAATGAGGAA
Chimpanzee              TCTAATAATACAGTAATCTCTCAGGATCTTGATTATAAAGAAGCAAAATGTAATAAGGAA
RedCheekedGibbon        TCTAATAATACAATAATCTCTCAGGATCTTGATTATAAAGAAGCAAAATGTAATAAGGAA
Human                   TCTAATAATACAGTAATCTCTCAGGATCTTGATTATAAAGAAGCAAAATGTAATAAGGAA
AgileGibbon             TCTAATAATACAATAATCTCTCAGGATCTTGATTATAAAGAAGCAAAATGTAATAAGGAA
Gorilla                 TCTAATGATACAGTAATCTCTCAGGATCTTGATTATAAAGAAGCAAAATGTAATAAGGAA
Howler                  TCTAATAATATGATAATTTCTCAGGCTCTTGATTATAAAGAAGCAAAATGTAATAAGGAA
Rhesus                  TCTAATAATACAATAATCTCTCAGGATCTTGATTATAAAGAAGCAAAATGTAATAAGGAA
WolfsGuenon             TCTAATAATACAATAATCTCTCAGGATCTTGATTATAAAGAAGCAAAATGTAATAAGGAA
LeafMonkey              TCTAATAATACAGTAATCTCTCAGGATCTTGATTATAAAGAAGCAAAATGTAACAAGGAA
BlackMangabey           TCTAATAATACAATAATCTCTCAGGATCTTGATTATAAAGAAGCAAAATGTAATAAGGAA
PileatedGibbon          TCTAATAATACAATAATCTCTCAGGATCTTGATTATAAAGAAGCAAAATGTAATAAGGAA
SquirrelMonkey          TCTAATAATATAATAATTTCTCAGGATCTTGGTTATAAAAAAGCAAAATGTAATAAGGAA
Titi                    TCTAATAATATAATAATTTCTCAGGATCTTGATTATGAAGAAGCAAAATGTAATAAGGAA
Orangutan               TCTAATAATACAGTAATCTCTCAGGATCTTGATTATAAAGAAGCAAAATGTAATGAGGAA
CEMacaque               TCTAATAATACAATAATCTCTCAGGATCTTGATTATAAAGAAGCAAAATGTAATAAGGAA
Talapoin                TCTAATAATACAATAATCTCTCAGGATCTTGATTATAAAGAAGCAAAACATAATAAGGAA
```

```
WhiteHandedGibbon       TCTAATAATACAATAATCTCTCAGGATCTTGATTATAAAGAAGCAAAATGTAATAAGGAA
Siamang                 TCTAATAATACAATAATCTCTCAGGATCTTGATTATAAAGAAGCAAAATGTAATAAGGAA
WhiteCheekedGibbon      TCTAATAATACAATAATCTCTCAGGATCTTGATTATAAAGAAGCAAAATGTAATAAGGAA
Colobus                 TCTAATAATACAATAATCTCTCAGGATCTTGATTATAAAGAAGCAAAATGTAATAAGGAA
OliveBaboon             TCTAATAATACAATAATCTCTCAGGATCTTGATTATAAAGAAGCAAAATGTAATAAGGAA
Marmoset                ---AATAATATAATAATCTCTCAGGATCTTGATTATAAAGAAGCAAAATGTAATAAGGAA
                           *** ***   **** ******* ***** **** ** ********  ***  *****

Bonobo                  AAACTGCAGTTATTTATTACCCCAGAAGCTGATTCTCTGTCATGCCTGCAGGAAGGACAG
BorneoOrangutan         AAACTACAGTTATTTATTACCCCAGAAGCTGATTCTCTGTCATGCCTGCAGGAAGGACAG
Chimpanzee              AAACTGCAGTTATTTATTACCCCAGAAGCTGATTCTCTGTCATGCCTGCAGGAAGGACAG
RedCheekedGibbon        AAACTACAGTTATTTATTACTCCAGAAGCTGATTCTCTGTCATGCCTGCAGGAAGGACAG
Human                   AAACTACAGTTATTTATTACCCCAGAAGCTGATTCTCTGTCATGCCTGCAGGAAGGACAG
AgileGibbon             AAACTACAGTTATTTATTACTCCAGAAGCTGATTCTCTGTCATGCCTGCAGGAAGGACAG
Gorilla                 AAACTACAGTTATTTATTACCCCAGAAGCTGATTCTCTGTCATGCCTGCAGGAAGGACAG
Howler                  AAACTACAGTTATTTATTACCCCAGAAGCTGATTCTCTGTCAAGCCTGCAGGAAGGACAG
Rhesus                  AAACTACAGTTATTTATTACCCCAGAGGCTCATTCTCTGTCGTTTCCGCAGGAAGGGCAG
WolfsGuenon             AAACTACAGTTATTTATTACCCCAGAGGCTGATTCTCTGTCGTTTCTGCAGGAAGGACAG
LeafMonkey              AAACTACAGTTATTTAGTATCCCAGAGGCTGATTCTCTGTCGTGTCTGCAGGAAGGACAG
BlackMangabey           GAACTACAGTTATTTATTACCCCAGAGGCTGATTCTCTGTCGTTTCTACAGGAAGGACAG
PileatedGibbon          AAACTACAGTTATTTATTACTCCAGAAGCTGATTCTCTGTCATGCCTGCAGGAAGGACAG
SquirrelMonkey          AAACCACAGTTATTTATTAACCCAGAAGCTGATTCTCTGTCATGCCTGCAGGAAGGAGAG
Titi                    AAACTACAGTTATTTATTACCCCAGAAGCTGATTCTCTGTCATGCTTGCAGGAAGGACCG
Orangutan               AAACTACAGTTATTTATTACCCCAGAAGCTGATTCTCTGTCATGCCTGCAGGAAGGACAG
CEMacaque               AAACTACAGTTATTTATTACCCCAGAGGCTGATTCTCTGTCGTTTCTGCAGGAAGGGCAG
Talapoin                AAACTACAGTTATTTATTACCCCAGAGGCTGATTCTCTGTCGTTTCTCCAGGAAGGACAG
WhiteHandedGibbon       AAACTACAGTTATTTATTACTCCAGAAGCTGATTCTCTGTCATGCCTGCAGGAAGGACAG
Siamang                 AAACTACAGTTATTTATTACTCCAGAAGCTGATTCTCTGTCATGCCTGCAGGAAGGACAG
WhiteCheekedGibbon      AAACTACAGTTATTTATTACTCCAGAAGCTGATTCTCTGTCATGCCTGCAGGAAGGACAG
Colobus                 AAACTACAGTTATTTAGTATCCCAGAGGCTGATTCTCTGTCGTGTCTGCAGGAGGGACAA
OliveBaboon             GAACTACAGTTATTTATTACCCCAGAGGCTGATTCTCTGTTGTTTCTACAGGAAGGACAG
Marmoset                AAAACGCAGTTATTTATTACCCCAGAAGCTGATTCTCTGTCATGCCTGCAGGAAGGACAG
                         **   ********** **   ***** *** *********        ***** **

Bonobo                  TGTGAAAATGATCCAAAAAGCAAAAAAGTTTCAGATATAAAAGAAGAGGTCTTGGCTGCA
BorneoOrangutan         TGTGAAAATGATCCAAAAAGCAAAAAAGTTTCGGATATAAAAGAAGAGGTCTTGGCTGCA
Chimpanzee              TGTGAAAATGATCCAAAAAGCAAAAAAGTTTCAGATATAAAAGAAGAGGTCTTGGCTGCA
RedCheekedGibbon        TGTGAAAATGATCCAAAAAGCAAAAAAGTTTCAGATATAAAAGAAGAGATCTTGGCTACA
Human                   TGTGAAAATGATCCAAAAAGCAAAAAAGTTTCAGATATAAAAGAAGAGGTCTTGGCTGCA
AgileGibbon             TGTGAAAATGATCCAAAAGGCAAAAAAGTGTCAGATATAAAAGAAGAGATCTTGGCTACA
Gorilla                 TGTGAAAATGATCCAAAAAGCAAAAAAGTTTCAGATATAAAAGAAGAGGTCTTGGCTGCA
Howler                  TGTGAAAATTATCCGAAAAGCAAAAAAGTTTCCGATATAAAAGAAGAGGTCTTGGCTGCA
Rhesus                  TATGAAAATGATCCAAAAAGCAGAAAAGTTTCAGATATAAAAGAAGAGGTCTTGGCTGCA
WolfsGuenon             TATGAAAATGATCCAAAAAGCAGAAAAGTTTCAGATATAAAAGAAGAGGTCTTGGCTGCA
LeafMonkey              TATGAAAATGATCCAAAAAGCAGAAAAGTTTCAGATATAAAAGAAGAGGTCTTGGCTGCA
BlackMangabey           TATGAAAATGATCCAAAAAGCAGAAAAGTTTCAGATATAAAAGAAGAGGTCTTGGCTGCA
PileatedGibbon          TGTGAAAATGATCCAAAAAGCAAAAAAGTTTCAGATATAAAAGAAGAGATCTTGGCTACA
SquirrelMonkey          TGTGAAAATTATCCAAAAAGCAAAAAGGTTTCTGATATAAAAGAAGAGGTCTTGGCCACA
Titi                    TGTGAAAATTATCCAAAAAGCAAAAAAGTTTCCAATATAAAAGAAGAAGTCTTGGCTGCA
Orangutan               TGTGAAAATGATCCAAAAAGCAAAAAAGTTTCGGATATAAAAGAAGAGGTCTTGGCTGCA
CEMacaque               TATGAAAATGATCCAAAAAGCAGAAAAGTTTCAGATATAAAAGAAGAGGTCTTGGCTGCA
Talapoin                TATGAAAATGATCCAAAAAGCAGAAAAGTTTCAGATATAAAAGAAGAGGTCTTGGCTGCA
WhiteHandedGibbon       TGTGAAAATGATCCAAAAAGCAAAAAAGTTTCAGATATAAAAGAAGAGATCTTGGCTACA
Siamang                 TGTGAAAATGATCCAAAAAGCAAAGAAGTTTCAGATATAAAAGAAGAGGTCTTGGCTGCA
WhiteCheekedGibbon      TGTGAAAATGATCCAAAAAGCAAAAAAGTTTCAGATATAAAAGAAGAGATCTTGGCTACA
Colobus                 TATGAAAATGATCCAAAAAGCAGAAAAGTTTCAGATATAAAAGAAGAGGTCTTGGCTGCA
OliveBaboon             TATGAAAATGATCCAAAAAGCAGAAAAGTTTCAGATATAAAAGAAGAGGTCTTGGCTGCA
Marmoset                TGTAAAAATTATCCAAAAAGCAAAGAAGTTTCTGATATAAAAGAAGAGGTCTTGGCCACA
                        * * ***** **** *** *** * * ** **  *************  *******  **

Bonobo                  GCATGTCACCCAGTACAACATTCAAAAGTGGAATACAGTGATACTGACTTTCAATCCCAG
BorneoOrangutan         GCATGTCACCCAGTACAAGATTCAAAAGTGGAATACAGTGATACTGACTTTCAATCCCAG
Chimpanzee              GCATGTCACCCAGTACAACATTCAAAAGTGGAATACAGTGATACTGACTTTCAATCCCAG
RedCheekedGibbon        GCATGTCACCCAGTACAACATTCAAAAGTGGAATATAGTGATACTGACTTTCAATCCCGG
Human                   GCATGTCACCCAGTACAACATTCAAAAGTGGAATACAGTGATACTGACTTTCAATCCCAG
AgileGibbon             GCATGTCACCCAGTACAACATTCAAAAATGGAATATAGTGATACTGACTTTCAATCCCAG
Gorilla                 GCATGTCACCCAGTACAACATTCAAAAGTGGAATACAGTGATACTGACTTTCAATCCCAG
Howler                  GCATGTCACCTGGTACAACATTCAGAAGTGGAATATTGTGATACTGACTTTCAGTCCCAG
Rhesus                  GCACATCACCCAGTACAACATTCAAAAGCAGAATATAGTGATACTGACTTTCAATCCCAG
WolfsGuenon             GCACATCACCCAGTACAACATTCAAAAGCAGAATATAGTGATACTGACTTTCAATCCCAG
LeafMonkey              GCACATCACCCAGTACAACATTCAAAAGCAGAATATAGTGATACTGACTTTCAATCCCAG
BlackMangabey           GCACATCACCCAGTACAACATTCAAAAGCAGAATATAGTGATACTGACTTTCAATCCCAG
PileatedGibbon          GCATGTCACCCAGTACAACATTCAAAAATGGAATATAGTGATACTGACTTTCAATCCCAG
```

```
SquirrelMonkey         GTGTGTCACCTGGTACAACATTCAGAAGTGGAATATTGTGATACTGACTTTCAGTCCCAG
Titi                   GCATGTCACCCGGTACAACATTCAGAAGTGGAATATTGTGATACTGACTTTCAGTCCCAG
Orangutan              GCATGTCACCCAGTACAAGATTCAAAAGTGGAATACAGTGATACTGACTTTCAATCCCAG
CEMacaque              GCACATCACCCAGTACAACATTCAAAAGCAGAATATAGTGATACTGACTTTCAATCCCAG
Talapoin               GCACATCACCCAGTACAACACTCAAAAGCAGAATATAGTGATACTGACTTTCAATCCCAG
WhiteHandedGibbon      GCATGTCACCCAGTACAACATTCAAAAATGGAATATAGTGATACTGACTTTCAATCCCAG
Siamang                GCATGTCACCCAGTACAACATTCAAAAGTGGAATATAGTGATACTGACTTTCAATCCCAG
WhiteCheekedGibbon     GCATGTCACCCAGTACAACATTCAAAAGTGGAATATAGTGATACTGACTTTCAATCCCGG
Colobus                GCACATCACCTAGTACAACATTCAAAAGCAGAATATAGTGATACTGACTTTCAATTCCAG
OliveBaboon            GCACATCACCCAGTACAACATTCAAAAGCAGAATATAGTGATACTGACTTTCAATCCCAG
Marmoset               GCATGTCACCTGGCACAACATTCAGAAGTGGAATATTGTGATACTGACTTTCAGTCCCAG
                       *    *****  * **** * *** **   *****  *************** * ** *

Bonobo                 AAAAGTCTTTTATATGACCATGAAAATGCCAGCACTCTTATTTTAACTCCTACTTCCAAG
BorneoOrangutan        AAAAGTCTTTTATATGACCATGAAAATGCCAGCACTCTTATTTTAACTCCTACTTCCAAG
Chimpanzee             AAAAGTCTTTTATATGACCATGAAAATGCCAGCACTCTTATTTTAACTCCTACTTCCAAG
RedCheekedGibbon       AAAAGTCTTTTATATGACCATGAAAATGCCAGCATTCTTATTTTAACTCCTACTTCCAAG
Human                  AAAAGTCTTTTATATGATCATGAAAATGCCAGCACTCTTATTTTAACTCCTACTTCCAAG
AgileGibbon            AGAAGTCTTTTATATGACCATGAAAATGCCAGCATTCTTATTTTAACTCCTACTTCCAAG
Gorilla                AAAAGTCTTTTATATGACCATGAAAATGCCAGCACTCTTATTTTAACTCCTACTTCCAAG
Howler                 AAAAGTCTTTTACATGACAATCAAAATGCCAGCACTCTTATTTTAACTCCTACTTCCAAG
Rhesus                 AAAAGTCCTTTATATGACCATGAAAATGCCAGCACTCTTATTTTAACTCCTACTTCCAAG
WolfsGuenon            AAAAGTCCTTTATATGACCATGAAAATGCCAGCACTCTTATTTTAACTCCTACTTCCAAG
LeafMonkey             AAAAGTCCTTTATATGACCATGAAAATGCCAGCACTCTTATTTTAACTCCTACTTCCAAG
BlackMangabey          AAAAGTCCTTTATATGACCATGAAAATGCCAGCACTCTTATTTTAACTCCTACTTCCAAG
PileatedGibbon         AAAAGTCTTTTATATGACCATGAAAATGCCAGCATTCTTATTTTAACTCCTACTTCCAAG
SquirrelMonkey         AAAAGTCTTTTTGTGACCATGAAAATGCCAGCACTCGTATTTTAACTCCTGCTTCCAAG
Titi                   AAAAGTCTTTTACATGACCATGAAAATGCCAGCACTTTTATTTTAACTCCTACTTCCAAG
Orangutan              AAAAGTCTTTTATATGACCATGAAAATGCCAGCACTCTTATTTTAACTCCTACTTCCAAG
CEMacaque              AAAAGTCCTTTATATGACCATGAAAATGCCAGCACTCTTATTTTAACTCCTACTTCCAAG
Talapoin               AAAAGTCTTTTATATGACCATGAAAATGCCAGCACTCTTATTTTAACTCCTACTTCCAAG
WhiteHandedGibbon      AAAAGTCTTTTATATGACCATGAAAATGCCAGCATTCTTATTTTAACTCCTACTTCCAAG
Siamang                AAAAGTCTTTTATATGACCATGAAAATGCCAGCATTCTTATTTTAACTCCTACTTCCAAG
WhiteCheekedGibbon     AAAAGTCTTTTATATGACCATGAAAATGCCAGCATTCTTATTTTAACTCCTACTTCCAAG
Colobus                AAAAGTCCTTTATATGACCATGAAAATGCCAGCACTCTTATTTTAACTCCTACTTCCAAG
OliveBaboon            AAAAGTTCTTTATATGACCATGAAAATGCCAGCACTCTTATTTTAACTCCTACTTCCAAG
Marmoset               AAAACTATTTTACATGACCATGAAAATGCCAGCTCTCTTATTTTAACTCCTGCTTTCAAG
                       * ** *  ***   ***  ** ***********  *  ************* *** ****

Bonobo                 GATGTTCTGTCAAACCTAGTCATGATTTCTAGAGGCAAAGAATCATACAAAATGTCAGAC
BorneoOrangutan        GATGTTCTGTCAAACCTAGTCATGATTTCTAGAGGCAAAGAATCATACAAAATGTCAGAC
Chimpanzee             GATGTTCTGTCAAACCTAGTCATGATTTCTAGAGGCAAAGAATCATACAAAATGTCAGAC
RedCheekedGibbon       GATGTTCTGTCAAACCTAGTCATGATTTCTAGAGGCAAAGAATCATACAAAATGTCAGAC
Human                  GATGTTCTGTCAAACCTAGTCATGATTTCTAGAGGCAAAGAATCATACAAAATGTCAGAC
AgileGibbon            GATGTTCTGTCAAACCTAGTCATGATTTCTAGAGGCAAAGAATCATACAAAATGTCAGAC
Gorilla                GATGTTCTGTCAAACCTAGTCATGATTTCTAGAGGCAAAGAATCATACAAAATGTCAGAC
Howler                 GATGTACTGTCAAACCTAGTCATGACTTCTAGGGGCAAAGAATCGTACAGAATTTCAGAC
Rhesus                 GATGTTCTGTCAAACCTAGTCAAGATTTCTAGAGGCAAAGAGTCATACAAAATGTCAGAC
WolfsGuenon            GATGTTCTGTCAAACCTAGTCATGATTTCTAGAGGCAAAGAGTCATACAAAATGTCAGAC
LeafMonkey             GATGTTCTGTCAAACCTAGTCATGATTTCTAGAGGCAAAGAATCATACAAAATGTCAGAC
BlackMangabey          GATGTTCTGTCAAACCTAGTCATGATTTCTAGAGGCAAAGAGTCATACAAAATGTCAGAC
PileatedGibbon         GATGTTCTGTCAAACCTAGTCATGATTTCTAGAGGCAAAGAATCATACAAAATGTCAGAC
SquirrelMonkey         GATGTACTGTCAAACCTAGTCAGGACTTCTAGAGGCAAAGAATCATACAGAATTTCAGAC
Titi                   GATGTACTGTCAAACCTAGTCATGACTTCTAGAGGCAAAGAATCATACAGAATTTCAGAC
Orangutan              GATGTTCTGTCAAACCTAGTCATGATTTCTAGAGGCAAAGAATCATACAAAATGTCAGAC
CEMacaque              GATGTTCTGTCAAACCTAGTCATGATTTCTAGAGGCAAAGAGTCATACAAAATGTCAGAC
Talapoin               GATGTTCTGTCAAACCTAGTCATGATTTCTAGAGGCAAAGAATCATATAAAATGTCAGAC
WhiteHandedGibbon      GATGTTCTGTCAAACCTAGTCATGATTTCTAGAGGCAAAGAATCATACAAAATGTCAGAC
Siamang                GATGTTCTGTCAAACCTAGTCATGATTTCTAGAGGCAAAGAATCATACAAAATGTCAGAC
WhiteCheekedGibbon     GATGTTCTGTCAAACCTAGTCATGATTTCTAGAGGCAAAGAATCATACAAAATGTCAGAC
Colobus                GATGTTCTGTCAAACCTAGTCATGATTTCTAGAGGCAAAGAATCATACAAAATGTCAGAT
OliveBaboon            GATGTTCTGTCAAACCTAGTCATGATTTCTAGAGGCAAAGAGTCATACAAAATGTCAGAC
Marmoset               GATGTACTGTCA---------------------GGCAAAGAATCATACAGAATTTCAGAC
                       ***** ******                     ******** ** ** * *** *****

Bonobo                 AAGCTCAAAGGTAACAATTATGAATCTGATGTTGAATTAACCAAAAATATTCCCATGGAA
BorneoOrangutan        AAGCTCAAAGGTAACAATTATGAATCTGATGTTGAATTAACCAAAAATATTCCCATGGAA
Chimpanzee             AAGCTCAAAGGTAACAATTATGAATCTGATGTTGAATTAACCAAAAATATTCCCATGGAA
RedCheekedGibbon       AAGGTCAAAGGTAACAATTATGAAACTGAAGTTGAATTAACCAAAAATATTCCCATGGAA
Human                  AAGCTCAAAGGTAACAATTATGAATCTGATGTTGAATTAACCAAAAATATTCCCATGGAA
AgileGibbon            AAGCTCAAAGGTAACAATTATGAAACTGAAGTTGAATTAACCAAAAATATTCCCATGGAA
Gorilla                AAGCTCAAAGGTAACAATTATGAATCTGATGTTGAATTAACCAAAAATATTCCCATGGAA
Howler                 GAGCTCAAATGTAACAATTATGAAGCTGATTTTGAATTAACCAAAAATATTCCCATGGAA
```

```
Rhesus                  GAGCTCAAAGGTAACAATTATGAGTCTGATTTTGAACTAACCAAAAATATTCCCATGGAA
WolfsGuenon             GAGCTCAAAGGTAACAATTATGAGTCTGATTTTGAACTAACCAAAAATATTCCCATGGAA
LeafMonkey              GAGCTCAAAGGTAACAATTATGAATCTGATTTTGAACTAACCAAAAATATTCCCATGGAA
BlackMangabey           GAGCTCAAAGGTAACAATTATGAGTCTGATTTTGAACTAACCAAAAATATTCCCATGGAA
PileatedGibbon          AAGCTCAAAGGTAACAATTATGAAACTGAAGTTGAATTAACCAAAAATATTCCCATGGAA
SquirrelMonkey          GAGCTCAAATGTAACAATTGTGAAGCTGATTTTGAATTAACCAAAAATATTCCCATGGAA
Titi                    GAGCTCAAATGTAACAATTATGAAGCTGATTTTGAATTAACCAAAAATATTCCCATGGAA
Orangutan               AAGCTCAAAGGTAACAATTATGAATCTGATGTTGAATTAACCAAAAATATTCCCATGGAA
CEMacaque               GAGCTCAAAGGTAACAATTATGAGTCTGATTTTGAACTAACCAAAAATATTCCCATGGAA
Talapoin                GAGCTCAAAGGTAACAATTATGAATCTGATTTTGAACTAACCAAAAATATTCCCATGGAA
WhiteHandedGibbon       AAGCTCAAAGGTAACAATTATGAAACTGAAGTTGAATTAACCAAAAATATTCCCATGGAA
Siamang                 AAGCTCAAAGGTAACAATTATGAAACTGATGTTGAATTAACCAAAAATATTCCCATGGAA
WhiteCheekedGibbon      AAGGTCAAAGGTAACAATTATGAAACTGAAGTTGAATTAACCAAAAATATTCCCATGGAA
Colobus                 GAGCTCAAAGGTAACAATTATGAATCTGATTTTGAACTAACCAAAAATATTCCCATGGAA
OliveBaboon             GAGCTCAAAGGTAACAATTATGAGTCTGATTTTGAACTAACCAAAAATATTCCCATGGAA
Marmoset                GAGCTCAAATGTAACAATTGTGAAGCCGATTTTGAGTTAATCAAAAATATTTCCATGGAA
                         ** ***** ********* ***  * **  ****  *** ********** ********

Bonobo                  AAGAATCAAGATGTATGTGCTTTAAATGAAAATTATAAAAACGTTGAGCTGTTGCCACCT
BorneoOrangutan         AAGAATCAAGATATATGTGCTTCAAATGAAAATGATAAAAACGTTGAGCTGTTGCCACCT
Chimpanzee              AAGAATCAAGATGTATGTGCTTTAAATGAAAATTATAAAAACGTTGAGCTGTTGCCACCT
RedCheekedGibbon        AAGAATCAAGATGTATGTGCTTTAAATGAAAATTCTAAAAATGTTGAGCTGTTGCCACCT
Human                   AAGAATCAAGATGTATGTGCTTTAAATGAAAATTATAAAAACGTTGAGCTGTTGCCACCT
AgileGibbon             AAGAATCAAGATGTATGTGCTTTAAATGAAAATTCTAAAAATGTTGAGCTGTTGCCACCT
Gorilla                 AAGAATCAAGATGTATGTGCTTTAAATGAAAATTATAAAAACGTTGAGCTGTTGCCACCT
Howler                  AATAATCAAGATGTACATGCTTTAAATGAAAATTCTAAAAACATTGAAGTGTTGCCACCT
Rhesus                  AAGAATCAAGATGTATGTGCTTTAAGTGAAAATTCTAAACATGTTGAGCTGTTGCCACCT
WolfsGuenon             AAGAATCAAGATGTATGTGCTTTAAGTGAAAATTCTAAACATGTTGAGCTGTTGCCACCT
LeafMonkey              AAGAATCAAGATGTATGTGCTTTAAGTGAAAATTCTAAAAATGTTGAGCTATTGCCACCT
BlackMangabey           AAGAATCAAGATGTATGTGCTTTAAGTGAAAATTCTAAACATGTTGAGCTGTTGCCACCT
PileatedGibbon          AAGAATCAAGATGTATGTGCTTTAAATGAAAATTCTAAAAATGTTGAGCTGTTGCCACCT
SquirrelMonkey          AATAATCAAGATGTACATACTTTAAATGAAAATTCTAAAAACATTGAGCTGTTGCCACCT
Titi                    AATAATCAAGATGTACATGCTTTAAATGAAAATTCTAAAAACATTGAGCTGTTGCCACCT
Orangutan               AAGAATCAAGATATATGTGCTTCAAATGAAAATGATAAAAACGTTGAGCTGTTGCCACCT
CEMacaque               AAGAATCAAGATGTATGTGCTTTAAGTGAAAATTCTAAACATGTTGAGCTGTTGCCACCT
Talapoin                AAGAATCAAGATGTATGTGCTTTAAGTGAAAATTCTAAAAATGTTGAGCTGTTGCCACCT
WhiteHandedGibbon       AAGAATCAAGATGTATGTGCTTTAAATGAAAATTCTAAAAATGTTGAGCTGTTGCCACCT
Siamang                 AAGAATCAAGATGTATGTGCTTTAAATGAAAATTCTAAAAATGTTGAGCTGTTGCCACCT
WhiteCheekedGibbon      AAGAATCAAGATGTATGTGCTTTAAATGAAAATTCTAAAAATGTTGAGCTTTTGCCACCT
Colobus                 AAGAATCAAGATGTATGTGCTTTAAGTGAAAATTCTAAAAATGTTGAGCTATTGCCACCT
OliveBaboon             AAGAATCAAGATGTATGTGCTTTAAGTGAAAATTCTAAACATGTTGAGCTGTTGCCACCT
Marmoset                AATAATCAAGATGTACACGCCTTAAATGAAAATTCTAAAAACATTGAGCTGTTGCCACCC
                        ** ********* **    * * ** *******  **** *  ****  * ******** 

Bonobo                  GAAAAATACATGAGAGTTGCATCACCTTCAAGAAAGGTACAATTCAACCAAAACACAAAT
BorneoOrangutan         GAAAAATACATAAGAGTAGCATCACCTTCAAGAAAGGTCCAGTTCAACCAAAACACAAAT
Chimpanzee              GAAAAATACATGAGAGTAGCATCACCTTCAAGAAAGGTACAATTCAACCAAAACACAAAT
RedCheekedGibbon        GAAAAATACATAAGAGTAGCATCACCTTCAAGAAAGGTACAATTCAACCAAAACACAAAT
Human                   GAAAAATACATGAGAGTAGCATCACCTTCAAGAAAGGTACAATTCAACCAAAACACAAAT
AgileGibbon             GAAAAATACATAAGAGTAGCATCACCTTCAAGAAAGGTACAATTCAACCAAAACACAAAT
Gorilla                 GAAAAATACATGAGAGTAGCATCACCTTCAAGAAAGGTACAATTCAACCAAAACACAAAT
Howler                  GAAAAACACATAACAGTAGCATCACCTTCAAAATATGTACAATTCAACCAAAACACAAAT
Rhesus                  GAAAAATACATAAGAGTAGCATCACCTTCAAGAAAGGTACAATTCAACCAAAACACAAAT
WolfsGuenon             GAAAAATACATAAGAGAAGCATCACCTTCAAGAAAGGTACAATTCAACCAAAACACAAAT
LeafMonkey              GAAAAATACATAAGAGTAGCATCACCTTCAAGAAAGGTACAATTCAACCAAAACACAAAT
BlackMangabey           GAAAAATACATAAGAGTAGCATCACCTTCAAGAAAGGTACAATTCAACCAAAACACAAAT
PileatedGibbon          GAAAAATACATAAGAGTAGCATCACCTTCAAGAAAGGTACAATTCAACCAAAACACAAAT
SquirrelMonkey          CAAAAATACATAATAGTCGCATCACCTTCAAGATATGTACAATTCAACCAAAACACAAAT
Titi                    GAAAAATACGTAACAGTAGCATCACCTTCAAGATATGTACAGTTCAACCAAAACACAAAT
Orangutan               GAAAAATACATAAGAGTAGCATCACCTTCAAGAAAGGTCCAGTTCAACCAAAACACAAAT
CEMacaque               GAAAAATACATAAGAGTAGCATCACCTTCAAGAAAGGTACAATTCAACCAAAACACAAAT
Talapoin                GAAAAATACATAAGAGTAGCATCACCTTCAAGAAAGGTACAATTCAACCAAAACACAAAT
WhiteHandedGibbon       GAAAAATACATAAGAGTAGCATCACCTTCAAGAAAGGTACAATTCAACCAAAACACAAAT
Siamang                 GAAAAATACATAAGAGTAGCATCACCTTCAAGAAAGGTACAATTCAACCAAAACACAAAT
WhiteCheekedGibbon      GAAAAATACATAAGAGTAGCATCACCTTCAAGAAAGGTACAATTCAACCAAAACACAAAT
Colobus                 GAAAAATACATAAGAGTAGCATCACCTTCAAGAAAGGTACAATTCAACCAAAACACAAAT
OliveBaboon             GAAAAATACATAAGAGTAGCATCACCTTCAAGAAAGGTACAATTCAACCAAAACACAAAT
Marmoset                GAAAAATACATAATAGTAGCATCACCTTCAAGATACGTACAGTTCAACCAAAACACAAAT
                         ***** ** * * **  ************* * * ** ** ******************

Bonobo                  CTAAGAGTAATCCAAAACAATCAAGAAGAAACTACTTCAATTTCAAAAATAACTGTCAAT
BorneoOrangutan         CTAAGAGTAATCCAAAAAAATCAAGAAGAAACTACTTCAATTTCAAAAATAACTGTCAAT
Chimpanzee              CTAAGAGTAATCCAAAACAATCAAGAAGAAACTACTTCAATTTCAAAAATAACTGTCAAT
```

```
RedCheekedGibbon        CTAAGAGTAATCCAAAAAGATCAAGAAGAAACTACTTTAATTTCAAAAATAACTGTCAAT
Human                   CTAAGAGTAATCCAAAAAAATCAAGAAGAAACTACTTCAATTTCAAAAATAACTGTCAAT
AgileGibbon             CTAAGAGTAATCCAAAAAGATCAAGAAGAAACTACTTTAATTTCAAAAATAACTGTCAAT
Gorilla                 CTAAGAGTAATCCAAAAAAATCAAGAAGAAACTACTTCAATTTCAAAAATAACTGTCAAT
Howler                  CTAGGAGTAATCCGAAAAGATCAAGAAGAAACTACTTTAATTTCAAAAATGACTGTCAAT
Rhesus                  CTAAGAGTAATCCAAAAAGATCAAGAAGAGACTACTTTAATTCCAAAAATAACTGTCAAT
WolfsGuenon             CTAAGAGTAATCCAAAAAGATCAAGAAGAAACTACTTTAATTCCAAAAATAACTGTCAAT
LeafMonkey              CTAAGAGTAATCCAAAAAGATCAAGAAGAAACTACTTTAATTCCAAAAATAACTGTCAAT
BlackMangabey           CTAAGAGTAATCCAAAAAGATCAAGAAGAGACTACTTTAATTCCAAAAATAACTGTCAAT
PileatedGibbon          CTAAGAGTAATCCAAAAAGATCAAGAAGAAACTACTTTAATTTCAAAAATAACTGTCAAT
SquirrelMonkey          CTAAGAGTAATCCAAAAACATCAAGAAGAAACTACTTTAATTTCAAAAATGAATGTCAAT
Titi                    CTAAGAGTAATCCAAAAAGATCAAGAAGAAACTACTTTAATTTCAAAAATGACTGTCAGT
Orangutan               CTAAGAGTAATCCAAAAAAATCAAGAAGAAACTACTTCAATTTCAAAAATAACTGTCAAT
CEMacaque               CTAAGAGTAATCCAAAAAGATCAAGAAGAGACTACTTTAATTCCAAAAATAACTGTCAAT
Talapoin                CTAAGAGTAATCCAAAAAGATCAAGAAGAAACTACTTTAATTCCAAAAATAACTGTCAAT
WhiteHandedGibbon       CTAAGAGTAATCCAAAAAGATCAAGAAGAAACTACTTTAATTTCAAAAATAACTGTCAAT
Siamang                 CTAAGAGTAATCCAAAAAGATCAAGAAGAAACTACTTTAATTTCAAAAATAACTGTCAAT
WhiteCheekedGibbon      CTAAGAGTAATCCAAAAAGATCAAGAAGAAACTACTTTAATTTCAAAAATAACTGTCAAT
Colobus                 CTAAGAGTAATCCAAAAAGATCAAGAAGAAACTACTTTAATTCCAAAAATAACTGTCAAT
OliveBaboon             CTAAGAGTAATCCAAAAAGATCAAGAAGAGACTACTTTAATTCCAAAAATAACTGTCAAT
Marmoset                CTAAGAGTAATCCAAAAAGATCAAGAAGAAACTACTTTAATTTCAAAAATGACTGTCAGT
                        *** ********* ***  ********** ******* **** ******* * ***** *

Bonobo                  CCAGACTCTGAAGAACTTTTCTCAGACAATGAGAATAATTTTGTCTTCCAAGTAGCTAAT
BorneoOrangutan         CCAGACTCTGAAGAACTTTTCTTAGACATTGAGAATGATTTTGTCTTCCAAGTAGCTAAT
Chimpanzee              CCAGACTCTGAAGAACTTTTCTCAGACAATGAGAATAATTTTGTCTTCCAAGTAGCTAAT
RedCheekedGibbon        CCAGACTCTGAAGAACATTTCTCAGACAATGAGAATAATTTTGTCTTCCAAGTAGCTAAT
Human                   CCAGACTCTGAAGAACTTTTCTCAGACAATGAGAATAATTTTGTCTTCCAAGTAGCTAAT
AgileGibbon             CCAGACTCTGAAGAACATTTCTCAGACAATGAGAATAATTTTGTCTTCCAAGTAGCTAAT
Gorilla                 CCAGACTCTGAAGAACTTTTCTCAGACAATGAGAATAATTTTGTCTTCCAAGTAGCTAAT
Howler                  CCAGACTCTGAAGAACTTTTGTCAGACAATGAGAATAATTTTGTCTTCCAAGTACCTAGT
Rhesus                  CCAGACTCTGAAGAACTTTTCTCAGACAATGAGAATAATTTTGTCTTCCAAGTAGCTAAT
WolfsGuenon             CCAGACTCTGAAGAACTTTTCTCAGACAATGAGAATAATTTTGTCTTCCAAGTAGCTAAT
LeafMonkey              CCAGACTCTGAAGAACTTTTCTCAGGCAATGAGAGTAATTTTGTCTTCCAAGTAGCTAAT
BlackMangabey           CCAGACTCTGAAGAACTTTTCTCGGACAATGAGAATAATTTTGTCTTCCAAGTAGCTAAT
PileatedGibbon          CCAGACTCTGAAGAACATTTCTCAGACAATGAGAATAATTTTGTCTTCCAAGTAGCTAAT
SquirrelMonkey          CCAGACTCTGAAGAACTTTTATCAGACAATGAGAATAATTTTGTCTTCCAAGTGGCTAGT
Titi                    CCAGACTCTGAAGAACTTTTATCAGACAATGAGAATAACTTTGTCTTCCAAGTAGCTAGT
Orangutan               CCAGACTCTGAAGAACTTTTCTTAGACATTGAGAATGATTTTGTCTTCCAAGTAGCTAAT
CEMacaque               CCAGACTCTGAAGAACTTTTCTCAGACAATGAGAATAATTTTGTCTTCCAAGTAGCTAAT
Talapoin                CCAGACTCTGAAGAACTTTTCTCAGACAATGAGAATAATTTTGTCTTCCAAGTAGCTAAT
WhiteHandedGibbon       CCAGACTCTGAAGAACATTTCTCAGACAATGAGAATAATTTTGTCTTCCAAGTAGCTAAT
Siamang                 CCAGACTCTGAAGAACATTTCTCAGACAATGAGAATAATTTTGTCTTCCAAGTAGCTAAT
WhiteCheekedGibbon      CCAGACTCTGAAGAACATTTCTCAGACAATGAGAATAATTTTGTCTTCCAAGTAGCTAAT
Colobus                 CCAGACTCTGAAGAACTTTTCTCAGGCAATGAGAATAATTTTGTCTTCCAAGTAGCTAAT
OliveBaboon             CCAGACTCTGAAGAACTTTTCTCGGACAATGAGAATAATTTTGTCTTCCAAGTAGCTAAT
Marmoset                CCAGACTCTGAAGAACTTTTATCAGACAATGAGAATAATTTTGTCTTCCAAGTAGCTAGT
                        **************** *** *  * ** ***** * * **************  *** *

Bonobo                  GAAAGAAATAATCTTGCTTTAGGAAATACTAAGGAACTTCATGAAACAGACTTGACTTGT
BorneoOrangutan         GAAAGGAATAATCTTGCTTTAGGAAATACTAAGGAACTTCATGAAACAGACTTGACTTGT
Chimpanzee              GAAAGAAATAATCTTGCTTTAGGAAATACTAAGGAACTTCATGAAACAGACTTGACTTGT
RedCheekedGibbon        GAAAGGAATAATCTTGCTTTAGGAAATACTAAGGAACTTCATGAAACAGACTTGACTTGT
Human                   GAAAGGAATAATCTTGCTTTAGGAAATACTAAGGAACTTCATGAAACAGACTTGACTTGT
AgileGibbon             GAAAGGAGTAATCTTGCTTTAGGAAATACTAAGGAACTTCATGAAACAGACTTGACTTGT
Gorilla                 GAAAGGAATAATCTTGCTTTAGGAAATACTAAGGAACTTCATGAAACAGACTTGACTTGT
Howler                  GAAAGGAATAATTTTGCTTTAGAAAATACTAAGGAACCTCATGAAGCAGACTTGACTTGT
Rhesus                  GAAAGGAATAATTTTGCTTTAGGAAATACTAAGGAACTTCATGAAACAGACTTGACTTGT
WolfsGuenon             GAAAGGAATAATTTTGCTTCAGGAAATACTAAGGAACTTCATGAAACAGACTTGACTTGT
LeafMonkey              GAAAGGAATAATTTTGCTTTAGGAAATACTAACGAACTTCTTGAAACAGACTTGACTTGT
BlackMangabey           GAAAGGAATAATTTTGCTTTAGGAAATACTAAGGAACTTCATGAAACAGACTTGACTTGT
PileatedGibbon          GAAAGGAGTAATCTTGCTTTAGGAAATACTAAGGAACTTCATGAAACAGACTTGACTTGT
SquirrelMonkey          AAAAGGAATAATTTTGCTTTGGAAAATACTAAGGAACTTCATGAAGCGGACTTGACTTGT
Titi                    GAAAGGAATAATTTTGCTTTAGAAAATACTAAGGAACTTCATGAAGCAGATTTGATTTGT
Orangutan               GAAAGGAATAATCTTGCTTTAGGAAATACTAAGGAACTTCATGAAACAGACTTGACTTGT
CEMacaque               GAAAGGAATAATTTTGCTTTAGGAAATACTAAGGAACTTCATGAAACAGACTTGACTTGT
Talapoin                GAAAGGAATAATTTTGCTTTAGGAAATACTAAGGAACTTCATGAAACAGACTTGACTTGT
WhiteHandedGibbon       GAAAGGAGTAATCTTGCTTTAGGAAATACTAAGGAACTTCATGAAACAGACTTGACTTGT
Siamang                 GAAAGGAATAATCTTGCTTTAGGAAATACTAAGGAACTTCATGAAACAGACTTGACTTGT
WhiteCheekedGibbon      GAAAGGAATAATCTTGCTTTAGGAAATACTAAGGAACTTCATGAAACAGACTTGACTTGT
Colobus                 GAAAGGAATAATTTTGCTTTAGGAAATACTAAGGAACTCCTTGAAACAGACTTGACTTGT
OliveBaboon             GAAAGGAATAATTTTGCTTTAGGAAATACTAAGGAACTTCATGAAACAGACTTGACTTGT
Marmoset                GAAAGGAATAATTTTGCTTTAGAAAGTACTGAGGAACTTCATGAAGCAGACTTGACTTGT
```

```
                         **** * **** ******  * ** **** * ****  * **** * ** **** ****

Bonobo                  GTAAACAAACCCATTTTCAAGAACTCTACCATGGTTTTATATGGAGACACAGGTGATAAA
BorneoOrangutan         GTAAACGAACCCATTTTCAAGAACTCTACCATGGTTTTATATGGAGACATAGGTGATAAA
Chimpanzee              GTAAACGAACCCATTTTCAAGAACTCTACCATGGTTTTATATGGAGACACAGGTGATAAA
RedCheekedGibbon        GTAAACGAACCCATTTTCAAGAACTCTACCGTGGTTTTATATGCAGACATAGGTGATAAA
Human                   GTAAACGAACCCATTTTCAAGAACTCTACCATGGTTTTATATGGAGACACAGGTGATAAA
AgileGibbon             GTAAACGAACCCATTTTCAAGAACTCTACCGTGGTTTTATATGCAGACATAGGTGATAAA
Gorilla                 GTAAACGAACCCATTTTCAAGAACCCTACCATGGTTTTATATGGAGACACAGGTGATAAA
Howler                  GTAAACAAACCCATTTTCAAGAACTCTACCATGGTATTATATGCAGACATAGTTGATAAA
Rhesus                  GTAAATGAACCCATTTTCAAGAACTCTACCATGGTTTTATATGCAGACATAGGTGATAAA
WolfsGuenon             GTAAACGAACCCATTTTCAAGAACTCTACCATGGTTTTATATGCAGACATAGGTGATAAA
LeafMonkey              GTAAACGAACCCGTTTTCAAGAACTCTACCATGGTTTTATATGCAGACATAGGTGATAAA
BlackMangabey           GTAAATGAACCCATTTTCAAGAACTCTACCATGGTTTTATATGCAGACATAGGTGATAAA
PileatedGibbon          GTAAACGAACCCATTTTCAAGAACTCTACCGTGGTTTTATATGCAGACATAGGTGATAAA
SquirrelMonkey          GTAAACAAACCCGCTTTCAAGAACTCTACCATGGTATTATATGCAGACATAGTTGATAAA
Titi                    GTAAACAAACCCATTTTCAAGAACTCTACCATGGTATTATATGCAGACATAGTTGATAAA
Orangutan               GTAAACGAACCCATTTTCAAGAACTCTACCATGGTTTTATATGGAGACATAGGTGATAAA
CEMacaque               GTAAATGAACCCATTTTCAAGAACTCTACCATGGTTTTATATGCAGACATAGGTGATAAA
Talapoin                GTAAACGAACCCATTTTCAAGAGCTCTACCATGGTTTTATATGCAGACATAGGTGATAAA
WhiteHandedGibbon       GTAAACGAACCCATTTTCAAGAACTCTACCATGGTTTTATATGCAGACATAGGTGATAAA
Siamang                 GTAAACGAACCCATTTTCAAGAACTCTACCGTGGTTTTATATGCAGACACAGGTGATAAA
WhiteCheekedGibbon      GTAAACGAACCCATTTTCAAGAACTCTACCGTGGTTTTATATGCAGACATAGGTGATAAA
Colobus                 GTAAACGAACCCATTTTCCAGAACTCTACCATGGTTTTATATGCAGACATAGGTGATAAA
OliveBaboon             GTAAATGAACCCATTTTCAAGAACTCTACCATGGTTTTATATGCAGACATAGGTGATAAA
Marmoset                GTAAACAAACCCAATTTTGAGAACTCTACCATGGTATTACATGCAGACATAGTTGATAAA
                        *****  *****  ***  *** * ***** **** *** *** ***** ** *******

Bonobo                  CAAGCAACCCAAGTGTCAATTAAAAAAGATTTG---------------GTTTATGTTCTT
BorneoOrangutan         CAAGCAACCCAAGTGTCAATTAAAAAAGATTTG---------------GTTTATGTTCTT
Chimpanzee              CAAGCAACCCAAGTGTCAATTAAAAAAGATTTG---------------GTTTATGTTCTT
RedCheekedGibbon        CAAGCAACCCAAGTGTCAACTAAAAAAGATTTG---------------GTTTATGATTTT
Human                   CAAGCAACCCAAGTGTCAATTAAAAAAGATTTG---------------GTTTATGTTCTT
AgileGibbon             CAAGCAACCCAAGTGTCAACTAAAAAAGATTTG---------------GTTTATGATTTT
Gorilla                 CAAGCAACCCAAGTGTCAATTAAAAAAGATTTG---------------GTTTATGTTCTT
Howler                  CAAGCAACCCAAGTGTCAATTAAAAAAGATTTAGACTCGTCAAATACAGTTTATGATCTT
Rhesus                  CAAGCAACCCAAGTGTCGATTAAAAAAGATTTGGACTCATCAAATACAGTTTATGATCTT
WolfsGuenon             CAAGCAACCCAAGTGTCGATTAAAAAAGATTTGGACTCATCAAATATAGTTTATGATCTT
LeafMonkey              CAAGCAACCCAAGTGTCGATTATGAAAGATTTGGACTCATCAAATATAGTTTATGATCTT
BlackMangabey           CAAGCAACCCAAGTGTCGATTAAAAAAGATTTGGACTCATCAAATATAGTTTATGATCTT
PileatedGibbon          CAAGCAACCCAAGTGTCAACTAAAAAAGATTTG---------------GTTTATGATTTT
SquirrelMonkey          CAAGCAACCCAAGTGTCAATTAAAAAGGATTTAGACTCATCAAATATAGTTTATGATCTT
Titi                    CAAGCAACCAAAGTGTCAATTAAAAAAGATTTAGACTCATCAAATATAGTTTATGATCTT
Orangutan               CAAGCAACCCAAGTGTCAATTAAAAAAGATTTG---------------GTTTATGTTCTT
CEMacaque               CAAGCAACCCAAGTGTCGATTAAAAAAGATTTGGACTCATCAAATATAGTTTATGATCTT
Talapoin                CAAGCAACCCAAGTGTCGATTAAAAAAGATTTGGACTCATCAAATATAGTTTATGATCTT
WhiteHandedGibbon       CAAGCAACCCAAGTGTCAACTAAAAAAGATTTG---------------GTTTATGATTTT
Siamang                 CAAGCAACCCAAATGTCAACTAAAAAAGATTTG---------------GTTTATGATTTT
WhiteCheekedGibbon      CAAGCAACCCAAGTGTCAACTAAAAAAGATTTG---------------GTTTATGATTTT
Colobus                 CAAGCAACTCAAGTGTCGATTATGAAAGATTTGGACTCATCAAATATAGTTTATGATCTT
OliveBaboon             CAAGCAACCCAAGTGTCGATTAAAAAAGATTTGGACTCATCAAATATAGTTTATGATCTT
Marmoset                CAAGCAACCCAAGTGTCAATTAAAAAAGATTTAGACTCATCAAATAAAGTTTATTATCTT
                        ********  ** **** * **  ** *****                ******  * **

Bonobo                  GCAGAGGAGAACAAAAATAATGTAAAGCAGCATATAAAAATGACCCTAGGTCAAGATTTA
BorneoOrangutan         GCAGAGGAGAACAAAAATAGTGTAAAGCAGCATATAAAAATGACTCTAGGTCAAGATTTA
Chimpanzee              GCAGAGGAGAACAAAAATAGTGTAAAGCAGCATATAAAAATGACTCTAGGTCAAGATTTA
RedCheekedGibbon        GCGGAGGAGAACGAAAATAGCGTAAAGCAGCATCTAAAAATGACTCTAGGTCAAGATTTA
Human                   GCAGAGGAGAACAAAAATAGTGTAAAGCAGCATATAAAAATGACTCTAGGTCAAGATTTA
AgileGibbon             GCGGAGGAGAACGAAAACAGCGTAAAGCAGCATCTAAAAATGACTCTAGGTCAAGATTTA
Gorilla                 GCAGAGGAGAACAAAAATAGTGTAAAGCAGCATATAAAAATGACTCTAGGTCAAGATTTA
Howler                  GCAGAGGAGAAAAAAAATAGTGTAAAGCAGCATCTAGAAATGACTCTAGGTCAAGATTTA
Rhesus                  GCAGAGGAGAACAAAAATAGTGTAAAGCAGCATCTAAAAATGACTCTAGGTCAAGATTTA
WolfsGuenon             GCAGAGGAGAACAAAAATAGTGTAAAGCAGCATCTAAAAATGACTCTAGGTCAAGATTTA
LeafMonkey              GCAGAGGAGAACAAAAATAGTGTAAAGCAGCATCTAAAAATGACTCTAGGTCAAGATTTA
BlackMangabey           GCAGAGGAGAACAAAAATAGTGTAAAGCAGCATCTAAAAATCACTCTAGGTCAAGATTTA
PileatedGibbon          GCGGAGGAGAACGAAAACAGCGTAAAGCAGCATCTAAAAATGACTCTAGGTCAAGATTTA
SquirrelMonkey          TCAGAAGAGAAAAAAAATAGTGTGAAGCAGCATCTAGAAATGACTCTAGGTCAAGATTCA
Titi                    GAAGAGGAGAAAAAAAATAGTGTAAAGCAGCATCTAGAAATGACTCTAGGTCAAGATTTA
Orangutan               GCAGAGGAGAACAAAAATAGTGTAAAGCAGCATATAAAAATGACTCTAGGTCAAGATTTA
CEMacaque               GCAGAGGAGAACAAAAATAGTGTAAAGCAGCATCTAAAAATGACTCTAGGTCAAGATTTA
Talapoin                GCAGAGGAGAACAAAAATAGTGTAAAGCAGCATCTAAAAATGACTCTAGCTCAGGATTTA
WhiteHandedGibbon       GCGGAGGAGAACGAAAACAGCGTAAAGCAGCATCTAAAAATGACTCTAGGTCAAGATTTA
```

```
Siamang                 GCGGAGGAGAACGAAAATAGCGTAAAGCAGCATCTAAAAATGACTCTAGGTCAAGATTTA
WhiteCheekedGibbon      GCGGAGGAGAACGAAAATAGCGTAAAGCAGCATCTAAAAATGACTCTAGGTCAAGATTTA
Colobus                 GCAGAGGAGAACAAAAATAGTGTAAAGCAGCATCTAAAAATGACTCTAGGTCAAGATTTA
OliveBaboon             GCAGAGGAGAACAAAAATAGTGTAAAGCAGCATCTAAAAATCACTCTAGGTCAAGATTTA
Marmoset                GCAGAGGAGAAAAAAAATAGTGTAAAGCAGCATCTAGAAATGACTCTAGGTCAAGATATA
                           ** *****  **** *  ** ********* ** **** ** **** *** ***  *

Bonobo                  AAATCGGACATCTCCTTGAATATAGATAAAATACCAGACAAAAATAATGATTACATGGAC
BorneoOrangutan         AAATCGGACGTC---TTGAATATAGATAAAATACCAGACAAAAATAATGATTACATGGAC
Chimpanzee              AAATCGGACATCTCCTTGAATATAGATAAAATACCAGACAAAAATAATGATTACATGGAC
RedCheekedGibbon        AAATCGGACATCTCCTTGAATATAGATAAAATACCAGACAAAAATAATGATTACATGGAC
Human                   AAATCGGACATCTCCTTGAATATAGATAAAATACCAGAAAAAAATAATGATTACATGAAC
AgileGibbon             AAATCGGACATC---TTGAATATAGATAAAATACCAGACAAAAATAATGATTACATGGAC
Gorilla                 AAATCGGACATCTCCTTGAATATAGATAAAATACCAGACAAAAATAATGATTACATGGAC
Howler                  AAATCAGATATCTCCTTGAATATAGATAAAATACCAAACAAAAATAATGATTACAAGGAC
Rhesus                  CAACCGGACATC---TTGAATATAGATAAAATTCCAGACAAAAATAATGATTGCATGGAC
WolfsGuenon             CAACCGGACATC---TTGAATATAGATAAAATTCCAGACAAAAATAATGATTGCATGGAC
LeafMonkey              CAACCGGACATCTCCTTGAATATAGATAAAATACCAGACAAAAATAATGATTGCATGGAC
BlackMangabey           CAACTGGACATC---TTGAATATAGATAAAATTCCAGACAAAAATAATGATTGCATGGAC
PileatedGibbon          AAATCGGACATC---TTGAATATAGATAAAATACCAGACAAAAATAATGATTACATGGAC
SquirrelMonkey          AAATCAGGCATCTCCTTGAATATAGATAAAATACCAAACAAAAATAATGATTACAAGGAC
Titi                    AAATCAGACATCTCCTTGAATATAGATAAAATACCAAACAAAAATAATGATTACAAGGAC
Orangutan               AAATCGGACGTC---TTGAATATAGATAAAATACCAGACAAAAATAATGATTACATGGAC
CEMacaque               CAACCGGACATC---TTGAATATAGATAAAATTCCAGACAAAAATAATGATTGCATGGAC
Talapoin                CAACCGGACATC---TTGAATATAGATAAAATACCAGACAAAAATAATGATTGCATGGGC
WhiteHandedGibbon       AAATCGGACATC---TTGAATATAGATAAAATACCAGACAAAAATAATGATTACATGGAC
Siamang                 AAATCGGACATCTCCTTGAATATAGATAAAATACCAGACAAAAATAATGATTACATGGAC
WhiteCheekedGibbon      AAATCGGACATCTCCTTGAATATAGATAAAATACCAGACAAAAATAATGATTACATGGAC
Colobus                 CAACCGGACATCTCCTTGAATATAGATAAAATACCAGACAAAAATAATGATTGCATGGAC
OliveBaboon             CAACCGGACATC---TTGAATATAGATAAAATTCCAGACAAAAATAATGATTGCATGGAC
Marmoset                AAATCAGACATCTCCTTGAATATAGATAAAATACCAAACAAAAATAATGATTACAAGGAC
                         **   *   **   ***************** *** * ************* ** *  *

Bonobo                  AAATGGGCAGGACTCTTAGGTCCAATTTCAAATCACAGCTTTGGAGGTAGCTTCAGAACA
BorneoOrangutan         AAATCGGCAGGACTCTTAGGTCCAATTTCAAATCACAGTTTTGGAGGTGGCTTCAGAACA
Chimpanzee              AAATGGGCAGGACTCTTAGGTCCAATTTCAAATCACAGCTTTGGAGGTAGCTTCAGAACA
RedCheekedGibbon        AAATGGGCAGGACTCTTGGGTCCAATTTCAAATCACAGTTTTGGAGGTAGCTTCAGAACA
Human                   AAATGGGCAGGACTCTTAGGTCCAATTTCAAATCACAGTTTTGGAGGTAGCTTCAGAACA
AgileGibbon             AAATGGGCAGGACTCTTGGGTCCAATTTCAAATCACAGTTTTGGAGGTAGCTTCAGAACA
Gorilla                 AAATGGGCAGGACTCTTAGGTCCAATTTCAAATCACAGTTTTGGAGGTAGCTTCAGAACA
Howler                  AAATGGGCAGGGCTCTTACGTCCAATTTCAAATCACAGTTTTGAAGGTAGGTTCAGAACA
Rhesus                  AAATGGGCAGGACCCTTAGATCCAATTTCAAATCACAGTTTTGGAGGTACTTTCAGAACA
WolfsGuenon             AAATGGGCAGGACCCTTAGATCCAATTTCAAATCACAGTTTTGGAGGTAGCTTCAGAACA
LeafMonkey              AAATGGGCAGGACCCTTAGATCCAATTTCAAATCACAGTTTTGGAGGTAGCTTCAGAACA
BlackMangabey           AAATGGGCAGGACCCTTAGATCCAATTTCAAATCACAGTTTTGGAGGTAGCTTCAGAACA
PileatedGibbon          AAATGGGCAGGACTCTTGGGTCCAATTTCAAATCACAGTTTTGGAGGTAGCTTCAGAACA
SquirrelMonkey          AAATGGGCAGGACTCTTACATCCAATTTCAAATCACAGTTTTGGGGGTAGCTTCAGAACA
Titi                    AAATGGGCAGGACTTTTACATCCAATTTCAAATCACAGTTTTGGAGGTAGCTTCAGAACA
Orangutan               AAATCGGCAGGACTCTTAGGTCCAATTTCAAATCACAGTTTTGGAGGTGGCTTCAGAACA
CEMacaque               AAATGGGCAGGACCCTTAGATCCAATTTCAAATCACAGTTTTGGAGGTTCTTTCAGAACA
Talapoin                AAATGGGCAGGACCCTTAGATCCAATTTCAAATCACAGTTTTGGAGGTAGCTTCAGAACA
WhiteHandedGibbon       AAATGGGCAGGACTCTTGGGTCCAATTTCAAATCACAGTTTTGGAGGTAGCTTCAGAACA
Siamang                 AAATGGGCAGGACTCTTGGGTCCAATTTCAAATCACAGTTTTGGAGGTAGCTTCAGAACA
WhiteCheekedGibbon      AAATGGGCAGGACTCTTGGGTCCAATTTCAAATCACAATTTTGGAGGTAGCTTCAGAACA
Colobus                 AAATGGGCAGGACCCTTAGATCCAATTTCAAATCACAGTTTTGGAGGTAGCTTCAGAACA
OliveBaboon             AAATGGGCAGGACCCTTAGATCCAATTTCAAATCACAGTTTTGGAGGTAGCTTCAGAACA
Marmoset                AAATGGGCAGGACTCTTACATCCAATTTCAAATCACAGTTTTGGAGGTAGCTTCAGAACA
                        **** ****** *  **   *****************  ****  ***   *********

Bonobo                  GCTTCAAATAAGGAAATCAAGCTCTCTGAACATAACATTAAGAAGAGCAAAATGTTCTTC
BorneoOrangutan         GCTTCAAATAAGGAAATCAAGCTCTCTGAACATAACATTAAGAAGAGCAAAATGTTCTTC
Chimpanzee              GCTTCAAATAAGGAAATCAAGCTCTCTGAACATAACATTAAGAAGAGCAAAATGTTCTTC
RedCheekedGibbon        GCTTCAAATAAGGAAATCAAGCTCTCTGAACGTAACATTAAGAAGAGCAAAATGTTCTTC
Human                   GCTTCAAATAAGGAAATCAAGCTCTCTGAACATAACATTAAGAAGAGCAAAATGTTCTTC
AgileGibbon             GCTTCAAATAAGGAAATCAAGCTCTCTGAACGTAACATTAAGAAGAGCAAAATGTTCTTC
Gorilla                 GCTTCAAATAAGGAAATCAAGCTCTCTGAACATAACATTAAGAAGAGCAAAATGTTCTTC
Howler                  GCTTCAAATAAGGAAATCAAGCTCTCTGAACATAACATTAAGAAAAGCAAAATGCTCTTC
Rhesus                  GCTTCAAATAAGGAAATCAAGGTCTCTGAACATAACATTAAGAAGAGCAAAATGTTCTTC
WolfsGuenon             GCTTCAAATAAGGAAATCAAGGTCTCTGAACATAACATTAAGAAGAGCAAAATGTTCTTC
LeafMonkey              GCTTCAAATAAGGAAATCAAGCTCTCTGAACATAACATTAAGAAGAGCAAAATGTTCTTC
BlackMangabey           GCTTCAAATAAGGAAATCAAGGTCTCTGAACATAACATTAAAAAGAGCAAAATGTTCTTC
PileatedGibbon          GCTTCAAATAAGGAAATCAAGCTCTCTGAACGTAACATTAAGAAGAGCAAAATGTTCTTC
SquirrelMonkey          GCTTCAAATAAGGAAATCAAGCTCTCTGAACATAACATTAAGAAAAGCAAAATGCTCTTC
```

```
Titi                    GCTTCAAATAAGGAAATCAAGCTCTCTGAACATAACATTAAGAAAAGCAAAATGCTCTTC
Orangutan               GCTTCAAATAAGGAAATCAAGCTCTCTGAACATAACATTAAGAAGAGCAAAATGTTCTTC
CEMacaque               GCTTCAAATAAGGAAATCAAGGTCTCTGAACATAACATTAAGAAGAGCAAAATGTTCTTC
Talapoin                GCTTCAAATAAGGAAATCAAGCTCTCTGAACATAACATTAAGAAGAGCAAAATGTTCTTC
WhiteHandedGibbon       GCTTCAAATAAGGAAATCAAGCTCTCTGAACGTAACATTAAGAAGAGCAAAATGTTCTTC
Siamang                 GCTTCAAATAAGGAAATCAAGCTCTCTGAACGTAACATTAAGAAGAGCAAAATGTTCTTC
WhiteCheekedGibbon      GCTTCAAATAAGGAAATCAAGCTCTCTGAACGTAACATTAAGAAGAGCAAAATGTTCTTC
Colobus                 GCTTCAAATAAGGAAATCAAGCTCTCTGAACATAACATTAAGAAGAGCAAAATGTTCTTC
OliveBaboon             GCTTCAAATAAGGAAATCAAGGTCTCTGAACATAACATTAAAAAGAGCAAAATGTTCTTC
Marmoset                GCTTCAAATAAGGAAATCAAGCTCTCTGAACATAACATTAAGAAAAGCAAAATGCTCTTC
                        ********************* ********* ********* ** ********* *****

Bonobo                  AAAGATATTGAAGAACAATATCCTACTAGTTTAGCTTGTGTTGAAATTGTAAATACCTTG
BorneoOrangutan         AGAGATATTGAAGAACAATATCCTACTAGTTTAGCTTGTGTTGAAATTGTAAATACCTTG
Chimpanzee              AAAGATATTGAAGAACAATATCCTACTAGTTTAGCTTGTGTTGAAATTGTAAATACCTTG
RedCheekedGibbon        AAAGATATTGAAGAACAATATCCTACTAGTTTAGCTTGTGTTGAAATTGTAAATACCTTG
Human                   AAAGATATTGAAGAACAATATCCTACTAGTTTAGCTTGTGTTGAAATTGTAAATACCTTG
AgileGibbon             AAAGATATTGAAGAACAATATCCTACTAGTTTAGCTTGTGTTGAAATTGTAAATACCTTG
Gorilla                 AAAGATATTGAAGAACAATATCCTACTAGTTTAGCTTGTGTTGAAATTGTAAATACCTTG
Howler                  AGAGATATCGAAGAACAATATCCTACTAGTTTAGTTTGTGTTGAAATTGTAAATACCTTG
Rhesus                  AAAGATATTGAAGAACAGTATCCTACTAGTTTAGCTTGTGTTGAAATTGTAAATACCTTG
WolfsGuenon             AAAGATATTGAAGAACAGTATCCTACTAGTTTAGCTTGTGTTGAAATTGTAAATACCTTG
LeafMonkey              AAAGATATTGAAGAACAGTATCCTACTAGTTTAGCTTGTGTTGAAATTGTAAATACCTTG
BlackMangabey           AAAGATATTGAAGAACAGTATCCTACTAGTTTAGCTTGTGTTGAAATTGTAAATACCTTG
PileatedGibbon          AAAGATATTGAAGAACAATATCCTACTAGTTTAGCTTGTGTTGAAATTGTAAATACCTTG
SquirrelMonkey          AGAGATATCGAAGAACAGTATCCTACTAGTTTAGCTTGTATTGAAATTGTAAATACCTTG
Titi                    AGAGATATCGAAGAACAATATCCTACTAGTTTAGCTTGTGTTGAAATTGTAAATACCTTG
Orangutan               AGAGATATTGAAGAACAATATCCTACTAGTTTAGCTTGTGTTGAAATTGTAAATACCTTG
CEMacaque               AAAGATATTGAAGAACAGTATCCTACTAGTTTAGCTTGTGTTGAAATTGTAAATACCTTG
Talapoin                AAAGATATTGAAGAACAGTATCCTACTAGTTTAGCTTGTGTTGAAATTATAAATACCTTG
WhiteHandedGibbon       AAAGATATTGAAGAACAATATCCTACTAGTTTAGCTTGTGTTGAAATTGTAAATACCTTG
Siamang                 AAAGATATTGAAGAACAATATCCTACTAGTTTAGCTTGTGTTGAAATTGTAAATACCTTG
WhiteCheekedGibbon      AAAGATATTGAAGAACAATATCCTACTAGTTTAGCTTGTGTTGAAATTGTAAATACCTTG
Colobus                 AAAGATATTGAAGAAGAGTATCCTACTAGTTTAGCTTGTGTTGAAATTGTAAATACCTTG
OliveBaboon             AAAGATATTGAAGAACAGTATCCTGCTAGTTTAGCTTGTGTTGAAATTGTAAATACCTTG
Marmoset                AGAGATATCGAAGAACAATATCCTACTAGTTCAGCTTGTGTTGAAATAGTAAATACCTTG
                        * ****** ****** * ****** ****** ** **** *******  ***********

Bonobo                  GCATTAGATAATCAAAAGAAACTGAGCAAGCCTCAGTCAATTAATACTGTATCTGCACAT
BorneoOrangutan         GCATTAGATAATCAAAAGAAACTGAGCAAGCCTCAGTCAATTAATACTGTATCTGCACAT
Chimpanzee              GCATTAGATAATCAAAAGAAACTGAGCAAGCCTCAGTCAATTAATACTGTATCTGCACAT
RedCheekedGibbon        GCATTAGATAATCAAAAGAAACTGAGCAAGCCTCAGTCAATTAATACTGTATCTGCACAT
Human                   GCATTAGATAATCAAAAGAAACTGAGCAAGCCTCAGTCAATTAATACTGTATCTGCACAT
AgileGibbon             GCATTAGATAATCAAAAGGAACTGAGCAAGCCTCAGTCAATTAATACTGTATCTGCACAT
Gorilla                 GCATTAGATAATCAAAAGAAACTGAGCAAGCCTCAGTCAATTAATACTGTATCTGCACAT
Howler                  GCATTAGATAATCAAAAGAAACTAAGCAAGCCTCAGTCAATCAAGACTGTATCTGCACAT
Rhesus                  GCGTTAGATAATCAAAAGAAACTGAGCAAGCCTCAGTCAATTAATACTGTATCTGCACAT
WolfsGuenon             GCGTTAGATAATCAAAAGAAACTGAGCAAGCCTCAGTCAATTAATACTGTATCTGCACAT
LeafMonkey              GCGTTAGATAAGCAAAAGAAACTGAGCAAGCCTCAGTCAATTAATACTGTATCTGCACAT
BlackMangabey           GCGTTAGATAATCAAAAGAAACTGAGCAAGCCTCAGTCAATTAATACTGTATCTGCACAT
PileatedGibbon          GCATTAGATAATCAAAAGGAACTGAGCAAGCCTCAGTCAATTAATACTGTATCTGCACAT
SquirrelMonkey          GCATTAGATAATCAAAAGAAACTAAGCAAGCCTCAGTCAATTAAGCCTGTATCTGCACAT
Titi                    GCATTAGATAATCGAAAGAAACTAAGCAAGCCTGAGTCAATTAGGACTGTATCTGCACAT
Orangutan               GCATTAGATAATCAAAAGAAACTGAGCAAGCCTCAGTCAATTAATACTGTATCTGCACAT
CEMacaque               GCGTTAGATAATCAAAAGAAACTGAGCAAGCCTCAGTCAATTAATACTGTATCTGCACAT
Talapoin                GCGTTAGATAATCAAAAGAAACTGAGCAAGCCTCAGTCAATTAATACTGTATCTGCACAT
WhiteHandedGibbon       GCATTAGATAATCAAAAGGAACTGAGCAAGCCTCAGTCAATTAATACTGTATCTGCACAT
Siamang                 GCATTAGATAATCAAAAGGAACTGAGCAAGCCTCAGTCAATTAATACTGTATCTGCACAT
WhiteCheekedGibbon      GCATTAGATAATCAAAAGAAACTGAGCAAGCCTCAGTCAATTAATACTGTATCTGCACAT
Colobus                 GCGTTAGATAATCAAAAGAAACTGAGCAAGCCTCAATCAATTAATACTGTATCTGCACAT
OliveBaboon             GCATTAGATAATCAAAAGAAACTGAGCAAGCCTCAGTCAATTAATACTGTATCTGCACAT
Marmoset                GCATTAGATAATCAAAAGAAACTAAGCAAGCCTCAGTCAATTAAGACTGTATCTGCACAT
                        ** ******** * **** **** ********* * ***** *   **************

Bonobo                  TTACAGAGTAGTGTAGTTGTTTCTGATTGTAAAAATAGTCATATAACCCCTCAGATGTTA
BorneoOrangutan         TTACCGAGTAGTGTAGTTGTTTCTGATTGTAAAAATAGTCATATAACCCCTCAGATGTTA
Chimpanzee              TTACAGAGTAGTGTAGTTGTTTCTGATTGTAAAAATAGTCATATAACCCCTCAGATGTTA
RedCheekedGibbon        TTACAGAGTAGTGTA---GTTTCTGATTGTAAAAATAGTCATATAAACCCTCGGATGTTA
Human                   TTACAGAGTAGTGTAGTTGTTTCTGATTGTAAAAATAGTCATATAACCCCTCAGATGTTA
AgileGibbon             TTACAGAGTAGTGTA---GTTTCTGATTGTAAAAATAGTCATATAACCCCTCGGATGTTA
Gorilla                 TTACAGAGTAGTGTAGTTGTTTCTGATTGTAAAAATAGTCATATAACCCCTCAGATGTTA
Howler                  GTACAGAGTAGTGTAGTTGTTTCTGATTGTGAAAATAGTCTTACAACCCCTCAGATGTTA
Rhesus                  TTACAGAGTAGTGTAGTTGTTTCTGATTGTAAAAATAGTCATATAACCCCTCAGATGTCA
```

```
WolfsGuenon          TTACAGAGTAGTGTAGTTGTTTCTGATTGTAAAAATAGTCATATAACCCCTCAGATGTCA
LeafMonkey           TTACAGAGTAGTGTAGTTGTTTCTGATTGTAAAAATAGTCATATAACCCCTCAGATGTCA
BlackMangabey        TTACAGAGTAGTGTAGTTGTTTCTGATTGTAAAAATAGTCATATAACCCCTCAGATGTCA
PileatedGibbon       TTACAGAGTAGTGTA---GTTTCTGATTGTAAAAATAGTCATATAACCCCTCGGATGTTA
SquirrelMonkey       GTACAGAGTAGTGTAGTTGTTTCTGATTGTAAAAATAGTCTTACAACCCCTCAGATGTTA
Titi                 GTACAGAGTAGTGTAGTTGTTTCTGTTTGTAAAAATAGTCTTACAACCCCTCAGATGTTA
Orangutan            TTACCGAGTAGTGTAGTTGTTTCTGATTGTAAAAATAGTCATATAACCCCTCAGATGTTA
CEMacaque            TTACAGAGTAGTGTAGTTGTTTCTGATTGTAAAAATAGTCATATAACCCCTCAGATGTCA
Talapoin             TTACAGAGTAATGTAGTTGTTTCTGATTGTAAAAATAGTCATATAACCCCTCAGATGTCA
WhiteHandedGibbon    TTACAGAGTAGTGTA---GTTTCTGATTGTAAAAATAGTCATATAACCCCTCGGATGTTA
Siamang              TTACAGAGTAGTGTA---GTTTCTGATTGTAAAAATAGTCATATAAACCCTCGGATGTTA
WhiteCheekedGibbon   TTACAGAGTAGTGTA---GTTTCTGATTGTAAAAATAGTCATATAAACCCTCGGATGTTA
Colobus              TTACAGAGTAGTGTAGTTGTTTCTGATTATAAAAATAGTCATATAACCCCTCAGATGTCA
OliveBaboon          TTACAGAGTAGTGTAGTTGTTTCTGATTGTAAAAATAGTCATATAACCCCTCAGATGTCA
Marmoset             GTACAGAGTAGTGTAGTTGGTTTTGATTGTAAAAATAGTCTTACAACCCCTCAGATGTTA
                      *** ***** ****   * ** ** ** * ********* ** ** ***** ***** *

Bonobo               TTTTCAAAGCAGGATTTTAATTCAAACCATAATTTAACACCTAGCCAAAAGGCAGAAATT
BorneoOrangutan      TTTTCAAAGCAGGATTTTAATTCAAACCATAATTTAACACCTAGCCAAAAGGCAGAAATT
Chimpanzee           TTTTCAAAGCAGGATTTTAATTCAAACCATAATTTAACACCTAGCCAAAAGGCAGAAATT
RedCheekedGibbon     TTTTCAAAGCAGGATTTTAATTCAAACCATAATTTAACACCTAGCCAAAAGGCAGAAATT
Human                TTTTCCAAGCAGGATTTTAATTCAAACCATAATTTAACACCTAGCCAAAAGGCAGAAATT
AgileGibbon          TTTTCAAAGCAGGATTTTAATTCAAACCATAATTTAACACCTAGCCAAAAGGCAGAAATT
Gorilla              TTTTCAAAGCAGGATTTTAATTCAAACCATAATTTAACACCTAGCCAAAAGGCAGAAATT
Howler               TTTTCAAAGGAGGATTTTAATTCAAACCATAACTTAACACCTAGCCAAAAGGCAGAAATT
Rhesus               TTTTCAAAGCAGGATTTTAATTCAAACCATAACTTAACACCTAGCCAAAAGGCAGAAATT
WolfsGuenon          TTTTCAAAGCAGGATTTTAATTCAAACCATAACTTAACACCTAGCCAAAAGGCAGAAATT
LeafMonkey           TTTTCAAAGCAGGATTTTAATTCAAACCATAACTTAACACCTAGCCAAAAGGCAGAAATT
BlackMangabey        TTTTCAAAGCAGGATTTTAATTCAAACCATAACTTAACACCTAGCCAAAAGGCAGAAATT
PileatedGibbon       TTTTCAAAGCAGGATTTTAATTCAAACCATAATTTAACACCTAGCCAAAAGGCAGAAATT
SquirrelMonkey       TTTTCAAAGGAGGATTTTAATTCAAACCATAACTTAACACCTAGCCAAAAGGCAGAAATT
Titi                 TTTTCAAAGGAGGGTTTTAATTCAAACCATAACTTAACACCTAGCCAAAAGGCAGAAATT
Orangutan            TTTTCAAAGCAGGATTTTAATTCAAACCATAATTTAACACCTAGCCAAAAGGCAGAAATT
CEMacaque            TTTTCAAAGCAGGATTTTAATTCAAACCATAACTTAACACCTAGCCAAAAGGCAGAAATT
Talapoin             TTTTCAAAGCAGGATTTTAATTCAAACCATAACTTAACACCTAGCCAAAAGGCAGAAATT
WhiteHandedGibbon    TTTTCAAAGCAGGATTTTAATTCAAACCATAATTTAACACCTAGCCAAAAGGCAGAAATT
Siamang              TTTTCAAAGCAGGATTTTAATTCAAACCATAATTTAACACCTAGCCAAAAGGCAGAAATT
WhiteCheekedGibbon   TTTTCAAAGCAGGATTTTAATTCAAACCATAATTTAACACCTAGCCAAAAGGCAGAAATT
Colobus              TTTTCAAAGCAGGATTTTAATTCAAACCATAACTTAACACCTAGCCAAAAGGCAGAAATT
OliveBaboon          TTTTCAAAGCAGGATTTTAATTCAAACCATAACTTAACACCTAGCCAAAAGGCAGAAATT
Marmoset             TTTTCAAAGGAAGATTTTAATTCAAATCATAACTTAACACCTAGCCAAAAGGCAGAAATT
                     ***** *** * * ************ ***** ***************************

Bonobo               ACAGAACTTTCTACCATATTAGAAGAATCAGGAAGTCAGTTTGAATTTACTCAGTTTAGA
BorneoOrangutan      ACAGAACTTTCTACTATATTAGAAGAATCAGGAAGTCAGTTTGAATTTACTCAGTTTAGA
Chimpanzee           ACAGAACTTTCTACCATATTAGAAGAATCAGGAAGTCAGTTTGAATTTACTCAGTTTAGA
RedCheekedGibbon     ACAGAACTTTCTACTATGTTAGAAGAATCAGGAAGTCAGTTTGAATTTACTCAGTTTAGA
Human                ACAGAACTTTCTACTATATTAGAAGAATCAGGAAGTCAGTTTGAATTTACTCAGTTTAGA
AgileGibbon          ACAGAACTTTCTACTATGTTAGAAGAATCAGGAAGTCAGTTTGAATTTACTCAGTTTAGA
Gorilla              ACAGAACTTTCTACTATATTAGAAGAATCAGGAAGTCAGTTTGAATTTACTCAGTTTAGA
Howler               ACAGAACTTTCTACTATATTAGAAGAATCAGGAAGTCAGTTTGAATTTACACAGTTTAGA
Rhesus               ACAGAACTTTCTACTATATTAGAAGAATCGGGAAGTCAGTTTGAATTTACTCAGTTTAGA
WolfsGuenon          ACAGAACTTTCTACTATATTAGAAGAATCAGGAAGTCAGTTTGAATTTACTCAGTTTAGA
LeafMonkey           ACAGAACTTTCCACTATATTAGAAGAATCAGGAAGTCAGTTTGAATTTACTCAGTTTAGA
BlackMangabey        ACAGAACTTTCTACTATATTAGAAGAATCAGGAAGTCAGTTTGAATTTACTCAGTTTAGA
PileatedGibbon       ACAGAACTTTCTACTATGTTAGAAGAATCAGGAAGTCAGTTTGAATTTACTCAGTTTAGA
SquirrelMonkey       ACAGAACTTTCTACTATATTAGAAGAATCAGGAAGTCAGTTTGAATTTACACAGTTTAGA
Titi                 ACAGAACTTTCTACTATATTAGAAGAATCAGGAAGTCAGTTTGAATTTACACAGTTTAGA
Orangutan            ACAGAACTTTCTACTATATTAGAAGAATCAGGAAGTCAGTTTGAATTTACTCAGTTTAGA
CEMacaque            ACAGAACTTTCTACTATATTAGAAGAATCAGGAAGTCAGTTTGAATTTACTCAGTTTAGA
Talapoin             ACAGAACTTTCTACTATATTAGAAGAATCAGGAAGTCAGTTTGAATTTACTCAGTTTAGA
WhiteHandedGibbon    ACAGAACTTTCTACTATGTTAGAAGAATCAGGAAGTCAGTTTGAATTTACTCAGTTTAGA
Siamang              ACAGAACTTTCTACTATGTTAGAAGAATCAGGAAGTCAGTTTGAATTTACTCAGTTTAGA
WhiteCheekedGibbon   ACAGAACTTTCTACTATGTTAGAAGAATCAGGAAGTCAGTTTGAATTTACTCAGTTTAGA
Colobus              ACAGAACTTTCTACTATATTAGAAGAATCAGGAAGTCAGTTTGAATTTACTCAGTTTAGA
OliveBaboon          ACAGAACTTTCTACTATATTAGAAGAATCAGGAAGTCAGTTTGAATTTACTCAGTTTAGA
Marmoset             ACAGAACTTTCTACTATATTAGAAGAATCAGGAAGTCAGTTTGAATTTACACAGTATAGA
                     *********** ** ** *********** ******************** **** ****

Bonobo               AAACCAAGCTACATATTGCAGAAGAGTACATTTGAAGTGCCTGAAAACCAGATGACTATC
BorneoOrangutan      AAACCAAGCTACATATTGCAGAAGAATACATTTGAAGTGCCTGAAAACCAGATGACTATC
Chimpanzee           AAACCAAGCTACATATTGCAGAAGAGTACATTTGAAGTGCCTGAAAACCAGATGACTATC
RedCheekedGibbon     AAACCAAGCTACATATTGCAGAAGAATACATTTGAAGTGCCTGAAAACCAGATGACTATC
```

```
Human                   AAACCAAGCTACATATTGCAGAAGAGTACATTTGAAGTGCCTGAAAACCAGATGACTATC
AgileGibbon             AAACCAAGCTACATATTGCAGAAGAATACATTTGAAGTGCCTGAAAACCAGATGACTATC
Gorilla                 AAACCAAGCTACATATTGCAGAAGAGTACATTTGAAGTGCCTGAAAACCAGATGACTATC
Howler                  AAACCAAGCTACATATTGCAGAATAATACGTTTGAAGTACCTGAAAACCAGATGACTATC
Rhesus                  AAACCAAGCTACATATTGCAGAAGAATACATTTGAAGTGCCTGAAAACCAGGTGACTATC
WolfsGuenon             AAACCAAGCTACATATTGCAGAAGAATACATTTGAAGTGCCTGAAAACCAGGTGACTATC
LeafMonkey              AAGCCAAGCTACATATTGCAGAAGAATACATTTGAAGTGCCTGAAAACCAGGTGACTATC
BlackMangabey           AAACCAAGCTACATATTGCAGAAGAATACATTTGAAGTGCCTGAAAACCAGGTGACTATC
PileatedGibbon          AAACCAAGCTACATATTGCAGAAGAATACATTTGAAGTGCCTGAAAACCAGATGACTATC
SquirrelMonkey          AAACCAAGCTACATATTGCAGAATAATACGTTTGAAGTACCTGAAAACCAGATGACTATC
Titi                    AAACCAAGCTACATATTGCAGAATAATACGTTTGAAGTACCTAAAAACCAAGTGACTATC
Orangutan               AAACCAAGCTACATATTGCAGAAGAATACATTTGAAGTGCCTGAAAACCAGATGACTATC
CEMacaque               AAACCAAGCTACATATTGCAGAAGAATACATTTGAAGTGCCTGAAAACCAGGTGACTATC
Talapoin                AAACCAAGCTACATATTGCAGAAGAATACATTTGAAGTGCCTGAAAACCAGGTGACTATT
WhiteHandedGibbon       AAACCAAGCTACATATTGCAGAAGAATACATTTGAAGTGCCTGAAAACCAGATGACTATC
Siamang                 AAACCAAGCTACATATTGCAGAAGAATACATTTGAAGTGCCTGAAAACCAGATGACTATC
WhiteCheekedGibbon      AAACCAAGCTACATATTGCAGAAGAATACATTTGAAGTGCCTGAAAACCAGATGACTATC
Colobus                 AAACCAAGCTACATATTGCAGAAGAAGACATTTGAAGTGCCTGAAAACCAGGTGACTATC
OliveBaboon             AAACCAAGCTACATATTGCAGAAGAATACATTTGAAGTGCCTGAAAACCAGGTGACTATC
Marmoset                AAACCAAGCTACATATTGCAGAATAATATGTTTGAAGTACCTGAAAACCAGATGACTATC
                        ** ******************** *  *  ******** *** *******  *******

Bonobo                  TTAAAGACCACTTCTGAGGAATGCAGAGATGCTGATCTTCATGTCATAATGAATGCCCCA
BorneoOrangutan         TTAAATACCACTTCTGAGGAATGCAGAGATGCTGATCTTCATGTCATAATGAATGCCCCA
Chimpanzee              TTAAAGACCATTTCTGAGGAATGCAGAGATGCTGATCTTCATGTCATAATGAATGCCCCA
RedCheekedGibbon        TTAAATACCACTTCTGAGGAATGCAGAGATGCTGATCTTCATGTCATAATGAATGCCCCA
Human                   TTAAAGACCACTTCTGAGGAATGCAGAGATGCTGATCTTCATGTCATAATGAATGCCCCA
AgileGibbon             TTAAATACCACTTCTGAGGAATGCAGAGATGCTGATCTTCATGTCATAATGAATGCCCCA
Gorilla                 TTAAAGACCACTTCTGAGGAATGCAGAGATGCTGATCTTCATGTCATAATGAATGCCCCA
Howler                  TTAAATACCACTTCTGAAGAACGCAGAAATGCTGATCTTCATCTCATAATGAATGCGCCA
Rhesus                  TTAAATACTACTTCTGAGGAAAACATAGAGGCTGGTCTT---GTCATAATGAATGCCCCA
WolfsGuenon             TTAAATACTACTTCTGAGGAAAACAGAGATGCTGGTCTT---GTCATAATGAATGTCCCA
LeafMonkey              TTAAATACTACTTCTGAGGAAAACAGAGATGCTGGTCTT---GTCATAATTAATGCCCCA
BlackMangabey           TTAAATACTACTTCTGCGGAAAACAGAGATGCTGGTCTT---GTCATAATGAATGCCCCA
PileatedGibbon          TTAAATACCACTTCTGAGGAATGCAGAGATGCTGATCTTCATGTCATAATGAATGCCCCA
SquirrelMonkey          TTAAGTACCACTTCTGAAGAATACAGAGATGCTGATCTTCATCTCATAACGAATGCGCCA
Titi                    TTAAATACCACTTCTGAAGAATACAGAGATGCTAATCTTCATCTCATAATGAATGCACCA
Orangutan               TTAAATACCACTTCTGAGGAATGCAGAGATGCTGATCTTCATGTCATAATGAATGCCCCA
CEMacaque               TTAAATACTACTTCTGAGGAAAACATAGAGGCTGGTCTT---GTCATAATGAATGCCCCA
Talapoin                TTAAATACTACTTCTGAGGAAAAAAGAGATGCTGGTCTT---GTCATAATGAATGCCCCA
WhiteHandedGibbon       TTAAATACCACTTCTGAGGAATGCAGAGATGCTGATCTTCATGTCATAATGAATGCCCCA
Siamang                 TTAAATACCACTTCTGAGGAATGCAGAGATGCTGATCTTCATGTCATAATGAATGCCCCA
WhiteCheekedGibbon      TTAAATACCACTTCTGAGGAATGCAGAGATGCTGATCTTCATGTCATAATGAATGCCCCA
Colobus                 TTAAATACTACT---GAGGAAAACAGAGATGCTGGTCTT---GTCATAATGAATGCCCCA
OliveBaboon             TTAAATACTACTTCTGTGGAAAACAGAGATGCTGGTCTT---GTCATAATGAATGCCCCA
Marmoset                TTAAATAGCACTTCTGAAGAATACAGAGATGCTGATCTTCATCTCGTAATGAACGCGCCA
                        ****  *  * *   *  ***   * * * ***  ****    ** ***  ** *  ***

Bonobo                  TCGACTGGTCAGGTAGACAGCAGCAAGCAATTTGAAGGTACAGTTGAAATTAAACGGAAG
BorneoOrangutan         TCAATTGGTCAGGTAGACAGCAGCAAGCAATTTGAAGGTACAGTTGGAATTAAACAGAAG
Chimpanzee              TCGATTGGTCAGGTAGACAGCAGCAAGCAATTTGAAGGTACAGTTGAAATTAAACGGAAG
RedCheekedGibbon        TCGATTGGTCAGGTAGACAGCAGCAAGCAATTTGAAGGTACAGTTGGAATTAAACAAAAG
Human                   TCGATTGGTCAGGTAGACAGCAGCAAGCAATTTGAAGGTACAGTTGAAATTAAACGGAAG
AgileGibbon             TCGATTGGTCAGGTAGACAGCAGCAAGCAATTTGAAGGTACAGTTGGAATTAAACAAAAG
Gorilla                 TCGATTGGTCAGTTAGACAGCAGCAAGCAATTTGAAGGTACAGTTGAAATTAAACGGAAG
Howler                  TCAATTGGTCAGGTAGACAGAAGCAAGCAATTTGAAGGTACAGTTGAAGTTAAACAAAAG
Rhesus                  TCGATTGGTCAGGTAGACAGCAGCAAGCAATTTGAAGGTACAGTAGGAATTAAACAAAAG
WolfsGuenon             TCGATTGGTCAGGTAAACAGCAGCAAGCAATTTGAAGGTACAGTAGGAATTAAACAAAAG
LeafMonkey              TCGATTGGTCAGGTAGACAGCAGCAAGCAATTTGAAGGTACAGTTGGAATTAAACAAAAG
BlackMangabey           TCGATTGGTCAGGTAGACAGCAGCAAGCAATTTGAAGGTACAGTAGGAATTAAACAAAAG
PileatedGibbon          TCGATTGGTCAGGTAGACAGCAGCAAGCAATTTGAAGGTACAGTTGGAATTAAACAAAAG
SquirrelMonkey          TCAATTGGTCAGGTAGACAGCAGCAAGCAGTTTGAAGGTACCATTGGAATTAAAGAAAAG
Titi                    TCAATTGGTCAGGTAGACAGCAGCAAGCAATTTGAAGGTACAGTTGGAATTAAACAAAAG
Orangutan               TCAATTGGTCAGGTAGACAGCAGCAAGCAATTTGAAGGTACAGTTGGAATTAAACAGAAG
CEMacaque               TCAATTGGTCAGGTAGACAGCAGCAAGCAATTTGAAGGTACAGTAGGAATTAAACAAAAG
Talapoin                TCGATTGGTCAGGTAGACAGCAGCAAGCAATTTGAAGGTACAGTAGGAATTAAACAAAAG
WhiteHandedGibbon       TCGATTGGTCAGGTAGACAGCAGCAAGCAATTTGAAGGTACAGTTGGAATTAAACAAAAG
Siamang                 TCGACTGGTCAGGTAGACAGCAACAAGCAATTTGAAGGTACAGTTGGAATTAAACAAAAG
WhiteCheekedGibbon      TCGATTGGTCAGGTAGACAGCAGCAAGCAATTTGAAGGTACAGTTGGAATTAAACAAAAG
Colobus                 TCGATTGGTCAGGTAGACAGCAGCAAGCAATTTGAAGGTACAGTTGAAATTAAACAAAAG
OliveBaboon             TCGATTGGTCAGGTAGACAGCAGCAAGCAATTTGAAGGTACAGTAGGAATTAAACAAAAG
Marmoset                TCTATTGGTCAGGTAGACAGCAGGAAGCAATTTGAAGGTACAGTTGGAATTAAACAAAAG
                        ** * ******* ** **** *  ***** ***********  * * * *****   ***
```

```
Bonobo                  TTTGCTGGCCTGTTGAAAAATGACTGTAACAAAAGTGCTTCTGGTTATTTAACAGATGAA
BorneoOrangutan         TTTGCTGGCCTGTTGAAAAATGACTGTAACAAAAGTGCTTCTGGTTATTTAACAGATGAA
Chimpanzee              TTTGCTGGCCTGTTGAAAAATGACTGTAACAAAAGTGCTTCTGGTTATTTAACAGATGAA
RedCheekedGibbon        TTTGCTGGCTTGTTGAAAAATGACTGTAACAAAAGTGCTTCTGGTTATTTAACAGATGAA
Human                   TTTGCTGGCCTGTTGAAAAATGACTGTAACAAAAGTGCTTCTGGTTATTTAACAGATGAA
AgileGibbon             TTTGCTGGCTTGTTGAAAAATGACTGTAACAAAAGTGCTTCTGGTTATTTAACAGGTGAA
Gorilla                 TTTGCTGGCCTGTTGAAAAATGACTGTAACAAAAGTGCTTCTGGTTATTTAACAGATGAA
Howler                  TTTGCTGGCTTGTTGAAAAATGACTATAACAAAAATGCATCTGGTTATTTAATAGATGAA
Rhesus                  TTTGCTGGCTTGTTGAAAAATGACTGTAACAAAAGTGCTTCTGGTTATTTAACAGATGAA
WolfsGuenon             TTTGCTGGCTTGTTGAAAAATGACTGTAACAAAAGTGCTTCTGGTTATTTAACAGATGAA
LeafMonkey              TTTGCTGGCTTGTTGAAAAATGACTGTAACAAAAGTGCTTCTGGTTATTTAACAGATGAA
BlackMangabey           TTTGCTGGCTTGTTGAAAAATGACTGTAACAAAAGTGCTTCTGGTTATTTAACAGATGAA
PileatedGibbon          TTTGCTGGCTTGTTGAAAAATGACTGTAACAAAAGTGCTTCTGGTTATTTAACAGGTGAA
SquirrelMonkey          TTTGCTGGCTTGTTGAAAAATGACTGTAACAAAAGTGCATCTGGTTATTTAACAGATGAA
Titi                    TCTGCTGGCTTGTTGAAAAATGACAGCAACAAAAGTGCATCTGGTTATTTAACAGATGAA
Orangutan               TTTGCTGGCCTGTTGAAAAATGACTGTAACAAAAGTGCTGCTGGTTATTTAACAGATGAA
CEMacaque               TTTGCTGGCTTGTTGAAAAATGACTGTAACAAAAGTGCTTCTGGTTATTTAACAGATGAA
Talapoin                TTTGCTGGCTTGTTGAAAAATGACTGTAACAAAAGTGCTTCTGGTTATTTAACAGATGAA
WhiteHandedGibbon       TTTGCTGGCTTGTTGAAAAATGACTGTAACAAAAGTGCTTCTGGTTATTTAACAGGTGAA
Siamang                 TTTGCTGGCTTGTTGAAAAATGACTGTAACAAAAGTGCTTCTGGTTATTTAACAGGTGAA
WhiteCheekedGibbon      TTTGCTGGCTTGTTGAAAAATGACTGTAACAAAAGTGCTTCTGGTTATTTAACAGATGAA
Colobus                 TTTGCTGGCTTGTTGAAAAATGACTGTAACAAAAGTGCTTCTGGTTATTTAACAGATGAA
OliveBaboon             TTTGCTGGCTTGTTGAAAAATGACTGTAACAAAAGTGCTTCTGGTTATTTAACAGATGAA
Marmoset                TTTGCTGTTTTGTTGAAAAATGACTGTAACAAAAGTGCGTCTGGTTATTTAACAGATGCA
                        * *****   **************   ******* ***  ************ ** ** *

Bonobo                  AATGAAGTGGGGTTTAGGGGCTTTTATTCTGCTCATGGCACAAAACTGAAGGTTTCTACT
BorneoOrangutan         AATGAAGTGGGGTTTAGGGGCTTTTATTCTGCTCATGGCGCAAAACTGAATGTTTCTACT
Chimpanzee              AATGAAGTGGGGTTTAGGGGCTTTTATTCTGCTCATGGCACAAAACTGAATGTTTCTACT
RedCheekedGibbon        AATGAAGTGGAGTTTAGGGGCTTTTATTCTGCTCATGGCTCAAAACTGAATGTTTCTACT
Human                   AATGAAGTGGGGTTTAGGGGCTTTTATTCTGCTCATGGCACAAAACTGAATGTTTCTACT
AgileGibbon             AATGAAGTGGAGTTTAGGGGCTTTTATTCTGCTCATGGCTCAAAACTGAATGTTTCTACT
Gorilla                 AATGAAGTGGGGTTTAGGGGCTTTTATTCTGCTCATGGCGCAAAACTGAATGTTTCTACT
Howler                  ------GTGGAGTTTAGAGGCTTTTATTCTGCTCATGGTGTAAAACTGAATGTTTCTACT
Rhesus                  AATGAAGTGGAGTTTAGGGGCTTTTATTCTGCTCATGGCGTAAAACTGAATGTTTCTACT
WolfsGuenon             AATGAAGTGGAGTTTAGGGGCTTTTATTCTGCTCATGGCGTAAAACTGAATGTTTCTACT
LeafMonkey              AATGAAGTGGAGTTTAGGGGCTTTTACTCTGCTCATGGCGTAAAGCTGAATGTTTCTACT
BlackMangabey           AATGAAGTGGCGTTTAGGGGCTTTTATTCTGCTCATGGCGTAAAACTGAATGTTTCTACT
PileatedGibbon          AATGAAGTGGAGTTTAGGGGCTTTTATTCTGCTCATGGCTCAAAACTGAATGTTTCTACT
SquirrelMonkey          AATGAAGTGGAGTTTAGAGGCTTTTATTCTGCTCATGGCATAAAACTGAATGTTTCTACT
Titi                    AATGAAGTGGAGTTTAGAGGCTTTTATTCTGCTCATGGCATAAAACTGAATGTTTCTACT
Orangutan               AATGAAGTGGGGTTTAGGGGCTTTTATTCTGCTCATGGCGCAAAACTGAATGTTTCTACT
CEMacaque               AATGAAGTGGAGTTTAGGGGCTTTTATTCTGCTCATGGCGTAAAACTGAATGTTTCTACT
Talapoin                AATGAAGTGGAGTTTAGGGGCTTTTTTTCTGCTCGTGGCGTAAAACTGAATGTTTCTACT
WhiteHandedGibbon       AATGAAGTGGAGTTTAGGGGCTTTTATTCTGCTCATGGCTCAAAACTGAATGTTTCTACT
Siamang                 AATGAAGTGGAGTTTAGGGGCTTTTATTCTGCTCATGGCTCAAAACTGAATGTTTCTACT
WhiteCheekedGibbon      AATGAAGTGGAGTTTAGGGGCTTTTATTCTGCTCATGGCTCAAAACTGAATGTTTCTACT
Colobus                 AATGAAGTGGAGTTTAGGGGCTTTTACTCTGCTCATGGCATAAAACTGAATGTTTCTACT
OliveBaboon             AATGAAGTGGCGTTTAGGGGCTTTTATTCTGCTCATGGCGTAAAACTGAATGTTTCTACT
Marmoset                GATGAAGTGGAGTTGAGAGGCTTTTATTCTGCTCATGGCGTAAAACTGAATGTTTCTACT
                              **** *** ** *******  ******* ***   *** ***** *********

Bonobo                  GAAGCTCTGCAAAAAGCTGTGAAACTGTTTAGTGATATTGAGAATATTAGTGAGGAAACT
BorneoOrangutan         GAAGCTCTGCAAAAAGCTGTGAAACTGTTTAGTGATATTGAGAATATTGGTGAGGAAACT
Chimpanzee              GAAGCTCTGCAAAAAGCTGTGAAACTGTTTAGTGATATTGAGAATATTAGTGAGGAAACT
RedCheekedGibbon        GAAGCTCTGCAAAAAGCTGTGAAACTGTTTAGTGATATTGAGAATATTAGTGAGGAAACT
Human                   GAAGCTCTGCAAAAAGCTGTGAAACTGTTTAGTGATATTGAGAATATTAGTGAGGAAACT
AgileGibbon             GAAGCTCTGCAAAAAGCTGTGAAACTGTTTAGTGATATTGAGAATATTAGTGAGGAAACT
Gorilla                 GAAGCTCTGCAAAAAGCTGTGAAACTGTTTAGTGATATTGAGAATATTAGTGAGGAAACT
Howler                  GAAGCTCTGCAAAAAGCTGTGAAACTGTTTAGTGATATTGAGAATATTAGTGAGGAAACT
Rhesus                  GAAGCTCTGCAAAAAGCTGTGAAACTGTTCAGTGATATTGAGAATATTAGTGAGGAAACT
WolfsGuenon             GAAGCTCTGCAAAAAGCTGTGAAACTGTTTAGTGATATTGAGAATATTAGTGAGGAAACT
LeafMonkey              GAAGCTCTGCAAAAAGCTGTGAAACTGTTTAGTGATATTGAGAATATTAGTGAGGAAACT
BlackMangabey           GAAGCTCTGCAAAAAGCTGTGAAACTGTTTAGTGATATTGAGAATATTAGTGAGGAAACT
PileatedGibbon          GAAGCTCTGCAAAAAGCTGTGAAACTGTTTAGTGATATTGAGAATATTAGTGAGGAAACT
SquirrelMonkey          GAAGCTCTGCAAAAAGCGGTGAAACTGTTTAGTGATATTGAGAATATTAGTGAGGAAACT
Titi                    GAAGCTCTGCAAAAAGCTGTGAAACTGTTTAGTGATACTGAGAATATTAGTGAGGAAACT
Orangutan               GAAGCTCTGCAAAAAGCTGTGAAACTGTTTAGTGATATTGAGAATATTGGTGAGGAAACT
CEMacaque               GAAGCTCTGCAAAAAGCTGTGAAACTGTTCAGTGATATTGAGAATATTAGTGAGGAAACT
Talapoin                GAAGCTCTGCAAAAAGCTGTGAAACTGTTTAGTGATATTGAGAATATTAGTGAGGAAACT
WhiteHandedGibbon       GAAGCTCTGCAAAAAGCTGTGAAACTGTTTAGTGATATTGAGAATATTAGTGAGGAAACT
Siamang                 GAAGCTCTGCAAAAAGCTGTGAAACTGTTTAGTGATATTGAGAATATTAGTGAGGAAACT
```

```
WhiteCheekedGibbon       GAAGCTCTGCAAAAAGCTGTGAAACTGTTTAGTGATATTGAGAATATTAGTGAGGAAACT
Colobus                  GAAGCTCTGCAAAAAGCTGTGAAACTGTTTAGTGATATTGAGAATATTAGTGAGGAAACT
OliveBaboon              GAAGCTCTGCAAAAAGCTGTGAAACTGTTTAGTGATATTGAGAATATTAGTGAGGAAACT
Marmoset                 GAAGCTCTGCAAAAAGCTGTGAAACTGTTTAGTGATATTGAGAGTATTAGTGAGGAAACT
                         ***************** *********** ******* ***** **** ***********

Bonobo                   TCTGCAGAGGTACATCCAATAAGTTTATCTTCAAGTAAATGTCATGATTCTGTTGTTTCA
BorneoOrangutan          TCTGCAGAAGTAGATCCAATAAGTTTATCTTCAAGTAAATGTCATGATTCTGTTGTTTCA
Chimpanzee               TCTGCAGAGGTACATCCAATAAGTTTATCTTCAAGTAAATGTCATGATTCTGTTGTTTCA
RedCheekedGibbon         TCTGCAGAAGTAGATCCAATAAGTTTATCTTCAAGTAAATGTCATGATTCTGTTGTTTCA
Human                    TCTGCAGAGGTACATCCAATAAGTTTATCTTCAAGTAAATGTCATGATTCTGTTGTTTCA
AgileGibbon              TCTGCAGAAGTAGATCCAATAAGTTTATCTTCAAGTAAATGTCATGATTCTGTTGTTTCA
Gorilla                  TCTGCAGAGGTACATCCAATAAGTTTATCTTCAAGTAAATGTCATGATTCTGTTGTTTCA
Howler                   TCTGCAGAAGTAGATCCAATAAGTTTATCTTCAAGTAAATGTCATCATTCTGTTGTTTCA
Rhesus                   TCTGCAGAAGTAGATCCAATAAGTTTATCTTCAAGTAAATTTCATGATTCTGTTGTTTCA
WolfsGuenon              TCTGCAGAAGTAGATCCAATAAATTTATCTTCAAGTAAATTTCATGATTCTGTTGTTTCA
LeafMonkey               TCTGCAGAAGTAGATCCAATAAGTTTATCTTCAAGTAAATGTCATGATTCTGTTGTTTCA
BlackMangabey            TCTGCAGAAGTAGATCCAATAAGTTTATCTTCAAGTAAATTTCATGATTCTGTTGTTTCA
PileatedGibbon           TCTGCAGAAGTAGATCCAATAAGTTTATCTTCAAGTAAATGTCATGATTCTGTTGTTTCA
SquirrelMonkey           TCTGCAGAAGTAGATCCAATAAGTTTATCTTCAAGTAAATGTCATGATTCTGTTGTTTCA
Titi                     TCTGCAGAAGTAGATCCATTAAGTTTATCTTCAAGTAAATGTCATCATTCTGTT---TCA
Orangutan                TCTGCAGAAGTAGATCCAATAAGTTTATCTTCAAGTAAATGTCATGATTCTGTTGTTTCA
CEMacaque                TCTGCAGAAGTAGATCCAATAAGTTTATCTTCAAGTAAATTTCATGATTCTGTTGTTTCA
Talapoin                 TCTGCAGAAGTAGATACAATAAGTTTATCTTCAAGTAAATGTCATGATTCTGTTGTTTCA
WhiteHandedGibbon        TCTGCAGAAGTAGATCCAATAAGTTTATCTTCAAGTAAATGTCATGATTCTGTTGTTTCA
Siamang                  TCTGCAGAAGTAGATCCAATAAGTTTATCTTCAAGTAAATGTCATGATTCTGTTGTTTCA
WhiteCheekedGibbon       TCTGCAGAAGTAGATCCAATAAGTTTATCTTCAAGTAAATGTCATGATTCTGTTGTTTCA
Colobus                  TCTGCAGAAGTAGATGCAATAAGTTTATCTTCAAGTAAATGTCATGATTCTGTGGTTTCA
OliveBaboon              TCTGCAGAAGTAGATCCAATAAGTTTATCTTCAAGTAAATTTCATGATTCTGTTGTTTCA
Marmoset                 TCTGCAGCAGTAGATCCAATAAGTTTATCTTCAAGTAAATGTCATGATTCTGTTGTTTCA
                         *******  *** ** ** *** ***************** **** *******    ***

Bonobo                   ATGTTTAAGATAGAAAATCATAATGATAAAACTGTAAGTGAAAAAAATAATAAATGCCAA
BorneoOrangutan          ATGTTTAAGATAGAAAATCAT---GATAAAACTGTAAGTGAAAAAAATAATATATGCCAA
Chimpanzee               ATGTTTAAGATAGAAAATCATAATGATAAAACTGTAAGTGAAAAAAATAATAAATGCCAA
RedCheekedGibbon         ATGTTTAAGATAGAAAATCATAGTGATAAAACTGTAAGTGAAAAAAATAATAAAAGCCAA
Human                    ATGTTTAAGATAGAAAATCATAATGATAAAACTGTAAGTGAAAAAAATAATAAATGCCAA
AgileGibbon              GTGTTTAAGATAGAAAATCATAATGATAAAACTGTAAGTGAAAAAAATAATAAAAGCCAA
Gorilla                  ATGTTTAAGATAGAAAATCATAATGATAAAACTGTAAGTGAAAAAAAT---AAATGCCAA
Howler                   ATGTTTAAGATAAAAAATCATAAT---------------GAGAAAAATAATGAATGCCAA
Rhesus                   ATGTTTAAGATAGAAAATCATAATGATAAAACTGTAAGTGGAAAAAAT---AAATGCCAA
WolfsGuenon              ATGTTTAAGATAGAAAATCATAATGATAAAACTGTAAGTGAAAAAAAT---AAATGCCAA
LeafMonkey               ATGTTTAAGATAGAAAATCATAATGATAAAATTGTAAGTGAAAAAAAT---AAATGCCAA
BlackMangabey            ATGTTTAAGATAGAAAATCATAATGATAAAACTGTAAGTGGAAAAAAT---AAATGCCAA
PileatedGibbon           GTGTTTAAGATAGAAAATCATAATGATAAAACTGTAAGTGAAAAAAATAATAAAAGCCAA
SquirrelMonkey           ATGTTTAAGATAGAAAATCATAAT---------------GAGAAAAATAATAAATGTCAA
Titi                     GTGTTTAAGATAGAAAATCATAAT---------------GAGAAAAATAATGAATGCCAA
Orangutan                ATGTTTAAGACAGAAAATCAT---GATAAAACTGTAAGTGAAAAAAATAATATATGCCAA
CEMacaque                ATGTTTAAGATAGAAAATCATAATGATAAAACTGTAAGTGGAAAAAAT---AAATGCCAA
Talapoin                 GTGTTTAAGATAGAAAATCATAATGATAAAAGTGTAAGTGAAAAAAAT---AAATGCCAA
WhiteHandedGibbon        GTGTTTAAGATAGAAAATCATAATGATAAAACTGTAAGTGAAAAAAATAATAAAAGCCAA
Siamang                  ATGTTTAAGATAGAAAATCATAATGATAAAACTGTAAGTGAAAAAAATAATAAAAGCCAA
WhiteCheekedGibbon       ATGTTTAAGATAGAAAATCATAGTGATAAAACTGTAAGTGAAAAAAATAATAAAAGCCAA
Colobus                  GTGTTTAAGATAGAAAATCATAATGATAAAATTGTAAGTGAAAAAAAT---AAATGCCAA
OliveBaboon              ATGTTTAAGATAGAAAATCATAATGATAAAACTGTAAGTGGAAAAAAT---AAATGCCAC
Marmoset                 GTGTTTAAGATAGAAAATCAT---GAT------------GAGAAAAATAATGAATGCCAA
                          ********* * ********                  *  ******     * * **

Bonobo                   CTGATATTACAAAATAATATTGAAATGACTACTGGCACTTCTGTTGAAGAAATTACTGAA
BorneoOrangutan          CTGATACTACAAAATAATATTGAAATGACTAGTGGCACTTTTGTTGAAGAAATTACTGAA
Chimpanzee               CTGATATTACAAAATAATATTGAAATGACTACTGGCACTTCTGTTGAAGAAATTACTGAA
RedCheekedGibbon         CTGATATTACAAAATAATATTGAAATGACTACTGGCACTTTTGTTGAAGAAATTACTGAA
Human                    CTGATATTACAAAATAATATTGAAATGACTACTGGCACTTTTGTTGAAGAAATTACTGAA
AgileGibbon              CTGATATTACAAAATAATATTGAAATGACTACTGGCACTTTTGTTGAAGAAATTACTGAA
Gorilla                  CTGATATTACAAAATAATATTGAAATGACTACTGGCACTTTTGTTGAAGAAATTACTGAA
Howler                   CTAATATTACAAAATAATATTGAAATGACTACTGGCGCTTTTGTTGAAGAAATTACTGAA
Rhesus                   CTGATATTACAAAATAATATTGAAATGACTACTGGGACTTTTGTTGAAGAAATTACTGAA
WolfsGuenon              CTGATGTTACAAAATAATATTGAAATAACTACTGGGACTTTTGTTGAAGAAATTACTGAA
LeafMonkey               CTGATGTTACAAAATAATATTGAAATGACTACTGGGACTTTTGTTGAAGAAATTACTGAA
BlackMangabey            CTGATGTTACAAAATAATATTGAAATGACTACTGGGACTTTTGTTGAAGAAATTACTGAA
PileatedGibbon           CTGATATTACAAAATAATATTGAAATGACTACTGGCACTTTTGTTGAAGAAATTATTGAA
SquirrelMonkey           CTAATATTACAAAATAATATTGAAATGACTACTGGCATTTTTGTTGAAGAAATTACTGAA
Titi                     CTAATATTACAAAAT------GAAATGACTAGTGGCACCTTTGTTGAAGAAATTACTGAA
```

```
Orangutan               CTGATACTACAAAATAATATTGAAATGACTAGTGGCACTTTTGTTGAAGAAATTACTGAA
CEMacaque               CTGATATTACAAAATAATATTGAAATGACTACTGGGACTTTTGTTGAAGAAATTACTGAA
Talapoin                CTGATGTTACAAAATAATATTGAAATGACTACTAGGACTTTTGTTGAAGAAATTACTGAA
WhiteHandedGibbon       CTGATATTACAAAATAATATTGAAATGACTACTGGCACTTTTGTTGAAGAAATTACTGAA
Siamang                 CTGATATTACAAAATAATATTGAAATGACTACTGGCACTTTTGTTGAAGAAATTACTGAA
WhiteCheekedGibbon      CTGATATTACAAAATAATATTGAAATGACTATTGGCACTTTTGTTGAAGAAATTACTGAA
Colobus                 CTGATGTTACAAAATAATATTGAAATGACTACTGGGACTTTTGTTGAAGAAATTACTGAA
OliveBaboon             CTGATGTTACAAAATAATATTGAAATGACTACTGGGACTTTTGTTGAAGAAATTACTGAA
Marmoset                CTAATATTACAAAATAATATTGAAATGACTACTGGCACTTTTGTTGAAGAAATTACTGAA
                        ** **  ********      ***** **** * *    * ************** ****

Bonobo                  AATTACAAGAGAAATACTGAAAATGAAGATAACAAATATACTGCTTCCAGTAGAAATTCT
BorneoOrangutan         AATTACAAGAGAAATACTGAAAATGAAGATAACAAATATACTGCTGCCAGTAGAAATTCT
Chimpanzee              AATTACAAGAGAAATACTGAAAATGAAGATAACAAATATACTGCTTCCAGTAGAAATTCT
RedCheekedGibbon        AATTACAAGAGAAATACTGAAAATGAAGATAACAAATATACTGTTGCCAGTAGAAATTCT
Human                   AATTACAAGAGAAATACTGAAAATGAAGATAACAAATATACTGCTGCCAGTAGAAATTCT
AgileGibbon             AATTACAAGAGAAATACTGAAAATGAAGATAACAAATATACTGTTGCCAGTAGAAATTCT
Gorilla                 AATTACAAGAGAAATACTGAAAATGAAGATAACAAATATACTGCTGCCAGTAGAAATTCT
Howler                  AATTACAAGAAAAATATTGAAAATGATGAT---AAATATACTGCTGCCAGTAGAAATACT
Rhesus                  AATTACAAGATAAATACTGAAAATGAAGATAACAAATATACTGCTGCCAGTAGAAATTCT
WolfsGuenon             AATTACAAGATAAATACTGAAAATGAAGATAACAAATATACTGCTGCCAGTAGAAATTCT
LeafMonkey              AATTACAAGATAAATACTGAAAATGAAGATAACAAATATACTGCTGCCAGTAGAAATTCT
BlackMangabey           AATTACAAGATAAATACTGAAAATGAAGATAACAGATATACTGCTGCCAGTAGAAATTCT
PileatedGibbon          AATTACAAGAGAAATACTGAAAATGAAGATAACAAATATACTGTTGCCAGTAGAAATTCT
SquirrelMonkey          AATTACAAGAAAAATATTGAAAATGAAGAT---AAATATACTGCTGCCAGTAGGAATACT
Titi                    AATTACAAGAAAAATATTGGAAATGAAGAT---AAATATACTGCTGCCAGTAGAAATACT
Orangutan               AATTACAAGAGAAATACTGAAAATGAAGATAACAAATATACTGCTGCCAGTAGAAATTCT
CEMacaque               AATTACAAGATAAATACTGAAAATGAAGATAACAAATATACTGCTGCCAGTAGAAATTCT
Talapoin                AATTACAATATAAATACTGAAAATGAAGAT---AAATATACTGCTGCCAGTAGAAATTCT
WhiteHandedGibbon       AATTACAAGAGAAATACTGAAAATGAAGATAACAAATATACTGTTGCCAGTAGAAATTCT
Siamang                 AATTACAAGACAAATACTGAAAATGAAGATAACAAATATACTGTTGCCAGTAGAAATTCT
WhiteCheekedGibbon      AATTACAAGAGAAATACTGAAAATGAAGATAACAAATATACTGTTGCCAGTAGAAATTCT
Colobus                 AATTACAAGATAAATACTGAAAATGAAGATAACAAATATACTGCTGCCAGTAGAAATTCT
OliveBaboon             AATTACAAGATAAATACTGAAAATGAAGATAACAGATATACTGCTGCCAGTAGAAATTCT
Marmoset                AATTACAAGAAAAATATTGAAAATGAAGAT---AAACATACTGCTACCAGTAGAAATACT
                        ******** * ***** ** ****** ***   * * ****** * ******* *** **

Bonobo                  CATAACTTAGAATTTGATGGCAGTGATTCAAGTAAAAATGATACTGTTTGTATTCATAAA
BorneoOrangutan         CATAACTTAGAATTTGATGGCAGTGATTCAAGTAAAAATGATACTGTTTGTATTCATAAA
Chimpanzee              CATAACTTAGAATTTGATGGCAGTGATTCAAGTAAAAATGATACTGTTTGTATTCATAAA
RedCheekedGibbon        CATAACTTAGAATTTGATGGCAGTGATTCAGGTAAAAATTATACTGTTTGTATTCATAAA
Human                   CATAACTTAGAATTTGATGGCAGTGATTCAAGTAAAAATGATACTGTTTGTATTCATAAA
AgileGibbon             CATAACTTAGAATTTGATGGCAGTGATTCAGGTAAAAATGATACTGTTTGTATTCATAAA
Gorilla                 CATAACTTAGAATTTGATGGCAGTGATTCAAGTAAAAATGATACTGTTTGTATTCATAAA
Howler                  CATAACTTAGAATTCAATGGCAGTGATTCCAGTAAAAATGATACTGTTTATATTCATAAA
Rhesus                  CGTAACTTAGAATTTGTTGGCAGTGATTCAAGTAAAAATGATACTGTTTGTATTCATAAA
WolfsGuenon             CGTAACTTAGAATTTGTTGGCAGTGATTCAAGTAAATATGATACTGTTTGTATTCATAAA
LeafMonkey              CGTAACTTAGAATTTGTTGGCAGTGATTCAAGTAAAAATGATACTATTTGTATTCATAAA
BlackMangabey           CATAACTTAGAATTTGTTGGCAGTGATTCAAGTAAAAATGATACTGTTTGTATTCATAAA
PileatedGibbon          CATAACTTAGAATTTGATGGCAGTGATTCAGGTAAAAATGATACTGTTTGTATTCATAAA
SquirrelMonkey          CATAACTTAGAATTTGATGGCAATGATTCTGGTAAAAATGATAGTATTTATATTCATGAA
Titi                    CATAACTTAGAATTTGATGGCAGTGATTCCAGTAAAAATGATGCTGTTTATATTCATAAA
Orangutan               CATAACTTAGAATTTGATGGCAGTGATTCAAGTAAAAATGATACTGTTTGTATTCATAAA
CEMacaque               CGTAACTTAGAATTTGTTGGCAGTGATTCAAGTAAAAATGATACTGTTTGTATTCATAAA
Talapoin                CGTAACTTAGAATTTGCTGGCAGTGATTCAAGTAAAAATGATACTGTTTGTATTCATAAA
WhiteHandedGibbon       CATAACTTAGAATTTGATGGCAGTGATTCAGGTAAAAATGATACTGTTTGTATTCATAAA
Siamang                 CATAACTTAGAATTTGATGGCAGTGATTCAGGTAAAAATGATACTGTTTGTATTCATAAA
WhiteCheekedGibbon      CATAACTTAGAATTTGATGGCAGTGATTCAGGTAAAAATTATACTGTTTGTATTCATAAA
Colobus                 CGTAACTTAGAATTTGTTGGCAGTGATTCAAGTAAAAATGATACTGTTTGTATTCATAAA
OliveBaboon             CGTAACTTAGAATTTGTTGGCAGTGATTCAAGTAAAAATGATACTGTTTGTATTCATAAA
Marmoset                CATAACTTAGAATTTGATGGCAGTGATTCTAGTAAAAATGATACTGTTCATATTCATAAA
                        * ************    ***** ******  ***** ** **  * **  ******* **

Bonobo                  GATGAAACGGACTTGCTATTTACTGATCAGCACAACATATGTCTTAAATTATCTGGCCAG
BorneoOrangutan         GATGAAACGGACTTGCTATTTACTGATCAGCACAACATATGTCTTAAATTATCTGGCCAG
Chimpanzee              GATGAAACGGACTTGCTATTTACTGATCAGCACAACATATGTCTTAAATTATCTGGCCAG
RedCheekedGibbon        GATGAAACGGACTTGCCATTTACTGATCAGCACAACATATGTCTTAAATTATCTGACCAG
Human                   GATGAAACGGACTTGCTATTTACTGATCAGCACAACATATGTCTTAAATTATCTGGCCAG
AgileGibbon             GATGAAATGGACTTGCCATTTACTGATCAGCACAACATATGTCTTAAATTATCTGACCAG
Gorilla                 GATGAAACGGACTTGCTATTTACTGATCAGCACAACATATGTCTTAAATTATCTGGCCAG
Howler                  GATGAAAATGACTTGCCACTTACTGATCAGCACAACAAATGTCTTAAATTATCTGGCCAG
Rhesus                  GATGAAAAGGACTTGCCATTTACTGATCAGCACAACATATGTCTTAAATTATCTGGCCAG
WolfsGuenon             GATGAAAAGGACTTGCCATTTACTGATCAGCACAGCATATGTCTTAAATTATCTGGCCAG
```

```
LeafMonkey            GATAAAAAGGACTTACCATTTACTGATCAGCACATCATATGTCTTAAATCATCAGGCCAG
BlackMangabey         GATGAAAAGGACTTGCCATTTACTGATCAGCACAACATATGTCTTAAATTATCTGGCCAG
PileatedGibbon        GATGAAATGGACTTGCCATTTACTGATCAGCACAACATATGTCTTAAATTATCTGACCAG
SquirrelMonkey        GATGAAAATGACCTGCCACTTACTAATCAGCGCAACATATATCTTAAATTATCTGGCCAG
Titi                  GATGAAAATGACTTGCCACTTACTAATCAGCATAACATATATCTTAAATTATCTGGCCAG
Orangutan             GATGAAACGGACTTGCTATTTACTGATCAGCACAACATATGTCTTAAATTATCTGGCCAG
CEMacaque             GATGAAAAGGACTTGCCATTTACTGATCAGCACAACATATGTCTTAAATTATCTGGCCAG
Talapoin              GATGAAAAAGACTTGCCATTTACTGATCATCACAACATATGTCTTAAATTATCTGGCCAG
WhiteHandedGibbon     GATGAAATGGACTTGCCATTTACTGATCAGCACAACATATGTCTTAAATTATCTGACCAG
Siamang               GATGAAACGGACTTGCCATTTACTGATCAGCACAACATATGTCTTAAATTATCTGACCAG
WhiteCheekedGibbon    GATGAAACGGACTTGCCATTTACTGATCAGCACAACATATGTCTTAAATTATCTGACCAG
Colobus               GATGAAAAGGACTTACCATTTACCGATCAGCACAACATATGTCTTAAATTATCTGGCCAG
OliveBaboon           GATGAAAAGGACTTGCCATTTACTGATCAGCACAACATATGTCTTAAATTATCTGGCCAG
Marmoset              GATGAAAATGACTTGCCACTTACTAATCAGCACAACATATATCTTAAATTATCTGGCCAG
                      *** ***  *** * * * ****  **** *  * ** ** ******** *** * ****

Bonobo                TTTATGAAGGAGGGAAACACTCAGATTAAAGAAGATTTGTCAGATTTAACTTTTTTGGAA
BorneoOrangutan       TTTATGAAGGAGGGAAACACTCAGATTAAAGAAGATTTGTCAGATTTAACTTTTTTGGAA
Chimpanzee            TTTATGAAGGAGGGAAACACTCAGATTAAAGAAGATTTGTCAGATTTAACTTTTTTGGAA
RedCheekedGibbon      TTTATGAAGGAGGGAAACACTCAGATTAAAGAAGGTTTGTCAGATTTAACTTTTTTGGAA
Human                 TTTATGAAGGAGGGAAACACTCAGATTAAAGAAGATTTGTCAGATTTAACTTTTTTGGAA
AgileGibbon           TTTATGAAGGAGGGAAACACTCAGATTAAAGAAGGTTTGTCAGATTTAACTTTTTTGGAA
Gorilla               TTTATGAAGGAGGGAAACACTCAGATTAAAGAAGATTTGTCAGATTTAACTTTTTTGGAA
Howler                TTTATGAAGGAGAGAAACACTCAGATTAAAGAAGATTTGTCAGATTTAACTTTTTTGGAA
Rhesus                TTTATGAAGGAGGGAAACACTCAGATTAAAGAAGGTTTGTCAGATTTAACTTTTTTGGAA
WolfsGuenon           TTTATGAAGGAGGGAAACACTCACATTAAAGAAGGTTTGTCAGATTTAACTTTTTTGGAA
LeafMonkey            TTTATGAAGGAGGGAAACACTCAGATGAAAGAAGGTTTGTCAGATTTAACTTTTTTAGAA
BlackMangabey         TTTATGAAGGAGGGAAACACTCAGATTAAAGAAGGTTTGTCAGATTTAACTTTTTTGGAA
PileatedGibbon        TTTATGAAGGAGGGAAACACTCAGATTAAAGAAGGTTTGTCAGATTTAACTTTTTTGGAA
SquirrelMonkey        TTTATGAAGAAGGGAAACACTCAGATTAAAGAAGATTTGTCAGATTTAACTTTTTTGGAA
Titi                  TTTATGAAGGAGGGAAACACTCAGATTAAAGAAAGTTTGTCAGATTTAACTTTTTTGGAA
Orangutan             TTTATGAAGGAGGGAAACACTCAGATTAAAGAAGATTTGTCAGATTTAACTTTTTTGGAA
CEMacaque             TTTATGAAGGAGGGAAACACTCAGATTAAAGAAGGTTTGTCAGATTTAACTTTTTTGGAA
Talapoin              TTTATGAAGGAGGGAAACACTCAGATTAAAGAAGGTTTGTCAGATTTAACTTTTTTGGAA
WhiteHandedGibbon     TTTATGAAGGAGGGAAACACTCAGATTAAAGAAGGTTTGTCAGATTTAACTTTTTTGGAA
Siamang               TTTATGAAGGAGGGAAACACTCAGATTAAAGAAGGTTTGTCAGATTTAACTTTTTTGGAA
WhiteCheekedGibbon    TTTATGAAGGAGGGAAACACTCAGATTAAAGAAGGTTTGTCAGATTTAACTTTTTTGGAA
Colobus               TTTATGAAGGAGGGAAACGCTCAGATTAAAGAAGGTTTGTCAGATTTAACTTTTTTGGAA
OliveBaboon           TTTATGAAGGAGGGAAACACTCAGATTAAAGAAGGTTTGTCAGATTTAACTTTTTTGGAA
Marmoset              TTTATGAAGGAGGGAAACACTCAGATTAAAGAAGATTTGTCAGATTTAACTTTTTTGGAA
                      ********* ** ***** **** ** ******  ********************* ***

Bonobo                GTTGTGAAAGCTCAAGAAGCATGTCATGGTAATACTTCAAATAAAGAACAGTTAACTGCT
BorneoOrangutan       GTTGTGAAAGCTCAAGAAACATGTCATGGTAATACTTCAAATAAAGAACAGTTAACTGCT
Chimpanzee            GTTGTGAAAGCTCAAGAAGCATGTCATGGTAATACTTCAAATAAAGAACAGTTAACTGCT
RedCheekedGibbon      GTTGTGAAAGCTCAAGAAACATGTCATGGTAATACTTCAAATAAAGAACAGTTAACTGCT
Human                 GTTGCGAAAGCTCAAGAAGCATGTCATGGTAATACTTCAAATAAAGAACAGTTAACTGCT
AgileGibbon           GTTGTGAAAGCTCAAGAAACATGTCATGGTAATACTTCAAATAAAGAACAGTTAACTGCT
Gorilla               GTTGTGAAAGCTCAAGAAGCATGTCATGGTAATACTTCAAATAAAGAACAGTTAACTGCT
Howler                GTTGTAAAAGCTCAAGAAACATGTCATGGTAAAACTTCAAATAAAGAACAATTAACTGCT
Rhesus                GTCGTGAAAGCTCAAGAAACATGTCATGGTAATACTTCAAATAAAGAACAGTTAACTGCT
WolfsGuenon           GTCGTGAAAGCTCAAGAAACATGTCATGGTAATACTTCAAATAAAGAACAGTTAACTGCT
LeafMonkey            GTTGTGAAAGCTCAAGAAACATGTCATGGTAATACTTCAAATAAAGAACAGTTAACTGCT
BlackMangabey         GTCGTGAAAGCTCAAGAAACATGTCATGGTAATACTTCAAATAAAGAACAGTTAACTGCT
PileatedGibbon        GTTGTGAAAGCTCAAGAAACATGTCATGGTAATACTTCAAATAAAGAACAGTTAACTGCT
SquirrelMonkey        GTCGTAAAAGCTCAAGAAACATGTCGTGGTAATACTTCAAATAAAGAACAGTTAACTGCT
Titi                  GTTGTAAAAGCTCAAGAAACATGTCATGGTAATACTTCAAATAAAGAACAGTTAACTGCT
Orangutan             GTTGTGAAAGCTCAAGAAACATGTCATGGTAATACTTCAAATAAAGAACAGTTAACTGCT
CEMacaque             GTCGTGAAAGCTCAAGAAACATGTCATGGTAATACTTCAAATAAAGAACAGTTAACTGCT
Talapoin              GTCGTGAAAGCTCAAGAAACATGTCATGGTAATACTTCAAATAAAGAACAGTTAACTGCT
WhiteHandedGibbon     GTTGTGAAAGCTCAAGAAACATGTCATGGTAATACTTCAAATAAAGAACAGTTAACTGCT
Siamang               GTTGTGAAAGCTCAAGAAACATGTCATGGTAATACTTCAAATAAAGAACAGTTAACGGCT
WhiteCheekedGibbon    GTTGTGAAAGCTCAAGAAACATGTCATGGTAATACTTCAAATAAAGAACAGTTAACTGCT
Colobus               GTTGTGAAAGCTCAGGAAACATGTCATGGTAATACCTCAAATAAAGAACAGTTAACTGCT
OliveBaboon           GTCGTGAAAGCTCAAGAAACATGTCATGGTAATACTTCAAATAAAGAACAGTTAACTGCT
Marmoset              GTTGTAAAAGCTCAAGAAACATGTCATGGTAATACTTCAAATAAAGAACAGTTAACTGCT
                      ** *  ******** *** ****** ****** ** ************** ***** ***

Bonobo                ACTAAAACGGAGCAAAATATAAAAGATTTTGAGACTTCTGATACATTTTTTCAGACTGCA
BorneoOrangutan       ACTAAAACGGAGCAAAATATAAAAGGTTTTGAGACTTTTGATACATCTTTTCAGACGGCA
Chimpanzee            ACTAAAACGGAGCAAAATATAAAAGATTTTGAGACTTCTGATACATTTTTTCAGACTGCA
RedCheekedGibbon      ACTAAAACAGAGCAAAATATAAAAGATTTTGAGACTTCTGATAAATCTTTTCAGACTGCA
Human                 ACTAAAACGGAGCAAAATATAAAAGATTTTGAGACTTCTGATACATTTTTTCAGACTGCA
```

```
AgileGibbon          ACTAAAACAGAGCAAAATATAAAAGATTTTGAGACTTTTGATAAATCTTTTCAGACTGCA
Gorilla              ACTAAAACGGAGCAAAATATAAAAGATTTTGAGACTTCTGATACATTTTTTCAGACTGCA
Howler               ACTAAAATGGAGCAAAATATAAAAGATTTGGAGACTTTTGATATATCTTTTCAGACAGCA
Rhesus               ACTAAAACAGAGCAAAATATAAAAGATTTTGAGACTTTTGATATATCTTTTCAGACTGCA
WolfsGuenon          ACTAAAACAGAGCAAAATATAAAAGATTTTGAGACTTTTGATTTATCTTTTCAGACTGCA
LeafMonkey           ACTAAAACAGAGCAAAATATAAAAGATTTTGAGACTTTTGATATATCTTTTCAGACTGCA
BlackMangabey        ACTAAAACAGAGCAAAATATAAAAGATTTTGAGACTTTTGATATATCTTTTCAGACTGCA
PileatedGibbon       ACTAAAACAGAGCAAAATATAAAAGATTTTGAGACTTTTGATAAATCTTTTCAGACTGCA
SquirrelMonkey       ACTAAAATGGAGGAAAATATAAAAGATTTTGAGACTTTTGATATATCTTTTCAGACAGCA
Titi                 ACTAAGATGGAGCAAAATATAAAAGATTTTGAGACTTTTGATATATCTTTTCAGACAGCA
Orangutan            ACTAAAACGGAGCAAAATATAAAAGGTTTTGAGACTTTTGATACATCTTTTCAGACGGCA
CEMacaque            ACTAAAACAGAGCAAAATATAAAAGATTTTGAGACTTTTGATATATCTTTTCAGACTGCA
Talapoin             ACTAAAACAGAGCAAAATATAAAAGATTTTGAGACTTTTGGTATATCTTTTCAGACTGCA
WhiteHandedGibbon    ACTAAAACAGAGCAAAATATAAAAGATTTTGAGACTTTTGATAAATCTTTTCAGACTGCA
Siamang              ACTAAAACAGAGCAAAATATAAAAGATTTTGAGACTTTTGATAAATCTTTTCAGACTGCA
WhiteCheekedGibbon   ACTAAAACAGAGCAAAATATAAAAGATTTTGAGACTTCTGATAAATCTTTTCAGACTGCA
Colobus              ACTAAAACAGAGCAAAATATAAAAGATTTTGAGACTTTTGATGTATCTTTTCAGACTGCA
OliveBaboon          ACTAAAACAGAGCAAAATATAAAAGATTTTGAGACTTTTGATATATCTTTTCAGACTGCA
Marmoset             ACTAAAATGGAGCAAAATATAAAAGACGTTGGGACTTTTGATCTGTCTTTTCAGACAGCA
                     ***** *  *** ************   * * ***** ** *   * ********* ***

Bonobo               AGTGGGAAAAATATTAGTGTCGCCAAAGAGTCATTTAATAAAATTGTAAATTTCTTTGAT
BorneoOrangutan      AGTGGGAAAAATATTAGTGTCGCCAAAGAGTCATTTAATAAAATTGTAAATTTCTTTGAT
Chimpanzee           AGTGGGAAAAATATTAGTGTCGCCAAAGAGTCATTTAATAAAATTGTAAATTTCTTTGAT
RedCheekedGibbon     AGTGGGAAAAATATTAGTGTCACCAAACAGTCATTTAATAAAATTGTAAATTTCTTTAAT
Human                AGTGGGAAAAATATTAGTGTCGCCAAAGAGTCATTTAATAAAATTGTAAATTTCTTTGAT
AgileGibbon          AGTGGGAAAAATATTAGTGTCACCAAACAGTCATTTAATAAAATTGTAAATTTCTTTAAT
Gorilla              AGTGGGAAAAATATTAGTGTCGCCAAAGAGTCATTTAATAAAATTGTAAATTTCTTTGAT
Howler               AGTGGGAAAAATATTAGTGTCTCCAAAGAGTCATTTAATAAAGTTGTAAATTTCTTTGAT
Rhesus               AGTGGGAAAAATATTAGTGTCACCAAAGAGTCATTTAATAAAATTGTAAATTTCTTTGAT
WolfsGuenon          AGTGGGAAAAATATTAGTGTCACCAAAGAGTCATTTAATAAAATTGTAAATTTCTTTGAT
LeafMonkey           AGTGGGAAAAATATTAGTGTCACCAAAGAGTCATTTAATAAAATTGTAAATTTCTTTGAT
BlackMangabey        AGTGGGAAAAATATTAGTGTCACCAAAGAGTCATTTAATAAAATTGTAAATTTCTTTGAT
PileatedGibbon       AGTGGGAAAAATATTAGTGTCACCAAACAGTCATTTAATAAAATTGTAAATTTCTTTAAT
SquirrelMonkey       AGTGGGAAAAATATTAGTGTCTCCAAAGAGTCATTTAATAAAGTTGTAAATTTCTTTGAT
Titi                 AGTGGGAAAAATATTATTGTCTCCAAAGAGTCATTTAATAAAGTTGTAAATTTCTTTGAT
Orangutan            AGTGGGAAAAATATTAGTGTCGCCAAAGAGTCATTTAATAAAATTGTAAATTTCTTTGAT
CEMacaque            AGTGGGAAAAATATTAGTGTCACCAAAGAGTCATTTAATAAAATTGTAAATTTCTTTGAT
Talapoin             AGTGGGAAAAATATTAGTGTCACCAAAGAGTCATTTAATAAAATTGTAAATTTCTTTGAT
WhiteHandedGibbon    AGTGGGAAAAATATTAGTGTCACCAAACAGTCATTTAATAAAATTGTAAATTTCTTTAAT
Siamang              AGTGGGAAAAATATTAGTGTCACCAAACAGTCATTTAATAAAATTGTAAATTTCTTTAAT
WhiteCheekedGibbon   AGTGGGAAAAATATTAGTGTCACCAAACAGTCATTTAATAAAATTGTAAATTTCTTTAAT
Colobus              AGTGGGAAAAATATTAGTGTCACCAAAGAGTCATTTAATAAAATTGTAAATTTCTTTGAT
OliveBaboon          AGTGGGAAAAATATTAGTGTCACCAAAGAGTCATTTAATAAAATTGTAAATTTCTTTGAT
Marmoset             AGTGGGAAAAATATTAGTATCTCCAAAGAGTCATTTAATAAAGTTGTAAATTTCTTTGAT
                     **************** * ** ***** ************** ************** **

Bonobo               CAGAAACCAGAAGAATTGCATAACTTTTCCTTAAATTCTGAATTACATTCTGACATAAGA
BorneoOrangutan      CAGAAACCAGAAGAATTGCATAACTTTTCCTTAAATTCTGAATTACATTCTGACATAAGA
Chimpanzee           CAGAAACCAGAAGAATTGCATAACTTTTCCTTAAATTCTGAATTACATTCTGACATAAGA
RedCheekedGibbon     CAGAAACCAGAAGAATTGCATAACTTTTCTTTAAATTCTGAATTACATTCTGACATAAGA
Human                CAGAAACCAGAAGAATTGCATAACTTTTCCTTAAATTCTGAATTACATTCTGACATAAGA
AgileGibbon          CAGAAACCAGAAGAATTGCATAACTTTTCTTTAAATTCTGAATTATATTCTGACATAAGA
Gorilla              CAGAAACCAGAAGAATTGCATAACTTTTCCTTAAATTCTGAATTACATTCTGACATAAGA
Howler               CAGAAACCAGAAGAATTGCATAACTTTTCCTTAAATTCTAAATTACATTCTGACATAAGA
Rhesus               CCGAAACCAGAAGAATTGCATAACTTTTCCTTAAATTCTAAATTACATTCTGACATAAGA
WolfsGuenon          CCAAAACCAGAAGAATTGCATAACTTTTCCTTAAATTCTAAATTACATTCTGACATAAGA
LeafMonkey           CCGAAACCAGAAGAATTGCATAACTTTTCCTTAAATTCTAAATTACATTCTGACATAAGA
BlackMangabey        CCGAAACCAGAAGAATTGCATAACTTTTCCTTAAATTCTAAATTACATTCTGACATAAGA
PileatedGibbon       CAGAAACCAGAAGAATTGCATAACTTTTCTTTAAATTCTGAATTATATTCTGACATAAGA
SquirrelMonkey       CAGAAACCAGAAGAATTGCATAACTTTTCCTTAAATTCTAAATTACATTCGGACGTAAGA
Titi                 CAGAAACCAGAAGAATTGCATAACTTTTCCTTAAATTCTAAATTACATTCTGACATAAGA
Orangutan            CAGAAACCAGAAGAATTGCATAACTTTTCCTTAAATTCTGAATTACATTCTGACATAAGA
CEMacaque            CCGAAACCAGAAGAATTGCATAACTTTTCCTTAAATTCTAAATTACATTCTGACATAAGA
Talapoin             CCGAAACCAGAAGAATTGCATAGCTTTTCCTTAAATTCTGAATTACATTCTGACATAAGA
WhiteHandedGibbon    CAGAAACCAGAAGAATTGCATAACTTTTCTTTAAATTCTGAATTATATTCTGACATAAGA
Siamang              CAGAAACCAGAAGAATTGCATAACTTTTCTTTAAATTCTGAATTACATTCTGACATAAGA
WhiteCheekedGibbon   CAGAAACCAGAAGAATTGCATAACTTTTCTTTAAATTCTGAATTACATTCTGACATAAGA
Colobus              CCGAAACCAGAAGAATTGCATAACTTTTCCTTAAATTCTAAATTACATTCTGACATAAGA
OliveBaboon          CCGAAACCAGAAGAATTGCATAACTTTTCCTTAAATTCTAAATTACATTCTGACATAAGA
Marmoset             CAGAAACCAGAAGAACTGCATAACTTTTCCTTAAATTCTAAATTACATTCTGACATAAGA
                     *  ************ ****** ****** ********* ***** **** *** *****
```

```
Bonobo                  AAGAACAAAATGGACATTCTAAGTTATGAGGAAACAGACATAGTTAAACACAAAATACTG
BorneoOrangutan         AAGAACAAAATGGACATTCTAAGTCATGAGGAAACAGACATAGTTAAAAACAAAATACTG
Chimpanzee              AAGAACAAAATGGACATTCTAAGTTATGAGGAAACAGACATAGTTAAACACAAAATACTG
RedCheekedGibbon        AAGAACAAAATGGACATTCTAAGTCATGAGGAAACAGACATAGTTAAAAACAAAATACTG
Human                   AAGAACAAAATGGACATTCTAAGTTATGAGGAAACAGACATAGTTAAACACAAAATACTG
AgileGibbon             AAGAACAAAATGGACATTCTAAGTCATGAGGAAACAGACATAGTTAAAAACAAAATACTG
Gorilla                 AAGAACAAAATGGACATTCTAAGTTATGAGGAAACAGACATAGTTAAACACAAAATACTG
Howler                  AAGAACAAAATGGACATTCTAAGTCATGAGGAAACAGACATTGTTAAAAACAAAACACTG
Rhesus                  AAGAACAAAATGGACATTCTAAGTCATGAGGAAACAGACACAGTTAAAAACAAAATACTG
WolfsGuenon             AAGAACAAAATGGACATTCTAAGTCATGAGGAAACAGACACAGTTAAAAACAAAATACTG
LeafMonkey              AAGAACAAAATGGACATTCTAAGTCATGAGGAAACAGACACAGTTAAAAACAAAATACTG
BlackMangabey           AAGAACAAAATGGACATTCTAAGTCATGAGGAAACAGACACAGTTAAAAACAAAATACTG
PileatedGibbon          AAGAACAAAATGGACATTCTAAGTCATGAGGAAACAGACATAGTTAAAAACAAAATACTG
SquirrelMonkey          AAGAACAAAATGGACATTCTAAGTCATGAGGAAACAAACATAGTTAAAAACAAAACACTG
Titi                    AAGAATAAAATGGACATTCTAAGTCATGAGGAAACAGACATAGTTAAAAACAAAACACTG
Orangutan               AAGAACAAAATGGACATTCTAAGTCATGAGGAAACAGACATAGTTAAAAACAAAATACTG
CEMacaque               AAGAACAAAATGGACATTCTAAGTCATGAGGAAACAGACACAGTTAAAAACAAAATACTG
Talapoin                AAGAACAAAATGGACATTCTAAGTCATGAGGAAACAGACACAGTTAAAAACAAAATACTG
WhiteHandedGibbon       AAGAACAAAATGGACATTCTAAGTCATGAGGAAACAGACATAGTTAAAAACAAAATACTG
Siamang                 AAGAACAAAATGGACATTCTAAGTCATGAGGAAACAGACATAGTTAAAAACAAAATACTG
WhiteCheekedGibbon      AAGAACAAAATGGACATTCTAAGTCATGAGGAAACAGACATAGTTAAAAACAAAATACTG
Colobus                 AAGAACAAAATGGACATTCTAAGTCATGAGGAAACAGACACAGTTAAAAACAAAATACTG
OliveBaboon             AAGAACAAAATGGACATTCTAAGTCATGAGGAAACAGACACAGTTAAAAACAAAATACTG
Marmoset                AAGAACAAAATGGACATTCTAAGTTATGAGGAAACAGACATAGTTAAAAACAAAACACTG
                        ***** ****************** *********** ***  ****** ****** ****

Bonobo                  AAAGAAAGCGTCCCAGTTGGTACTGGAAATCAACTAGTGACCTTCCAGGGACAACCCGAA
BorneoOrangutan         AAAGAAAGTGTCCCAGTTGGTACTGGAAATCAACTAGTGACCTTCCAGGAACAACCTGAA
Chimpanzee              AAAGAAAGCGTCCCAGTTGGTACTGGAAATCAACTAGTGACCTTCCAGGGACAACCCGAA
RedCheekedGibbon        AAAGAAAGTGTCCCAGGTGGTACTGGAAATCAACTAGTGACCTTCCAGGAACAACCCGAA
Human                   AAAGAAAGTGTCCCAGTTGGTACTGGAAATCAACTAGTGACCTTCCAGGGACAACCCGAA
AgileGibbon             AAAGAAAGTGTCCCAGGTGGTACTGGAAATCAACTAGTGACCTTCCAGGAACAACCCGAA
Gorilla                 AAAGAAAGTGTCCCAGTTGGTACTGGAAATCAACTAGTGACCTTCCAGGGACAACCCGAA
Howler                  AACGAAAGTATCCCAGTTGGCACTGGAAATCAACTAGTGACCATCCAGGAACAACCCAAA
Rhesus                  AAAGAAAGTGTCCCAGTTGGTACTGGAAATCAACTAGTGACCTTCCAGGAACGACCCCAA
WolfsGuenon             AAAGAAAGTGTCCCAGTTGGTACTGGAAATCAACTAGTGACCTTCCAGGAACGACCCCAA
LeafMonkey              AAAGAAAGTGTCCCAGTTGGTACTGGAAATCAACTAGTGACCTTCCAGGAACGACCCCAA
BlackMangabey           AAAGAAAGTGTCCCAGTTGGTACTGGAAATCAACTAGTGACCTTCCAGGAACGACCCCAA
PileatedGibbon          AAAGAAAGTGTCCCAGGTGGTACTGGAAATCAACTAGTGACCTTCCAGGAACAACCCGAA
SquirrelMonkey          AAAGAAAGTATCCCAGTTGGCACTGGAGATCAACTAGTGACCATCCAGGAACAACCCAAA
Titi                    AAAGAAACTATCCCAGTTGGCACTGGAAATCAACTATTGACCATCCAGGAACAACCCAAA
Orangutan               AAAGAAAGTGTCCCAGTTGGTACTGGAAATCAACTAGTGACCTTCCAGGAACAACCTGAA
CEMacaque               AAAGAAAGTGTCCCAGTTGGTACTGGAAATCAACTAGTGACCTTCCAGGAACGACCCCAA
Talapoin                AAAGAAAGTGTCCCAGTTGGTACTGGAAATCAACTAGTGACCTTCCAGGAACAACCCCAA
WhiteHandedGibbon       AAAGAAAGTGTCCCAGGTGGTACTGGAAATCAACTAGTGACCTTCCAGGAACAACCCGAA
Siamang                 AAAGAAAGTGTCCCAGGTGGTACTAGAAATCAACTAGTGACCTTCCAGGAACAACCCGAA
WhiteCheekedGibbon      AAAGAAAGTGTCCCAGGTGGTACTGGAAATCAACTAGTGACCTTCCAGGAACAACCCGAA
Colobus                 AAAGAAAGTGTCCCAGTTGGTACTGGAAATCAACTAGTGACCTTCCAGGAACGACCCCAA
OliveBaboon             AAAGAAAGTGTCCCAGTTGGTACTGGAAATCAACTAGTGACCTTCCAGGAACGACCCCAA
Marmoset                AAAGAAAGTATCCCAGTTGGCACTGGAAATCAACTAGTGACCATCCAGGAACAACCCAAA
                        ** ****   ****** *** *** ** ******** ***** ****** ** ***  **

Bonobo                  CGTGAT---GAAAAGATCAAAGAACCTACTCTGTTGGGTTTTCATACAGCTAGTGGGAAA
BorneoOrangutan         CGTGAT---GAAAAGATCAAAGAACCTACTCTGTTGGGTTTTCATACAGCTAGTGGGAAA
Chimpanzee              CGTGAT---GAAAAGATCAAAGAACCTACTCTGTTGGGTTTTCATACAGCTAGTGGGAAA
RedCheekedGibbon        CGTGAT---GAAAAGATCAAAGAACCTACTCTGTTGGGTTTTCATACAGCTAGTGGAAAA
Human                   CGTGAT---GAAAAGATCAAAGAACCTACTCTATTGGGTTTTCATACAGCTAGCGGGAAA
AgileGibbon             CGTGAT---GAAAAGATCAAAGAACCTACTCTGTTGGGTTTTCATACAGCTAGTGGAAAA
Gorilla                 CGTGAT---GAAAAGATCAAAGAACCTACTCTGTTGGGTTTTCATACAGCTAGCGGGAAA
Howler                  AGTGATGTCGAAAAGATCAAAGAACCTACCCTGTTGGGTTTTCACACAGCCAGTGGGAAA
Rhesus                  GGTGAT---GAAAAGATCAAAGAACCTACTCTGTTGGGTTTTCATACAGCTAGTGGGAAA
WolfsGuenon             GGTGAT---GAAGAGATCAAAGAACCTACTCTGTTGGGTTTTCATACAGCTAGTGGGAAA
LeafMonkey              GGTGAT---GAAAAGATCAAAGAACCTAATCTGTTGGGTTTTCATACAGCTAGTGGGAAA
BlackMangabey           GGTGAT---GAAAAGATCAAAGAACCTACTCTGTTGGGTTTTCATACAGCTAGTGGGAAA
PileatedGibbon          CGTGAT---GAAAAGATCAAAGAACCTACTCTGTTGGGTTTTCATACAGCTAGTGGAAAA
SquirrelMonkey          AGTGATATTGAAAAGATCAAAGAACCTACCCTGTTGGGTTTTCACACAGCTAGTGGGAAA
Titi                    AGTGATATCGAAAAGATCAAAGAACGTACCCTGTTGGGTTTTCACACGGCTAGTGGGAAA
Orangutan               CGTGAT---GAAAAGATCAAAGAACCTACTCTGTTGGGTTTTCATACAGCTAGTGGGAAA
CEMacaque               GGTGAT---GAAAAGATCAAAGAACCTACTCTGTTGGGTTTTCATACAGCTAGTGGGAAA
Talapoin                GGTGAT---GAAAAGATCAAAGAACCTACTCTGTTGGGTTTTCATACAGCTAGTGGGAAA
WhiteHandedGibbon       CGTGAT---GAAAAGATCAAAGAACCTACTCTGTTGGGTTTTCATACAGCTAGTGGAAAA
Siamang                 CGTGAT---GAAAAGATCAAAGAACCTACTCTGTTGGGTTTTCATACAGCTAGTGGAAAA
WhiteCheekedGibbon      CGTGAT---GAAAAGATCAAAGAACCTACTCTGTTGGGTTTTCATACAGCTAGTGGAAAA
```

```
Colobus                 GGTGAT---GAAAAGATCAAAGAACCTAATCTGATGGGTTTTCATACAGCTAGTGGGAAA
OliveBaboon             GGTGAT---GAAAAGATCAAAGAACCTACTCTGTTGGGTTTTCATACAGCTAGTGGGAAA
Marmoset                AGTAATATCAAAAAGATCAAAGAACCTACCCTGTTGGGTTTTCACACAGCTAGTGGGAAA
                         ** **    ** ************ **  **  ********** ** ** ** ** ***

Bonobo                  AAAGTTAAAATTGCAAAGGAATCTTTGGACAAAGTGAAAAATCTTTTTGATGAAAAAGAG
BorneoOrangutan         AAAGTTAAAATTGCAAAGGAATCTTTGGACAAAGTGAAAAATCTTTTTGATGAAAAAGAG
Chimpanzee              AAAGTTAAAATTGCAAAGGAATCTTTGGACAAAGTGAAAAATCTTTTTGATGAAAAAGAG
RedCheekedGibbon        AAAGTTAAAATTGCAAAGGAATCTTTGGATAAAGTGAAAAATCTTTTTGATGAAAAAGAG
Human                   AAAGTTAAAATTGCAAAGGAATCTTTGGACAAAGTGAAAAACCTTTTTGATGAAAAAGAG
AgileGibbon             AAAGTTAAAATTGCAAAGGAATCTTTGGATAAAGTGAAAAATCTTTTTGATGAAAAAGAG
Gorilla                 AAAGTTAAAATTGCAAAGGAATCTTTGGACAAAGTGAAAAATCTTTTTGATGAAAAAGAG
Howler                  AAAGTTAAAATTGCAAAGGAATCTTTGGACAAAGTGAAAAATCTTTTTGATGAAAGAGAG
Rhesus                  AAAGTTAAAATTACAAAGGAATCTTTGGACAAAGTGAAAAATCTTTTTGATGAAAAAGAG
WolfsGuenon             AAAGTTAAAATTACAAAGGAATCTTTGGACAAAGTGAAAAATCTTTTTGATGAAAAAGAG
LeafMonkey              AAAGTTAAAATTACAAAGGAATCTTTGGACAGAGTGAAAAATCTTTTTGATGAAAAAGAG
BlackMangabey           AAAGTTAAAATTACAAAGGAATCTTTGGACAAAGTGAAAAATCTTTTTGATGAAAAAGAG
PileatedGibbon          AAAGTTAAAATTGCAAAGGAATCTTTGGATAAAGTGAAAAATCTTTTTGATGAAAAAGAG
SquirrelMonkey          AAAGTTAAAATTGCGAAGGAATCTTTGGACAAAGTGAAAAACCTTTTTGATGAAAAAGAG
Titi                    AAAGTAAAAATTGCAAAGGAATCTTTGGACAAAGTGAAAAATCTTTTTGATGAAAAAGAG
Orangutan               AAAGTTAAAATTGCAAAGGAATCTTTGGACAAAGTGAAAAATCTTTTTGATGAAAAAGAG
CEMacaque               AAAGTTAAAATTACAAAGGAATCTTTGGACAAAGTGAAAAATCTTTTTGATGAAAAAGAG
Talapoin                AAAGTTAAAATTACAAAGGAATCTTTGGACAAAGTGAAAAATCTTTTTGATGAAAAAGAG
WhiteHandedGibbon       AAAGTTAAAATTGCAAAGGAATCTTTGGATAAAGTGAAAAATCTTTTTGATGAAAAAGAG
Siamang                 AAAGTTAAAATTGCAAAGGAATCTTTGGATAAAGTGAAAAATCTTTTTGATGAAAAAGAG
WhiteCheekedGibbon      AAAGTTAAAATTGCAAAGGAATCTTTGGATAAAGTGAAAAATCTTTTTGATGAAAAAGAG
Colobus                 AAAGTTAAAATTACAAAGGAATCTTTGGACAGAGTGAAAAATCTTTTTGATGAAAAAGAG
OliveBaboon             AAAGTTAAAATTACAAAGGAATCTTTGGACAAAGTGAAAAATCTTTTTGATGAAAAAGAG
Marmoset                AAAGTTAAAATTGCAAAGGAATCTTTGGACAAAGTGAAAAACCTTTTTGATGAAAAAGGG
                        ***** ****** * ************** * ********* ************ ** *

Bonobo                  CAAGGTACTAGTGAAATCACCAGTTTTAGCCATCAATGGGCAAAGACCCTAAAGTACAGA
BorneoOrangutan         CAAGGTACTTGTGAAATCACCAGTTTTAGCCATCAATGGGCAAAGACCCTAAAGTACAGA
Chimpanzee              CAAGGTACTAGTGAAATCACCAGTTTTAGCCATCAATGGGCAAAGACCCTAAAGTACAGA
RedCheekedGibbon        CAAGGTACTAGTGAAATCACCAGTTTTAGCCATCAACGGGCAAAGACCCTAAAGTACAGA
Human                   CAAGGTACTAGTGAAATCACCAGTTTTAGCCATCAATGGGCAAAGACCCTAAAGTACAGA
AgileGibbon             CAAGGTACTAGTGAAATCACCAGTTTTAGCCATCAACGGGCAAAGACCCTAAAGTACAGA
Gorilla                 CAAGGTACTAGTGAAATCACCAGTTTTAGCCATCAATGGGCAAAGACCCTAAAGTACAGA
Howler                  CAAGATACTGGTGAAATCACCAGTTTTAGCCATCAAGGGGCAAAGACCCTAAAGTACAGA
Rhesus                  CAAGGTACTAGTGAAATCACCAGTTTTAGCCATCAATGGGCAAAGACCCTAAAGTACAGA
WolfsGuenon             CAAGGTACTAGTGAAATCACCAGTTTTAGCCATCAATGGGCAAAGACCCTAAAGTACAGA
LeafMonkey              CAAGGTACTAGTGAAATCACCAGTTTTAGCCATCAATGGGCAAAGACCCTAAAGTACAAA
BlackMangabey           CAAGGTACTAGTGAAATCACCAGTTTTAGCCATCAATGGGCAAAGACCCTAAAGTACAGA
PileatedGibbon          CAAGGTACTAGTGAAATCACCAGTTTTAGCCATCAACGGGCAAAGACCCTAAAGTACAGA
SquirrelMonkey          CAAGATACTGGTGAAATCACCAATTTTAGCCATCAAGGAGCAAAGACCCTGAAGTACAGA
Titi                    CAAGATACTGGTGAAATCATCAGTTTTAGCCATCAAGGGGCAAATACCCTAAAGTACAGA
Orangutan               CAAGGTACTTGTGAAATCACCAGTTTTAGCCATCAATGGGCAAAGACCCTAAAGTACAGA
CEMacaque               CAAGGTACTAGTGAAATCACCAGTTTTAGCCATCAATGGGCAAAGACCCTAAAGTACAGA
Talapoin                CAAGGTACTAGTGAAATCACCAGTTTTAGCCATCAATGGGCAAAGACCCTAAAGTACAGA
WhiteHandedGibbon       CAAGGTACTAGTGAAATCACCAGTTTTAGCCATCAACGGGCAAAGACCCTAAAGTACAGA
Siamang                 CAAGGTACTAGTGAAATCACCAGTTTTAGCCATCAACGGGCAAAGACCCTAAAGTACAGA
WhiteCheekedGibbon      CAAGGTACTAGTGAAATCACCAGTTTTAGCCATCAACGGGCAAAGACCCTAAAGTACAGA
Colobus                 CAAGGTACTAGTGAAATCACCAGTTTTAGCCATCAATGGGCAAAGACCCTAAAGTACAAA
OliveBaboon             CAAGGTACTAGTGAAATCACCAGTTTTAGCCATCAATGGGCAAAGACCCTAAAGTACAGA
Marmoset                CAAGATACTGGTGAAATCACCAGTTTTAGCCATCAAGGGGCAAAGACCCTAAAGTACAGA
                        **** **** ********* ** ************* * ***** ***** ******* *

Bonobo                  GAGGCCTGTAAAGACCTTGAATTAGCATGTGAGACCATTGAGGTCACAACTGCCCCAAAG
BorneoOrangutan         GATGCCCGTAAAGACCTTGAATTAGCATGTGAGACAATTGAGATCACAACTGCCCCAAAG
Chimpanzee              GAGGCCTGTAAAGACCTTGAATTAGCATGTGAGACCATTGAGATCACAACTGCCCCAAAG
RedCheekedGibbon        GAGGCCTGTAAAGATCTTGAATTAGCATGTGAGACAATTGAGATCACAACTGCCCCAAAG
Human                   GAGGCCTGTAAAGACCTTGAATTAGCATGTGAGACCATTGAGATCACAGCTGCCCCAAAG
AgileGibbon             GAGGCCTGTAAAGACCTTGAATTAGCATGTGAGACAATTGAGATCACAACTGCCCCAAAG
Gorilla                 GAGGCCTGTAAAGACCTTGAATTAGCATGTGAGACCATTGAGATCACAACTGCCCCAAAG
Howler                  GAGGCCTATAAAGACCTTGAATTAGTGTGTAAGACAGTTGAGATAACAACTACTCGAAAG
Rhesus                  GAGGCTTGTAAAGACCTTGAATTAGCGTGTGAAACAGTTGAGATCACAACTGCCCCAAAG
WolfsGuenon             GAGGCTTGTCAAGACCTTGAATTAGCGTGTGAAACAGTTGAGATCACAACTGCCCCAAAG
LeafMonkey              GAGGCTTGTAAAGACCTTGAATTAGCATGTGAAACAGTTGAGATCACAACTGCCCCAAAG
BlackMangabey           GAGGCTTGTAAAGACCTTGAATTAGCGTGTGAAACAGTTGAGATCACAACTGCCCCAAAG
PileatedGibbon          GAGGCCTGTAAAGACCTTGAATTAGCATGTGAGACAATTGAGATCACAACTGCCCCAAAG
SquirrelMonkey          GAGGCCTATAAAGACCATGAATTAGTGTGTGAGACAATTGAGATAGCAACTACTACAAAG
Titi                    GAGGCCTATAAAGACCTTGAATTAGCGTGTGAGGCAATTGAGATAGCAACTACTCCAAAA
Orangutan               GATGCCCGTAAAGACCTTGAATTAGCACGTGAGACAATTGAGATCACAACTGCCCCAAAG
```

```
CEMacaque               GAGGCTTGTAAAGACCTTGAATTAGCGTGTGAAACAGTTGAGATCACAACTGCCCCAAAG
Talapoin                GAGGCTTGTAAAGACCTTGAATTAGCGTGTGAAACAGTTGAGATCACGACTGCCCCAAAG
WhiteHandedGibbon       GAGGCCTGTAAAGACCTTGAATTAGCATGTGAGACAATTGAGATCACAACTGCCCCAAAG
Siamang                 GAGGCCTGTAAAGACCTTGAATTAGCATGTGAGACGATTGAGATCACAACTGCCCCAAAG
WhiteCheekedGibbon      GAGGCCTGTAAAGATCTTGAATTAGCATGTGAGACAATTGAGATCACAACTGCCCCAAAG
Colobus                 GAGGCTTGTAAAGACCTTGAATTAGCATGTGAAACAGTTGAGATCACAACTGCCCCAAAG
OliveBaboon             GAGGCTTGTAAAGACCTTGAATTAGCGTGTGAAACAGTTGAGATCACAACTGCCCCAAAG
Marmoset                GAGGCCTATAAAGACCTTGAATTAGTGCGTGAGACAGTTGAGATAGCAACTACTCGAAAG
                        ** **   * **** * ********   ** *  *  ***** *  *  ** *   ***

Bonobo                  TGTAAAGAAATGCAGAATTCTCTCAATAATGATAAAAACCTTGTTTCTATTGAGACTGTG
BorneoOrangutan         TGTAAAGAAATGCAGAATTCTCGCAATAATGATAAAAACCTTGTTTCTATTGAGACTGTG
Chimpanzee              TGTAAAGAAATGCAGAATTCTCTCAATAATGATAAAAACCTTGTTTCTATTGAGACTGTG
RedCheekedGibbon        TGTAAAGAAATGCAGAATTCTCTCAAT---GATAAAAACCTTGTTTCTATTGAGACTGTG
Human                   TGTAAAGAAATGCAGAATTCTCTCAATAATGATAAAAACCTTGTTTCTATTGAGACTGTG
AgileGibbon             TGTAAAGAAATGCAGAATTCTCTCAAT---GATAAAAACCTTGTTTCTATTGAGACTGTG
Gorilla                 TGTAAAGAAATGCAGAATTCTCTCAATAATGATAAAAACCTTGTTTCTATTGAGACTGTG
Howler                  TGTGAAGAAATGCAGAATTCTCTCAGTAATGATAAAAATCTTGTTTCTGTTGAGACTGTG
Rhesus                  TGTAAAGAAATGCAGAATTCTCTCAATAATGATAAAAACCTTGTTTCTATTGAGACTGTG
WolfsGuenon             TGTAAAGAAATGCAGAATTCTCTCAATAATGATAAAAACCTTGTTTGTATTGAGACTGTG
LeafMonkey              TGTAAAGAAATGCAGAATTATCTCAATAATGTTAAAAACCTTGTTTCTGTTGAGACTGTG
BlackMangabey           TGTAAAGAAATGCAGAATTCTCTCAATAATGATAAAAACCTTGTTTCTATTGAGACTGTG
PileatedGibbon          TGTAAAGAAATGCAGAATTCTCTCAAT---GATAAAAACCTTGTTTCTATTGAGACTGTG
SquirrelMonkey          CCTGAAGAAATGCAGAATTCTCTCAGTAATGATAAAAATCTTGTTTCTATTGAGACTGTG
Titi                    TGTGAAGAAATGCAGAATTCTCTCAGTAATGAT------------TCTATTAAGACTGTG
Orangutan               TGTAAAGAAATGCAGAATTCTCGCAATAATGATAAAAACCTTGTTTCTATTGAGACTGTG
CEMacaque               TGTAAAGAAATGCAGAATTCTCTCAATAATGATAAAAACCTTGTTTCTATTGAGACTGTG
Talapoin                TGTAAAGAAATGCAGAATTCTCTCAATAATGATAAAAACCTTGTTTCTATTGAGACTGTG
WhiteHandedGibbon       TGTAAAGAAATGCAGAATTCTCTCAAT---GATAAAAACCTTGTTTCTATTGAGACTGTG
Siamang                 TGTAAAGAAATGCAGAATTCTCTCAAT---GATAAAAACCTTGTTTCTATTGAGACTGTG
WhiteCheekedGibbon      TGTAAAGAAATGCAGAATTCTCTCAAT---GATAAAAACCTTGTTTCTATTGAGACTGTG
Colobus                 TGTAAAGAAATGCAGAATTATCTCAATAATGATAAAAACCTTGTTTCTATTGAGACTGTG
OliveBaboon             TGTAAAGAAATGCAGAATTCTCTCAATAATGATAAAAACCTTGTTTCTATTGAGACTGTG
Marmoset                CATGAAGAAATGCAGAATTCTCTCAGTAATGATAAAAATCTTGTTTCTATTGAGACTGTG
                          * *************** ** ** *   * *            * * ** ********

Bonobo                  GTGCCACCTAAGCTCTTAAGTGATAATTTATGTAGACAAACTGAAAATCTCAAAACATCA
BorneoOrangutan         GTGCCACCTAAGCTCTTAAGTGATAATTTATGTAGACAAACTGAAAATCTCAAAACATCA
Chimpanzee              GTGCCACCTAAGCTCTTAAGTGATAATTTATGTAGACAAACTGAAAATCTCAAAACATCA
RedCheekedGibbon        GTGCCACCTAAGCTCTTAAGTGATAATTTATGTAGCCAAACTGAAAATCTCAAAACATCA
Human                   GTGCCACCTAAGCTCTTAAGTGATAATTTATGTAGACAAACTGAAAATCTCAAAACATCA
AgileGibbon             GTGCCACCTAAGCTCTTAAGTGATAATTTATGTAGCCAAACTGAAAATCTCAAAACATCA
Gorilla                 GTGCCACCTAAGCTCTTAAGTGACAATTTATGTAGACAAACTGAAAATCTCAAAACATCA
Howler                  GTGCCACCTAAGCTCTTAAGTGATAATTTATATAGACATACTGAAAGTCTCAAAACATCT
Rhesus                  GTGCCACCTAAGCTGTTAAGTGATCATTTGTGTAGACAAACTGAAAATCTCAAAACGTCA
WolfsGuenon             GTGCCACCTAAGTTGTTAAGTGATAATTTGTGTAGACAAACTGAAAATCTCAAAACGTCA
LeafMonkey              GTGCCACCTAAGCTGTTAAGTGATAATTTGTGTAGACAAACTGAAAATCTCAAAACATCA
BlackMangabey           GTGCCACCTAAGCTGTTAAGTGATCATTTGTGTAGACAAACTGAAAATCTCAAAACGTCA
PileatedGibbon          GTGCCACCTAAGCTCTTAAGTGATAATTTATGTAGCCAAACTGAAAATCTCAAAACATCA
SquirrelMonkey          GTGCCACCTAAGCTTTTAAGTGATAATTTATATCAACATACTGAAAGTCTCAAAACATCT
Titi                    GTGCCACCTAAGCTCTTAAGTGATAATTTATGTAGGCATACTGAAAGTCTCAAAACATCT
Orangutan               GTGCCACCTAAGCTCTTAAGTGATAATTTATGTAGACAAACTGAAAATCTCAAAACATCA
CEMacaque               GTGCCACCTAAGCTGTTAAGTGATCATTTGTGTAGACAAACTGAAAATCTCAAAACGTCA
Talapoin                GTGCCACCTAAGTTGTTAAGTGATAATTTGTGTAGACAAACTGAAAATCTCAAAACATCA
WhiteHandedGibbon       GTGCCACCTAAGCTCTTAAGTGATAATTTATGTAGCCAAACTGAAAATCTCAAAACATCA
Siamang                 GTGCCACCTAAGCTCTTAAGTGATAATTTATGTAGCCAAACTGAAAATCTCAAAACATCA
WhiteCheekedGibbon      GTGCCACCTAAGCTCTTAAGTGATAATTTATGTAGCCAAACTGAAAATCTCAAAACATCA
Colobus                 GTGCCACCTAAGCTGTTAAGTGATAATTTGTGTAGACAAACTGAAAATCTCAAAACATCA
OliveBaboon             GTGCCACCTAAGCTGTTAAGTGATCATTTGTGTAGACAAACTGAAAATCTCAAAACGTCA
Marmoset                GTGCCACCTAAGCCCTTAAGTGATAATTTATATAGACATACTGAAAGTCTCAAAACATCT
                        ************   ********  **** * *   ** ******* ********* **

Bonobo                  AAAAGTATCTTTTTGAAAGTTAAAGTACATGAAAATGTAGAAAAAGAAACAGCAAAAAGT
BorneoOrangutan         AAAAGTATCTTTTTGAAAGTTAAAGTACATGAAAATGTAGAAAAAGAAACAGCAAAAAGT
Chimpanzee              AAAAGTATCTTTTTGAAAGTTAAAGTACATGAAAATGTAGAAAAAGAAACAGCAAAAAGT
RedCheekedGibbon        AAAAGTATCTTTTTGAAAGTTAAAGTACATGAAAATGTAGAAAAAGAAACAGCAAAAAGT
Human                   AAAAGTATCTTTTTGAAAGTTAAAGTACATGAAAATGTAGAAAAAGAAACAGCAAAAAGT
AgileGibbon             AAAAGTATCTTTTTGAAAGTTAAAGTACATGAAAATGTAGAAAAAGAAACAGCAAAAAGT
Gorilla                 AAAAGTATCTTTTTGAAAGTTAAAGTACATGAAAATGTAGAAAAAGAAACAGCAAAAAGT
Howler                  GAAAGTATCTTTTTGAAAGTTAAAGAACATGAAAATGTAGAAAAAGAAACAGCAAAAAGT
Rhesus                  AAAAGTATCTTTTTGAAAGTTAAAGTACATGAAAATGTAGAAAAAGAAACAGCAAAAAGT
WolfsGuenon             AAAAGTATCTTTTTGAAAGTTAAAGTACATGAAAATGTAGAAAAAGAAACAGCAAAAAGT
LeafMonkey              AAAAGTATCTTTTTGAAAGTTAAAGTACATGAAAATATCAAAAAAGAAACAGCAAAAAGT
```

```
BlackMangabey           AAAAGTATCTTTTTGAAAGTTAAAGTACATGAAAATGTAGAAAAAGAAACAGCAAAAAGT
PileatedGibbon          AAAAGTATCTTTTTGAAAGTTAAAGTACATGAAAATGTAGAAAAAGAAACAGCAAAAAGT
SquirrelMonkey          GAAAGTATCTTTTTGAAAGTTAAAGAACATGAAAATGTAGAAAAAGAAACAGCAAAAAGT
Titi                    GAAAGTGTGTTTTTGAAAGTTAAAGAACATGAAAATGTAGAAAAAGAAACAGCAAAAAGT
Orangutan               AAAAGTATCTTTTTGAAAGTTAAAGTACATGAAAATGTAGAAAAAGAAACAGCAAAAAGT
CEMacaque               AAAAGTATCTTTTTGAAAGTTAAAGTACATGAAAATGTAGAAAAAGAAACAGCAAAAAGT
Talapoin                AAAAGTATCTTTTTGAAAGTTAAAGTACATGAAAATGTAGAAGAAGAAACAGCAAAAAGT
WhiteHandedGibbon       AAAAGTATCTTTTTGAAAGTTAAAGTACATGAAAATGTAGAAAAAGAAACAGCAAAAAGT
Siamang                 AAAAGTATCTTTTTGAAAGTTAAAGTACATGAAAATGTAGAAAAAGAAACAGCAAAAAGT
WhiteCheekedGibbon      AAAAGTATCTTTTTGAAAGTTAAAGTACATGAAAATGTAGAAAAAGAAACAGCAAAAAGT
Colobus                 AAAAGTATCTTTTTGAAAGTTAAAGTACATGAAAATGTCGAAAAAGAAACAGCAAAAAGT
OliveBaboon             AAAAGTATCTTTTTGAAAGTTAAAGTACATGAAAATGTAGAAAAAGAAACAGCAAAAAGT
Marmoset                GAAGGTAACTTTTTGAAAGTTAAAGAACATGAAAATGTAGAAAAAGAAACAGCAGAAAGT
                         ** **   **************** ********** *  ** *********** *****

Bonobo                  CCTGCAACTTGTTACACAAATCAGTCCCCTTATTCAGTCATTGAAAATTCAGCCTTAGCT
BorneoOrangutan         CCTGCAACTTGTTACACAAATCAGTCCCCTTATTCAGTCATTGAAAATTCAGCCTTAGCT
Chimpanzee              CCTGCAACTTGTTACACAAATCAGTCCCCTTATTCAGTCATTGAAAATTCAGCCTTAGCT
RedCheekedGibbon        CCTGCAGCTTGTTACACAAATCAGTCCCCTTATTCAGTCATTGAAAATTCAGCCTTAGCT
Human                   CCTGCAACTTGTTACACAAATCAGTCCCCTTATTCAGTCATTGAAAATTCAGCCTTAGCT
AgileGibbon             CCTGCAGCTTGTTACACAAATCAGTCCCCTTATTCAGTCATTGAAAATTCAGCCTTAGCT
Gorilla                 CCTGCAACTTGTTACACAAATCAGTCCCCTTATTCAGTCATTGAAAATTCAGCCTTAGCT
Howler                  CCTGCAACTTGTTACACAAATCAGTCCCCTTATTCAGTCACTGAAAGTTCAGCCTTAGCT
Rhesus                  CCTGCAACTTGTTACACAAATCAGTCCCCTTATTCAGTCATTGAAAATTCAGCCTTGGCT
WolfsGuenon             CCTACAACTTGTTACACAAATCAGTCCCCTTATTCAGTCATTGAAAATTCAGCCTTGGCT
LeafMonkey              CCTGCAACTTGCTACACAAATCAGTCCCCTTATTCAGTCATTGAAAATTCAGCCTTGGCT
BlackMangabey           CCTGCAACTTGTTACACAAATCAGTCCCCTTATTCAGTCATTGAAAATTCAGCCTTGGCT
PileatedGibbon          CCTGCAGCTTGTTACACAAATCAGTCCCCTTATTCAGTCACTGAAAATTCAGCCTTAGCT
SquirrelMonkey          CCTGCAACGTGTTACGCCAATCAGTCCCCTTATTCAGTCATTGAAAATTCAGCCTTAGCT
Titi                    CCTGCAACTTGTTACACAAATCAATCCCCTTATTCAGTCACTGAAAATTCAGCTTTAGCT
Orangutan               CCTGCAACTTGTTACACAAATCAGTCCCCTTATTCAGTCATTGAAAATTCAGCCTTAGCT
CEMacaque               CCTGCAACTTGTTACACAAATCAGTCCCCTTATTCAGTCATTGAAAATTCAGCCTTGGCT
Talapoin                CCTGCAACTTGTTACACAAATCAGTCCCCTTATTCAGTCATTGAAAATTCAGCCTTGGCT
WhiteHandedGibbon       CCTGCAGCTTGTTACACAAATCAGTCCCCTTATTCAGTCATTGAAAATTCAGCCTTAGCT
Siamang                 CCTGCAGCTTGTTACACAAATCAGTCCCCTTATTCAGTCATTGAAAATTCAGCCTTAGCT
WhiteCheekedGibbon      CCTGCAGCTTGTTACACAAATCAGTCCCCTTATTCAGTCATTGAAAATTCAGCCTTAGCT
Colobus                 CCTGCAACTTGCTACACAAATCAGTCCCCTTATTCAGTCATTGAAAATTCAGCCTTGGCT
OliveBaboon             CCTGCAACTTGTTACACAAATCAGTCCCCTTATTCAGTCATTGAAAATTCAGCCTTGGCT
Marmoset                CCTGCAACTTGTTACAGAAATCAGTCCCCTTACTCGGTCATTGAAAATTCAGCCTTAGCT
                        *** ** * ** ***   ***** ******** ** **** ***** ****** ** ***

Bonobo                  TTTTATACAAGTTGTAGTAGAAAAACTTCTGTGAGTCAGACTTCATTACTTGAAGCAAAA
BorneoOrangutan         TTTTACACAAGTTGTAGTAGAAAAACTTCTGTGAGTCAGACTTCATTAACTGAAGCAAAA
Chimpanzee              TTTTATACAAGTTGTAGTAGAAAAACTTCTGTGAGTCAGACTTCATTACTTGAAGCAAAA
RedCheekedGibbon        TTTTACACAAGTTGTAGTAGAAAAACTTCTGTGAGTCAGACTTCATTACTTGAAGCAAAA
Human                   TTTTACACAAGTTGTAGTAGAAAAACTTCTGTGAGTCAGACTTCATTACTTGAAGCAAAA
AgileGibbon             TTTTACACAAGTTGTAGTAGAAAAACTTCTGTGAGTCAGACTTCATTACTTGAAGCAAAA
Gorilla                 TTTTACACAAGTTGTAGTAGAAAAACTTCTGTGAGTCAGACTTCATTACGTGAAGCAAAA
Howler                  TTTTGCACAAGTTGTAGTAGAAAAACTTCTGTGAGTCAGGCTTCATCACTTGAAGCAAAA
Rhesus                  TTTTACACAAGTTGTAGTAGAAAACGTTCTGTGAGTCAAACTTCATTACTTGAAGCAAAA
WolfsGuenon             TTTTACACAAGTTGTAGTAGAAAACGTTCTGTGAGTCAAACTTCATTACTTGAAGCAAAA
LeafMonkey              TTTTACACGAGTTGTAGTAGAAAACCTTCTGTGAGTCAAACTTCATTACTTGAAGCAAAA
BlackMangabey           TTTTACACAAGTTGTAGTACAAAACGTTCTGTGAGTCAAACTTCATTACTTGAAGCAAAA
PileatedGibbon          TTTTACACAAGTTGTAGTAGAAAAACTTCTGTGAGTCAGACTTCATTACTTGAAGCAAAA
SquirrelMonkey          TTTTACACAAATTGTAGTAGAAAAACTTCTGTGAGTCAGGCTTCATCACTTGAAGCAAAA
Titi                    TTTTACACAAATTGTAGTACAAAAACTTCTGTGAGTCAGGCTTCATCACTTGAAGCAAAA
Orangutan               TTTTACACAAGTTGTAGTAGAAAAACTTCTGTGAGTCAGACTTCATTAACTGAAGCAAAA
CEMacaque               TTTTACACAAGTTGTAGTAGAAAACGTTCTGTGAGTCAAACTTCATTACTTGAAGCAAAA
Talapoin                TTTTACACAAGTTGTAGTAGAAAACGTTCTGTGAGTCAAACTTCATTACTTGAAGCAAAA
WhiteHandedGibbon       TTTTACACAAGTTGTAGTAGAAAAACTTCTGTGAGTCAGACTTCATTACTTGAAGCAAAA
Siamang                 TTTTACACAAGTTGTAGTAGAAAAACTTCTGTGAGTCAGACTTCATTACTTGAAGCAAAA
WhiteCheekedGibbon      TTTTACACAAGTTGTAGTAGAAAAACTTCTGTGAGTCAGACTTCATTACTTGAAGCAAAA
Colobus                 TTTTACACGAGTTGTAGTAGAAAACCTTCTGTGAGTCAAACTTCATTACTTGAAGCAAAA
OliveBaboon             TTTTACACAAGTTGTAGTACAAAACGTTCTGTGAGTCAAACTTCATTACTTGAAGCAAAA
Marmoset                TTTTACACATATTGTAGTAGAAAAACTTCTGTGAGTCAGGCTTCATCACTTGAAGCAAAA
                        ****  **   ******** ****  ************  ****** *  **********

Bonobo                  AAATGGCTTAGAGAAGGAATATTTGATGGTCAACCAGAAAGAATAAATACTGCAGATTAT
BorneoOrangutan         AAATGGCTTAGAGAAGGAATATTTGATGGTCAACCAGAAAGAATAAATACTGCAGATTAT
Chimpanzee              AAATGGCTTAGAGAAGGAATATTTGATGGTCAACCAGAAAGAATAAATACTGCAGATTAT
RedCheekedGibbon        AAATGGCTTAGAGAAGGAATATTTGATAGTCAACCAGAAAGAATAAATACTGCAGATTAT
Human                   AAATGGCTTAGAGAAGGAATATTTGATGGTCAACCAGAAAGAATAAATACTGCAGATTAT
AgileGibbon             AAATGGCTTAGAGAAGGAATATTTAATAGTCAACCAGAAAGAATAAATACTGCAGATTAT
```

```
Gorilla                 AAATGGCTTAGAGAAGGAATATTTGATGGTCAACCAGAAAGAATAAATACTGCAGATTAT
Howler                  AAATGTCTTAGA---GGAGAATTTGATGATCAACCAGAAAGAATAAACACTGTAGATTAT
Rhesus                  CAATGGCTTAGAGAAGGAATATTTGATGATCAACCAGAAAGAATAAATACTGCAGATTAT
WolfsGuenon             CAATGGCTTAGAGAAGGAATATTTGATGATCAACCAGAAAGAATAAATACTGCAGATTAT
LeafMonkey              CAGTGGCTTAAAGAAGGAATATTTGATGATCAACCAGAAAGAATAAATACTGCAGATTAT
BlackMangabey           CAATGGCTTAGAGAAGGAATATTTGATGATCAACCAGAAAGAATAAATACTGCGGATTAT
PileatedGibbon          AAATGGCTTAGAGAAGGAATATTTGATAGTCAACCAGAAAGAATAAATACTGCAGATTAT
SquirrelMonkey          AAATGGCTTAGAGAAGGAGAATTTGATGATCAACCAGAAAAAATAAATACTGTAGATTAT
Titi                    AAATGGCTTAGAGAAGGAGAATTTGATGATCAACCAGAAAGAATAAATACTTTAGATTAT
Orangutan               AAATGGCTTAGAGAAGGAATATTTGATGGTCAACCAGAAAGAGTAAATACTGCAGATTAT
CEMacaque               CAATGGCTTAGAGAAGGAATATTTGATGATCAACCAGAAAGAATAAATACTGCAGATTAT
Talapoin                CAATGGCTTAGAGAAGGAATATTTGATGATCAACCAGAAAGAATAAATACTGCAGATTAT
WhiteHandedGibbon       AAATGGCTTAGAGAAGGAATATTTGATAGTCAACCAGAAAGAATAAATACTGCAGATTAT
Siamang                 AAATGGCTTAGGGAAGGAATATTTGATAGTCAACCAGAAAGAATAAATACTGCAGATTAT
WhiteCheekedGibbon      AAATGGCTTAGAGAAGGAATATTTGATAGTCAACCAGAAAGAATAAATACTGCAGATTAT
Colobus                 CAATGGCTTAAAGAAGGAATATTTGATGATCAACCAGAAAGAATAAATACTGCAGATTAT
OliveBaboon             CAATGGCTTAGAGAAGGAATATTTGATGATCAACCAGAAAGAATAAATACTGCGGATTAT
Marmoset                CAATGGCTTAGAGAAGGAGAATTTGATGATCAACCAGAAAATATAAATACTGTAGATTTT
                         * ** ****     ***  **** **  ***********   **** ***   **** *

Bonobo                  GTAGGAAATTATTTGTATGAAAATAATTCAAACAGTACTATAGCTGAAAATGACAAAAAT
BorneoOrangutan         GTAGGAAATTCTTTGTATGAAAATAATTCAAAT---ACTATAGCTGAAAATGACAAAAAT
Chimpanzee              GTAGGAAATTATTTGTATGAAAATAATTCAAACAGTACTATAGCTGAAAATGACAAAAAT
RedCheekedGibbon        GTAGGAAATTCATTGTATGAAAATAATTCAAACAATACTATAGCTGAAAATGACAAAAAT
Human                   GTAGGAAATTATTTGTATGAAAATAATTCAAACAGTACTATAGCTGAAAATGACAAAAAT
AgileGibbon             GTAGGAAATTCATTGTATGAAAATAATTCAAACAATACTGTAGCTGAAAATGACAAAAAT
Gorilla                 GTAGGAAATTATTTGTATGAAAATAACTCAAACAGTACTATAGCTGAAAATGACAAAAAT
Howler                  GTAGGAAATTCTTTGTATGAAAATAATTCAAACAATACTATAACTGAAAGTAACAAAAAT
Rhesus                  GTAGGAAATTCTTTGTATGAAAATAATTCAAAGAATACTATAGCTGAAAGTGACAAAAAT
WolfsGuenon             GTAGGAAATTCTTTGTATGAAAATAATTCAAAGAATACTATAGCTGAAAGTGACAAAAAT
LeafMonkey              GTAGGAAATTCTTTGTATGAAAATAATTCAAACAATACTATAGCTGAAAGTGACAAAAAT
BlackMangabey           GTAGGAAATTCTTTGTATGAAAATAATTCAAAGAATACTATAGCTGAAAGTGACAAAAAT
PileatedGibbon          GTAGGAAATTCATTGTATGAAAATAATTCAAACAATACTGTAGCTGAAAATGACAAAAAT
SquirrelMonkey          GTAGGAAATTCTTTGTATGAAAATAATTCAAACAGTACTATAACCGAAGGTAACAAAAAT
Titi                    GTAGGAAATTCTTCATGTGAAAATAATTCAAACAATACTATAACTGAAAGTAACAAAAAT
Orangutan               GTAGGAAATTCTTTGTATGAAAGTAATTCAAAT---ACTATAGCTGAAAATGACAAAAAT
CEMacaque               GTAGGAAATTCTTTGTATGAAAATAATTCAAAGAATACTATAGCTGAAAGTGACAAAAAT
Talapoin                GTAGGAAATTCTTTGTATGAAAATAATTCAAACAATACTATAGCTGAAAGTGACAAAAAT
WhiteHandedGibbon       GTAGGAAATTCATTGTATGAAAATAATTCAAACAATACTGTAGCTGAAAATGACAAAAAT
Siamang                 GTAGGAAATTCATTGTATGAAAATAATTCAAACAATACTGTAGCTGAAAATGACAAAAAT
WhiteCheekedGibbon      GTAGGAAATTCATTGTATGAAAATAATTCAAACAATACTATAGCTGAAAATGACAAAAAT
Colobus                 GTAGGAAATTCTTTGTATGAAAATAATTCAAACAACACTATAGCTGAAAGTGACAAAAAT
OliveBaboon             GTAGGAAATTCTTTGTATGAAAATAATTCAAAGAATACTATAGCTGAAAGTGACAAAAAT
Marmoset                GTAGGAAATTCTTTGTATGAAAATAATTCAAACAATACTATAACTGAAGGTAACAAAAAT
                        **********  *  * ***** *** *****    *** ** * ***  * ********

Bonobo                  CATCTCTCCGCAAAACAAGATACTTATTTAAGTAACAGTAGCATGTCTAACAGCTATTCC
BorneoOrangutan         CATCTCTCTGAAAAACAAGATACTTATTTAAGTAACAGTAGCATGTCTAACAGCTATTCC
Chimpanzee              AATCTCTCTGCAAAACAAGATACTTATTTAAGTAACAGTAGCATGTCTAACAGCTATTCC
RedCheekedGibbon        CATCTCTCCGAAAAACAAGATACTTATTTAAGTAATAGTAGCATGTCTAACAGCTATTCC
Human                   CATCTCTCCGAAAAACAAGATACTTATTTAAGTAACAGTAGCATGTCTAACAGCTATTCC
AgileGibbon             CATCTCTCCGAAAAACAAGATACTTATTTAAGTAACAGTAGCATGTCTAACAGCTATTCC
Gorilla                 CATCTCTCCGAAAAACAAGATACTTATTTAAGTAACAGTAGCATGTCTAACAGCTATTCC
Howler                  CATCTCTCCGAAAAACAAGCAACTTATTTAAGTAACAGTAGCATGTCTAACAGCTATTCC
Rhesus                  CATCCCTCCGAAAAACAAGATACTACTTTAAATAACAGTAGCATGTCTAACAGCTGTTCC
WolfsGuenon             CATCCCTCCAAAAAACAAGATACTACTTTAAATAACAGTAGCATGTCTAACAGCTATTCC
LeafMonkey              CATCCCTCCGAAAAACAAGATACTTCTTTAAATAACAGTAGCATGCCTAACAGCTATTCC
BlackMangabey           CATCCCTCCGAAAAACAAGATACTACTTTAAATAACAGTAGCATGTCTAACAGCTATTCC
PileatedGibbon          CATCTCTCCGAAAAACAAGATACTTATTTAAGTAACAGTAGCATGTCTAACAGCTATTCC
SquirrelMonkey          CATCTCTCTGAAAAACAAGATACTTATTTAAGTAACAGCAGG---TCTAACAGCTATTCC
Titi                    CATATCTCCGAAAAACAAGATACTTATTTAAGTAAGAGTAGCATGTCTAACAGCTATTCC
Orangutan               CATCTCTCTGAAAAACAAGATACTTATTTAAGTAACAGTAGCATGTCTAACAGCTATTCC
CEMacaque               CATCCCTCCGAAAAACAAGATACTACTTTAAATAACAGTAGCATGTCTAACAGCTATTCC
Talapoin                CATCCCTCCGAAAAACAAGATACTACTTTAAATAACAGTAGCATGTCTAACAGCTATTTC
WhiteHandedGibbon       CATCTCTCCGAAAAACAAGATACTTATTTAAGTAACAGTAGCATGTCTAACAGCTATTCC
Siamang                 CATCTCTCTGAAAAACAAGATACTTATTTAAGTAACAGTAGCATGTCTAACAGCTATTCC
WhiteCheekedGibbon      CATCTCTCCGAAAAACAAGATACTTATTTAAGTAATAGTAGCATGTCTAACAGCTATTCC
Colobus                 CATCCCTCCGAAAAACAAGATACTTCTTTAAATAACAGGAGCATGCCTAATAGCTATTCC
OliveBaboon             CATCCCTCCGAAAAACAAGATACTACTTTAAATAACAGTAGCATGTCTAACAGCTATTCC
Marmoset                CATCTCTCCGAAAAACAAGATACTTATTTAAGTAACAGTAGCATGTCTAACAGCTATTCC
                         **  ***   ********  ***  ***** *** ** **     **** **** ** *

Bonobo                  TACCATTCTGATGAGGTATATAATGATTCAGGATATCTCTCAAAAAATAAACTTGATTCT
```

```
BorneoOrangutan         TACCATTCTGATGAGGTACATAATGATTCAGGATATCTCTCAAAAAATAAACTTGATTCT
Chimpanzee              TACCATTCTGATGAGGTATATAATGATTCAGGATATCTCTCAAAAAATAAACTTGATTCT
RedCheekedGibbon        TACCATTCTGATGAGGTATATAATGATTCGGGGTATATCTCAAAAAATAAACTTGATTCT
Human                   TACCATTCTGATGAGGTATATAATGATTCAGGATATCTCTCAAAAAATAAACTTGATTCT
AgileGibbon             TACCATTCTGATGAGGTATATAATGATTCGGGATATCTCTCAAAAAATAAACTTGATTCT
Gorilla                 TACCATTCTGATGAGGTATATAATGATTCAGGATATCTCTCAAAAAATAAACTTGATTCT
Howler                  TACCATTCTGATGAGGTATATAATGATTCAGGATATCTCTCAAAAAATAAACTTGATTCT
Rhesus                  TACCTTTCTGATGAAGTATATAGTGATTCGGGATATCTCTCAAAAAATAAACTTGATTCT
WolfsGuenon             TACCTTTCTGATGAAGTATATAGTGATTCGGGATATCTCTCAAAAAATAAACTTGATTCT
LeafMonkey              TACCTTTCCGATGAGGTATATAATGATTCGGGATATCTCTCAAAAAATAAACTTGATTCT
BlackMangabey           TACCTTTCTGATGAAGTATATAGTGATTCGGGATATCTCTCAAAAAATAAACTTGATTCT
PileatedGibbon          TACCATTCTGATGAGGTATATAATGATTCGGGATATCTCTCAAAAAATAAACTTGATTCT
SquirrelMonkey          TACCATTCTGATGAGGTATATAATGATTCAGGATATCTCTCAAAAGTAAACTTGATTCT
Titi                    TACCATTCTGATGAGGTATATAATGATTCAGGATATCTCTCAAAAAATAAACTTGATTCT
Orangutan               TACCATTCTGATGAGGTACATAATGATTCAGGATATCTCTCAAAAAATAAACTTGATTCT
CEMacaque               TACCTTTCTGATGAAGTATATAGTGATTCGGGATATCTCTCAAAAAATAAACTTGATTCT
Talapoin                TACCTTTCTGATGAAGTATATAGTGATTCGGGATATCTCTCAAAAAATAAACTTGATTCT
WhiteHandedGibbon       TACCATTCTGATGAGGTATATAATGATTCGGGATATCTCTCAAAAAATAAACTTGATTCT
Siamang                 TACCACTCTGATGAGGTATATAATGATTCGGGATATCTCTCAAAAAATAAACTTGATTCT
WhiteCheekedGibbon      TACCATTCTGATGAGGTATATAATGATTCGGGGTATATCTCAAAAAATAAACTTGATTCT
Colobus                 TACCTTTCTGATGAGGTATATAATGATTCGGGGTATCTCTCAAAAAATAAACTTGATTCT
OliveBaboon             TACCTTTCTGATGAAGTATATAGTGATTCGGGATATCTCTCGAAAAATAAACTTGATTCT
Marmoset                TACCATTCTGATGAGGTATATAATGATTCAGGATATCGCTCAAAAAATAATCTTGATTCT
                        ****  ** ***** *** *** ****** ** ***  *** **** *** *********

Bonobo                  GGTATTGAGCCAGTATTGAAGAATGTTGAAGATCAAAAAAACACTAGTTTTTCCAAAGTA
BorneoOrangutan         GGTATTGAGCCAGTATTGAAGGATGTTGAAGATCAAAAAAACACTAGTTTTTCCAAAGTA
Chimpanzee              GGTATTGAGCCAGTATTGAAGAATGTTGAAGATCAAAAAAACACTAGTTTTTCCAAAGTA
RedCheekedGibbon        GGTATTGAGCCAGTATTGAAGAATGTTGAAGACCAAAAAAACACTAGTTTTTCCGAAGTA
Human                   GGTATTGAGCCAGTATTGAAGAATGTTGAAGATCAAAAAAACACTAGTTTTTCCAAAGTA
AgileGibbon             GGTATTGAGCCAGTATTGAAGAATGTTGAAGACCAAAAAAACACTAGTTTTTCCAAAGTA
Gorilla                 GGTATTGAGCCAGTATTGAAGAATGTTGAAGATCAAAAAAACACTAGTTTTTCCAAAGTA
Howler                  GGTATTGAGCCAGTAGTGAAGAATGTTGAAGATCAAAAAAACACTAGTTTTTCCAAAGTA
Rhesus                  GGTATTGAGCCAGTATTGAAGAAAGTTGAAGACCAAAAAAACACTAGTTTTTCCAAAGTA
WolfsGuenon             GGTATTGAGCCAGTATTGAAGAAAGTTGAAGACCAAAAAAACACTAGTTTTTCCAAAGTA
LeafMonkey              GGTATTGAGCCAGTATTGAAGAATGCTGAAGACCAAAAAAACACTAGTTTTTCCAAAGTA
BlackMangabey           GGTATTGAGCCAGTATTGAAGAAAGTTGAAGACCAAAAAAACACTAGTTTTTCCAAAGTA
PileatedGibbon          GGTATTGAGCCAGTATTGAAGAATGTTGAAGACCAAAAAAACACTAGTTTTTCCAAAGTA
SquirrelMonkey          GGTATTGAGCCAGTAATGAAGAATGTTGAAGACCAAAAAAACACTAGTTTTTCCAAAGTA
Titi                    GGTATTGAGCCAGTAGTGAAGAATGTTGAAGACCAAAAAAACATTAGTTTTTCCAAAGTA
Orangutan               GGTATTGAGCCAGTATTGAAGGATGTTGAAGATCAAAAAAACACTAGTTTTTCCAAAGTA
CEMacaque               GGTATTGAGCCAGTATTGAAGAAAGTTGAAGACCAAAAAAACACTAGTTTTTCCAAAGTA
Talapoin                GGTATTGAGCCAGTATTGAAGAATGTTGAAGACCAAAAGAACACTAGTTTTTCCAAAGTA
WhiteHandedGibbon       GGTATTGAGCCAGTATTGAAGAATGTTGAAGACCAAAAAAACACTAGTTTTTCCAAAGTA
Siamang                 GGTATTGAGCCAGTATTGAAGAATGTTGAAGACCAAAAAAACACTAGTTTTTCCAAAGTA
WhiteCheekedGibbon      GGTATTGAGCCAGTATTGAAGAATGTTGAAGACCAAAAAAACACTAGTTTTTCCGAAGTA
Colobus                 GGTATTGAGCCAGTATTGAAGAATGTTGAAGACCAAAAAAACACTAGTTTTTCCAAACTA
OliveBaboon             GGTATTGAGCCAGTATTGAAGAAAGTTGAAGACCAAAAAAACACTAGTTTTTCCAAAGTA
Marmoset                GGTATTGAGCCAGTAGTGAAGAATGTTGAAGACCAGAAAAACACTCGTTCTTCCAAAGTA
                        *************** ***** * * ****** ** ** **** * *** **** ** **

Bonobo                  ATATCCAATGTAAAAGATGCAAATGCATACCCGCAAACTATAAATGAAGATATTTGTGTT
BorneoOrangutan         ATATCCAATGTAAAAGATGCAAATGCATACCCGCAAACTGTAAATGAAGATATTTGTGTT
Chimpanzee              ATATCCAATGTAAAAGATGCAAATGCATACCCGCAAACTATAAATGAAGATATTTGTGTT
RedCheekedGibbon        ATATCCAATGTAAAAGATGCAAATGCATACCCGCAAACTGTAAATGAAGATATTTGTGTT
Human                   ATATCCAATGTAAAAGATGCAAATGCATACCCACAAACTGTAAATGAAGATATTTGCGTT
AgileGibbon             ATATCCAATGTAAAAGATGCAAATGCATACCCGCAAACTCTAAATGAAGATATTTGTGTT
Gorilla                 ATATCCAATGTAAAAGATGCAAATGCATACCCGCAAACTGTAAATGAAGATATTTGTGTT
Howler                  ACATCCAATGTGAAAGATGCAAATGCATACCCGCAAACTGTAAATGAAGATAGTTGTGTT
Rhesus                  ATATCCAGTGTAAAAGATGCAAATACATACCCTCAAACTGTAAATGAAGGTATTTGTGTT
WolfsGuenon             ATATCCAGTGTAAAAGATGCAAATACATACCCTCAAACTGTAAATGAAGGTATTTGTGTT
LeafMonkey              ATATCCAGTGTAAAAGATGCAAATACATACCCTCAAACTGTAAATGAAGGTATTTGTGTT
BlackMangabey           ATATCCAGTGTAAAAGATGCAAATACATACCCTCAAACTGTAAATGAAGGTATTTGTGTT
PileatedGibbon          ATATCCAATGTAAAAGATGCAAATGCATACCCGCAAACTCTAAATGAAGATATTTGTGTT
SquirrelMonkey          ACATCCAGTGTGAAAGATGCAAATGCATACCCGCAAACTGTAAATGAAGATATTTGTGTT
Titi                    ACATCCAATGTGAAAGATGCAAATGCATATCCGCACACTGTAAATGAAGATATTTGTGTT
Orangutan               ATATCCAATGTAAAAGATGCAAATGCATACCCGCAAACTGTAAATGAAGATATTTGTGTT
CEMacaque               ATATCCAGTGTAAAAGATGCAAATACATACCCTCAAACTGTAAATGAAGGTATTTGTGTT
Talapoin                ATATCCAGTGTAAAAGATGCAAATACATACCCTCAAACTGTAAATGAAGGTATTTGTGTT
WhiteHandedGibbon       ATATCCAATGTAAAAGATGCAAATGCATACCCGCAAACTCTAAATGAAGATATTTGTGTT
Siamang                 ATATCCAATGTAAAAGATGCAAATGCATACCCGCAAACTGTAAATGAAGATATTTGTGTT
WhiteCheekedGibbon      ATATCCAATGTAAAAGATGCAAATGCATACCCGCAAACTGTAAATGAAGATATTTGTGTT
Colobus                 ATATCCAGTGTAAAAGATGCAAATACATACCCTCAAACTGTAAATGAAGGTATTTGTGTT
```

```
OliveBaboon          ATATCCAGTGTAAAAGATGCAAATACATACCCTCAAACTGTAAATGAAGGTATTTGTGTT
Marmoset             ACATTCAATGTGAAAGATGCAAATGCATACCCACAAACTGTAAATGAAGATACTTGTGTT
                     * ** ** *** ************ **** ** ** *** ********* ** *** ***

Bonobo               GAGGAACTTGTGACTAGCTCTTCACCCTGCAAAAATAAAAATGCAGCCATTAAATTGTCC
BorneoOrangutan      GAGGAACTTGTGACTAGCTCTTCACCCTGCAAAAATAAAAATGCAGCCATTAAATTGTCC
Chimpanzee           GAGGAACTTGTGACTAGCTCTTCACCCTGCAAAAATAAAAATGCAGCCATTAAATTGTCC
RedCheekedGibbon     GAGGAACTTGTGACTAGCTCTTCACCCTGCAAAAATAAAAATGCAGCCATTAAATTATCC
Human                GAGGAACTTGTGACTAGCTCTTCACCCTGCAAAAATAAAAATGCAGCCATTAAATTGTCC
AgileGibbon          GAGGAACTTGTGACTAGCTCTTCACCCTGCAGAAATAAAAATGCAGCCATTAAATTATCC
Gorilla              GAGGAACTTGTGACTAGCTCTTCACCCTGCAAAAATAAAAATGCAGCCATTAAATTGTCC
Howler               GAGGAACTTGTGACTAGCTCTTCACCCTGCAAAAATAAAAATAAAGCCATTAAATTGTCC
Rhesus               GAGGAACTTGTGACTAGCTCTTCACCCTGCAAAAATAAAAATGCAGCCATTAAATTGTCC
WolfsGuenon          GAGGAACTTGTGACTAGCTCTTCACCCTGCAAAAATAAAAATGCAGCCATTAAATTGTCC
LeafMonkey           GAGGAACTTGTGGCTAGCTCTTCACCCTGCAAAAATAAAGATGCATCCATTAAATTGTCC
BlackMangabey        GAGGAACTTGTGACTAGCTCTTCACCCTGCAAAAATAAAAATGCAGCCATTAAATTGTCC
PileatedGibbon       GAGGAACTTGTGACTAGCTCTTCACCCTGCAAAAATAAAAATGCAGCCATTAAATTATCC
SquirrelMonkey       GAGGAGCTTGTGACTAGCTCTTCACCCTGCAAAAATAAAAATGCAGCCATTAAATTGTCC
Titi                 GAGGAACTTGTGACTAGCTCTTCACCCTGCAAAAATAAAAATGTAGCCATTAAATTGTCC
Orangutan            GAGGAACTTGTGACTAGCTCTTCACCCTGCGAAAATAAAAATGCAGCCATTAAATTGTCC
CEMacaque            GAGGAACTTGTGACTAGCTCTTCACCCTGCAAAAATGAAAATGCAGCCATTAAATTGTCC
Talapoin             GAGGAACTTGTGACTAGCTCTTCACCCTGCAAAAATAAAAATGCAGCCATTAAATTGTCC
WhiteHandedGibbon    GAGGAACTTGTGACTAGCTCTTCACCCTGCAAAAATAAAAATGCAGCCATTAAATTATCC
Siamang              GAGGAACTTGTGACTAGCTCTTCACCCTGCAAAAATAAAAATGCAGCCATTAAATTATCC
WhiteCheekedGibbon   GAGGAACTTGTGACTAGCTCTTCACCCTGCAAAAATAAAAATGCAGCCATTAAATTATCC
Colobus              GAGGAACTTGTGGCTAGCTCTTCACCCTGCAAAAATAAAGATGCATCCATTAAATTGTCC
OliveBaboon          GAGGAACTTGTGACTAGCTCTTCACCCTGCAAAAATAAAAATGCAGCCATTAAATTGTCC
Marmoset             GAGGAACTTGTGACTAGCTCATCATCCTGCAAAAATAAAAATGCAGCCATTAGATTGTCC
                     ***** ****** ******* *** *****  **** ** **  * ****** *** ***

Bonobo               ATATCTAATAGTAATAATTTTGAGGTAGGGCCACCTGCATTTAGGATAGCCAGTGGTAAA
BorneoOrangutan      ATATCTAATAGTAATAATTTTGCGGTAGGGCCACCTGCATTTAGGATAGCCAGTGGTAAA
Chimpanzee           ATATCTAATAGTAATAATTTTGAGGTAGGGCCACCTGCATTTAGGATAGCCAGTGGTAAA
RedCheekedGibbon     ATATCTAATAGTAATAATTTTGAGGTAGGGCCACCTGCATTTAGTACAGCCAGTGGCAAA
Human                ATATCTAATAGTAATAATTTTGAGGTAGGGCCACCTGCATTTAGGATAGCCAGTGGTAAA
AgileGibbon          ATATCTAATAGTAATAATTTTGAGGTAGAGCCACCTGCATTTAGTACAGCCAGTGGCAAA
Gorilla              ATATCTAATAGTAATAATTTTGAGGTAGGGCCACCTGCATTTAGGATAGCCAGTGGTAAA
Howler               ATACATAACTGTAATAATTTTGAGGTAGCGCCACATGCATTTAGTACAGCCAGTGGTAAA
Rhesus               ATATCTAATTGTAATAATTTTGAGGTAGGGCCACCTGCATTTAGTACAGCCAGTGGTAAA
WolfsGuenon          ATATCTAATTGTAATAATTTTGAGGTAGGGCCACCTGCATTTAGTACAGCCAGTGGTAAA
LeafMonkey           ATATCTGATTGTAGTAATTTTGAGGTAGGGCCACCTGCATTTAGTACAGCCAGTGGTAAA
BlackMangabey        ATATCTAATTGTAATAATTTTGAGGTAGGGCCACCTGCATTTAGTACAGCCAGTGGTAAA
PileatedGibbon       ATATCTAATAGTAATAATTTTGAGGTAGAGCCACCTGCATTTAGTACAGCCAGTGGCAAA
SquirrelMonkey       ATATCTAATTGTAATAATTTTGAGGTAGGGCCGCATGCATTTAGTACAGCCAGTGGTAAA
Titi                 ATATCTAATTGTAATAATTTTGAGGTAGGGCCGCATGCATTTAGTACAGCCAGTGGTAAA
Orangutan            ATATCTAATAGTAATAATTTTGCGGTAGGGCCACCTGCATTTAGGATAGCCAGTGGTAAA
CEMacaque            ATATCTAATTGTAATAATTTTGAGGTAGGGCCACCTGCATTTAGTACAGCCAGTGGTAAA
Talapoin             ATATCTAATTGTAATAATTTTGAAGTAGGGCCACCTGCATTTAGTACAGCCAGTGGTAAA
WhiteHandedGibbon    ATATCTAATAGTAATAATTTTGAGGTAGAGCCACCTGCATTTAGTACAGCCAGTGGCAAA
Siamang              ATATCTAATAGTAATAATTTTGAGGTAGGGCCACCTGCATTTAGTACAGCCAGTGGCAAA
WhiteCheekedGibbon   ATATCTAATAGTAATAATTTTGAGGTAGGGCCACCTGCATTTAGTACAGCCAGTGGCAAA
Colobus              ATGTCTAATTGTAATAATTTTGAGGTAGGGCCACCTGCATTTAGTACAGCCAGTGGTAAA
OliveBaboon          ATATCTAATTGTAATAATTTTGAGGTAGGGCCACCTGCATTTAGTACAGCCAGTGGTAAA
Marmoset             ACGTCTAACTGTAATAATTCTGAGGTAGGGCCACATGCATTTAGTACAGCCAGTGGCAAA
                     *    * *  *** ***** **  **** *** * ********* * ********* ***

Bonobo               ATCATTTGTGTTTCACATGAAACAATTAAAAAAGTGAAAGACATATTTACAGACAGTTTC
BorneoOrangutan      ATCATTTGTGTTTCACATGAAACAATTAAAAAAGTGAAAGACATATTTACAGACAGTTTC
Chimpanzee           ATCGTTTGTGTTTCACATGAAACAATTAAAAAAGTGAAAGACATATTTACAGACAGTTTC
RedCheekedGibbon     ATCATTTGTGTTTCACATGAAACAATTAAAAAAGTGAAAGAGATATTTACAGACAGTTTC
Human                ATCGTTTGTGTTTCACATGAAACAATTAAAAAAGTGAAAGACATATTTACAGACAGTTTC
AgileGibbon          ATCATTTGTGTTTCACATGAAACAATTAAAAAAGTGAAAGAGATATTTACAGACAGTTTC
Gorilla              ATCGTTTGTGTTTCACATGAAACAATTAAAAAAGTGAAAGACATGTTTACAGACAGTTTC
Howler               ATAGTTTGTGTTTCACATGAAACAATTAAAGAAGTGAAAGAGAGATTTACAGACAGTTGC
Rhesus               ATCATTTGTGTTTCACATGAAACAATTAAAAAAGTGAAAGAGATATTTACAGACAGTTTC
WolfsGuenon          ATCATTTGTGTTTCACATGAAACAATTAAAAAAGTGAAAGAGATATTTACAGACAGTTTC
LeafMonkey           ATCATTTGTGTTTCACATGAAACAATTAAAAAAGTGAAAGAGATATTTACAGACAGTTTT
BlackMangabey        ATCATTTGTGTTTCACATGAAACAATTAAAAAAGTGAAAGAGATATTTACAGACAGTTTC
PileatedGibbon       ATCATTTGTGTTTCACATGAAACAATTAAAAAAGTGAAAGAGATATTTACAGACAGTTTC
SquirrelMonkey       ATGGTTTGTGTTTCACATGAAACAATTAAAAAAGTGAAAAAGAGATTTACAGACAGTTGC
Titi                 ATAGTTTGTGTTTCACATGAAACAATTAAAAAAGTGAAAGAGATACTTACAGAGAGTTGC
Orangutan            ATCATTTGTGTTTCACATGAAACAATTAAAAAAGTGAAAGACATATTTACAGACAGTTTC
CEMacaque            ATCATTTGTGTTTCACATGAAACAATTAAAAAAGTGAAAGAGATATTTACAGACAGTTTC
```

```
Talapoin                ATCATTTGTGTTTCACATGAAACAATTAAAAAAGTGAAAGAGATATTTACAGACAGTTTC
WhiteHandedGibbon       ATCATTTGTGTTTCACATGAAACAATTAAAAAAGTGAAAGAGATATTTACAGACAGTTTC
Siamang                 ATCATTTGTGTTTCACATGAAACAATTAAAAAAGTGAAAGAGATATTTACAGACAGTTTC
WhiteCheekedGibbon      ATCATTTGTGTTTCACATGAAACAATTAAAAAAGTGAAAGAGATATTTACAGACAGTTTC
Colobus                 ATCATTTGTGTTTCACATGAAACAATTAAAAAAGTGAAAGAGATATTTACAGACAGTTTT
OliveBaboon             ATCATTTGTGTTTCACATGAAACAATTAAAAAAGTGAAAGAGATATTTACAGACAGTTTC
Marmoset                ATAGTTTGTGTTTCACATGAAACAATTAAAAAAGTGAAAGAGAAATTTACAGACAGTTGC
                        **  ************************** ******** * *   ******* ****

Bonobo                  AGTAAAGTAATTAAGGAAAACAACGAGAATAAATCAAAAATTTGCCAAACGAAAATTATG
BorneoOrangutan         AGTAAAGTAATTAAGGAAAACAATGAGAATAAATCAAAAATTTGCCAAAAGAAAATTGTG
Chimpanzee              AGTAAAGTAATTAAGGAAAACAACGAGAATAAATCAAAAATTTGCCAAACGAAAATTATG
RedCheekedGibbon        AGTAAAGTAATTAAGGAAAACAATGAGAATAAATCAAAAATTTGCCAAACGAAAATTGTG
Human                   AGTAAAGTAATTAAGGAAAACAACGAGAATAAATCAAAAATTTGCCAAACGAAAATTATG
AgileGibbon             AGTAAAGTAATTAAGGAAAACAATGAGAATAAATCAAAAATTTGCCAAACGAAAATTGTG
Gorilla                 AGTAAAGTAATTAAGGAAAACAACGAGAATAAATCAAAAATTTGCCAAACGAAAATTATG
Howler                  AGTAAAGTAATTAAGGAAAACACTGAGAATAAATCAAATAATTGCCAAACAAAAATTCTG
Rhesus                  GGTAAAGTAATTAAGGAAAACAATGAGAATAAATCAAATATTTGCCAAACAAAAATTGTG
WolfsGuenon             GGTAAAGTAATTAAGGAAAACAATGAGAATAAATCAAATATTTGCCAAACAAAAATTGTG
LeafMonkey              GGTAAAGTAATTAAGGAAAACGATGAGAATAAATCAAATATTTGCCAAACAAAAATTGTG
BlackMangabey           GGTAAAGTAATTAAGGAAAACAATGAGAATAAATCAAATATTTGCCAAACAAAAATTGTG
PileatedGibbon          AGTAAAGTAATTAAGGAAAACAATGAGAATAAATCAAAAATTTGCCAAACGAAAATTGTG
SquirrelMonkey          AGTAAAGTAATTAAGGAAAACACTGAGAGTAAATCAAATAATTGCCAAACAAAAATTCTG
Titi                    AGTAAAGTAATTAAGGAAAACACTGAGAATAAATCAAATAATTGCCAAACAAAAATTCTG
Orangutan               AGTAAAGTAATTAAGGAAAACAATGAGAATAAATCAAAAATTTGCCAAAAGAAAATTGTG
CEMacaque               GGTAAAGTAATTAAGGAAAACAATGAGAATAAATCAAATATTTGCCAAACAAAAATTGTG
Talapoin                GGTAAAGTAATTAAGGAAAACAACGAGAATAAATCAAATATTTGCCAAACAAAAATTGTG
WhiteHandedGibbon       AGTAAAGTAATTAAGGAAAACAATGAGAATAAATCAAAAATTTGCCAAACGAAAATTGTG
Siamang                 AGTAAAGTAATTAAGGAAAACAATGAGAATAAATCAAAAAATTGCCAAACGAAAATTGTG
WhiteCheekedGibbon      AGTAAAGTAATTAAGGAAAACAATGAGAATAAATCAAAAATTTGCCAAACGAAAATTGTG
Colobus                 GGTAAAGTAATTAAGGAAAACGATGGGAATAAATCAAATATTTGCCAAACAAAAATTGTG
OliveBaboon             GGTAAAGTAATTAAGGAAAACAATGAGAATAAATCAAATATTTGCCAAACAAAAATTGTG
Marmoset                AGTAAAGTAATTAAGGAAAACACTGAGAATAAATCAAATAATTGCCAAACAAAAATTCTG
                         ********************   * ** ********* * ********  ****** **

Bonobo                  GCAGGTTGTTACGAGGCA---TTGGATGATTCAGAGGATATTCTTCATAACTCTCTAGAT
BorneoOrangutan         GCAGGTTGTTACGAGGCA---TTGGATGATTCTGAGGATATTCTTCATAACTCTCTAGAT
Chimpanzee              GCAGGTTGTTACGAGGCA---TTGGATGATTCAGAGGATATTCTTCATAACTCTCTAGAT
RedCheekedGibbon        GCAGGTTGTTACGATGATTGTATGGATGATTCAGAGGATATTTTTCATAACTCTCTAGAT
Human                   GCAGGTTGTTACGAGGCA---TTGGATGATTCAGAGGATATTCTTCATAACTCTCTAGAT
AgileGibbon             GCAGGTTGTTACGATGATTGTATGGATGATTCAGAGGATATTTTTCATAACTCTCTAGAT
Gorilla                 GCAGGTTGTTACGAGGCA---TTGGATGATTCAGAGGATATTCTTCATAACTCTCTAGAT
Howler                  GCAGGTTGTTACAAGGCA---TTGAATGATTCAGAGGATATTTTTCATAGTTCTCCAGAT
Rhesus                  GCAGGTTGTTACGAGGCA---TTGGATGATTCAGAGGATATTTTTCATAACTCTCTAGAT
WolfsGuenon             GCAGGTTGTTACGAGGCA---TTGGATGATTCAGAGGATATTTTTCATAACTCTCTAGAT
LeafMonkey              GCACGTTGTTACGAGGCA---TTGGATGATTCAGAGGATATTTTTCATAACTCTCTAGAT
BlackMangabey           GCAGGTCGTTACGAGGCA---TTGGATGATTCAGAGGATATTTTTCATAACTCTCTAGAT
PileatedGibbon          GCAGGTTGTTACGATGATTGTATGGATGATTCAGAGGATATTTTTCATAACTCTCTAGAT
SquirrelMonkey          GCAGGT---TACAAGGCA---TTGAATGATTCAGAGGATATTTTTCATAATTCTTCAGAT
Titi                    GCAGGTTGTTACAAGACA---TTGAATGATTCAGAGGATATTTTTCATAATTCTCCAGAT
Orangutan               GCAGGTTGTTACGAGGCA---TTGGATGATTCTGAGGATATTCTTCATAACTCTCTAGAT
CEMacaque               GCAGGTTGTTACGAGGCA---TTGGATGATTCAGAGGATATTTTTCATAACTCTCTAGAT
Talapoin                GCAGGT---TACGAGGCA---TTGGATGATTCAGAGGATATTTTTCATAACTCTCTAGAT
WhiteHandedGibbon       GCAGGTTGTTACGATGATTGTATGGATGATTCAGAGGATATTTTTCATAACTCTCTAGAT
Siamang                 GCAGGTTGTTACGATGATTGTATGGATGATTCAGAGGATATTTTTCATAACTCTCTAGAT
WhiteCheekedGibbon      GCAGGTTGTTACGATGATTGTATGGATGATTCAGAGGATATTTTTCATAACTCTCTAGAT
Colobus                 GCACGTTGTTACGAGGCA---TTGGATGATTCAGAGGATATTTTTCATAACTCTCTAGAT
OliveBaboon             GCAGGTCGTTACGAGGCA---TTGGATGATTCAGAGGATATTTTTCATAACTCTCTAGAT
Marmoset                GCAGGTTGTTACAAGGCA---TTAAATGAATCAGAGGATATTTTTCATAATTCTTCAGGT
                        *** **   *** *        *  **** ** ********* ******  ***  ** *

Bonobo                  AATGATGAATGTAGCACGCATTCACATAAGGTTTTTGCTGACATTCAGAGTGAAGAAATT
BorneoOrangutan         AATGATGAATGTAGCATGCATTCACATAAGGTTTTTGCTGACATTCAAAGTGAAGAAGTT
Chimpanzee              AATGATGAATGTAGCACGCATTCACATAAGGTTTTTGCTGACATTCAGAGTGAAGAAATT
RedCheekedGibbon        AATGATGAATGTAGCATGCATTCACATAAGGTTTTTGCTGACATTCAAAGTGAAGAAATT
Human                   AATGATGAATGTAGCACGCATTCACATAAGGTTTTTGCTGACATTCAGAGTGAAGAAATT
AgileGibbon             AATGATGAATGTAGCATGCATTCACATAAGGTTTTTGCTGACATTCAAAGTGAAGAAATT
Gorilla                 AATGATGAATGTAGCACGCATTCACATAAGGTTTTTGCTGACATTCAGAGTGAAGAAATT
Howler                  AATGATGAATGTAGTATGCATTCACATAAGGTTTTTGCTGACGTTCAAAGTGAAGAAATT
Rhesus                  AGTGATGAGTGTAGCATGCATTCACATAAGGTTTTTGCTGACATTCAAAGTGAAGAAATT
WolfsGuenon             AGTGATGAGTGTAGCATGCATTCACATAAGGTTTTTGCTGACATTCAAAGTGAAGAAATT
LeafMonkey              ACTGATGAATGTAGCATGCATTCACATAAGGTTTTTGCTGACATTCAAAGTGAAGAAATT
BlackMangabey           AGTGATGAGTGTAGCATGCATTCACATAAGGTTTTTGCTGACATTCAAAGTGAAGAAATT
```

```
PileatedGibbon         AATGATGAATGTAGCATGCATTCACATAAGGTTTTTGCTGACATTCAAAGTGAAGAAATT
SquirrelMonkey         AACGATGAATGTAGCATGCATTCACATAAGGTTTTTGCTGACATTCAAAATGAAGAAATT
Titi                   AATGATGAATGTAGCATGCATTCACATAAGGTTTTTGCTGACATTCAAACTGAAGAAATT
Orangutan              AATGATGAATGTAGCATGCATTCACATAAGGTTTTTGCTGACATTCAAAGTGAAGAAGTT
CEMacaque              AGTGATGAGTGTAGCATGCATTCACATAAGGTTTTTGCTGACATTCAAAGTGAAGAAATT
Talapoin               AGTGATGAGTGTAGCATGCATTCACATAAGGTTTTTGCTGACATTCAAGGTGAA---ATT
WhiteHandedGibbon      AATGATGAATGTAGCACGCATTCACATAAGGTTTTTGCTGACATTCAAAGTGAAGAAATT
Siamang                AATGATGAATGTAGCATGCATTCACATAAGGTTTTTGCTGACATTCAAAGTGAAGAAATT
WhiteCheekedGibbon     AATGATGAATGTAGCATGCATTCACATAAGGTTTTTGCTGACATTCAAAGTGAAGAAATT
Colobus                AGAGATGAGTGTAGCATGCATTCACCTAAGGGTTTTGCTGACATTCAAAGTGAAGAAATT
OliveBaboon            AGTGATGAGTGTAGCATGCATTCACATAAGGTTTTTGCTGACATTCAAAGTGAAGAAATT
Marmoset               AATGATGAATGTAGCATGCATTCACATAAAGTTTTTGCTGACATTCAAAGTGAAGAAATT
                       *  ***** ***** * ******** *** * ********** ****   ****    **

Bonobo                 TTACAACATAACCAAAATATGTCTGGGTTGGAGAAAGTTTCTAAAATATCACCTTGTGAT
BorneoOrangutan        TTACAACATAACCAAAATATGTCTGGATTGGAGAAAGTTTCTAAAATATCAGCTTGTGAT
Chimpanzee             TTACAACATAACCAAAATATGTCTGGGTTGGAGAAAGTTTCTAAAATATCACCTTGTGAT
RedCheekedGibbon       TTACAACATAACCAAAATATGTCTGAACTGGAGAAAGTTTCTAAAATATCACCTTGTGAT
Human                  TTACAACATAACCAAAATATGTCTGGATTGGAGAAAGTTTCTAAAATATCACCTTGTGAT
AgileGibbon            TTACAACATAACCAAAATATGTGTGAACTGGAGAAAGTTTCTAAAATATCACCTTGTGAT
Gorilla                TTACAACATAACCAAAATATGTCTGGATTGGAGAAAGTTTCTAAAATATCACCTTGTGAT
Howler                 TTACAACATAACCAAAAAATGTCTGGAGTGGAGAAAGTTTCTAAAATACCACCTTGTGAT
Rhesus                 TTACAACATAACCAAAATATGTCTGGATTGGAGCAAGTTTCTAAAATGTCACCTTTTGAT
WolfsGuenon            TTACAACATAACCAAAATATGTCTGGATTGGAGCAAGTTTCTAAAGTGTCACCTTGTGAT
LeafMonkey             TTACAACATAACCAAAATATGTCTGGATTGGAGCAAGTTTCTAAAATGTCACCTTGTGAT
BlackMangabey          TTACAACATAACCAAAATATGTCTGGATTGGAGCAAGTTTCTAAAATGTCACCTTGTGAT
PileatedGibbon         TTACAACATAACCAAAATATGTGTGAACTGGAGAAAGTTTCTAAAATATCACCTTGTGAT
SquirrelMonkey         TTACAACATAGCCAAAAAAAGTCTGGAGTGGAGAAAGTTTCTAAAATACCACCTTGTGAT
Titi                   TTGCAACATAACCAAAGAACGTCTGGAGTGGAGAAAGTTTCTAAAATACCACCTTGTGAT
Orangutan              TTACAACATAACCAAAATATGTCTGGATTGGAGAAAGTTTCTAAAATATCAGCTTGTGAT
CEMacaque              TTACAACATAACCAAAATATGTCTGGATTGGAGCAAGTTTCTAAAATGTCACCTTTTGAT
Talapoin               TTACAGGATAACCAAAATATGTCTGGATTGGAGCAAGTTTCTAAAATGTCACCTTGTAAA
WhiteHandedGibbon      TTACAACATAACCAAAATATGTGTGAACTGGAGAAAGTTTCTAAAATATCACCTTGTGAT
Siamang                TTACAACATAACCAAAATATGTGTGAACTGGAGAAAGTTTCTAAAATATCACCTTGTGAT
WhiteCheekedGibbon     TTACAACATAACCAAAATATGTCTGAACTGGAGAAAGTTTCTAAAATATCACCTTGTGAT
Colobus                TTACAACATAACCAAAATATGTCTGGATTGGAGCAAGTTTCTAAAATGTCACCTTGTGAT
OliveBaboon            TTACAACATAACCAAAATATGTCTGGATTGGAGCAAGTTTCTAAAATATCACCTTGTGAT
Marmoset               TTACAACATAACCAAAAAATGTTTGGAGTGGAGAAAGTTTCTGAAATACCACCTTGTGAT
                       ** **  *** *****  * ** **   ***** ******** ** *  ** *** * *

Bonobo                 GTTAGTTTGGAAACTTCAGATATATGTAAATGTAGTATAGGGAAGCTTCATAAGTCAGTC
BorneoOrangutan        GTTAGTTTGGAAACTTCAGATATATGTAAATGTAGTATAGGGAAGCTTCGTAAGTCAGTC
Chimpanzee             GTTAGTTTGGAAACTTCAGATATATGTAAATGTAGTATAGGGAAGCTTCATAAGTCAGTC
RedCheekedGibbon       GTTAGTTTGGAAACTTCAGATATATGTAAATGTAGTATAGGGAAGCTTCCTAAGTCAGTC
Human                  GTTAGTTTGGAAACTTCAGATATATGTAAATGTAGTATAGGGAAGCTTCATAAGTCAGTC
AgileGibbon            GTTAGTTTGGAAACTTCAGATATATGTAAATGTAGTATAGGGAAGCTTCCTAAGTCAGTC
Gorilla                GTTAGTTTGGAAACTTCAGATATATGTAAATGTAGTATAGGGAAGCTTCATAAGTCAGTC
Howler                 GTTGGTTTGGAAACTTCAGATATATATAAATTTAGTATAGGGAAGCTTCCTAAGTCAGTC
Rhesus                 GTTAGTTTGGAAACTTCAGATATATGTAAATGTAGTATAGGGAAGCTTCCTAAGTCAGTC
WolfsGuenon            GTTAGTTTGGAAACTTCAGATATATGTAAATGTAGTATAGGGAAGCTTCCTAAGTCAGTC
LeafMonkey             ATTAGTTTGGAAACTTCAGATATACATAAATGTAGTATAGAGAAGCTTCCTAAATCAGTC
BlackMangabey          GTTAGTTTGGAAACTTCAGATATATGTAAATGTAGTATAGGGAAGTTTCCTAAGTCAGTC
PileatedGibbon         GTTAGTTTGGAAACTTCAGATGTATGTAAATGTAGTATAGGGAAGCTTCCTAAGTCAGTC
SquirrelMonkey         GTTAGTTTGGAAACTTCAGATATATATAAATTTAGTATAGGGAAGCTTCCTAAGTCAGTC
Titi                   GTTAGTTTGGAAACTTCAGATACATATAAATTTAGTATAGGGAAGCTTCCTAAGTCAGTC
Orangutan              GTTAGTTTGGAAACTTCAGATATATGTAAATGTAGTATAGGGAAGCTTCCTAAGTCAGTC
CEMacaque              GTTAGTTTGGAAACTTCAGATATATGTAAATGTAGTATAGGGAAGCTTCCTAAGTCAGTC
Talapoin               GTTAGTTTGGAAACTTCAGATACATGTAAATGT---ATAGGGAAGCTTCCTAAGTCAGTC
WhiteHandedGibbon      GTTAGTTTGGAAACTTCAGATATATGTAAATGTAGTATAGGGAAGCTTCCTAAGTCAGTC
Siamang                GTTAGTTTGGAAACTTCAGATATATGTAAATGTAGTATAGGGAAGCTTCCTAAGTCAGTC
WhiteCheekedGibbon     GTTAGTTTGGAAACTTCAGATATATGTAAATGTAGTATAGGGAAGCTTCCTAAGTCAGTC
Colobus                GTTAGTTTGGAAACTTCAGATATACGTAAATGTAGTATAGAGAAGCTTCCTAAGTCAGTC
OliveBaboon            GTTAGTTTGGAAACTTCAGATATATGTAAATGTAGTATAGGGAAGTTTCCTAAGTCAGTC
Marmoset               GTTAGTTTGGAAACTTCAGGTATATATAAATTTAGTATGGGGAAGCTTCCTAAGTCAGTC
                        ** *************** *  *  ***** *   ** * **** *** *** ******

Bonobo                 TCATCTACAAATACTTGTGGGATTTTTAGCACAGCAAGTGGAAAATCTGTCCAGGTATCA
BorneoOrangutan        TCATCTACAAATACTTGTGGGATTTTTAGCACAGCAAGTGGAAAATCTGTCCAGATATCA
Chimpanzee             TCATCTACAAATACTTGTGGGATTTTTAGCACAGCAAGTGGAAAATCTGTCCAGGTATCA
RedCheekedGibbon       TCATCTACAAATACTTGTGGGATGTTTAGCACAGGAAGTGGAAAATCAATCCAGGTATCA
Human                  TCATCTGCAAATACTTGTGGGATTTTTAGCACAGCAAGTGGAAAATCTGTCCAGGTATCA
AgileGibbon            TCATCTACAAATACTTGTGGGATTTTTAGCACAGGAAGTGGAAAATCGATCCAGGTATCA
Gorilla                TCATCTACAAATACTTGTGGGATTTTTAGCACAGCAAGTGGAAAATCTGTCCAGGTATCA
```

```
Howler                  TCGTCTACAAATTCTTGTGGGATTTTTAGCACAGCAAGTGGAAAATCTGTACAGGTATCA
Rhesus                  CCATCTACAAATACTTGTGGGATTTTTAGCACAGCAAGTGGAAAATCTGTCCAGGTATCA
WolfsGuenon             CCATCTACAAATACTTGTGGGATTTTTAGCACAGCAAGTGGAAAATCTGTCCAGGTATCA
LeafMonkey              CCGTCTACAAATACTTGTGGGATTTTTAGCACAGCAAGTGGAAAATCTGTCCAGGTATCA
BlackMangabey           CCATCTACAAATACTTGTGGGATTTTTAGCACAGCAAGTGGAAAATCTGTCCGGGTATCA
PileatedGibbon          TCATCTACAAATACTTGTGGGATTTTTAGCACAGGAAGTGGAAAATCGATCCAGGTATCA
SquirrelMonkey          TCATCTACAAATACTTGTGGGATTTTCAGCACAGCAAGTGGAAAATCTGTACAGGTATCA
Titi                    TCGTCTACAAATGCTTGTGGGATTTTTAGCACAGCAAGTGGAAAATCTGTACAGGTATCA
Orangutan               TCATCTACAAATACTTGTGGGATTTTTAGCACAGCAAGTGGAAAATCTGTCCAGATATCA
CEMacaque               CCATCTACAAATACTTGTGGGATTTTTAGCACAGCAAGTGGAAAATCTGTCCAGGTATCA
Talapoin                CCATCTACAAATACTTGTGGGATTTTTAGCACAGCAAGTGGAAAATCTGTCCAGGTATCA
WhiteHandedGibbon       TCATCTACAAATACTTGTGGGATTTTTAGCACAGGAAGTGGAAAATCGATCCAGGTATCA
Siamang                 TCATCTACAAATACTTGTGGGATTTTTAGCACAGGAAGTGGAAAATCGATCCAGGTATCA
WhiteCheekedGibbon      TCATCTACAAATACTTGTGGGATGTTTAGCACAGGAAGTGGAAAATCGATCCAGGTATCA
Colobus                 CCGTCTACAAATACTTGTGGGATTTTTAGCACAGCAAGTGGAAAATCTGTCCAGGTATCA
OliveBaboon             CCATCTACAAATACTTGTGGGATTTTTAGCACAGCAAGTGGAAAATCTGTCCAGGTATCA
Marmoset                TTGTCTACAAATACTTGTGGGATTTTTAGCACAGCAAGTGGAAAATCTGTACAGGTATCA
                           *** ***** ********** ** ******* ***********   * * * *****

Bonobo                  GATGCTTCATTACAAAACGCAAGACAAGTGTTTTCTGAAATAGAAGACAGTACCAAGCAA
BorneoOrangutan         GATGCTTCATTACAAAAGGCAAGACAAGTGTTTTCTGAAATAGAAGATAGTACCAAGCAA
Chimpanzee              GATGCTTCATTACAAAACGCAAGACAAGTGTTTTCTGAAATAGAAGACAGTACCAAGCAA
RedCheekedGibbon        GATGCTTCATTACAAAAAGCAAGACAAGTGTTTTCTGAAATAGAAGATAGTACCAAGCAA
Human                   GATGCTTCATTACAAAACGCAAGACAAGTGTTTTCTGAAATAGAAGATAGTACCAAGCAA
AgileGibbon             GATGCTTCATTACAAAAAGCAAGACAAGTGTTTTCTGAAATAGAAGATAGTACCAAGCAA
Gorilla                 GATGCTTCATTACAAAACGCAAGACAAGTGTTTTCTGAAATAGAAGATAGTACCAAGCAA
Howler                  AACGCTTCATTACAAAAGGCAAGACAAGTGTTTTCTAAGATAGACGATAGTACCAAGCAA
Rhesus                  GATGCTTCATTACAAAAGGCAAGACAAGTGTTTTCTGAAATAGAAGATAGTACCAAGCAA
WolfsGuenon             GATGCTTCATTACAAAAGGCAAGACAAGTGTTTTCTGAAATAGAAGATAGTACCAAGCAA
LeafMonkey              GATGCTTCATTACAAAAGGCAAGACAAGTGTTTTCTGAAATAGAAGATAGTACCAAGCAA
BlackMangabey           GATGCTTCATTACAAAAGGCAAGACAAGTGTTTTCTGAAATAGAAGATAGTACCAAGCAA
PileatedGibbon          GATGCTTCATTACAAAAAGCAAGACAAGTGTTTTCTGAAATAGAAGATAGTACCAAGCAA
SquirrelMonkey          AATGCTTCATTACAAAAGGCAAGACAAGTGTTTTCTGAGATAGACGATAGTACCAAGCAA
Titi                    AACGCTTCATTACAAAAGGCAAGACAAGTGTTTTCTGAGATAGACGATAGTACCAAGCAA
Orangutan               GATGCTTCATTACAAAAGGCAAGACAAGTGTTTTCTGAAATAGAAGATAGTACCAAGCAA
CEMacaque               GATGCTTCATTACAAAAGGCAAGACAAGTGTTTTCTGAAATAGAAGATAGTACCAAGCAA
Talapoin                GATGCTTCATTACAAAAGGCAAGACAAGTGTTTTCTGAAATAGAAGATAGTACCAAGCAA
WhiteHandedGibbon       GATGCTTCATTACAAAAAGCAAGACAAGTGTTTTCTGAAATAGAAGATAGTACCAAGCAA
Siamang                 GATGCTTCATTACAAAAAGCAAGACAAGTGTTTTCTGAAATAGAAGATAGTACCAAGCAA
WhiteCheekedGibbon      GATGCTTCATTACAAAAAGCAAGACAAGTGTTTTCTGAAATAGAAGATAGTACCAAGCAA
Colobus                 GATGCTTCATTACAAAAGGCAAGACAAGTGTTTTCTGAAATAGAAGATAGTACCAAGCAA
OliveBaboon             GATGCTTCATTACAAAAGGCAAGACAAGTGTTTTCTGAAATAGAAGATAGTACCAAGCAA
Marmoset                AATGCTTCATTGCAAAAGGCAAGACAAGTGTTTTCTGAGATAGATGATAGTACCAAGCAA
                         * ******** ***** ****************** * ***** ** ************

Bonobo                  GTCTTTTCCAAAGTATTGTTTAAAAGTAACGAACATTCAGACCAGCTCACAAGAGAAGAA
BorneoOrangutan         GTCTTTTCCAAAGTATTGTTTAAGAGTAACGAACATTCAGACCAGCTCACAAGACAAGAA
Chimpanzee              GTCTTTTCCAAAGTATTGTTTAAAAGTAACGAACATTCAGACCAGCTCACAAGAGAAGAA
RedCheekedGibbon        GTCTTTTCCAAAGTATTGTTTAAAAGTAACGAACATTCAGACCAGCTCACAAGAGAAGAA
Human                   GTCTTTTCCAAAGTATTGTTTAAAAGTAACGAACATTCAGACCAGCTCACAAGAGAAGAA
AgileGibbon             GTCTTTTCCAAAGTATTGCTTAAAAGTAACGAACATTCAGACCAGCTCACAAGAGAAGAA
Gorilla                 GTCTTTTCCAAAGTATTGTTTAAAAGTAACGAACATTCAGACCAGCTCACAAGAGAAGAA
Howler                  CTCTTTTCCAAAGTAGTGTTTAAAAATAATGAACATTCAGACAAGCTCACAAGAGAAGAA
Rhesus                  GTCTTTTCCAAAGTATTGTTTAAAAGTAATGAACATTCAGACCAGCTCACAAGAGAAGAA
WolfsGuenon             GTCTTTTCCAAAGTATTGTTTAAAAGTAATGAACATTCAGACCAGCTCACAAGAGAAGAA
LeafMonkey              GTCTTTTCCAAAGTATTGTTTAAAAGTAATGAACATTCAGACCAGCTCACAAGAGAAGAA
BlackMangabey           GTCTTTTCCAAAGTATTGTTTAAAAGTAATGAACATTCAGACCAGCTCACAAGAGAAGAA
PileatedGibbon          GTCTTTTCCAAAGTATTGCTTAAAAGTAACGAACATTCAGACCAGCTCACAAGAGAAGAA
SquirrelMonkey          CTCTTTTCCAAAGTATTGTTTAAAAATAATGAACATTCAGACCAGCTCACAAGAGAAGAG
Titi                    CTCTTTTCCAAAGTATTGTTTAAAAATAATGAACATTCAGACCAGCTCACAAGAGAAGAA
Orangutan               GTCTTTTCCAAAGTATTGTTTAAGAGTAACGAACATTCAGACCAGCTCACAAGACAAGAA
CEMacaque               GTCTTTTCCAAAGTATTGTTTAAAAGTAATGAACATTCAGACCAGCTCACAAGAGAAGAA
Talapoin                GTCTTTTCCAAAGTATTGTTTAAAAGTAATGAACATTCAGACCAGCTCACAAGAGAAGAA
WhiteHandedGibbon       GTCTTTTCCAAAGTATTGCTTAAAAGTAACGAACATTCAGACCAGCTCACAAGAGAAGAA
Siamang                 GTCTTTTCCAAAGTATTGTTTAAAAGTAACGAACATTCAGACCAGCTCACAAGAGAAGAA
WhiteCheekedGibbon      GTCTTTTCCAAAGTATTGTTTAAAAGTAACGAACATTCAGACCAGCTCACAAGAGAAGAA
Colobus                 GTCTTTTCCAAAGGATTGTTTAAAAGTAATGAACATTCAGACCAGCTCACAAGAGAAGGA
OliveBaboon             GTCTTTTCCAAAGTATTGTTTAAAAGTAATGAACATTCAGACCAGCTCACAAGAGAAGAA
Marmoset                CTCTCTTCCGAAGTATTGTTTACAAATAATGAACATTCAGACCAGCTCACAAGAGAAGAA
                         *** **** *** * ** ***  * *** ************ *********** ***

Bonobo                  AATACTGCTATACGTACTCCAGAACATTTAATA---TCCCAAAAAGGCTTTTCATATAAT
BorneoOrangutan         AATACTGCTATACATACTCCAGAAAATTTAACA---TCCCAAAAAGGCTTTTCATATAAT
```

```
Chimpanzee          AATACTGCTATACGTACTCCAGAACATTTAATA---TCCCAAAAAGGCTTTTCATATAAT
RedCheekedGibbon    AATACTGCTATACATACTCCAAAACATTTAATATCATCCCAAAAAGGCTTTTCATATAAT
Human               AATACTGCTATACGTACTCCAGAACATTTAATA---TCCCAAAAAGGCTTTTCATATAAT
AgileGibbon         AATACTGCTATACATACTCCAAAACATTTAATATCATCCCAAAAAGGCTTTTCATATAAT
Gorilla             AATACTGCTATACGTACTCCAGAACATTTAATA---TCCCAAAAAGGCTTTTCATATAAT
Howler              AATACTACTACACAGACGGCAGAACATTTGATATCATCCCATAAAGGCTTTTCATATAAT
Rhesus              AATACTACTATACATACTCCAAAACATTTAATATCATCCCAAAAAGACTTTTCACATAAT
WolfsGuenon         AATACTACTATACATACTCCAAAACATTTAATATCATCCCAAAAAGACTTTTCATATAAT
LeafMonkey          AATACTACTATACATACTCCAAAACATTTAATATCATCCCAAAAAGACTTTTCATATAAT
BlackMangabey       AATACTACTATACATACTCCAAAACATTTAATATCATCCCAAAAAGACTTTTCATATAAT
PileatedGibbon      AATACTGCTATACATACTCCAAAACATTTAATATCATCCCAAAAAGGCTTTTCATATAAT
SquirrelMonkey      AATACTACTACACAGACGGCAGAACATTTGATATCATCCCAAAAAGGCTTTTCATATAAT
Titi                AATACTACTACACAGATGGCAGAACATTTGATATCATCCCAAAAAGGCTTTTCATATAAT
Orangutan           AATACTGCTATACATACTCCAGAAAATTTAACA---TCCCAAAAAGGCTTTTCATATAAT
CEMacaque           AATACTACTATACATACTCCAAAACATTTAATATCATCCCAAAAAGACTTTTCACATAAT
Talapoin            AATACTACTATACATACTCCAAAACATTTAATATCATCCCAAAAAGACTTTTCATATAAT
WhiteHandedGibbon   AATACTGCTATACATACTCCAAAACATTTAATATCATCCCAAAAAGGCTTTTCATATAAT
Siamang             AATACTGCTATACATACTCCAAAACATTTAATATCATCCCAAAAAGGCTTTTCATACAAT
WhiteCheekedGibbon  AATACTGCTATACATACTCCAAAACATTTAATATCATCCCAAAAAGGCTTTTCATATAAT
Colobus             AATACTACTATACATACTCCAAAACATTTAATATCATCCCAGAAAGACTTTTCATATAAT
OliveBaboon         AATACTACTATACATACTCCAAAACATTTAATATCATCCCAAAAAGACTTTTCATATAAT
Marmoset            AATACTGCTACACAGATAGCAGAACATTTGATACCATCCCAAAAAGGCTTTTCATATAAT
                    ****** *** **  *   ** ** **** * *   ***** **** ******* * ***

Bonobo              GTGGTAAATTCATCTGCTTTCTCTGGATTTAGTACAGCAAGTGGAAAGCAAGTTTCCATT
BorneoOrangutan     GTGGTAAATTCATCTGCTTTCTCTGGATTTAGTACAGCAAGTGGAAAGCAAGTTTCCATT
Chimpanzee          GTGGTAAATTCATCTGCTTTCTCTGGATTTAGTACAGCAAGTGGAAAGCAAGTTTCCATT
RedCheekedGibbon    GTGGTAAATTCATCTGCTTTCTCTGGATTTAGTACAGCAAGTGGAAGGCAAGTTTCCATT
Human               GTGGTAAATTCATCTGCTTTCTCTGGATTTAGTACAGCAAGTGGAAAGCAAGTTTCCATT
AgileGibbon         GTGGTAAATTCATCTGCTTTCTCTGGATTTAGTACAGCAAGTGGAAGGCAAGTTTCCATT
Gorilla             GTGGTAAATTCATCTGCTTTCTCTGGATTTAGTACAGCAAGTGGAAAGCAAGTTTCCATT
Howler              GTGGTAAATTCATCTACTTTTACTGGATTTAATACAGCAAGTGGAAAGCAAGTTTCCGTT
Rhesus              GTGGTAAATTCATCTGCTTTCTCTGGATTTAGTACAGCAAGTGGAAAGCAAGTTTCCATT
WolfsGuenon         GTGGTAAATTCATCTGCTTTCTCTGGATTTAGTACAGCAAGTGGAAAGCAAGTTTCCATT
LeafMonkey          GTGGTAAATTCATCTGCTTTCTCTGGATTTAGTACAGCAAGTGGAAAGCAAGTTTCCATT
BlackMangabey       GTGGTAAATTCATCTGCTTTCTCTGGATTTAGTACAGCAAGTGGAAAGCAAGTTTCCATT
PileatedGibbon      GTGGTAAATTCATCTGCTTTCTCTGGATTTAGTACAGCAAGTGGAAGGCAAGTTTCCATT
SquirrelMonkey      ATGGTAAATTCATCTGCTTTTTCTGGATTTAATACAGCAAGTGGAAAGCAAGTTTCCATT
Titi                GTGGTAAATTCATCTGCTTTCTCTGGATTTAATACAGCAAGTGGAAAGCAAGTTTCCATT
Orangutan           GTGGTAAATTCATCTGCTTTCTCTGGATTTAGTACAGCAAGTGGAAAGCAAGTTTCCATT
CEMacaque           GTGGTAAATTCATCTGCTTTCTCTGGATTTAGTACAGCAAGTGGAAAGCAAGTTTCCATT
Talapoin            GTGGTAAATTCATCTGCTTTCTCTGGATTTAGTACAGCAAGTGGAAAGCAAGTTTCCATT
WhiteHandedGibbon   GTGGTAAATTCATCTGCTTTCTCTGGATTTAGTACAGCAAGTGGAAGGCAAGTTTCCATT
Siamang             GTGGTAAATTCATCTGCTTTCTCTGGATTTAGTACAGCAAGTGGAAGGCAAGTTTCCATT
WhiteCheekedGibbon  GTGGTAAATTCATCTGCTTTCTCTGGATTTAGTACAGCAAGTGGAAGGCAAGTTTCCATT
Colobus             GAGGTACAATCATCTGCTTTCTCTGGATTTAGTACAGCAAGTGGAAAGCAAGTTTCCATT
OliveBaboon         GTGGTAAATTCATCTGCTTTCTCTGGATTTAGTACAGCAAGTGGAAAGCAAGTTTCCATT
Marmoset            ---GTAAATTCATCTGCTTTTTCTGGATTTAATACAGCAAGTGGAAAGCAAGTTTCCATT
                       *** * ****** ****  ********* ************** ********** **

Bonobo              TTAGAAAGTTCCTTACACAAAGTTAAGGGAGTGTTAGAGGAATTTGATTTAATCAGAACT
BorneoOrangutan     TTAGAAAGTTCCTTACACGAAGTTAAGGGAGTGTTAGAGGAATTTGATTTAATCAGAACT
Chimpanzee          TTAGAAAGTTCCTTACACAAAGTTAAGGGAGTGTTAGAGGAATTTGATTTAATCAGAACT
RedCheekedGibbon    TCAGAAAGTTCCTTACGCAAAGTTAAGGGAATGTTAGAAGAATTTGATTTAATCAGAACT
Human               TTAGAAAGTTCCTTACACAAAGTTAAGGGAGTGTTAGAGGAATTTGATTTAATCAGAACT
AgileGibbon         TCAGAAAGTTCCTTACGCAAAGTTAAGGGAATGTTAGAGGAATTTGATTTAATCAGAACT
Gorilla             TTAGAAAGTTCCTTACACAAAGTTAAGGGAGTGTTAGAGGAATTTGATTTAATCAGAACT
Howler              TCAGAAAGTGCCTTACACAAAGTTAAGGGAATGTTAGAGGAATTTGATTTAATCAGAGCT
Rhesus              TCAGAAAGTTCCTTACACAAAGTTAAGGGAATGTTAGAGGAATTTGATATAATCAGAACT
WolfsGuenon         TCAGAAAGTTCGTTACACAAAGTTAAGGGAATGTTAGAGGAATTTGATATAATCAGAACT
LeafMonkey          TCAGAAAGTTCCTTACACAAAGTTAAGGGAATGTTAGAAGAATTTGATATAATCAGAACT
BlackMangabey       TCCGAAAGTTCCTTACACAAAGTTAAAGGAATGTTAGAGGAATTTGATATAATCAGAACT
PileatedGibbon      TCAGAAAGTTCCTTACGCAAAGTTAAGGGAATGTTAGAGGAATTTGATTTAATCAGAACT
SquirrelMonkey      TCAGAAAGTGCCTTACACAAAGTTAAGGGAATGTTAAAGGAATTTGATTTAATCAGAACT
Titi                TCAGAAAGTGCCTTACACAAAGTTAAGGGAATGTTAGAGGAATTTGATTTAATCAGAACT
Orangutan           TTAGAAGGTTCCTTACACGAAGTTAAGGGAGTGTTAGAGGAATTTGATTTAATCAGAACT
CEMacaque           TCAGAAAGTTCCTTACACAAAGTTAAGGGAATGTTAGAGGAATTTGATATAATCAGAACT
Talapoin            TCAGAAAGTTCCTTACACAAAGTTAAGGGAATGTTAGAGGAATTTGATATAATCAGAACT
WhiteHandedGibbon   TCAGAAAGTTCCTTATGCAAAGTTAAGGGAATGTTAGAGGAATTTGATTTAATCAGAACT
Siamang             TCAGAAAGTTCCTTACGCAAAGTTAAGGGAATGTTAGAGGAATTTGATTTAATCAGAACT
WhiteCheekedGibbon  TCAGAAAGTTCCTTACGCAAAGTTAAGGGAATGTTAGAAGAATTTGATTTAATCAGAACT
Colobus             TCAGAAAGTTCCTTACACAAAGTTAAGGGAATGTTAGAGGAATTTGATATAATCAGAACT
OliveBaboon         TCCGAAAGTTCCTTACACAAAGTTAAAGGAATGTTAGAGGAATTTGATATAATCAGAACT
```

```
Marmoset                TCAGAAAGTGCCTTGCACAAAGTTAAGGGAATGTTAGAGGAATTTGATTTAATCAGAACT
                        *  *** ** * **   * ******* *** ***** * ********* ******** **

Bonobo                  GAGCATAGTCTTCACTATTCACCTACGTCTAGACAAAATGTATCAAAAATACTTCCTCGT
BorneoOrangutan         GAGCATAGTCTTCACTATTCACCTACGTCTAGACAAAATGTATCAAAAATACTTCCTTGT
Chimpanzee              GAGCATAGTCTTCACTATTCACCTACGTCTAGACAAAATGTATCAAAAATACTTCCTCGT
RedCheekedGibbon        GAGCATAGTCTTCACTATTCACCAACATCTAGACAAAATGTATCAAAAATACTTCCTTGT
Human                   GAGCATAGTCTTCACTATTCACCTACGTCTAGACAAAATGTATCAAAAATACTTCCTCGT
AgileGibbon             GAGCATAGTCTTCACTATTCACCTACATCTAGACAAAATGTATCAAAAATACTTCCTTGT
Gorilla                 GAGCATAGTCTTCACTATTCACCTACGTCTAGACAAAATGTATCAAAAATACTTCCTCGT
Howler                  GAGCATAGTCTTCACTATTCACCTATATCTAGACAAAATGTATCAAAAATACTTCCTTGT
Rhesus                  GAGCATAGTCTTCACTATTCACCTACATCTAGACAAAATGTATCAAAAATACTTCCTTGT
WolfsGuenon             GAGCATAGTCTTCACTATTCACCTACATCTGGACAAAATGTATCAAAAATACTTCCTTGT
LeafMonkey              GAGCATAGTCTTCACTATTCACCTACATCTAGGCAAAATGTATCAAAAATACTTCCTTGT
BlackMangabey           GAGCATAGTCTTCACTATTCACCTACATCTAGACAAAATGTATCAAAAATACTTCCTTGT
PileatedGibbon          GAGCATAGTCTTCACTATTCACCTACATCTAGACAAAATGTATCAAAAATACTTCCTTGT
SquirrelMonkey          GAGCATAGTCTTCATCATTCACCTACATCTAGACAAAATGTATCAAAAATACTTCCTTGT
Titi                    GAGCATAGTCTTCACCATTCACCTACATCTAGACAAAATGCATCAAAAATGGTTCCTTGT
Orangutan               GAGCATAGTCTTCACTATTCACCTACGTCTAGACAAAATGTATCAAAAATACTTCCTTGT
CEMacaque               GAGCATAGTCTTCACTATTCACCTACATCTAGACAAAATGTATCAAAAATACTTCCTTGT
Talapoin                GAGCATAGTCTTCACTATTCACCTACATCTAGACAAAATGTATCAAAAATACTTCCTTGT
WhiteHandedGibbon       GAGCATAGTCTTCACTATTCACCTACATCTAGACAAAATGTATCAAAAATACTTCCTTGT
Siamang                 GAGCATAGTCTTCACTATTCACCTACATCTAGACAAAATGTATCAAAAATACTTCCTTGT
WhiteCheekedGibbon      GAGCATAGTCTTCACTATTCACCTACATCTAGACAAAATGTATCAAAAATACTTCCTTGT
Colobus                 GAGCATAGTCTTCACTGTTCACCGACATCTAGACAAAATGTATCAAAAATACTTCCTTGT
OliveBaboon             GAGCATAGTCTTCACTATTCACCTACATCTAGACAAAATGTATCAAAAATACTTCCTTGT
Marmoset                GAGCATAGTCTTCACCATTCACCTATATCTAGACAAAATGTATCAAAAATACTTCCTTGT
                        **************   ****** *  *** * ******* *********  ***** **

Bonobo                  GTTGATAAGAGAAACCCAGAGCACTGTGTAAACTCAGAAATGGAAAAAACCTGCAGTAAA
BorneoOrangutan         GTTGATAAGAGAAACCCAGAGCACTGTGTAAACTCAGAAATGGAAAAAACCTGCAGTAAA
Chimpanzee              GTTGATAAGAGAAACCCAGAGCACTGTGTAAACTCAGAAATGGAAAAAACCTGCAGTAAA
RedCheekedGibbon        GTTGATAAGAGAACCCCAGAGCACTGTGTAAACTCAGAAATGGAAAAAACCTGCAGTAAA
Human                   GTTGATAAGAGAAACCCAGAGCACTGTGTAAACTCAGAAATGGAAAAAACCTGCAGTAAA
AgileGibbon             GTTGATAAGAGAACCCCAGAGCACTGTGTAAACTCAGAAATGGAAAAAACCTGCAGTAAA
Gorilla                 GTTGATAAGAGAAACCCAGAGCACTGTGTAAACTCAGAAATGGAAAAAACCTGCAGTAAA
Howler                  GGTGTTAAGAGAATCCCAGAGCACTGTGTAAACTCAGAAATGGAGGAAACCTGCAGTAAA
Rhesus                  GTTGATAAGAGAACCCCAGAGCACTGTGTAAACTCAGAAATGGAAAAAGCCTGCAGTAAA
WolfsGuenon             GTTGATAAGAGAACCCCAGAGCACTGTGTGAACTCAGAAATGGAAAAAGCCTGCAGTAAA
LeafMonkey              GTTGATAAGAGAACCCCAGAGCACTGTGTAAACTCAGAAATGGAAAAAGCCTGCAGTAAA
BlackMangabey           GTTGATAAGAGAACCCCAGAGCACTGTGTAAACTCAGAAATGGAAAAAGCCTGCAGTAAA
PileatedGibbon          GTTGATAAGAGAACCCCAGAGCACTGTGTAAACTCAGAAATGGAAAAAACCTGCAGTAAA
SquirrelMonkey          GGTGTTAAGAGAATCCCAGAGCACTGTGTAAACTCAGAAATGGAGAAAACCTGCAGTAAA
Titi                    GGTGTAAAGAGAATCCCAGAGCACTGTGTAAACTCAGAAATGGAGAAAACCTGCAGTAAA
Orangutan               GTTGATAAGAGAAACCCAGAGCACTCTGTAAACTCAGAAATGGAAAAAACCTGCAGTAAA
CEMacaque               GTTGATAAGAGAACCCCAGAGCACTGTGTAAACTCAGAAATGGAAAAAGCCTGCAGTAAA
Talapoin                GTTGATAAGAGAACCCCAGAGCACTGTGTAAACTCAGAAATGGAAAAAGCCTGCAGTAAA
WhiteHandedGibbon       GTTGATAAGAGAACCCCAGAGCACTGTGTAAACTCAGAAATGGAAAAAACCTGCAGTAAA
Siamang                 GTTGATAAGAGAACCGCAGAGCACTGTGTAAACTCAGAAATGGAAAAAACCTGCAGTAAA
WhiteCheekedGibbon      GTTGATAAGAGAACCCCAGAGCACTGTGTAAACTCAGAAATGGAAAAAACCTGCAGTAAA
Colobus                 GTTGATAAGAGAACCCCAGAGCACTGTGTAAACTCAGAAATGGAAAAAGCCTGCAGTAAA
OliveBaboon             GTTGATAAGAGAACCCCAGAGCACTGTGTAAACTCAGAAATGGAAAAAGCCTGCAGTAAA
Marmoset                GTTGTTAAGAGAAACCCAGAGCACTGTGTAAACTCAGAAATGGAGAAAACCTGCAGTAAA
                        * **  ******* * ********* *** **************  ** ***********

Bonobo                  GAATTTAAATTATCAAATAACTTAAATGTTGAAGGTGGTTCTTCAGAAAATAATCACTCT
BorneoOrangutan         GAATTTAAATTATCAAATAACTTTAATGTTGAAGGTGGTTCTTCAGAAAATAATCACTCT
Chimpanzee              GAATTTAAATTATCAAATAACTTAAATGTTGAAGGTGGTTCTTCAGAAAATAATCACTCT
RedCheekedGibbon        GAATTTAAATTATCAAATAACTTTAATGTTGAAGGTGGTTCTTCAGAAAATAATCACTCT
Human                   GAATTTAAATTATCAAATAACTTAAATGTTGAAGGTGGTTCTTCAGAAAATAATCACTCT
AgileGibbon             GAATTTAAATTATCAAATAACTTTAATGTTGAAGGTGGTTCTTCAGAAAATAATCACTCT
Gorilla                 GAATTTAAATTATCAAATAACTTAAATGTTGAAGGTGGTTCTTCAGAAAATAATCACTCT
Howler                  GAATTTAAATTATCAAATAACTTCAATGTTGAAAGTGGTTCTTCAGAAAATAATCACTCT
Rhesus                  GAATTTAATTTATCAAATAACTTTAATGTTGAAGGTGGTTCTTCAGAAAATAATCACTCT
WolfsGuenon             GAATTTAATTTGTCAGATAACTTTAATGTTGAAGGTGGTTCTTCAGAAAATAATCACTCT
LeafMonkey              GAATTTAATTTATCAAATAACTTTAATGTTGAAGGTGGTTCTTCAGAAAATAATCACTCT
BlackMangabey           GAATATAATTTATCAAATAACTTTAATGTTGAAGGTGGTTCTTCAGAAAATAATCACTCT
PileatedGibbon          GAATTTAAATTATCAAATAACTTTAATGTTGAAGGTGGTTCTTCAGAAAATAATCACTCT
SquirrelMonkey          GAATTTAAATTATCAAATAACTTAAATGTTGAAGGTGGTTCTTCAGAACATAATCACTCT
Titi                    GAATTTAAATTATCAAATAACTTTAATGTTGAAGGTGATTCTTCAGAAAATAATCACTCT
Orangutan               GAATTTAAATTATCAAATAACTTTAATGTTGAAGGTGGTTCTTCAGAAAATAATCACTCT
CEMacaque               GAATTTAATTTATCAAATAACTTTAATGTTGAAGGTGGTTCTTCAGAAAACAAT------
Talapoin                GAATTTAATTTATCAGATAACTTTAATGTTGAAGGTGGTTCTTCAGAAAATAATCACTCT
```

```
WhiteHandedGibbon       GAATTTAAATTATCAAATAACTTTAATGTTGAAGGTGGTTCTTCAGAAAATAATCACTCT
Siamang                 GAATTTAAATTATCAAATAACTTTAATGTTGAAGGTGGTTCTTCAGAAAATAATCACTCT
WhiteCheekedGibbon      GAATTTAAATTATCAAATAACTTTAATGTTGAAGGTGGTTCTTCAGAAAATAATCACTCT
Colobus                 GAATTTAATTTATCAAATAACTTTAATGTTGAAGGTGGTTTTTCAGAAAATAATCACTCT
OliveBaboon             GAATATAATTTATCAAATAACTTTAATGTTGAAGGTGGTTCTTCAGAAAATAATCACTCT
Marmoset                GAATTTAAATTATCAAATAACTTTAATATTGAAGGTGGTTCTTCAGAACATAATCAGTCT
                        **** *** ** *** ******* *** ***** *** ** ******* * ***

Bonobo                  ATTAAAGTTTCTCCATATCTCTCTCAATTTCAACAAGACAAACAACAGTTGGTATTAGGA
BorneoOrangutan         ATTAAAGTTTCTCCATATCTCTCTCAATTTCAACAAGATAAACAACAGTTGGTATTAGGA
Chimpanzee              ATTAAAGTTTCTCCATATCTCTCTCAATTTCAACAAGACAAACAACAGTTGGTATTAGGA
RedCheekedGibbon        ATTAAAGTGTCTCCATATCTCTCTCAATTTAAACAAGACAAACAACAGTTGGTATCAGGA
Human                   ATTAAAGTTTCTCCATATCTCTCTCAATTTCAACAAGACAAACAACAGTTGGTATTAGGA
AgileGibbon             ATTAAAGTGTCTCCATATCTCTCTCAATTTAAACAAGACAAACAACAGTTGGTATCAGGA
Gorilla                 ATTAAAGTTTCTCCATATCTCTCTCAATTTCAACAAGACAAACAGCAGTTGGTATTAGGA
Howler                  ATTAAAGTTTCTCCATCTCTCTCTCAGTTTAAACAAGACAAGCAACAGTTGGTATTAGGA
Rhesus                  ATTAAAGTTTCTCCATCTCTCTCTCAATTTAAACAAGACAAACAACAGTTGGTATTAGGA
WolfsGuenon             ATTAAAGTTTCTCCATATCTCTCTCAATTTAAACAAGACAAACAACAGTTGGTATTAGGA
LeafMonkey              ATTAAAGTTTCTCCATCTCTCTCTCAATTTAAACAAGACAAACAACAGTTGGTATTAGGA
BlackMangabey           ATTAAAGTTTCTCCATCTCTCTCTCAATTTAAACAAGACAAACAACAGTTGGTATTAGGA
PileatedGibbon          ATTAAAGTGTCTCCATATCTCTCTCAATTTAAACAAGACAAACAACAGTTGGTATCAGGA
SquirrelMonkey          ATGAAAGTTTCTCCATATCTCTCTCAGTTTAAACAAGACGAACAACAGTTGGTATTAGGA
Titi                    ACTAAAGTTTCTCCATATCTCTCTCAGTCTAAACAAGACAAA---CAGTTGATATTAGGA
Orangutan               ATTAAAGTTTCTCCATATCTCTCTCAATTTCAACAAGATAAACAACAGTTGGTATTAGGA
CEMacaque               ATTAAAGTTTCTCCATCTCTCTCTCAATTTAAACAAGACAAACAACAGTTGGTATTAGGA
Talapoin                ATTAAAGTTTCTCCATCTCTCTCTCAATTTAAACAAGACAAACAACAGTTGGTATTAGGA
WhiteHandedGibbon       ATTAAAGTGTCTCCATATCTCTCTCAATTTAAACAAGACAAACAACAGTTGGTATCAGGA
Siamang                 ATTAAAGTGTCTCCATATCTCTCTCAATTTAAACAAGACAAACAACAGTTGGTATCAGGA
WhiteCheekedGibbon      ATTAAAGTGTCTCCATATCTCTCTCAATTTAAACAAGACAAACAACAGTTGGTATCAGGA
Colobus                 ATTAAAGTTTCTCCATCTCTCTCTCAATTTAAACAAGACAAACAACAGTTGGTATTAGGA
OliveBaboon             ATTAAAGTTTCTCCATCTCTCTCTCAATTTAAACAAGACAAACAACAGTTGGTATTAGGA
Marmoset                ATTAAAGTTTCTCCATATCTCTCTCAGCTTAAACAAGACAAACAACAGTTGGTATTAGGC
                        *  ***** ******* *********   * *******  *    ****** *** ***

Bonobo                  ACCAAAGTCTCACTTGTTGAGAACATTCATGTTTTGGGAAAAGAACAGGCTTCACCTGAA
BorneoOrangutan         ACCAAAGTCTCACTTGTTGAGAACATTCATGTTTTGGGAAAAGAACAGACTTCACCTGAA
Chimpanzee              ACCAAAGTCTCACTTGTTGAGAACATTCATGTTTTGGGAAAAGAACAGGCTTCACCTGAA
RedCheekedGibbon        GCCAAAGTCTCACTTGTTGAAAACATTCATGTTTTGGGAAAAGAACAGGCTTCACCTGAA
Human                   ACCAAAGTGTCACTTGTTGAGAACATTCATGTTTTGGGAAAAGAACAGGCTTCACCTAAA
AgileGibbon             GCCAAAGTGTCACTTGTTGAGAACATTCATGTTTTGGGAAAAGAACAGGCTTCACCTGAA
Gorilla                 ACCAAAGTCTCACTTGTTGAGAACATTCATGTTTTGGGAAAAGAACAGGCTTCACCTGAA
Howler                  ACCGAAGTCTCACTTGTTGAAAACATTCATGTTTTGGGAAAAGAACAGGCTTCACTTGAA
Rhesus                  ACCAAAGTCTCACTTGTTGAGAACATTCATGTTTTGGGAAAAGAACAGGCTTCACCTGAA
WolfsGuenon             ACCAAAGTCTCACTTGTTGAGAACATTCATGTTTTGGGAAAAGAACAGGCTTCACCTGAA
LeafMonkey              ACCAAAGTCTCACTTGTTGAGAACATTCATGTTTTGGGAAAAGAACAGGCTTCACCTGAA
BlackMangabey           ACCAAAGTCTCACTTGTTGAGAACATTCATGTTTTGGGAAAAGAACAGGCTTCACCTGAA
PileatedGibbon          GCCAAAGTCTCACTTGTTGAGAACATTCATGTTTTGGGAAAAGAACAGGCTTCACCTGAA
SquirrelMonkey          ACCGAAGTCTCACTTGTTGAAAACATTCATGTTTTGGGAAAAGAACAGGCTTCACGTGAA
Titi                    ACTGAAGTCTCACTTGTTGAAAACATTGATGTTTTGGGAAAAGAACAGGCTTCACTTGAA
Orangutan               ACCAAAGTCTCACTTGTTGAGAACATTCATGTTTTGGGAAAAGAACAGACTTCACCTGAA
CEMacaque               ACCAAAGTCTCACTTGTTGAGAACATTCACGTTTTGGGAAAAGAACAGGCTTCACCTGAA
Talapoin                ACCAAAGGCTCACTTGTTGAGAACATTCATGTTTTGGGAAAAGAACAGGCTTCACCTGAA
WhiteHandedGibbon       GCCAAAGTCTCACTTGTTGAGAACATTCATGTTTTGGGAAAAGAACAGGCTTCACCTGAA
Siamang                 ACCAAAGTCTCACTTGTTGAGAACATTCATGTTTTGGGAAAAGAACAGGCTTCACCTGAA
WhiteCheekedGibbon      GCCAAAGTCTCACTTGTTGAAAACATTCATGTTTTGGGAAAAGAACAGGCTTCACCTGAA
Colobus                 ACCAAAGTCTCACTTGTTGAGAACATTCATGTTTTGGGAAAAGAACAGGCTTCACCTGAA
OliveBaboon             ACCAAAGTCTCACTTGTTGAGAACATTCATGTTTTGGGAAAAGAACAGGCTTCACCTGAA
Marmoset                ACCAAAGTCTCACTTGTTGAAAACATTGATGTTTTGGGAAAAGAACAGGCTTCACTTGAA
                         *  ***  *********** ****** * ****************** ****** * **

Bonobo                  AACGTAAAAATGGAAATTGGTAAAACTGAAACTTTTTCTGATGTTCCTGTGAAAACAAAT
BorneoOrangutan         AACGTAAAAATGGAAATGGGTAAAACTGAAACTTTTTCTGATGTTCCTGTGAAAACAAAT
Chimpanzee              AACGTAAAAATGGAAATTGGTAAAACTGAAACTTTTTCTGATGTTCCTGTGAAAACAAAT
RedCheekedGibbon        AATGTAAAAATGGAAATTGGTAAAACTGAAACTTTTTCTGATGTTCCTGTGAAAACAAAT
Human                   AACGTAAAAATGGAAATTGGTAAAACTGAAACTTTTTCTGATGTTCCTGTGAAAACAAAT
AgileGibbon             AACGTAAAAATGGAAATTGGTAAAACTGAAACTTTTTCTGATGTTCCTGTGAAAACAAAT
Gorilla                 AACGTAAAAATGGAAATTGGTAAAACTGAAGCTTTTTCTGATGTTCCTGTGAAAACAAAT
Howler                  AACATAAAAATGGAAATTGGTAAAACTGAAACTTTCTCTGATGTTTCTGTGAAAACAAAT
Rhesus                  AATGTAAAAATGGAAATTGGTAAAACTGAAGCTTTTTCTGATGTTCCTGTGAAAACAAAT
WolfsGuenon             AACGTAAAAATGGAAATTGGTAAAACTGAAACTTTTTCTGATGTTCCTGTGAAAACAAAT
LeafMonkey              AACGTAAAAATGGAAATTGGTAAAACTGAAGCTTTTTCTGATGTTCCTGTGAAAACAAAT
BlackMangabey           AATGTAAAAATGGAAATTGGTAAAACTGAAGCTTTTTCTGATGTTCCTGTGAAAACAAAT
PileatedGibbon          AACGTAAAAGTGGAAATTGGTAAAACTGAAACTTTTTCTGATGTTCCTGTGAAAACAAAT
```

```
SquirrelMonkey        AACATAAAAATGGAAATTGGTAAAACTGAAACTTTCTCTGATGTTTCTGTGAAAACAAAT
Titi                  AACGTAAAAATGGAAATTGGTAAAACTGAAACTTTCTCTGATGTTTCTGTGAAAACAAAT
Orangutan             AACGTAAAAATGGAAATGGGTAAAACTGAAACTTTTTCTGATGTTCCTGTGAAAACAAAT
CEMacaque             AATGTAAAAATGGAAATTGGTAAAACTGAAGCTTTTTCTGATGTTCCTGTGAAAACAAAT
Talapoin              AATGTAAAAATGGAAATTGGTAAAACTGAAGCTTTTTCTGATGTTCCTGTGAAAACAAAT
WhiteHandedGibbon     AACGTAAAAATGGAAATTGGTAAAACTGAAACTTTTTCTGATGTTCCTGTGAAAACAAAT
Siamang               AACATAAAAATGGAAATTGGTAAAACTGAAACTTTTTCTGATGTTCCTGTGAAAACAAAT
WhiteCheekedGibbon    AATGTAAAAATGGAAATTGGTAAAACTGAAACTTTTTCTGATGTTCCTGTGAAAACAAAT
Colobus               AACGTAAAAATGGAAATTGGTAAAACTGAAGCTTTTTCTGATGTTCCTGTGAAAACAAAT
OliveBaboon           AATGTAAAAATGGAAATTGGTAAAACTGAAGCTTTTTCTGATGTTCCTGTGAAAACAAAT
Marmoset              AACATAAAAATGGAAATTGGTAAAACTGAAACTTTCTCTGATGTTTCTGTGAAAACAAAT
                      **  ***** ******* ************ **** ********* **************

Bonobo                ATAGAAGTTTGTTCTACTTACTCCAAAGATTCAGAAAACTACTTTGAAACAGAAGCAGTA
BorneoOrangutan       ATAGAAGTTTGTTCTACTTACTCCAAAGATTCAGAAAACTACTTTGAAACAGAAGCAGTA
Chimpanzee            ATAGAAGTTTGTTCTACTTACTCCAAAGATTCAGAAAACTACTTTGAAACAGAAGCAGTA
RedCheekedGibbon      ACAGAAGTTTATTCTACTTACTCCAAAGATCCAGAAAACTACTTTGAAACAGAAGCAGTA
Human                 ATAGAAGTTTGTTCTACTTACTCCAAAGATTCAGAAAACTACTTTGAAACAGAAGCAGTA
AgileGibbon           ACAGAAGTTTATTCTACTTACTCCAAAGATCCAGAAAACTACTTTGAAACAGAAGCAGTA
Gorilla               ATAGAAGTTTGTTCTACTTACTCCAAAGATTCAGAAAACTACTTTGAAACAGAAGCAGTA
Howler                ATGGGAGTTTGTTCTACTTCCTTCAAAGACTCAGAAAACTACTTTGAAACAGAAGCAGTA
Rhesus                ATAGAAGTTTGTTCTACTTACTCCAAAGATTCAGAAAACTACTTTGAAACAGAAGCAGTA
WolfsGuenon           ATAGAAGTTTGTTCTACTTACTCCAAAGATTCAGAAAACTACTTTGAAACAGAAGCAGTA
LeafMonkey            ATAGAAGTTTGTTCTACTTACTCCAAAGATTCAGAAAACTACTTTGAAACAGAAGCAGTA
BlackMangabey         ATAGAAGTTTGTTCTACTTACTCCAAAGATTCAGAAAACTACTTTGAAACAGAAGCAGTA
PileatedGibbon        ACAGAAGTTTATTCTACTTACTCCAAAGATCCAGAAAACTACTTTGAAACAGAAGCAGTA
SquirrelMonkey        ACAGAATTTGGTTCTGCTTACTCCAAAGACTCAGAAAACTACTTTGAAACAGAAGCAGTA
Titi                  ATAGAAGTTTGTTCTCCTTACTCCAAAGACTCAGAAAACTACTTTGAAACAGAAGCAGTA
Orangutan             ATAGAAGTTTGTTCTACTTACTCCAAAGATTCAGAAAACTACTTTGAAACAGAAGCAGTA
CEMacaque             ATAGAAGTTTGTTCTACTTACTCCAAAGATTCAGAAAACTACTTTGAAACAGAAGCAGTA
Talapoin              ATAGAAGTTTGTTCTACTTACTCCAAAGATTCAGAAAACTACTTTGAAACAGAAGCAGTA
WhiteHandedGibbon     ACAGAAGTTTATTCTACTTACTCCAAAGATCCAGAAAACTACTTTGAAACAGAAGCAGTA
Siamang               ACAGAAGTTTATTCTACTTACTCCAAAGATCCAGAAAACTACTTTGAAACAGAAGCAGTA
WhiteCheekedGibbon    ACAGAAGTTTATTCTACTTACTCCAAAGATCCAGAAAACTACTTTGAAACAGAAGCAGTA
Colobus               ATAGAAGTTTGTTCTACTTACTCCAAAGATTCAGAAAACTACTTTGAAACAGAAGCAGTA
OliveBaboon           ATAGAAGTTTGTTCTACTTACTCCAAAGATTCAGAAAACTACTTTGAAACAGAAGCAGTA
Marmoset              ACAGAAGTTTGTTCTACTTACTCCAAAGACTCAGAAAACTGCTTTGAAACAGAAGCAGTA
                      *  * * **  **** *** ** ******  ********* *******************

Bonobo                GAAATTGCTAAAGCTTTTATGGAAGATGATGAACTGACAGATTCTGAACTGCCAAGTCAT
BorneoOrangutan       GAAATTGCTAAAGCGTTTATGGAAGATGATGAACTGACAGATTCTGAACTGCCAAGTCAT
Chimpanzee            GAAATTGCTAAAGCTTTTATGGAAGATGATGAACTGACAGATTCTGAACTGCCAAGTCAT
RedCheekedGibbon      GAAATTGCTAAAGCTTTTATGGAAGATGATGAACTGACAGATTCTGAACTGCCAAGTCAT
Human                 GAAATTGCTAAAGCTTTTATGGAAGATGATGAACTGACAGATTCTAAACTGCCAAGTCAT
AgileGibbon           GAAATTGCTAAAGCTTTTATGGAAGATGATGAACTGACAGATTCTGAACTGCCAAGTCAT
Gorilla               GAAATTGCTAAAGCTTTTATGGAAGATGATGAACTGACAGATTCTGAACTGCCAAGTCAT
Howler                GAGATTGCTAAAGCTTTTATGGAAGATGATGAACTGATAGAATCTGAAGTGCCAAGTCAT
Rhesus                GAAATTGCTAAAGCTTTTATGGAAGATGGTGAACTGACAGATTCTGAACTGCCAAGTCAT
WolfsGuenon           GAAATTGCTAAAGCTTTTATGGAAGATGATGAACTGACAGATTCTGAACTGCCAAGTCAT
LeafMonkey            GAAATTGCTAAAGCTTTTATGGAAGATGATGAACTGACAGATTCTGAACTGCCAAGTCAT
BlackMangabey         GAAATTGCTAAAGCTTTTATGGAAGATGGTGAACTGACAGATTCTGAACTGCCAAGTCTT
PileatedGibbon        GAAATTGCTAAAGCTTTTATGGAAGATGATGAACTGACAGATTCTGAACTGCCAAGTCAT
SquirrelMonkey        GAGATTGCTAAAGCTTTTATGGAAGATGATGAACTGATAGATTCTGAACTGCCAAGTCAT
Titi                  GAGATTGCTAAAGCTTTTATGGAAGATGATGAACTGATAGATTTTGAACTGCCAAGTCAT
Orangutan             GAAATTGCTAAAGCGTTTATGGAAGATGATGAACTGACAGATTCTGAACTGCCAAGTCAT
CEMacaque             GAAATTGCTAAAGCTTTTATGGAAGATGGTGAACTGACAGATTCTGAACTGCCAAGTCAT
Talapoin              GAAATTGCTAAAGCTTTTATGGAAGATAGTGAACTGACAGATTCTGAACTGCCAAGTCAT
WhiteHandedGibbon     GAAATTGCTAAAGCTTTTATGGAAGATGATGAACTGACAGATTCTGAACTGCCAAGTCAT
Siamang               GAAATTGCTAAAGCTTTTATGGAAGATGATGAACTGACAGATTCTGAACTGCCAAGTCAT
WhiteCheekedGibbon    GAAATTGCTAAAGCTTTTATGGAAGATGATGAACTGACAGATTCTGAACTGCCAAGTCAT
Colobus               GAAATTGCTAAAGCTTTTATGGAAGATGATGAACTGACAGATTCTGAACTGCCAAGTCAT
OliveBaboon           GAAATTGCTAAAGCTTTTATGGAAGATGGTGAACTGACAGATTCTGAACTGCCAAGTCAT
Marmoset              GAGATTGCTAAAGCTTTTATGGAAGATGATGAACTGATAGATTCTGAACTGCCAAGTCAT
                      ** *********** ************  ******** *** * * ** ********* *

Bonobo                GCCACACATTCTCTTTTTACATGTCCCGAAAATGAGGAAATGGTTTTGTCAAATTCAAGA
BorneoOrangutan       GCCACACATTCTCTTTTTACATGTCCCAAAAATGAGGAAATGTTTTTGTCAAATTCAAGA
Chimpanzee            GCCACACATTCTCTTTTTACATGTCCCGAAAATGAGGAAATGGTTTTGTCAAATTCAAGA
RedCheekedGibbon      GCCACACATTCTCTTTTTACGTGTCCCAAAAATGAGGAAATGGTTTTGTCAAATTCAAGA
Human                 GCCACACATTCTCTTTTTACATGTCCCGAAAATGAGGAAATGGTTTTGTCAAATTCAAGA
AgileGibbon           GCCACACATTCTCTTTTTACATGTCCCAAAAATGAGGAAATGGTTTTGTCAAATTCAAGA
Gorilla               GCCACACATTCTCTTTTTACATGTCCCGAAAATGAAGAAATGGTTTTGTCAAATTCAAGA
Howler                GCCACACACTCTCTTTTTACATGTCCCAAAAATGACGAAATGGTTTTGTCAAATTCAAGA
```

```
Rhesus                  GCCACACACTCTCTTTTTACATGTCCTCAAAATGAGGAAATGGTTTTGTCAAATTCAAGA
WolfsGuenon             GCCACACATTCTCTTTTTACATGTCCCAAAAATGAGGAAATGGTTTTGTCAAATTCAAGA
LeafMonkey              GCCACACGCTCTCTTTTTACATGTCCCCAAAATGAGGAAATGGTTTTGTCAAATTCAAGA
BlackMangabey           GCCACACACTCTCTTTTTACATGTCCTCAAAATGAGGAAATGGTTTTGTCAAATTCAAGA
PileatedGibbon          GCCACACATTCTCTTTTTACATGTCCCAAAAATGAGGAAATGGTTTTGTCAAATTCAAGA
SquirrelMonkey          GCCACGCACTCTCTTTTTACATGTCCCAAAAATGACGAAATGGTTTTGTCAAATTCAAGA
Titi                    GCCACGCACTCTCTTTTTACATGTCCCAAAAATGACAAAATGGTTTTGTCAAATTCAAGA
Orangutan               GCCACACATTCTCTTTTTACATGTCCCAAAAATGAGGAAATGTTTTTGTCAAATTCAAGA
CEMacaque               GCCACACACTCTCTTTTTACATGTCCTCAAAATGAGGAAATGGTTTTGTCAAATTCAAGA
Talapoin                GCCACACACTCTCTTTTTACATGTCCTCAAAATGAGGAAATGGTTTTGTCAAATTCAAGA
WhiteHandedGibbon       GCCACACATTCTCTTTTTACATGTCCCAAAAATGAGGAAATGGTTTTGTCAAATTCAAGA
Siamang                 GCCACACATTCT------------------------------------------------
WhiteCheekedGibbon      GCCACACATTCTCTTTTTCCGTGTCCCAAAAATGAGGAAATGGTTTTGTCAAATTCAAGA
Colobus                 GCCACACACTCTCTTTTTACATGTCCCCAAAATGAGAAAATGGTTTTGTCAAATTCAAGA
OliveBaboon             GCCACACACTCTCTTTTTACATGTCCTCAAAATGAGGAAATGGTTTTGTCAAATTCAAGA
Marmoset                GCCTCACACACTCTTATTACATGTCCCAAAAATGACGAAATGGTTTTGTCAAATTCAAGA
                        *** * *   **

Bonobo                  ATTGGAAAAAGAAGAGGAGAGCCCCTTATCTTAGTG
BorneoOrangutan         ATTGGAAAAAGAAGAGGAGAGCCCCTTATCTCAGTG
Chimpanzee              ATTGGAAAAAGAAGAGGAGAGCCCCTTATCTTAGTG
RedCheekedGibbon        ATTGGAAAAAGAAGAGGAGAGGCCCTTATCTCAGTG
Human                   ATTGGAAAAAGAAGAGGAGAGCCCCTTATCTTAGTG
AgileGibbon             ATTGGAAAAAGAAGAGGAGAGGCCCTTATCTCAGTG
Gorilla                 ACTGGGAAAAGAAGAGGAGAGCCCCTTATCTTAGTG
Howler                  ATTGGAAAAAGAAGAGGAGAGGCCATTATCTCAGTG
Rhesus                  ATTGGAAAAAGAAGAGGAGAGGCCCTTATCTCAGCG
WolfsGuenon             ATTGGAAAAAGAAGAGGAGAGGCCCTTATCTCAGTG
LeafMonkey              ATTGGAAAAAGAAGGGGAGAGGCCCTTATCTCAGCA
BlackMangabey           ATTGGAAAAAGAAGAGGAGAGGCCCTTATCTCAGCG
PileatedGibbon          ATTGGAAAAAGAAGAGGAGAGGCCCTTATCTCAGTG
SquirrelMonkey          AATGGAAAAAGAAGAGGAGAGGCCATTATCTCAGTG
Titi                    ATTGGCAAAAGAAGAGGAGAGGCCATTATCTCAGTG
Orangutan               ATTGGAAAAAGAAGAGGAGAGCCCCTTATCTCAGTG
CEMacaque               ATTGGAAAAAGAAGAGGAGAGGCCCTTATCTCAGCG
Talapoin                ATTGGAAAAAGAAGAGGAGAGGCCCTTATCTCAGCG
WhiteHandedGibbon       ATTGGAAAAAGAAGAGGAGAGCCCCTTATCTCAGTG
Siamang                 ------------------------------------
WhiteCheekedGibbon      ATTGGAAAAAGAAGAGGAGAGGCCCTTATCTCAGTG
Colobus                 ATTGGAAAAAGAAGGGGAGAGGCCCTTATCTCAGCA
OliveBaboon             ATTGGAAAAAGAAGAGGAGAGGCCCTTATCTCAGCG
Marmoset                ATTGGAAAAAGAAGAGGAGAGGCCATTATCTCAGTG
```
