# Supplementary material for: Rapid evolution of BRCA1 and BRCA2 in humans and other primates
Source: BMC Evol Biol. 2014 Jul 11;14:155. doi: 10.1186/1471-2148-14-155 (PMC4106182; doi:10.1186/1471-2148-14-155)
Supplement: Additional file 3 — Evolution of BRCA2 over the course of primate speciation. dN/dS values for each branch of the primate phylogeny were calculated using the free-ratio model in PAML [13]. Branches exhibiting dN/dS values > 1 are shown in bold italics. Dashes (-) represent branches where zero synonymous substitutions are predicted to have occurred. On these branches, dS = 0 and dN/dS can therefore not be calculated. In these instances, the numbers of non-synonymous (N) and synonymous (S) substitutions predicted to have occurred along each branch are indicated in parentheses (N:S). Of these, branches that experienced 4 or more non-synonymous changes are italicized. [file 1471-2148-14-155-S3.pdf]

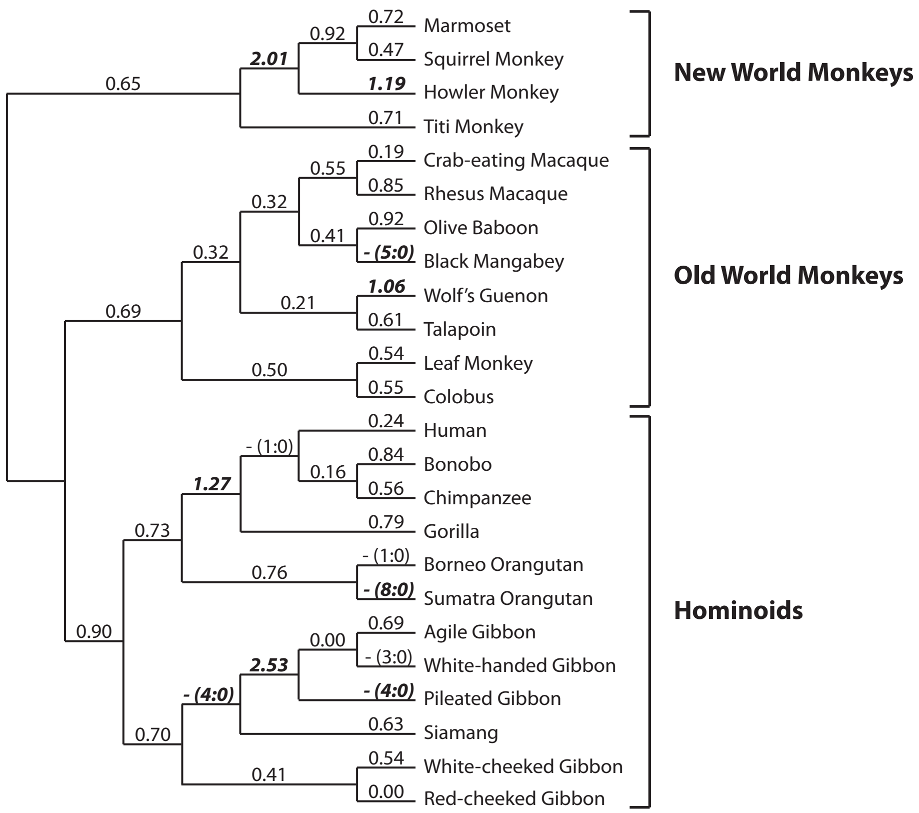

Legend for Additional File 1. Evolution of BRCA2 over the course of primate speciation. dN/dS values for each branch of the primate phylogeny were calculated using the free-ratio model in PAML [13]. Branches exhibiting dN/dS values > 1 are shown in bold italics. Dashes (-) represent branches where zero synonymous substitutions are predicted to have occurred. On these branches, dS = 0 and dN/dS can therefore not be calculated. In these instances, the numbers of non-synonymous (N) and synonymous (S) substitutions predicted to have occurred along each branch are indicated in parentheses (N:S). Of these, branches that experienced 4 or more non-synonymous changes are italicized.
