# Supplementary material for: Rapid evolution of BRCA1 and BRCA2 in humans and other primates
Source: BMC Evol Biol. 2014 Jul 11;14:155. doi: 10.1186/1471-2148-14-155 (PMC4106182; doi:10.1186/1471-2148-14-155)
Supplement: Additional file 4 — Degree of relatedness in Pan troglodyte (chimpanzee) individuals. description – sex, age, and relatedness of chimpanzee individuals used in this study. [file 1471-2148-14-155-S4.pdf]

Additional file 2. Degree of relatedness in *Pan troglodyte* (chimpanzee) individuals

Individual	Sex	Age	*Pan troglodytes* subspecies	Progeny	Parent	Half siblings
1	F	51	verus			
2	F	50				
3	F	48		34		
4	F	46		25		
5	M	46	verus			
6	M	43		20		
7	F	46				
8	M	45				
9	F	45				
10	F	42	verus	30, 27, 21		
11	F	42	verus			
12	M	42	verus			
13	F	39				
14	F	35				
15	F	31		28		16
16	F	30				15
17	M	30	verus			27
18	F	29				
19	F	29	schweinfurthii			
20	M	28			6	
21	F	27	verus		10	30, 27
22	F	26				
23	F	25				
24	M	25	verus			26, 30
25	M	23			4	
26	F	23	verus			24, 30
27	F	23	verus		10	30, 21, 17
28	M	21			15	
29	M	20				
30	M	19	verus		10	24, 26, 21, 27
31	F	19				
32	M	18				33
33	F	14				32
34	F	11			3	
