# Supplementary material for: Rapid evolution of BRCA1 and BRCA2 in humans and other primates
Source: BMC Evol Biol. 2014 Jul 11;14:155. doi: 10.1186/1471-2148-14-155 (PMC4106182; doi:10.1186/1471-2148-14-155)
Supplement: Additional file 5 — Sources and unique identifiers of Pan troglodyte genomic DNA used to generate BRCA1 exon 11 sequences. description – sources and unique identifiers of chimpanzee genomic DNA used in this study. [file 1471-2148-14-155-S5.pdf]

Additional file 3. Sources and unique identifiers of *Pan troglodyte* genomic DNA used to generate *BRCA1* exon 11 sequences

**Individual**	**Pan troglodyte subspecies**	**Source**
35	*verus*	Coriell
36	*verus*	Coriell
37	*verus*	Coriell
38	*verus*	Coriell
39	*verus*	Coriell
40	*verus*	Coriell
41	*verus*	Coriell
42	*verus*	Coriell
43	*verus*	Coriell
44	*verus*	Coriell
