# Supplementary material for: Rapid evolution of BRCA1 and BRCA2 in humans and other primates
Source: BMC Evol Biol. 2014 Jul 11;14:155. doi: 10.1186/1471-2148-14-155 (PMC4106182; doi:10.1186/1471-2148-14-155)
Supplement: Additional file 6 — Degree of relatedness in Macaca mulatta (rhesus macaque) individuals. description – relatedness of rhesus macaque individuals used in this study. [file 1471-2148-14-155-S6.pdf]

Additional file 4. Degree of relatedness in *Macaca mulatta* (rhesus macaque) individuals

Individual	Sex	Age	Progeny	Parent	Half Sibling
1	F	9			
2	F	8			7
3	F	8	24		
4	F	8		44	
5	F	8			13
6	F	8			
7	F	8			2
8	F	8			
9	F	8			
10	F	8			37
11	F	7			
12	F	7			23
13	F	6			5
14	F	6			
15	F	6			
16	F	5		41	
17	F	5			
18	F	4			
19	F	4		41	
20	F	4			
21	F	4			
22	F	4			
23	F	4			12
24	F	4		3	
25	F	4			
26	F	4			
27	F	4			28
28	F	4			27
29	F	4			
30	F	22			44
31	F	22			
32	F	20			
33	F	20			
34	F	20	40		
35	M	20			
36	F	18			
37	F	16			10
38	F	16			
39	F	15			
40	M	16		34	
41	M	15	16, 19		
42	F	15			

43	M	26			
44	M	24	5-059		30
