# Supplementary material for: Rapid evolution of BRCA1 and BRCA2 in humans and other primates
Source: BMC Evol Biol. 2014 Jul 11;14:155. doi: 10.1186/1471-2148-14-155 (PMC4106182; doi:10.1186/1471-2148-14-155)
Supplement: Additional file 7 — Pan paniscus (bonobo) individuals information. description – sex and sources of bonobo samples used in this study. [file 1471-2148-14-155-S7.pdf]

Additional file 5. *Pan paniscus* (bonobo) individuals information

Individual	Sex	Progeny	Parent	Source
1	M	NA	NA	Language Research Center
2	M	NA	NA	IPBIR
3	M	NA	NA	
4	F	NA	NA	IPBIR
5	F	NA	NA	Columbus Zoo
6	M	NA	NA	Columbus Zoo
7	F	NA	NA	
