# Supplementary material for: Rapid evolution of BRCA1 and BRCA2 in humans and other primates
Source: BMC Evol Biol. 2014 Jul 11;14:155. doi: 10.1186/1471-2148-14-155 (PMC4106182; doi:10.1186/1471-2148-14-155)
Supplement: Additional file 8 — Sources and unique identifiers of cell lines used to generate primate cDNA libraries and sequences. description – sources and unique identifiers of cell lines used in this study. [file 1471-2148-14-155-S8.pdf]

Additional file 6. Sources and unique identifiers of cell lines used to generate primate cDNA libraries and sequences

**Common Name**		**Source**	**Cell Type**
Bonobo	*Pan paniscus*	Coriell	
Borneo Orangutan	*Pongo pygmaeus*	Coriell	B-lymphocyte
Agile Gibbon	*Hylobates agilis*	Coriell	Fibroblast
White-handed Gibbon	*Hylobates lar*	Coriell	
Pileated Gibbon	*Hylobates pileatus*	Coriell	
Siamang	*Symphalangus syndactylus*	Coriell	Fibroblast
White-cheeked Gibbon	*Nomascus leucogenys*	Coriell	Fibroblast
Red-cheeked Gibbon	*Nomascus gabriellae*	Coriell	
Crab-eating Macaque	*Macaca fascicularis*	Coriell	B-lymphocyte
Olive Baboon	*Papio anubis*	Coriell	Fibroblast
Black Mangabey	*Lophocebus albigena*	Coriell	Fibroblast
Wolf's Guenon	*Cercopithecus wolfi*	Coriell	Fibroblast
Talapoin	*Miopithecus talapoin*	Coriell	Fibroblast
Leaf Monkey	*Trachypithecus francoisi*		Fibroblast
Colobus	*Colobus guereza*	Coriell	Fibroblast
Squirrel Monkey	*Saimiri sciureus*	Coriell	Fibroblast
Howler Monkey	*Alouatta sara*	Coriell	Fibroblast
Titi Monkey	*Callicebus cupreus*	Coriell	Fibroblast
