# Supplementary material for: Rapid evolution of BRCA1 and BRCA2 in humans and other primates
Source: BMC Evol Biol. 2014 Jul 11;14:155. doi: 10.1186/1471-2148-14-155 (PMC4106182; doi:10.1186/1471-2148-14-155)
Supplement: Additional file 9 — Primers used for BRCA1 amplification and sequencing. description – Primers used to amplify and sequence BRCA1. [file 1471-2148-14-155-S9.pdf]

Additional file 7. Primers used for *BRCA1* amplification and sequencing
(PCR primers), *sequencing primers
Bold = fragment cloned into TA vector before sequencing
All others sequenced directly from PCR product pool

Bonobo	(AD268/AD269) AD269*, AD277*, AD278*, AD296*, AD297*, AD303*, AD304*, AD305*<br>(AD285/AD288) AD280*, AD282*, AD284*, AD286*, AD293*, AD295*,<br>**(AD268/AD269)** M13R*<br>**(AD285/AD288)** M13F*, M13R*
Borneo Orangutan	(AD268/AD269) AD268*, AD276*, AD277*, AD278*, AD291*<br>(AD285/AD288) AD279*, AD280*, AD281*, AD292*, AD293*, AD272*<br>**(AD267/AD277)** M13F*, M13R*
Agile Gibbon	(AD268/AD279) AD276*, AD277*, AD278*, AD291*<br>(AD297/AD279) AD277*, AD278*, AD279*, AD285*, AD291*, AD297*<br>(AD292/AD288) AD271*, AD281*, AD282*, AD292*, AD293*, DL001*<br>**(AD285/AD280)** M13F*, M13R*<br>**(AD295/AD281)** M13F*, M13R*
White-handed Gibbon	(AD285/AD288) AD269*, AD271*, AD279*, AD280*, AD282*, AD286*, AD292*, AD295*, DL001*<br>**(AD284/AD269)** M13F*, AD277*, AD278*, M13R*
Pileated Gibbon	(AD285/AD288) AD269*, AD271*, AD279*, AD280*, AD282*, AD286*, AD292*, AD295*, DL001*<br>**(AD268/AD269)** M13F*, AD277*, AD278*, M13R*
Siamang	(AD268/AD279) AD276*, AD284*, AD277*, AD278*, AD291*, AD269*, AD279*<br>(AD285/AD287) AD279*, AD280*, AD281*, AD292*, AD293*, AD272*<br>(AD285/AD288) AD287*, AD288*<br>**(AD293/AD274)** M13F*, M13R*<br>**(AD268/AD279)** M13F*, AD277*, AD297*, AD303*
White-cheeked Gibbon	(AD268/AD279) AD276*, AD284*, AD277*, AD278*, AD287*, AD288*<br>(AD284/AD269) AD276*, AD277*, AD278*, AD291*<br>(AD285/AD288) AD279*, AD280*, AD281*, AD292*, AD293*, AD272*<br>**(AD268/AD277)** M13F*, M13R*
Red-cheeked Gibbon	(AD268/AD279) AD279*, AD285*, AD305*<br>(AD285/AD288) AD272*, AD279*, AD285*, AD280*, AD281*, AD282*, AD292*, AD293*, AD295*<br>**(AD268/AD279)** M13F*, AD277*, AD278*, AD279*, AD291*, AD303*, M13R*
Crab-eating Macaque	(AD268/AD269) AD276*, AD268*, AD277*, AD278*, AD291*<br>(AD285/AD287) AD279*, AD280*, AD281*, AD292*, AD282*, AD293*, AD272*<br>(AD295/AD288) DL002*, AD292*, AD282*<br>**(AD267/AD277)** M13F*, M13R*
Olive Baboon	(AD268/AD269) AD276*, AD277*, AD278*, AD291*<br>(AD285/AD287) AD279*, AD280*, AD281*, AD282*, AD292*, AD293*, AD272*
Black Mangabey	(AD268/AD269) AD276*, AD278*, AD291*<br>(AD285/AD287) AD279*, AD280*, AD281*, AD292*, AD293*, AD272*

	**(AD268/AD277)** M13F*, M13R*
Wolf’s Guenon	(AD268/AD269) AD276*, AD277*, AD278*, AD291*<br>(AD285/AD287) AD279*, AD280*, AD281*, AD282*, AD292*, AD271*, AD287*, AD293*, AD272*
Talapoin	(AD268/AD269) AD276*, AD277*, AD278*, AD269*<br>(AD285/AD287) AD279*, AD280*, AD281*, AD282*, AD292*, AD293*, AD287*, AD272*
Colobus	(AD284/AD269) AD276*, AD277*, AD278*, AD291*<br>(AD268/DL004) AD277*, AD278*, AD297*, AD303*<br>(AD285/AD287) AD279*, AD285*, AD280*, AD281*, AD292*, AD293*, AD272*<br>(DL005/DL008) AD280*, AD281*, AD282*, AD292*, AD293*, DL002*<br>**(AD297/AD278)** M13F*, M13R*
Squirrel Monkey	(AD268/AD269) AD276*, AD277*, AD278*, AD269*<br>(AD270/AD275) AD279*, AD280*, AD281*, AD282*, AD292*, AD293*, AD272*
Howler Monkey	(AD268/AD269) AD276*, AD277*, AD278*, AD291*<br>(AD270/AD275) AD279*, AD280*, AD281*, AD282*, AD292*, AD293*, AD272*
Titi Monkey	(AD268/AD269) AD276*, AD277*, AD278*, AD269*<br>(AD270/AD275) AD279*, AD280*, AD281*, AD282*, AD292*, AD293*, AD271*, AD272*
Bonobo Individuals (Exon 11)	(DL237/AD269) AD277*, AD278*, AD297*, AD290*, AD291*, AD285*, DL535*, DL626*<br>(AD286/DL240) AD270*, AD279*, AD286*, AD280*, AD295*, DL632*
Chimpanzee Individuals (Exon 11)	(DL237/AD269) AD277*, AD278*, AD290*, AD291*, DL535*<br>(AD285/DL240) AD270*, AD279*, AD280*, AD295*, DL533*, DL632*
Rhesus Individuals (Exon 11)	(DL605/AD279) AD277*, AD278*, AD291*, AD297*, DL626*<br>(AD285/DL607) AD279*, AD280*, AD285*, AD295*, DL607*, DL630*
