# Supplementary material for: Rapid evolution of BRCA1 and BRCA2 in humans and other primates
Source: BMC Evol Biol. 2014 Jul 11;14:155. doi: 10.1186/1471-2148-14-155 (PMC4106182; doi:10.1186/1471-2148-14-155)
Supplement: Additional file 10 — Sequences of primers used for BRCA1 sequencing. description – sequences of primers used to amplify and sequence BRCA1. [file 1471-2148-14-155-S10.pdf]

Additional file 8. Sequences of primers used for *BRCA1* sequencing

Primer	Orientation	Sequence (5'-3')
AD267	Forward	AAACTGCGACTGCGCGGC
AD268	Forward	GGTTTCTCAGATAACTGGGCC
AD269	Reverse	GCGCTTTGAAACCTTGAATGTATTCTGC
AD270	Forward	GAAGTTAGCACTCTAGGGAAGGC
AD271	Reverse	CCAAGGGTGAATGATGAAAGCTCC
AD272	Forward	ATGATTTTGAAGTCAGAGGAGATGTGG
AD275	Reverse	AAGGACTGAAGAGTGAGAAGCGC
AD276	Reverse	GGGTGATTTGTAACAATTCTTGATCTCCC
AD277	Reverse	GAAAACCACTCATTAACTTTCTGAATGCTGC
AD278	Reverse	GTAGAAGACTTCCTCCTCAGCC
AD279	Reverse	AAGTCCATGTTTATTTGGAGTAATGAGTCC
AD280	Reverse	CTCTTCTGAGGACTCTAATTTCTTGGC
AD281	Reverse	AGCTTCTAGTTCAGCCATTTCCTGC
AD282	Reverse	AGAGGTTGAAGATGGTATGCTGCC
AD284	Forward	CTCTGCTCTGGGTAAAGTTCATTGG
AD285	Forward	GATCTGTAGAGAGTAGCAGTATTTCATTGG
AD286	Forward	TGTGCAGCATTTGAAAACCCCAAGG
AD287	Reverse	CTCTGTACCTGTGGCTGGC
AD288	Reverse	CTCATTCTTGGGGTCCTGTGG
AD290	Forward	TAGTAGTCAGTAGAAATCTAAGCCCACC
AD291	Forward	GCAGATTTGGCAGTTCAAAAGACTCC
AD292	Forward	GGAGTTGGTCTGAGTGACAAGG
AD293	Forward	AACCAGGAGTGGAAAGGTCATCC
AD295	Forward	GGTTCCAGTGATGAAAACATTCAAGC
AD297	Forward	CTGTTTCAAACTTGCATGTGGAGCC
AD303	Forward	TATGGGCTACAGAAACCGTGCC
AD304	Reverse	CGGTTTCTGTAGCCCATACTTTGG
AD305	Reverse	GAAACTTCATCTTTTAGATGTTCAGG
DL001	Forward	CAGATGCTGAGTTTGTGTGTGAACG
DL002	Forward	CTGATAAAGCTCCAGCAGGAAATGG
DL004	Reverse	TGGTAGAAGACTTCCTCCTCAGC
DL005	Forward	ACTCCTGATGACCTGTTAGATGATGG
DL008	Reverse	GTCCGTTCACACACAAACTCAGC
DL237	Forward	CCAATCTGCTTTTGATTCACTCTTAGACG
DL240	Reverse	GATTAAATTCCTTGCTTTGGGACACTGG
DL533	Forward	CCAAAGATAATAGAAATGACACAGAAGGC
DL535	Forward	CAAATACTCATGCCAGCTCATTACAGC
DL605	Forward	TGGTTGATTTCCACCTCCAAGG
DL607	Reverse	GTCAGAGATGACATCCTAGCTGG
DL626	Reverse	CATGAATATGCCTGGTAGAAGACTTCC
DL630	Forward	GAAGTTAGCACTCTAGGGAAGGC
DL632	Forward	GCCAAGAAATTAGAGTCCTCAGAAGAG
M13F	Forward	GACGTTGTAAAACGACGGCCAG
M13R	Reverse	CAGGAAACAGCTATGACCATGATTACGC
