# Supplementary material for: Rapid evolution of BRCA1 and BRCA2 in humans and other primates
Source: BMC Evol Biol. 2014 Jul 11;14:155. doi: 10.1186/1471-2148-14-155 (PMC4106182; doi:10.1186/1471-2148-14-155)
Supplement: Additional file 11 — Primers used for BRCA2 amplification and sequencing. description – primers used to amplify and sequence BRCA2. [file 1471-2148-14-155-S11.pdf]

Additional file 9. Primers used for *BRCA2* amplification and sequencing
(PCR primers), *sequencing primer
Bold = fragment cloned into TA vector before sequencing
All others sequenced directly from PCR product pool

Howler Monkey	(DL267/DL274) DL132*, DL135*, DL137*, DL143*, DL153*, DL154*, DL201*, DL202*, DL273*<br>(DL128/DL132) DL129*<br>(DL154/DL145) DL154*, DL143*
Titi Monkey	(DL129/DL145) DL130*, DL132*, DL133*, DL135*, DL137*, DL140*, DL142*, DL143*, DL152*, DL153*<br>(DL129/DL146) DL201*, DL202*, DL203*
Squirrel Monkey	(DL129/DL145) DL131*, DL135*, DL143*, DL154*<br>(DL273/DL274) DL133*, DL134*, DL136*, DL137*, DL138*, DL201*, DL202*, DL203*<br>(DL144/DL147) DL144*, DL145*
Leaf Monkey	(DL130/DL146) DL130*, DL131*, DL135*, DL137*, DL138*<br>(DL144/DL147) DL145*
Colobus	(DL130/DL146) DL132*, DL133*, DL134*, DL135*, DL136*, DL137*, DL139*, DL142*, DL152*, DL153*, DL154*<br>(DL128/DL146) DL138*, DL143*, DL145*,
Wolf's Guenon	(DL130/DL147) DL132*, DL133*, DL134*, DL135*, DL136*, DL137*, DL139*, DL140*, DL145*, DL152*, DL154*<br>(DL250/DL253) DL202*, DL203*
Talapoin	(DL130/DL146) DL132*, DL133*, DL134*, DL135*, DL136*, DL137*, DL138*, DL139*, DL140*, DL142*, DL143*, DL145*, DL152*, DL154*
Olive Baboon	(DL129/DL145) DL133*, DL139*, DL140*, DL154*,<br>(DL128/DL145) DL130*, DL132*, DL133*, DL134*, DL135*, DL136*, DL137*, DL139*, DL140*, DL142*, DL143*, DL203*, DL204*
Black Mangabey	(DL128/DL147) DL130*, DL132*, DL133*, DL135*, DL136*, DL137*, DL139*, DL142*, DL143*, DL152*, DL153*, DL154*,
Crab-eating Macaque	(DL128/DL145) DL130*, DL132*, DL133*, DL135*, DL136*, DL137*, DL139*, DL140*, DL142*, DL152*, DL153*, DL154*<br>(DL128/DL146) DL143*, DL145*,
White-cheeked Gibbon	(DL128/DL145) DL130*, DL132*, DL133*, DL135*, DL136*, DL137*, DL139*, DL140*, DL145*, DL152*, DL153*, DL204*, DL244*
Agile Gibbon	(DL267/DL274) DL133*, DL134*, DL136*, DL137*, DL138*, DL140*, DL142*, DL143*, DL153*, DL201*, DL204*<br>(DL130/DL132) DL131*, DL267*
Siamang	(DL272/DL274) DL135*, DL202*<br>(DL129/DL136) DL130*<br>**(DL273/DL274)** M13F*, M13R*, DL134*, DL137*, DL201*, DL203*
Borneo Orangutan	(DL128/DL146) DL130*, DL132*, DL133*, DL135*, DL136*, DL137*, DL139*, DL140*, DL142*, DL152*, DL153*, DL154*, DL203*, DL242*, DL243*<br>**(DL138/DL142)** M13F*, M13R*
Gorilla	(DL128/DL146) DL130*, DL132*, DL133*, DL135*, DL136*, DL137*, DL138*, DL139*, DL140*, DL142*, DL143*, DL145*, DL152*, DL153*, DL201*, DL202*, DL204*

Pileated Gibbon	(DL130/DL146) DL132*, DL134*, DL135*, DL136*, DL137*, DL139*, DL140*, DL142*, DL143*, DL145*, DL153*, DL204*<br>(DL128/DL147) DL152*
Red-cheeked Gibbon	(DL128/DL146) DL130*, DL132*, DL133*, DL135*, DL136*, DL137*, DL139*, DL140*, DL142*, DL143*, DL145*, DL152*, DL153*, DL203*
Bonobo	(DL128/DL146) DL130*, DL131*, DL132*, DL133*, DL135*, DL136*, DL138*, DL139*, DL143*, DL152*, DL154*, DL202*
White-handed Gibbon	(DL128/DL146) DL132*, DL134*, DL135*, DL136*, DL137*, DL139*, DL142*, DL143*, DL145*, DL152*, DL153*, DL204*
