# Supplementary material for: Rapid evolution of BRCA1 and BRCA2 in humans and other primates
Source: BMC Evol Biol. 2014 Jul 11;14:155. doi: 10.1186/1471-2148-14-155 (PMC4106182; doi:10.1186/1471-2148-14-155)
Supplement: Additional file 12 — Sequences of primers used for BRCA2 sequencing. description – sequences of primers used to amplify and sequence BRCA2. [file 1471-2148-14-155-S12.pdf]

**Additional file 10. Sequences of primers used for *BRCA2* sequencing**

| Primer | Orientation | Sequence (5'-3') |
|---|---|---|
| DL128 | Forward | GAAAGTGGACTGGAAATACATACTGTTTGC |
| DL129 | Forward | CATGGAAGGAGGATACCTTATGTCC |
| DL130 | Forward | GTTCAGCCCAGTTTGAAGCAAATGC |
| DL131 | Reverse | GGAAGTAGGAGTTAAAATAAGAGTGCTGG |
| DL132 | Reverse | GGTTGAATTGTACCTTTCTTGAAGGTGATGC |
| DL133 | Forward | GTGTAAAGCAGCATCTAAAAATGACTCTAGG |
| DL134 | Forward | CCAATTTCAAATCACAGTTTTGGAGGTAGC |
| DL135 | Reverse | CTTCTTAATGTTATGTTCAGAGAGCTTGATTTCC |
| DL136 | Reverse | GCTTGCTGCTGTCTACCTGACC |
| DL137 | Forward | CCAGTTTATGAAGGAGGGAAACACTC |
| DL138 | Forward | GTTGGTACTGGAAATCAACTAGTGACC |
| DL139 | Reverse | CTTTGCCCATTGATGGCTAAAACTGG |
| DL140 | Reverse | GGTAGGAATAGCTGTTAGACATGCTAC |
| DL142 | Reverse | CTTGCTTTCCACTTGCTGTACTAAATCC |
| DL143 | Forward | CCAGAGCACTGTGTAAACTCAGAAATGG |
| DL144 | Forward | GCTTAAACAAGACAAACAACAGTTGGTATTAGG |
| DL145 | Reverse | GCTCTAAAGAAACATGATGCATAAACAATCTTCG |
| DL146 | Reverse | GGTAATCGGCTCTAAAGAAACGTGATGC |
| DL147 | Reverse | GATTTAGACAGAAATTCTTGACCAGGTGC |
| DL152 | Reverse | GCTTCTTGAGCTTTCGCAACTTCC |
| DL153 | Forward | CTATTGAGACTGTGGTGCCACC |
| DL154 | Forward | GTGGGATTTTTAGCACAGCAAGTGG |
| DL201 | Reverse | GGTCACTAGTTGATTTCCAGTACCAAC |
| DL202 | Forward | TGTGACTAGCTCTTCACCCTGC |
| DL203 | Reverse | GTAACAACCTGCCATAATTTTCGTTTGGC |
| DL204 | Forward | GGAAAATCTGTCCAGGTATCAGATGC |
| DL242 | Forward | CCTTATTCAGTCATTGAAAATTCAGCCTTAGC |
| DL243 | Reverse | GGTGAAGAGCTAGTCACAAGTTCC |
| DL244 | Forward | CCATATCTAATAGTAATAATTTTGAGGTAGGGCC |
| DL250 | Forward | CAACTGCCCCAAAGTGTAAAGAAATGC |
| DL253 | Reverse | CTGGGGTTCTCTTATCAACACAAGG |
| DL267 | Forward | AACTAGCTCTTTTGGGACAATTCTGAGG |
| DL273 | Forward | AGACACAGGTGATAAACAAGCAACCC |
| DL274 | Forward | GAGAATGTGTGGCATGACTTGGC |
| M13F | Forward | GACGTTGTAAAACGACGGCCAG |
| M13R | Reverse | CAGGAAACAGCTATGACCATGATTACGC |
